# Transport Channels of Air Pollutants Affecting the Southern Sichuan Basin Based on Gridded Dispersion Simulation

**DOI:** 10.3390/ijerph20075396

**Published:** 2023-04-04

**Authors:** Yinpeng Mo, Guangming Shi, Xia Jiang, Tianzhi Luo, Shuhua Zhou, Fumo Yang

**Affiliations:** 1 Department of Environmental Science and Engineering, Sichuan University, Chengdu 610065, China; 2 College of Carbon Neutrality Future Technology, Sichuan University, Chengdu 610065, China; 3 National Engineering Research Center on Flue Gas Desulfurization, Chengdu 610065, China; 4 Yibin Eco-environment Monitoring Station, Yibin 644002, China

**Keywords:** atmospheric transport channel, air pollution prevention, gridded dispersion simulation

## Abstract

Air pollutants suspended in the atmosphere have a large impact on air quality, climate, and human health. As one of the important populated and industrialized regions in China, the Sichuan Basin (SCB) has confronted severe air pollution in recent years. Previous studies have shown that regional transport played a significant role in the formation of regional pollution in the SCB, particularly in the southern basin. Using Yibin and Zigong as representative receptor cities, we further identified the transport channels affecting the southern basin by conducting gridded dispersion simulations. A total of seven channels were identified, including three for cyclonic transport, three through the mountainous areas between the Longquan Mountain and the Huaying Mountain, and one along the Yangtze River. Varying seasonal distributions of their occurrence frequencies were observed. Furthermore, observational evidence for several universal channels was presented during a typical transport case. The transport pathways identified in this study can guide the planning of regional distribution of emission sources and the measures for regional joint prevention and control of air pollution.

## 1. Introduction

Air pollutants suspended in the atmosphere have great impacts on air quality, climate, and human health [1,2,3]. China has confronted severe air pollution accompanied with rapid economic growth and accelerated urbanization [4]. In response, several strict emission control measures aiming to control air pollution have been issued by the central government of China in the past decade, and remarkable results have been achieved [5,6,7]. However, air pollution in China is still serious, especially in the areas with intensive human activities such as the Beijing–Tianjin–Hebei agglomeration, the Yangtze River Delta, the Pearl River Delta, and the Sichuan Basin (SCB) [8,9].

**Figure 1:**
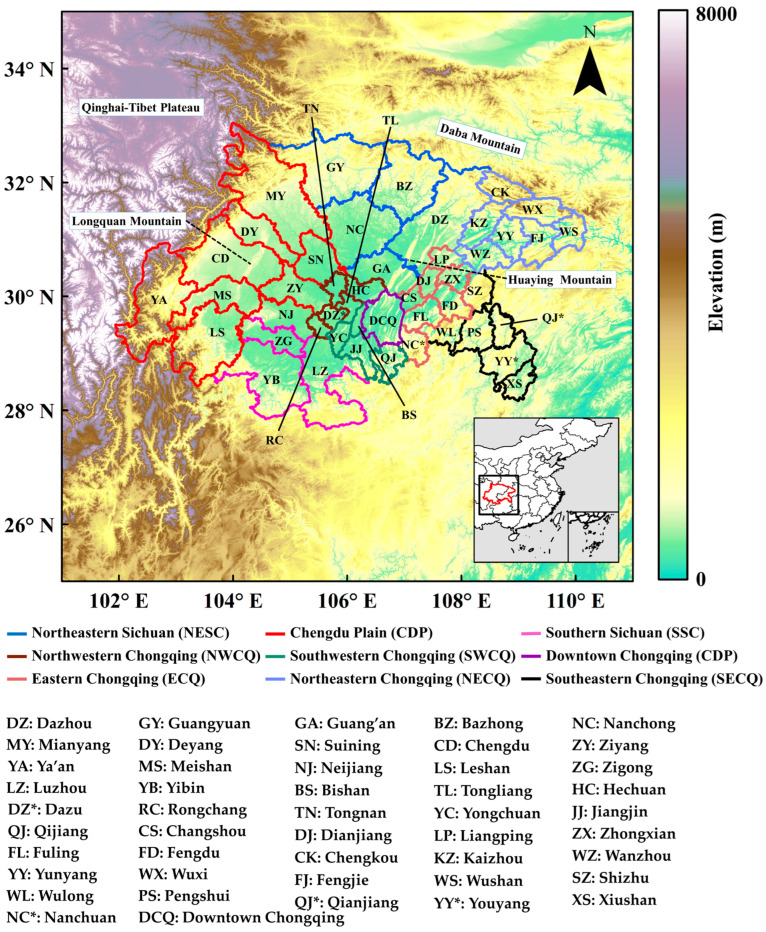
The administrative divisions and urban agglomerations in SCB. The background shadings represent the topography of this region. The black square and the red polyline in the inner subplot are the domain of the dispersion simulation and the location of SCB, respectively. The asterisks are used to distinguish the cities with the same abbreviation.

Among these urban clusters, the SCB is characterized by its complex terrain and special meteorological conditions. It is located in the east leeside of the Qinghai–Tibet Plateau and surrounded by high mountains (Figure 1). Owing to the impact of topography and atmospheric circulations [10], air pollutants emitted in the basin rarely affect the outer regions [11], though outer pollutants could be relatively easily transported into the basin [11,12]. With low wind speed and poor vertical diffusion conditions, SCB is exposed to air stagnation conditions for nearly half of the year [4]. Frequent air stagnation leads to frequent extreme haze events in the basin and the pollution usually covers a large area, forming regional pollution [11,12,13,14]. The transportation of pollutants plays a significant role in pollution processes, especially in the southern part of the basin [12,15,16,17,18,19,20]. Many studies adopted receptor-based trajectory models or chemical transport models to identify the sources and quantify the transporting contributions of pollutants to southern SCB [13,21,22,23,24]. However, the transport pathways of pollutants are still unclear, which are crucial for designing regional joint control measures.

By conducting the gridded dispersion simulation with the hybrid single particle Lagrangian integrated trajectory (HYSPLIT) model [25], we tried to identify the transport pathways of air pollutants within SCB in this study. The HYSPLIT model was driven to perform forward dispersion simulations of tracer particles over a 72 h period for cities and counties in the SCB. The distributions of tracer particles released in each city or county were clustered monthly to obtain the classified distribution patterns, and the mobility pathway of the high concentration center in each classified pattern was identified as a transport channel. The transport channels affecting the receptor cities were then obtained by integrating the pathways. The identified transport channels were expected to provide a scientific basis for the prevention and control of regional air pollution in southern SCB.

## 2. Materials and Methods

### 2.1. Study Area

The study area covered the prefecture-level cities of Sichuan Province and Chongqing Municipality in the basin area, as shown as the polyline in Figure 1. According to the topography properties and socioeconomic status [11,12,14], these cities were grouped into nine urban agglomerations, as shown in Figure 1, including Chengdu Plain (CDP), Northeastern Sichuan (NESC), Southern Sichuan (SSC), Downtown Chongqing (DCQ), Southwestern Chongqing (SWCQ), Northwestern Chongqing (NWCQ), Southeastern Chongqing (SECQ), Eastern Chongqing (ECQ), and Northeastern Chongqing (NECQ). Two cities in SSC, Yibin and Zigong, were set as the receptor cities in the dispersion simulation. Tracer particles were emitted in other urban agglomerations and tracked after emission to identify the possible transport pathways to the receptor cities.

### 2.2. Dispersion Simulation

The HYSPLIT model was used to simulate the dispersion of emitted tracer particles [25]. These HYSPLIT dispersion simulations were driven by the meteorological data fields from the WRF (weather research and forecasting) model version 4.0. The innermost WRF domain was configured to cover the SCB and the surrounding area with a horizontal resolution of 3 km and 35 vertical layers. The longwave and shortwave radiation schemes were set according to the RRTMG (rapid radiative transfer model for general circulation models) and Dudhia schemes, respectively. The Yonsei University (YSU) scheme was used for the planetary boundary layer (PBL) parameterization. For the microphysics, the Morrison two-moment scheme was adopted. NCEP FNL (National Centers for Environmental Prediction final) data with a resolution of 1° × 1° were employed as initial and boundary conditions [26]. The WRF simulation was initialized as a “cold start” at 00:00 UTC each day and ran for 36 h. The first 12 h were discarded as model spin-up time and the output for the next 24 h was retained. This process was repeated to produce continuous meteorological data fields throughout the simulation period.

The domain of dispersion simulation covered the SCB bounding between 25 and 35° N and 101 and 111° E, shown as the black square in the inner subplot of Figure 1. The tracer particles were released as area sources in each 0.25° × 0.25° grid covering the emitting cities with identical emission rate of 1 kg/h. The emission started at 00:00 each day and lasted for 24 h.

The distribution of released tracer particles was simulated between the ground and 10,000 m vertically above the ground. Tracer particle concentrations between 100–300 m and 500–750 m were extracted to represent the dispersion characteristics in the lower boundary layer (LBL) and upper boundary layer (UBL), respectively. The model output was set as gridded concentrations in the simulation domain with a resolution of 0.05° × 0.05°. The average distribution of particle concentrations between 0 and the 24th hour, the 24th and 48th hours, and the 48th and 72nd hours after the release were recorded. The dispersion simulations were carried out in January, April, July, and October 2018 to represent winter, spring, summer, and autumn, respectively.

In total, we obtained the concentration matrix with dimensions of NE × NR × NP × ND × NL after dispersion simulations. NE was the number of 0.25° × 0.25° emitting grids. NR was the number of 0.05° × 0.05° sampling grids. NP was the number of days during the simulation period (123 in this study). ND was the number of recorded temporally average concentrations (three in this study, representing the 0–24th hours, 24th–48th hours, and 48th–72nd hours, respectively). NL was the number of vertical layers (two in this study, representing 100–300 m and 500–750 m, respectively).

### 2.3. Identification of Transport Channels

Transport channels for tracer particles released in each prefecture-level city were identified using objective clustering techniques. Firstly, the concentrations due to emissions from the emitting grids covering the territory of a prefecture-level city were averaged to represent the transport of particles released in that city. Then, the PTT (T-type orthogonal rotational principal component analysis) module of the cost733class software [27] was implemented for the averaged concentrations matrix with dimensions of NR × ND every day of a certain month. The classification was performed separately for each vertical layer. The maximum number of categories in the classification was set to nine. Then, the mobility pathway of the high particle concentration zone was identified as the transport channel for each category. Finally, all of the transport channels that had the effect of transporting tracer particles to the receptor cities were extracted and their occurrence frequencies were calculated.

Taking the transport of tracer particles released in Nanchong at UBL as an example, the average concentrations for the first classified category (in total, nine categories were identified) are shown in Figure 2. The concentration values shown in the figure were normalized to the average concentration in the simulation domain and were thus in arbitrary units. In this channel, the released tracer particles travelled southward along Suining, Ziyang, Neijiang, and affected SSC, as shown in Figure 2a–c. Based on the movement path of the high concentration zone, the transport channel affecting the receptor cities could be identified as shown in Figure 2d. The average concentrations for the classified categories and the identified transport channels originating in all of the prefecture-level cities are presented in Figures S1–S232.

After identifying the transport channels originating in all of the prefecture-level cities, we manually summarized the major channels affecting the receptor cities for each urban agglomeration and concluded on the universal channels affecting SSC in the basin. The occurrence frequencies of these channels at both LBL and UBL in four seasons were estimated.

## 3. Results and Discussion

### 3.1. Transporting Characteristics of Channels Originating in Each Urban Agglomeration

**Figure 3:**
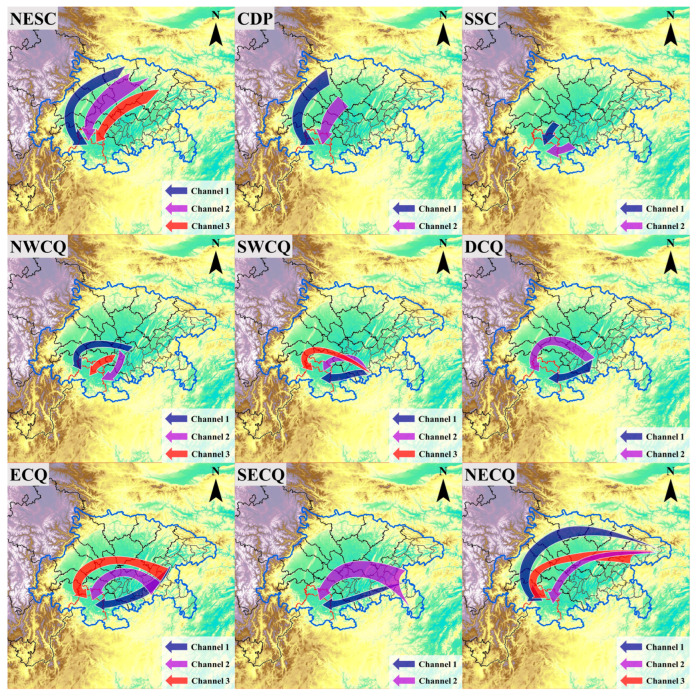
The transport channels affecting the receptor cities originating in the nine urban agglomerations.

By analyzing the transportation characteristics in Figures S1–S232, two or three main transport channels originating in different urban agglomerations and affecting the receptor cities were summarized. These channels are illustrated in Figure 3 and their pathways are described in Table 1. Tracer particles released in NESC could be transported to the receptor cities via western, middle, and eastern channels. The western channel extended westerly along the northern edge of the basin and turned southerly after entering CDP. The middle channel extended directly southwesterly along the mountainous region between the Longquan Mountain and the Huaying Mountain. In the eastern channel, tracer particles were transported along the western side of the Huaying Mountain. The particles released in CDP arrived at the receptor cities via two channels, similar to the first two channels originating in NESC, and were transported southerly along pathways to the west and east of the Longquan Mountain, respectively. For the northern part of SSC, the tracer particles were transported southerly to the receptor cities, while for the eastern part of SSC, such as Luzhou, there was a channel extending westerly along the Yangtze River.

The transport channels for NWCQ, SWCQ, and DCQ showed similar characteristics. The first channel for NWCQ extended westerly and turned south after reaching the Longquan Mountain, before affecting the receptor cities along the Yangtze River. The second channel transported the tracer particles south along the valleys and turned west after reaching the Yangtze River. The third channel represented the pathway along the western side of the Huaying Mountain. Owing to the blockage of the Huaying Mountain, the released tracer particles in DCQ could either travel south to the Yangtze River and then west along the river, or travel northwest to the eastern part of CDP through Hechuan and follow the second channel originating in CDP. As for SWCQ, the tracer particles could be transported directly to the receptor cities along the Yangtze River (the first channel). In the second channel, they could be transported northwesterly to NWCQ and then move along the third channel originating in NWCQ. Additionally, they could be transported to the east side of the Longquan Mountain by stronger east winds and turn south, heading to the receptor cities (the third channel).

The effect of tracer particles released in the east part of Chongqing on the receptor cities was less significant. The tracer particles released in ECQ and SECQ shared two similar transport channels heading to the receptor cities. The first one represented the pathway along the Yangtze River. The second one extended to the west and coincided with the third channel originating in NWCQ after reaching it. An additional channel existed in the northern region for ECQ, which ran westward to the eastern side of the Longquan Mountain through Guang’an and Ziyang, before turning south towards the receptor cities. For NECQ, the released tracer particles could be transported through channels located in northern and southern pathways. The first channel extended westward along the northern edge of the basin, then followed the first channel originating in NESC. The second and third channels extended westward along the southern pathway through Dazhou, then followed the third and second channel originating in NESC after reaching Nanchong, respectively.

### 3.2. Seasonal Distribution of Channels Originating in Each Urban Agglomeration

Typical prefecture-level cities were selected to characterize the occurrence frequencies of the identified channels originating in each urban agglomeration, and the results are presented in Table 2. For the western part of NESC, taking Guangyuan as an example, only the first and the second channels occurred and their occurrence frequencies were similar at LBL and UBL over the four seasons. The first channel occurred on 58.1% and 64.5% of winter days at LBL and UBL, respectively. It occurred at lower frequencies in spring, about 36.7% at LBL and 26.7% at UBL, respectively. Even lower frequencies were observed in summer and autumn, ranging from 9.7% to 19.4%. For the eastern part of NESC (Dazhou), the first channel occurred uniformly in winter, spring, and summer in around 10% of the days, but did not occur in autumn. The second channel occurred mainly in winter and summer, accounting for 22.6% and 6.5% at LBL and UBL in winter, respectively, and 9.7% and 12.9% at LBL and UBL in summer, respectively. In autumn, the second channel was observed only at UBL on 9.7% of the days. The third channel was absent in winter and spring and was present on 9.7% and 6.5% of the days in summer at LBL and UBL, respectively, and 16.1% of the days in autumn at LBL.

The transport characteristics of the tracer particles released in CDP varied between the western and eastern sides of the Longquan Mountain. For the western side, taking Deyang as an example, only the first channel occurred at frequencies of 60.0–73.3% in winter and spring and 25.8–41.9% in summer and autumn, respectively. For the eastern side, taking Ziyang as an example, the second channel occurred almost daily in winter at both LBL and UBL. During summer, the effect of the tracer particles released in Ziyang on the receptor cities was reduced because the second channel occurred on less than 20% of the days and the first channel was absent. In spring and autumn, tracer particles from Ziyang could be transported westward and affect the receptor cities following the first channel at occurrence frequencies of 46.7% at UBL in spring and more than 50% at both LBL and UBL in autumn, respectively. Meanwhile, the second channel occurred in 33.3–46.7% and 12.9–19.4% of the days in spring and autumn, respectively.

As adjacent regions to the receptor cities, these two cities in SSC exhibited frequent transport with variable significance in different seasons. The tracer particles from Neijiang were transported southerly along the first channel in 100%, 77.4–87.1%, 76.7–80.0%, and 71.0–74.2% of the days in winter, summer, spring, and autumn, respectively. Meanwhile, the tracer particles from Luzhou were transported westerly along the second channel at lower frequencies, at about 74.2–93.5%, 41.9–71.0%, 56.7–63.3%, and 29.0% in autumn, winter, spring, and summer, respectively.

Taking Dazu as an example of NWCQ, the tracer particles affected the receptor cities mainly along the third channel in all seasons, accounting for about two-thirds of the days in winter, half of the days in spring and autumn, and less than a quarter of the days in summer. The second channel occurred only on 22.6% of the days in autumn at LBL. The first channel was even rarer, occurring on only 9.7% of days in autumn and 3.2% of days in summer at UBL. The tracer particles from Yongchuan, an example of SWCQ, affected the receptor cities in winter, spring, and autumn. However, there was no influence in summer, except for the second channel, which occurred on 9.7% of days at UBL. The channel along the Yangtze River (the first channel) occurred at higher frequencies than the other two channels, accounting for 29.0% and 41.9% of the days in winter at LBL and UBL, respectively, and 60.0% in spring at LBL and 35.5% in autumn at both LBL and UBL. The second channel occurred less frequently, accounting for 19.4% and 12.9% of winter days at LBL and UBL, respectively, and 26.7% of spring days at UBL. The third channel occurred on 12.9% of days in winter at both LBL and UBL, and 16.7% and 32.3% of days in spring and autumn at UBL, respectively.

Regarding DCQ, the first channel occurred in winter and autumn, while the second channel was more frequent in winter and spring. Tracer particles affected the receptor cities along the Yangtze River on 25.8% of winter days at both LBL and UBL and on 48.4% and 25.8% of autumn days at LBL and UBL, respectively. The second channel accounted for 32.3%, 60.0%, 16.1% and 29.0% of days in winter, spring, summer and autumn at UBL, respectively, and 29.0% of days in winter as UBL.

Tracer particles from ECQ, SECQ, and NECQ affected the receptor cities with lower frequencies than other urban agglomerations. The first channel of ECQ occurred only in spring, accounting for 26.7% and 10.0% at LBL and UBL, respectively. The third channel occurred only in winter at frequencies of 25.8% and 9.7% at LBL and UBL, respectively. The second channel occurred on 36.7% of days in spring at LBL and on 16.1% and 12.9% of days in summer at LBL and UBL, respectively. For SECQ, the first channel occurred only in winter at a frequency of 16.1% at LBL. The second channel occurred on 67.7% of days in winter at UBL and on less than 10.0% of days in summer and autumn, but did not occur in spring. As for NECQ, the first channel occurred on 16.1% and 6.5% of days in winter at LBL and UBL, respectively, and on less than 10.0% of the days in spring and summer. However, the tracer particles were unable to reach the receptor cities along the first channel in autumn. The second channel occurred only in winter at UBL at a frequency of 22.6%. The third channel occurred in winter at LBL and in summer at UBL at frequencies of 19.4% and 9.7%, respectively.

### 3.3. The Universal Channels Affecting the Receptor Cities and Their Seasonal Characteristics

**Figure 4:**
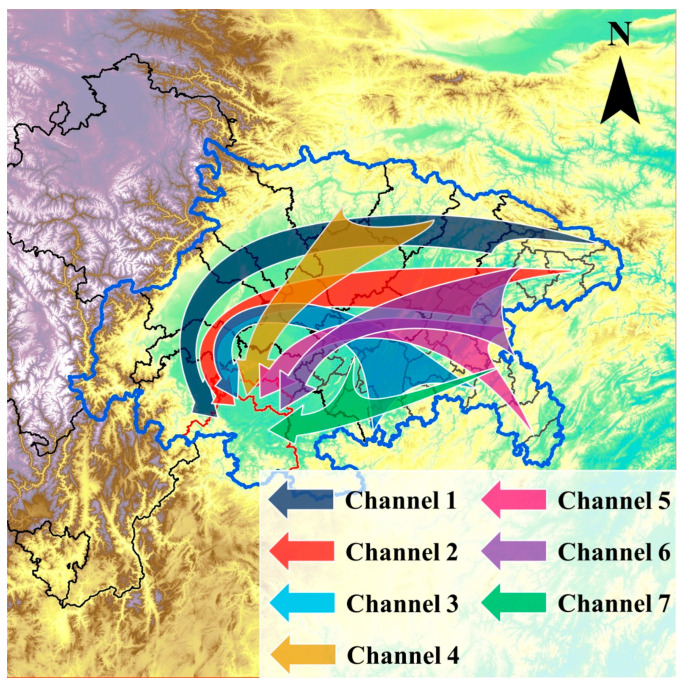
Universal transport channels affecting the receptor cities.

A total of seven universal channels affecting the receptor cities were summarized and are shown in Figure 4 by integrating the channels originating in separated urbans. The first three channels showed similar cyclonic transport characteristics originating in variant regions of the basin. The first channel originated in NECQ, extended along the northern edge of the basin, turned into CDP, and then reached the receptor cities. The second channel originated in NECQ too, but ran westward along a path south of the first channel, and turned south in the eastern side of the Longquan Mountain. The third channel originated in DCQ and SWCQ, extended northwestward, and turned south in the eastern side of the Longquan Mountain like the second channel. The fourth channel represented the southward transport originating in the northern basin and along the mountainous region between the Longquan Mountain and the Huaying Mountain. The fifth and sixth channels showed westward transport originating in SECQ and NECQ, respectively. The last channel represented the transport along the Yangtze River.

**Figure 5:**
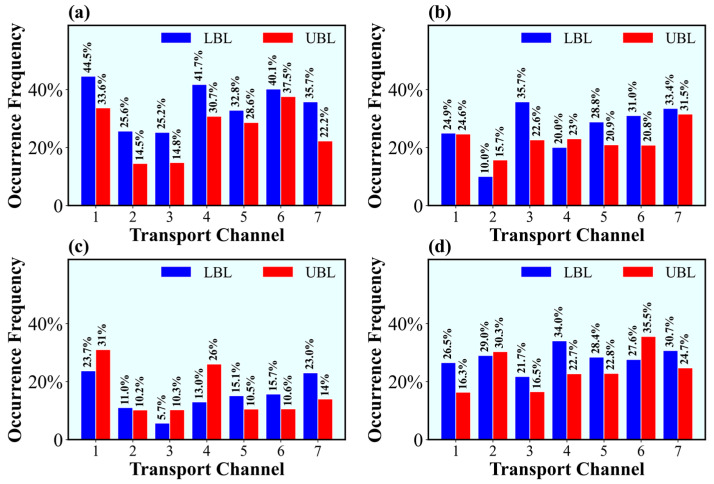
Occurrence frequencies of universal channels in (**a**) winter, (**b**) summer, (**c**) autumn, and (**d**) spring.

The occurrence frequencies of the universal channels are shown in Figure 5. A common phenomenon was that the occurrence frequencies were higher at LBL than those at UBL, revealing that the transport of tracer particles to the receptor cities occurred mostly in the lower part of the boundary layer. The receptor cities were affected by transport from other cities more frequently in winter than in other seasons. The occurrence frequencies of the universal channels varied between 14.5% and 44.5% in winter, followed by autumn and spring, with occurrence frequencies of 16.0–35.5% and 10.0–35.7%, respectively. During summer, the universal channels occurred in the lowest frequencies, ranging from 5.7% to 31.0%.

The prevailing channels varied among different seasons. In winter, driven by the prevailing invasion of air masses crossing the north, northeast, or east edge from outer regions of the basin [18], the most frequent channels included the first, fourth, sixth, and seventh channels, which occurred in excess of a third of the days. In spring, the channels originating in ECQ and SECQ were more frequent, accounting for more than 30% of the days for the third, seventh, and sixth channels. During summer, the channels originating in the northern and southeastern basin occurred more frequently, such as the first, fourth, and seventh channels. In autumn, the channels originating in the eastern basin were more prominent, especially the sixth and second channels, which accounted for 35.5% and 30.3% of the days at UBL, respectively. At the same time, the fourth channel also occurred on 34.0% of the days at LBL.

### 3.4. Typical Atmospheric Circulation Features Inducing the Universal Channels

**Figure 6:**
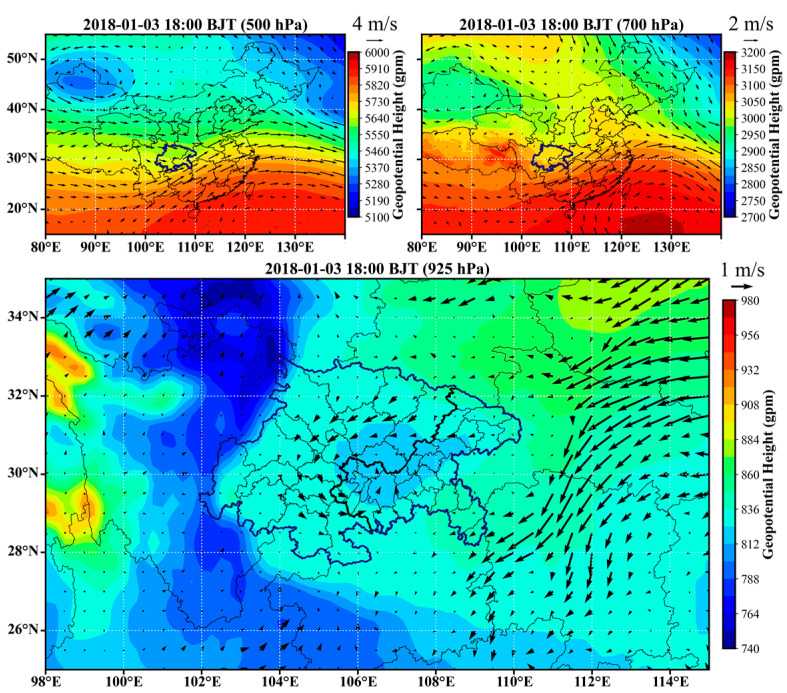
Typical circulation features in winter. The arrows represent horizontal wind vectors.

**Figure 7:**
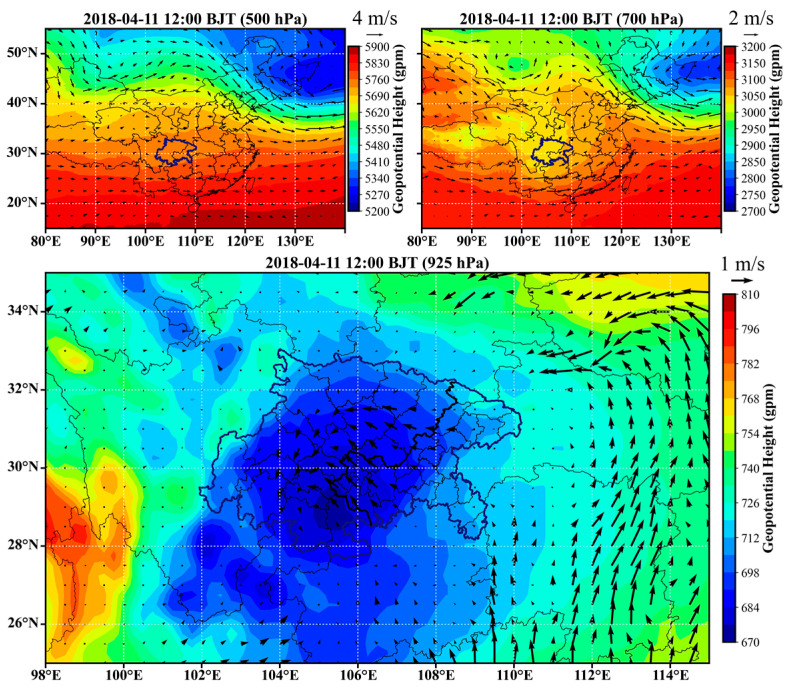
Typical circulation features in spring. The arrows represent horizontal wind vectors.

**Figure 8:**
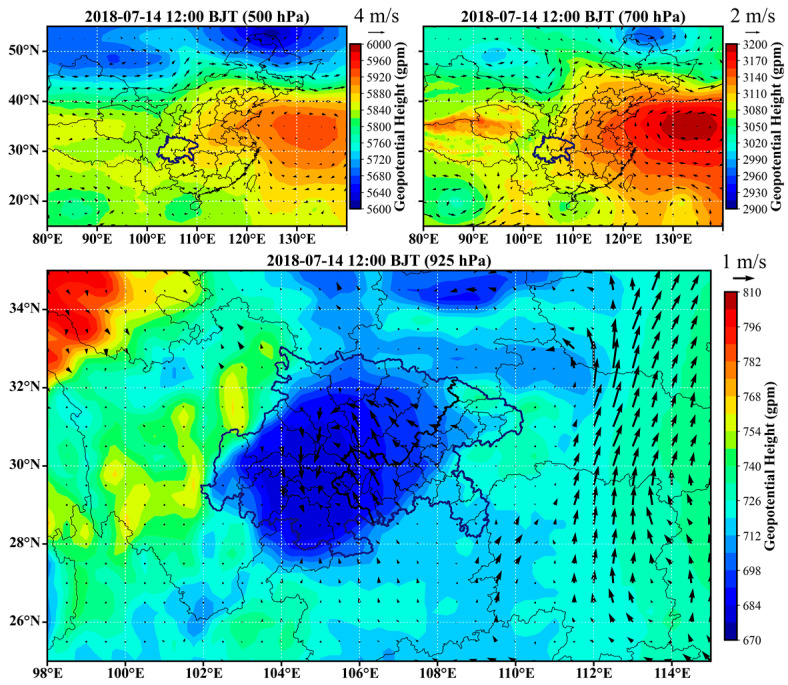
Typical circulation features in summer. The arrows represent horizontal wind vectors.

**Figure 9:**
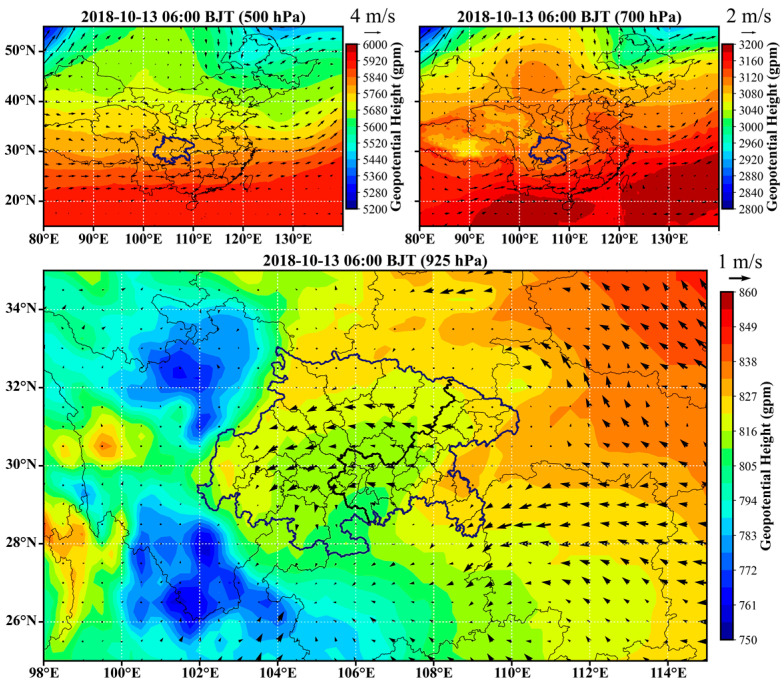
Typical circulation features in autumn. The arrows represent horizontal wind vectors.

In order to characterize the atmospheric circulations that induced the universal channels, examples were selected for each season and the circulation features are provided in Figure 6, Figure 7, Figure 8 and Figure 9. The geopotential height and horizontal wind fields were extracted from the fifth-generation ECMWF reanalysis dataset (ERA5) [28]. In winter, cold vortex dominated northeastern China, in the rear of which strong subsidence created a high-pressure system at the lower layers. As a result, the near surface layer of the SCB was generally influenced by the strong high pressure to the north or northeast and the relatively weak low pressure in the basin, as shown in Figure 6. The easterly or northeasterly winds induced by the high-pressure system entered the basin and then coupled with the cyclonic circulations induced by the low-pressure system. Consequently, the first, fourth, and sixth channels were more significant in such a circulation scenario. In spring, the vortex in northeastern China became weaker and the trough over the SCB became stronger, especially at 700 hPa, as shown in Figure 7. The basin area was controlled by a strong low-pressure system, which led to distinct cyclonic transport features, such as the third channel. In the meantime, the southeasterly winds generated the transport upstream along the Yangtze River (the seventh channel). In summer, the east to the SCB were dominated by the subtropical high, and the low-pressure system that controlled the surface layer of the SCB was located further north than in winter and spring. As shown in Figure 8, the cyclonic circulation covered the northern and western edges, in which case the first channel was dominant. An apparent northerly wind flow to the west of the low-pressure center resulted in the fourth channel. The southeasterly winds in the downstream region of the Yangtze River resulted in the seventh channel, as in spring. In autumn, the SCB was usually controlled by the flat westerlies at upper layers and was located behind a high-pressure system at the near surface layer, as shown in Figure 9. The easterly winds prevailed over most of the basin, turning northeasterly in the western basin and northerly in the southern basin. These flows were consistent with the high frequencies of the second and sixth channels.

### 3.5. A Typical Transport Case

**Figure 10:**
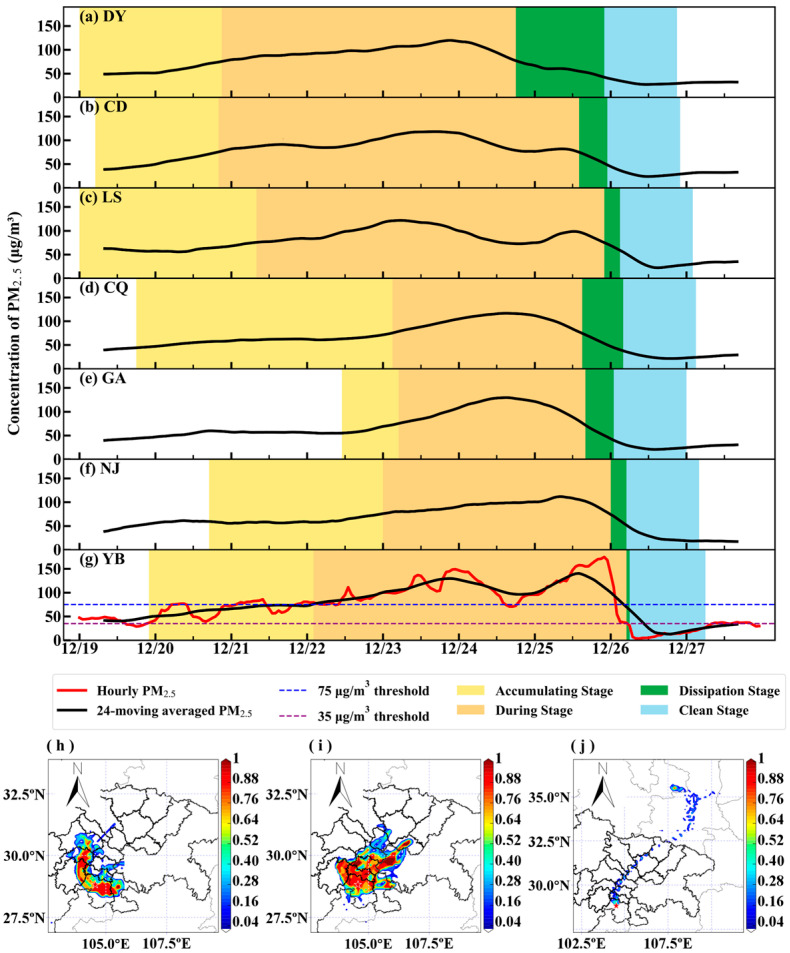
The evolution of PM_2.5_ concentrations in (**a**) Deyang, (**b**) Chengdu, (**c**) Leshan, (**d**) DCQ, (**e**) Guang’an, (**f**) Neijiang, and (**g**) Yibin, and the PSCF results of Yibin in the (**h**) accumulating stage, (**i**) during stage, and (**j**) clean stage.

A PM_2.5_ pollution event that occurred during December 19–27 in 2019 was analyzed to partly validate the identified universal channels. The whole period was divided into three stages, namely, the accumulating stage, during stage, and dissipation stage, respectively, by adopting the method proposed by Zheng et al. [29] The evolution of this event in Yibin and several other cities is presented in Figure 10, along with the results of the potential source contribution function (PSCF) analysis [30] driven by the WRF meteorological fields described in Section 2.2. In the accumulating stage, the transport pathway characterized by PSCF coincided with the first universal channel. The fact that cities along the transport channel (Deyang, Chengdu, Meishan, Leshan, and Yibin) started the accumulating stage successively provided evidence for the existence of this channel. The second, fifth, and sixth universal channels dominated the transport in the during stage, and the sixth channel dominated after the pollution event. Similarly, these PSCF results were in agreement with observations. Cities in the upwind regions, such as DCQ, Guang’an, and Neijiang, started the dissipation stages successively. As a result of the transport of pollutants, the PM_2.5_ concentration in Yibin increased rapidly before the dissipation stage.

## 4. Conclusions

The transport channels affecting the southern SCB were identified by performing gridded dispersion simulations. The first three channels presented cyclonic transport characteristics originating in the east part of the basin and extending along the rim of the basin or the eastern side of the Longquan Mountain. The first channel occurred frequently in all seasons. The second channel occurred at lower frequencies in spring and summer. The third channel was more common in spring. The fourth, fifth, and sixth channels represented the pathways through the mountainous region between the Longquan Mountain and the Huaying Mountain, originating in the northern, eastern, and southeastern basin, respectively. These channels showed relatively high frequencies in all seasons, except for the fifth and sixth channels in summer. In addition, the seventh channel depicted the transport of tracer particles to the upstream region along the Yangtze River.

The transport characteristics summarized in this study were consistent with previous studies based on the air quality observations and wind fields [12,31]. We further identified the transport pathways from dispersion simulations. The method we proposed for finding the transport pathways may be useful in other regions. The results of this study may have application potential in regional emission source distribution planning and regional joint prevention and control of air pollution. Although we presented evidence for several universal channels during a typical transport case, the validation of the simulation results and quantification of the transport effects are currently lacking. Thus, integrated observations and chemical transport modeling are required to thoroughly characterize the transport of air pollutants in the SCB.

## Figures and Tables

**Figure 2:**
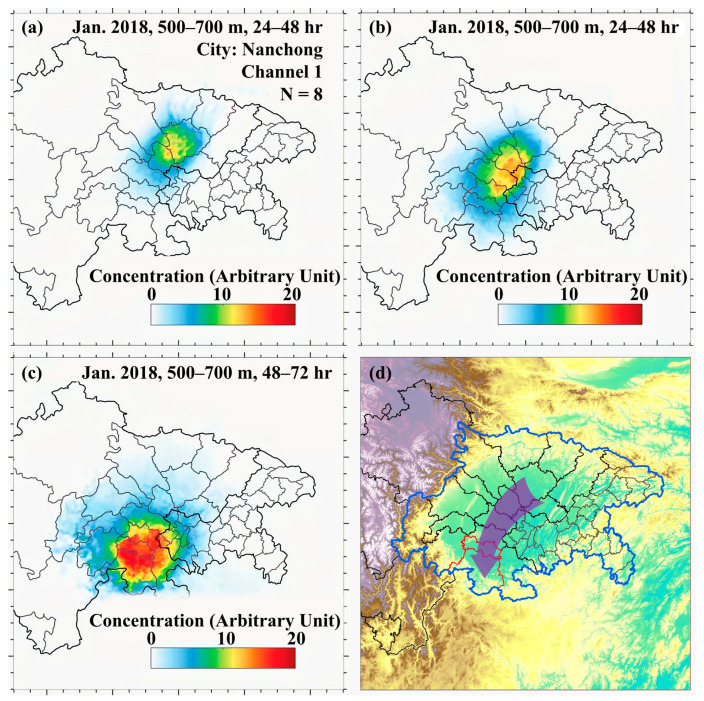
The distributions of tracer particle concentrations between (**a**) 0 and the 24th hour, (**b**) the 24th and 48th hours, (**c**) the 48th and 72nd hours since the release of tracer particles, and (**d**) the identified transport channel for the first classified category from clustering analysis in Nanchong.

**Table 1:** Transport channels of tracer particulates affecting the receptor cities originating in different urban agglomerations of SCB.

Region	Channel Number	Path
NES	1	Guangyuan → Mianyang → Deyang → Chengdu →Meishan, Ya’an → Leshan → RCs
	2	Guangyuan, Bazhong → Nanchong → Suining → Ziyang, Chengdu → Neijiang, Meishan → RCs
	3	Dazhou → Nanchong → Suining → Ziyang, Tongnan → Neijiang, Rongchang, Dazu → RCs
CP	1	Mianyang → Deyang → Chengdu → Meishan, Ya’an →Leshan → RCs
	2	Suining → Ziyang, Chengdu → Neijiang, Meishan → RCs
SS	1	Neijiang → RCs
	2	Luzhou → RCs
NWC	1	Hechuan → Tongnan →Ziyang → Chengdu → Meishan → Leshan → RCs
	2	Tongnan, Hechuan → Tongliang → Yongchuan, Dazu, Rongchang, Bishan → Jiangjin → Luzhou → RCs
	3	Tongnan → Ziyang → Neijiang, Dazu, Rongchang → RCs
SWC	1	Qijiang → Jiangjin, Bishan → Yongchuan, Luzhou → RCs
	2	Qijiang → Jiangjin, DCQ → Yongchuan, Bishan → Rongchang, Dazu → Neijiang, Ziyang, Suining → RCs
	3	Qijiang → Jiangjin, DCQ → Yongchuan, Bishan → Neijiang → Meishan → Leshan → RCs
DCQ	1	DCQ → Qijiang, Jiangjin, Bishan → Yongchuan, Luzhou → RCs
	2	DCQ → Bishan, Hechuan → Tongliang, Tongnan → Ziyang, Suining → Chengdu, Meishan, Neijiang → Leshan → RCs
EC	1	Zhongxian → Fengdu → Fuling → DCQ, Nanchuan → Qijiang, Jiangjin → Yongchuan, Luzhou → RCs
	2	Nanchuan, Fuling, Fengdu, Shizhu → Changshou, Dianjiang → DCQ, Guang’an → Hechuan, Nanchong → Tongnan, Suining → Ziyang → Neijiang → RCs
	3	Nanchuan, Fuling, Fengdu, Shizhu → Changshou, Dianjiang → DCQ, Guang’an → Hechuan, Nanchong → Suining → Deyang, Chengdu → Ziyang, Meishan → Leshan → RCs
SEC	1	Xiushan → Youyang → Pengshui, Qianjiang → Wulong, Fuling → DCQ, Nanchuan → Qijiang, Jiangjin → Yongchuan, Luzhou → RCs
	2	Xiushan → Youyang → Pengshui, Qianjiang → Fengdu, Fuling → Changshou, Dianjiang → Guang’an, Hechuan → Tongnan, Suining → Ziyang → Neijiang → RCs
NEC	1	Wushan → Fengjie → Yunyang → Kaizhou, Wanzhou → Dazhou → Bazhong, Nanchong → Guangyuan → Mianyang → Deyang, Suining → Chengdu → Meishan → Leshan → RCs
	2	Wushan → Fengjie → Yunyang → Kaizhou, Wanzhou → Dazhou → Guang’an → Nanchong → Suining → Ziyang, Tongnan → Neijiang, Rongchang, Dazu → RCs
	3	Wushan → Fengjie → Yunyang → Kaizhou, Wanzhou → Dazhou → Nanchong, Guang’an → Suining → Ziyang → Meishan → Leshan → RCs

**Table 2:** The occurrence frequencies of transport channels affecting the receptor cities originating in representative cities of each urban agglomeration.

Urban Agglomeration/Representative City	Channel	Layer	Season			
			Winter	Spring	Summer	Autumn
NESC/Guangyuan	1	LBL	58.1%	36.7%	19.4%	12.9%
	2	UBL	64.5%	26.7%	12.9%	9.7%
	2	LBL	/	16.7%	9.7%	12.9%
		UBL	/	10.0%	9.7%	9.7%
NESC/Dazhou	1	LBL	9.7%	10.0%	6.5%	/
		UBL	12.9%	6.7%	6.5%	/
	2	LBL	22.6%	/	9.7%	/
		UBL	6.5%	/	12.9%	9.7%
	3	LBL	/	/	9.7%	16.1%
		UBL	/	/	6.5%	/
CDP/Deyang	1	LBL	71.0%	73.3%	29.0%	41.9%
		UBL	61.3%	60.0%	32.3%	25.8%
	2	LBL	/	/	/	/
		UBL	/	/	/	/
CDP/Ziyang	1	LBL	/	/	/	58.1%
		UBL	/	46.7%	/	51.6%
	2	LBL	100.0%	33.3%	19.4%	12.9%
		UBL	100.0%	46.7%	16.1%	19.4%
SSC/Neijiang	1	LBL	96.8%	76.7%	87.1%	74.2%
		UBL	100.0%	80.0%	77.4%	71.0%
SSC/Luzhou	2	LBL	71.0%	56.7%	29.0%	93.5%
		UBL	41.9%	63.3%	29.0%	74.2%
NWCQ/Dazu	1	LBL	/	/	/	/
		UBL	/	/	3.2%	9.7%
	2	LBL	/	/	/	22.6%
		UBL	/	/	/	/
	3	LBL	71.0%	50.0%	9.7%	41.9%
		UBL	64.5%	53.3%	22.6%	51.6%
SWCQ/Yongchuan	1	LBL	29.0%	60.0%	/	35.5%
		UBL	41.9%	/	/	35.5%
	2	LBL	19.4%	/	/	/
		UBL	12.9%	26.7%	9.7%	/
	3	LBL	12.9%	/	/	/
		UBL	12.9%	16.7%	/	32.3%
DCQ/DCQ	1	LBL	25.8%	/	/	48.4%
		UBL	25.8%	/	/	25.8%
	2	LBL	29.0%	/	/	/
		UBL	32.3%	60.0%	16.1%	29.0%
ECQ/Changshou	1	LBL	/	26.7%	/	/
		UBL	/	10.0%	/	/
	2	LBL	/	36.7%	16.1%	/
		UBL	/	/	12.9%	/
	3	LBL	25.8%	/	/	/
		UBL	9.7%	/	/	/
SECQ/Wulong	1	LBL	16.1%	/	/	/
		UBL	/	/	/	/
	2	LBL	/	/	6.5%	/
		UBL	67.7%	/	9.7%	9.7%
NECQ/Kaizhou	1	LBL	16.1%	10.0%	6.5%	/
		UBL	6.5%	10.0%	9.7%	/
	2	LBL	/	/	/	/
		UBL	22.6%	/	/	/
	3	LBL	19.4%	/	/	/
		UBL	/	/	9.7%	/

## Data Availability

The data presented in this study are available on request from the corresponding author.

## Supplementary Materials

The following supporting information can be downloaded at https://www.mdpi.com/article/10.3390/ijerph20075396/s1. Figures S1–S232: The identified channels originating in each prefecture-level city; Tables S1–S43: The seasonal occurrence frequencies of each channel originating in each prefecture-level city.
