# Supplementary material for: Transport Channels of Air Pollutants Affecting the Southern Sichuan Basin Based on Gridded Dispersion Simulation
Source: Int J Environ Res Public Health. 2023 Apr 4;20(7):5396. doi: 10.3390/ijerph20075396 (PMC10094188; doi:10.3390/ijerph20075396)
Supplement: Supplementary file 1 [file ijerph-20-05396-s001.zip › ijerph-2250362-supplementary.pdf]

Supplementary for

Transport channels of air pollutants affecting the southern Sichuan Basin informed from gridded dispersion simulation

Yinpeng Mo [1,2], Guangming Shi [2,3,*], Xia Jiang [4], Tianzhi Luo [4], Shuhua Zhou [4] and Fumo Yang [2,3]

[1] Department of Environmental Science and Engineering, Sichuan University, Chengdu 610065, Sichuan, China
[2] College of Carbon Neutrality Future Technology, Sichuan University, Chengdu 610065, Sichuan, China
[3] National Engineering Research Center on Flue Gas Desulfurization, Chengdu 610065, Sichuan, China
[4] Yibin Eco-environment Monitoring Station, Yibin 644002, Sichuan, China
[*] Correspondence: shigm@scu.edu.cn

In these supplementary materials, we presented the identified channels originating in all the prefecture-level cities in Figure S1–S232 and their seasonal occurrence frequencies in Table S1–S43.

1. Northeast Sichuan Urban Agglomeration

1.1 Dazhou

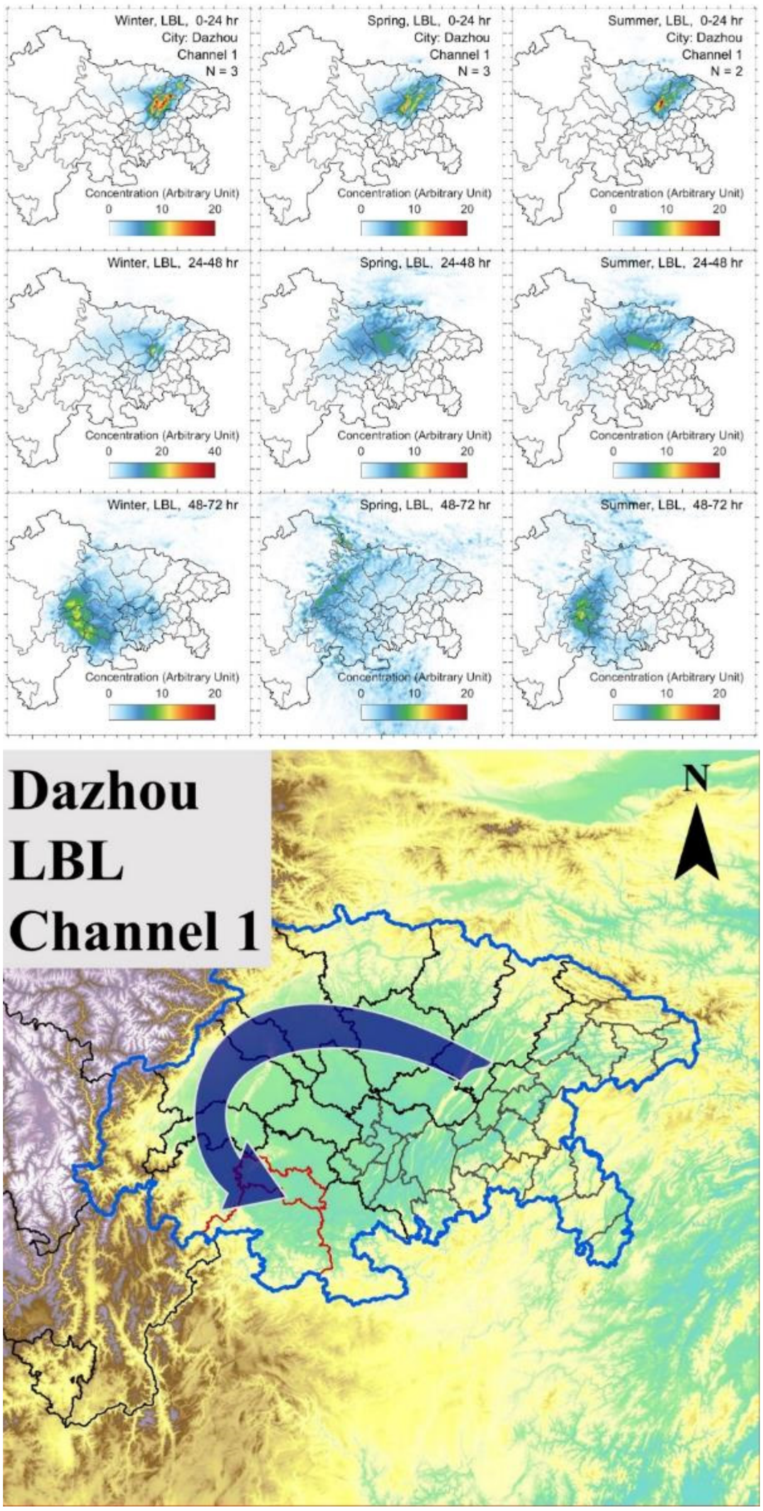

Figure S1 Channel 1 originating in Dazhou at LBL.

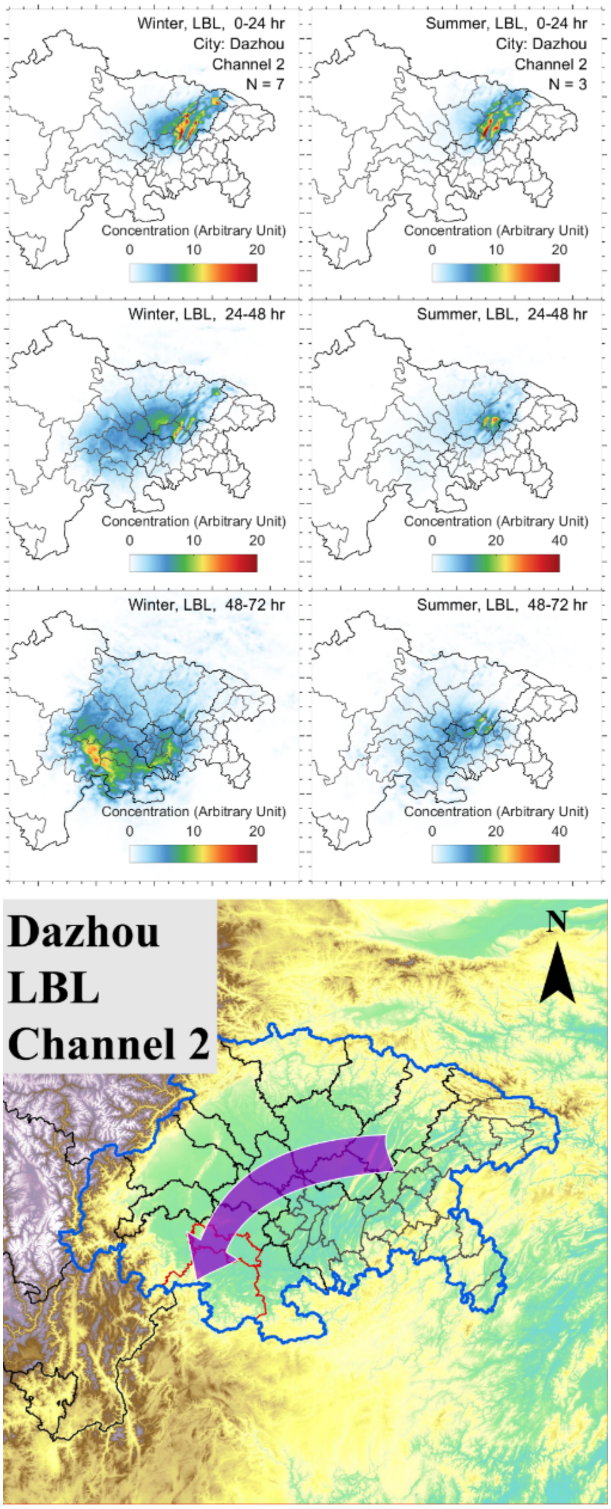

Figure S2 Channel 2 of Dazhou at LBL.

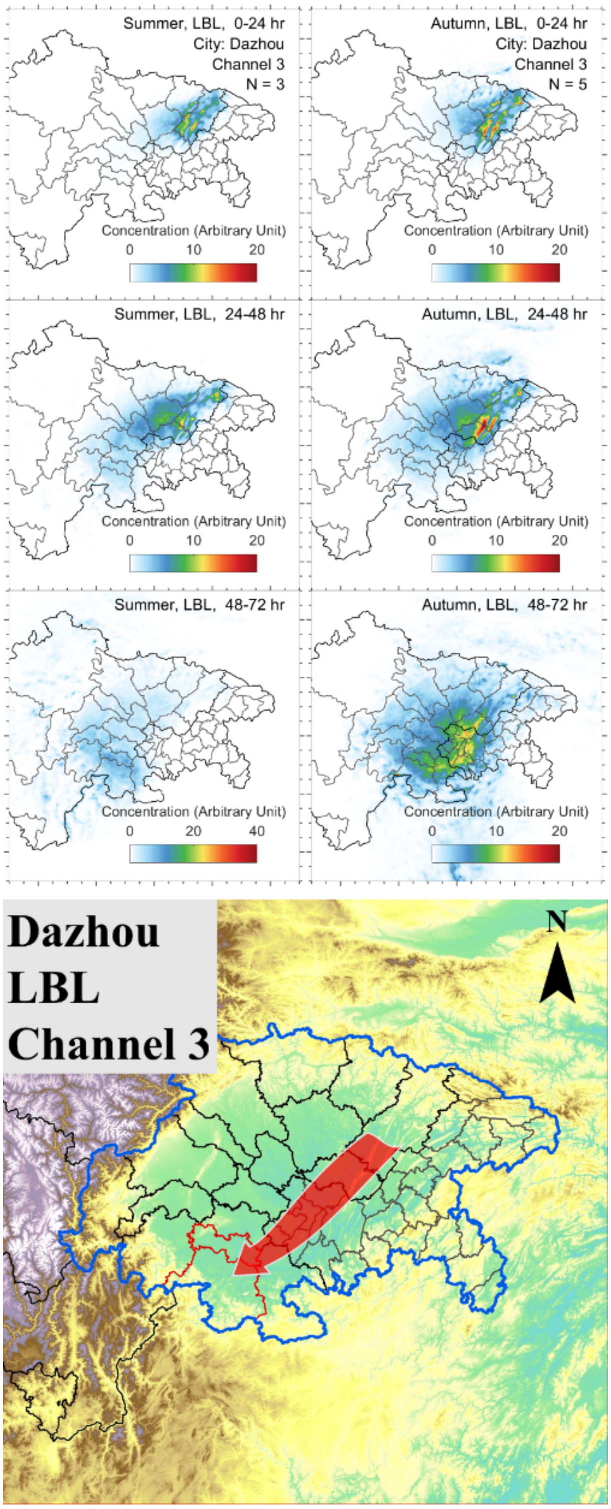

Figure S3 Channel 3 of Dazhou at LBL.

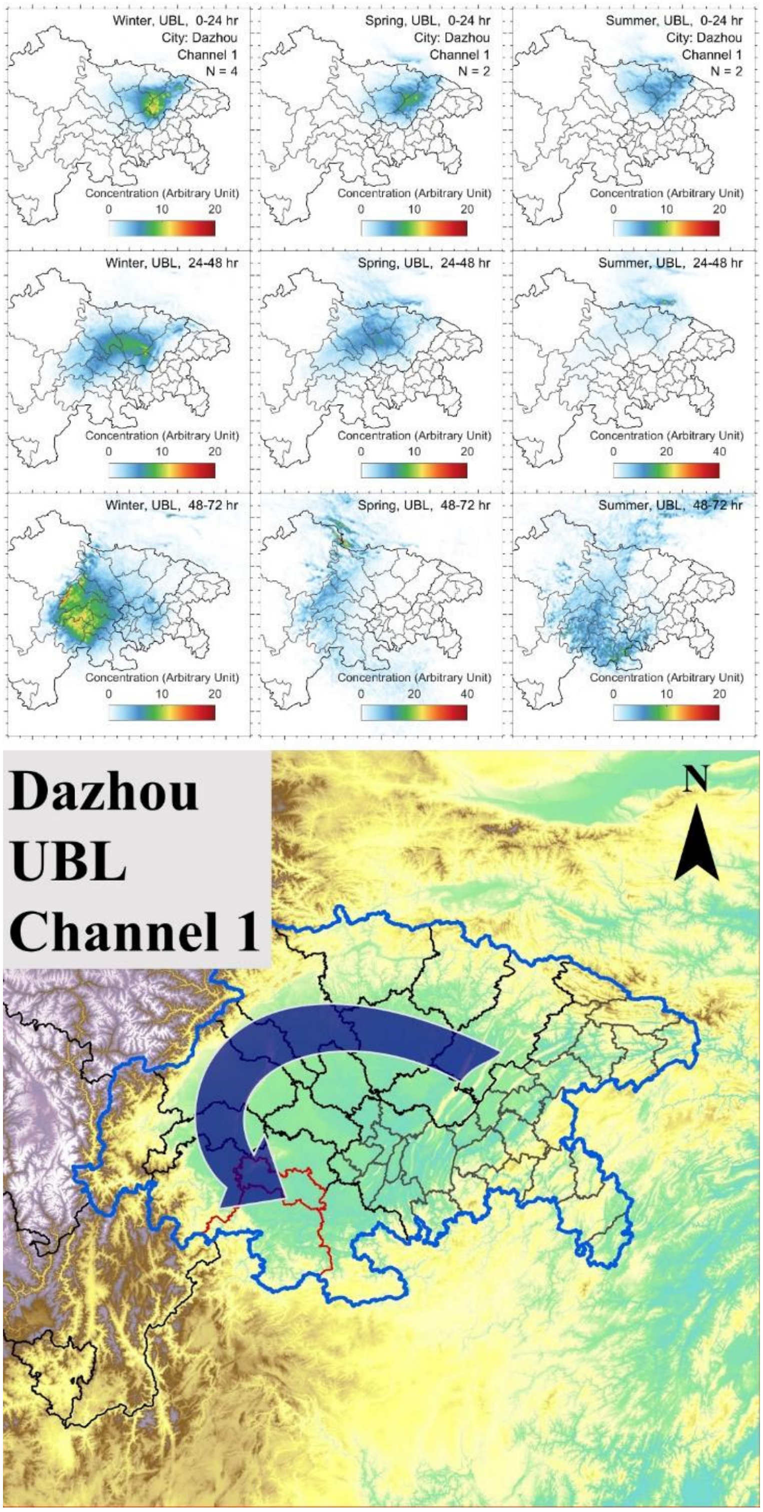

Figure S4 Channel 1 of Dazhou at UBL.

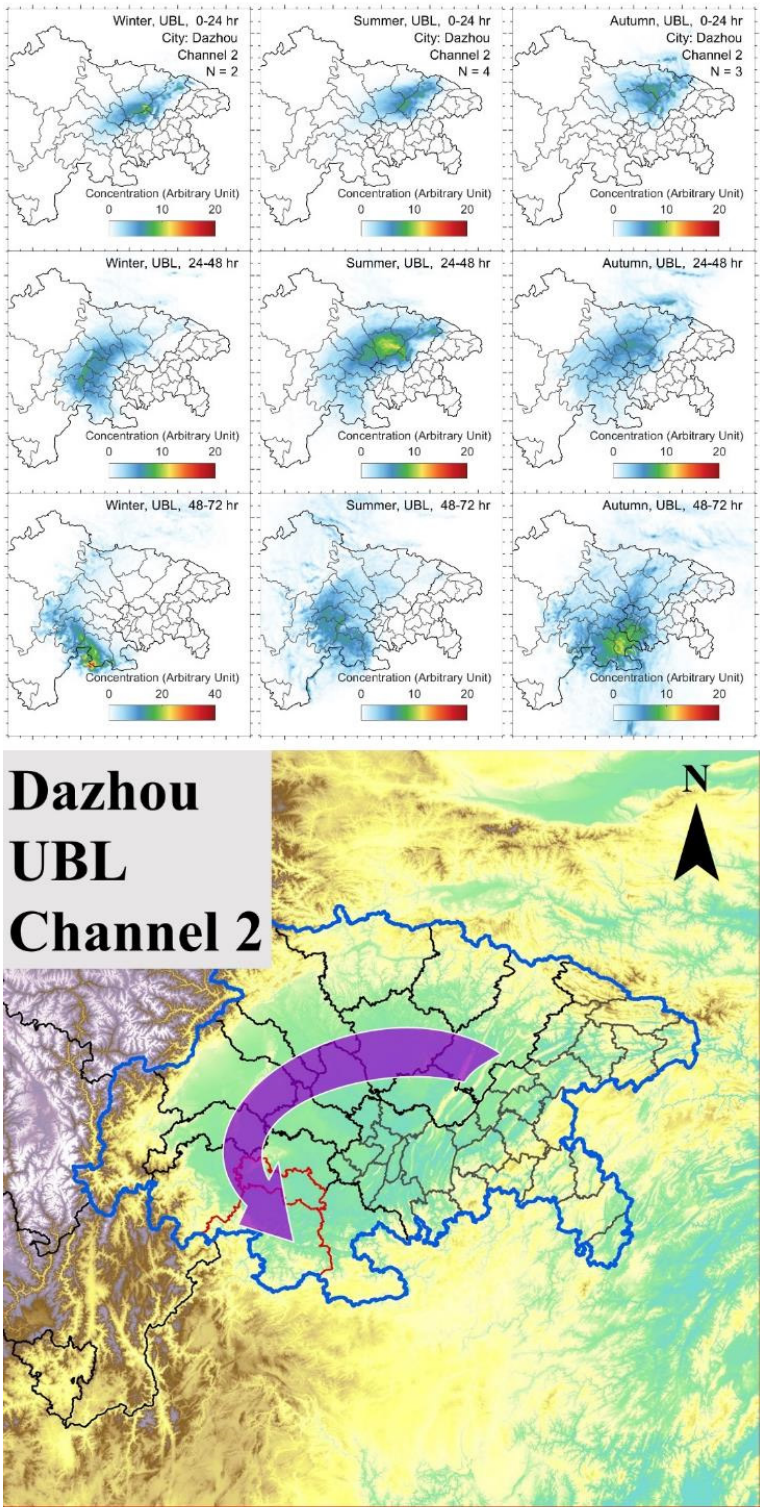

Figure S5 Channel 2 of Dazhou at UBL.

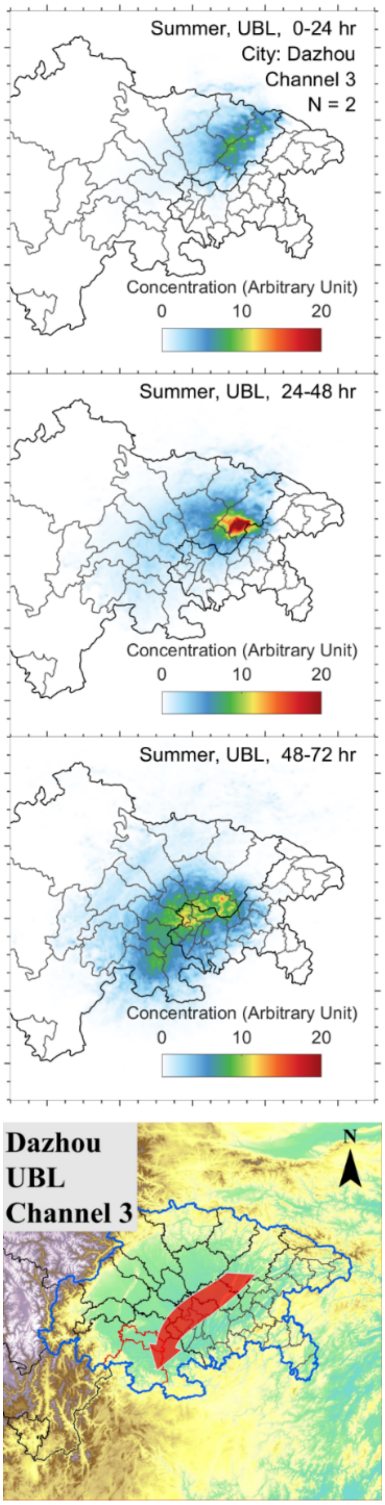

Figure S6 Channel 3 of Dazhou at UBL.

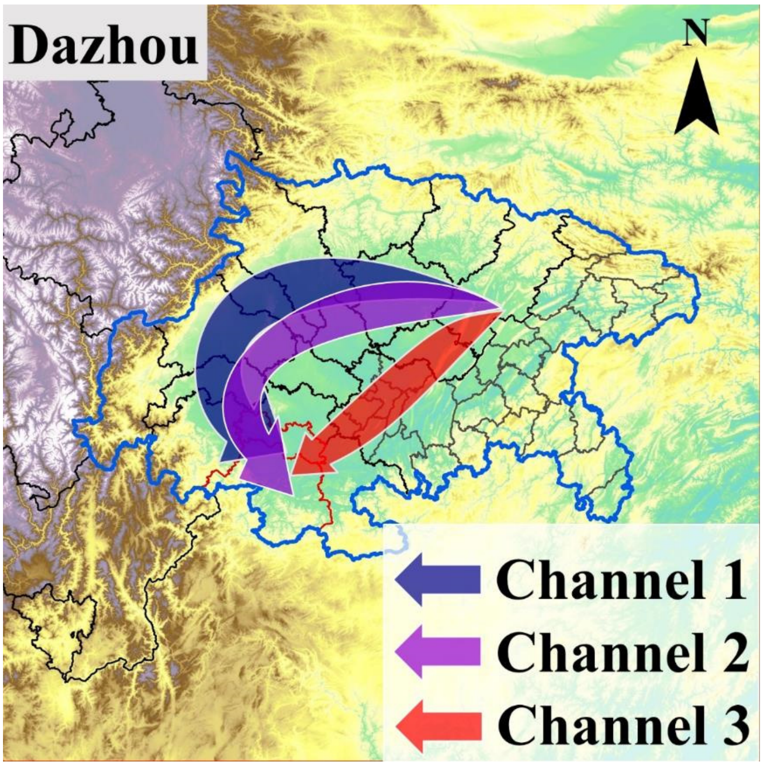

Figure S7 The identified 3 channels originating in Dazhou.

Table S1 Occurrence frequencies of each channel originating in Dazhou in four seasons.

City	Channel	Layer	Season			
			autumn	spring	summer	winter
Dazhou	1	LBL		10.0%	6.5%	9.7%
		UBL		6.7%	6.5%	12.9%
	2	LBL			9.7%	22.6%
		UBL	9.7%		12.9%	6.5%
	3	LBL	16.1%		9.7%	
		UBL			6.5%	

1.2 Bazhong

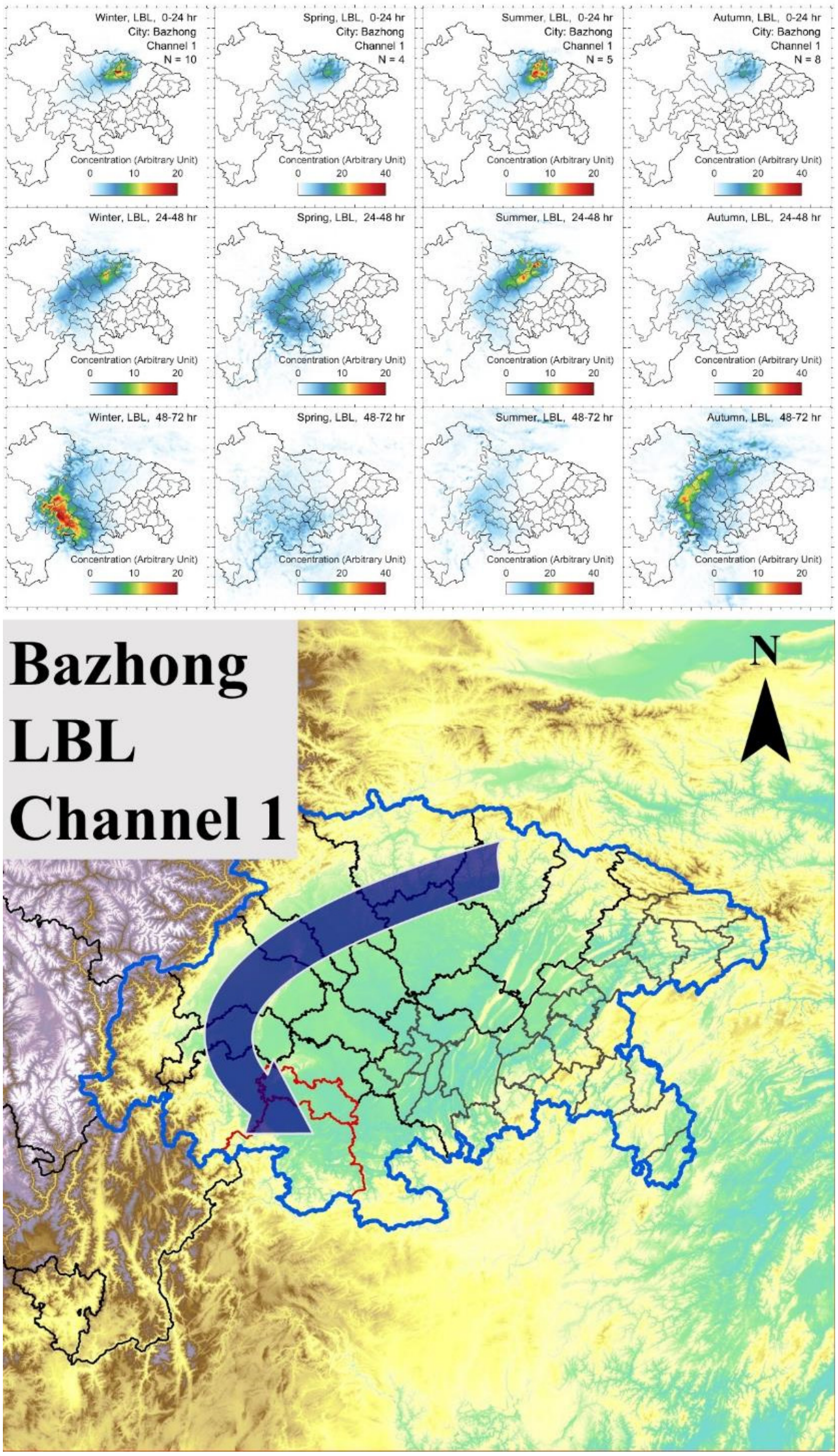

Figure S8 Channel 1 of Bazhong at LBL.

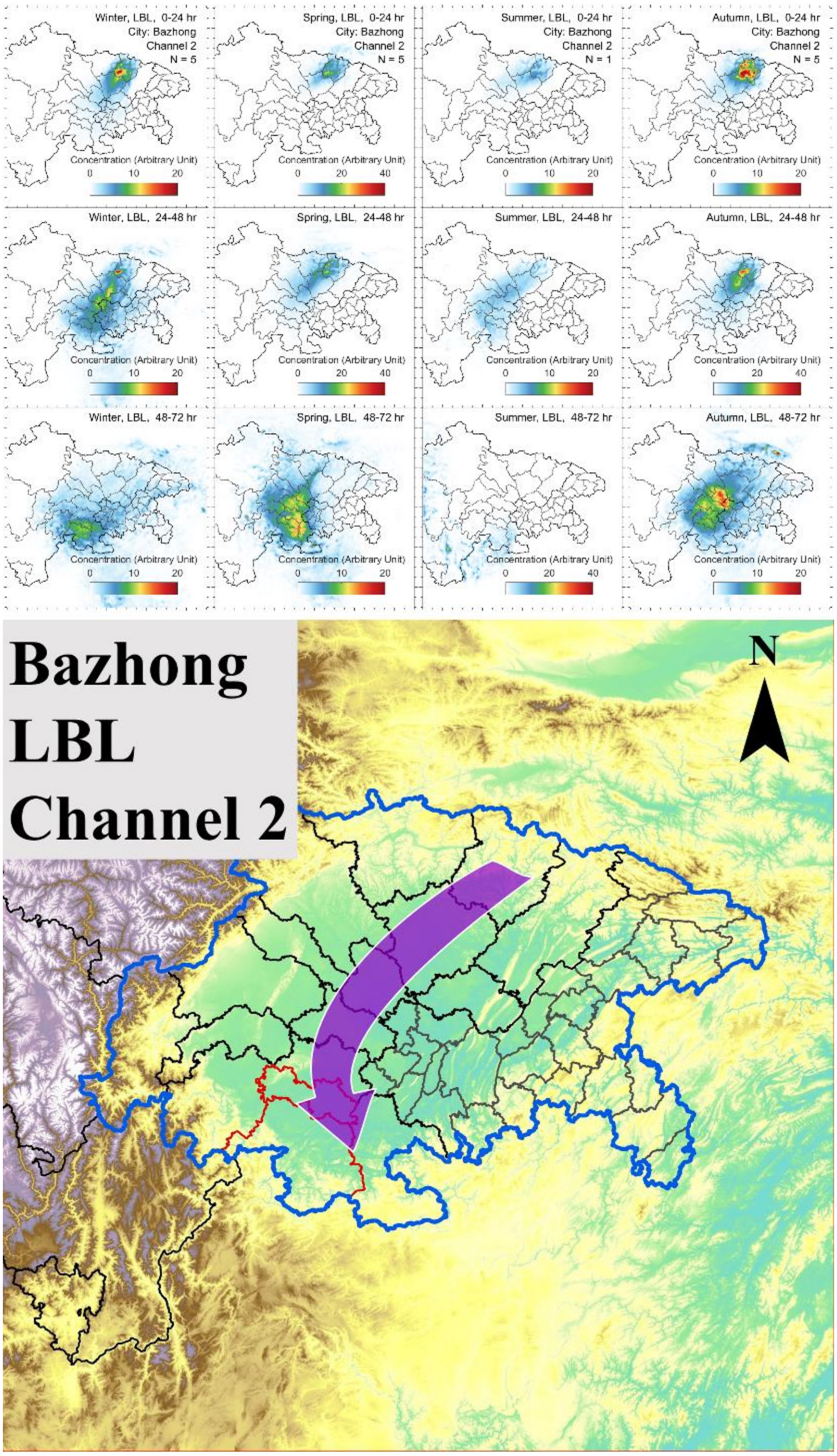

Figure S9 Channel 2 of Bazhong at LBL.

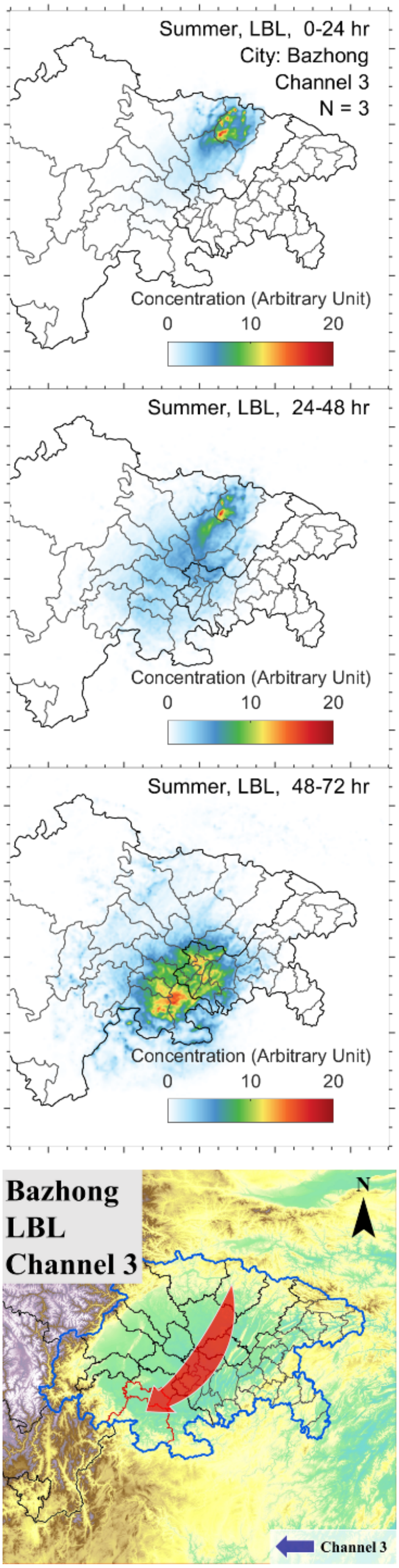

Summer, LBL, 0-24 hr
City: Bazhong
Channel 3
N = 3
Concentration (Arbitrary Unit)
0
10
20
Summer, LBL, 24-48 hr
Concentration (Arbitrary Unit)
0
10
20
Summer, LBL, 48-72 hr
Concentration (Arbitrary Unit)
0
10
20
Bazhong
LBL
Channel 3
N
Channel 3

Figure S10 Channel 3 of Bazhong at LBL.

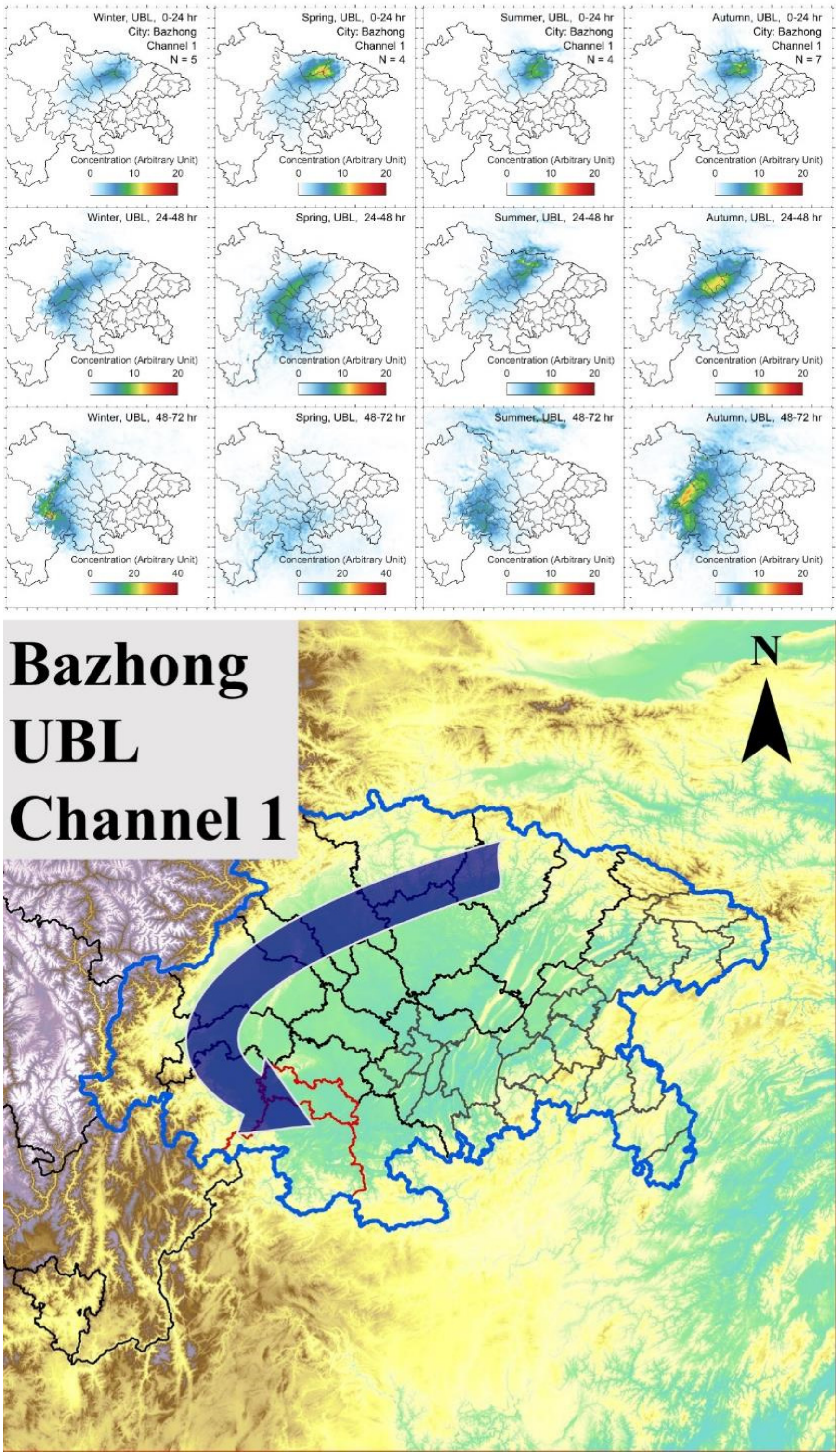

Figure S11 Channel 1 of Bazhong at UBL.

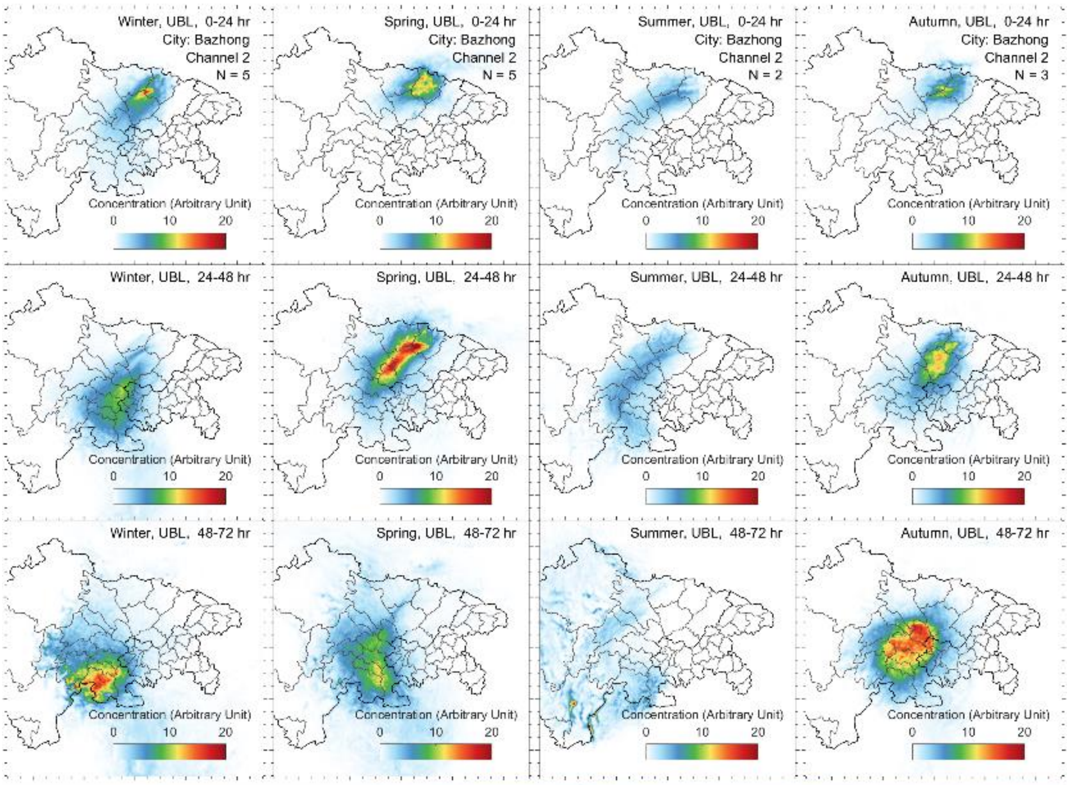
Winter, UBL, 0-24 hr
City: Bazhong
Channel 2
N = 5
Spring, UBL, 0-24 hr
City: Bazhong
Channel 2
N = 5
Summer, UBL, 0-24 hr
City: Bazhong
Channel 2
N = 2
Autumn, UBL, 0-24 hr
City: Bazhong
Channel 2
N = 3
Winter, UBL, 24-48 hr
Spring, UBL, 24-48 hr
Summer, UBL, 24-48 hr
Autumn, UBL, 24-48 hr
Winter, UBL, 48-72 hr
Spring, UBL, 48-72 hr
Summer, UBL, 48-72 hr
Autumn, UBL, 48-72 hr
Concentration (Arbitrary Unit)
0 10 20

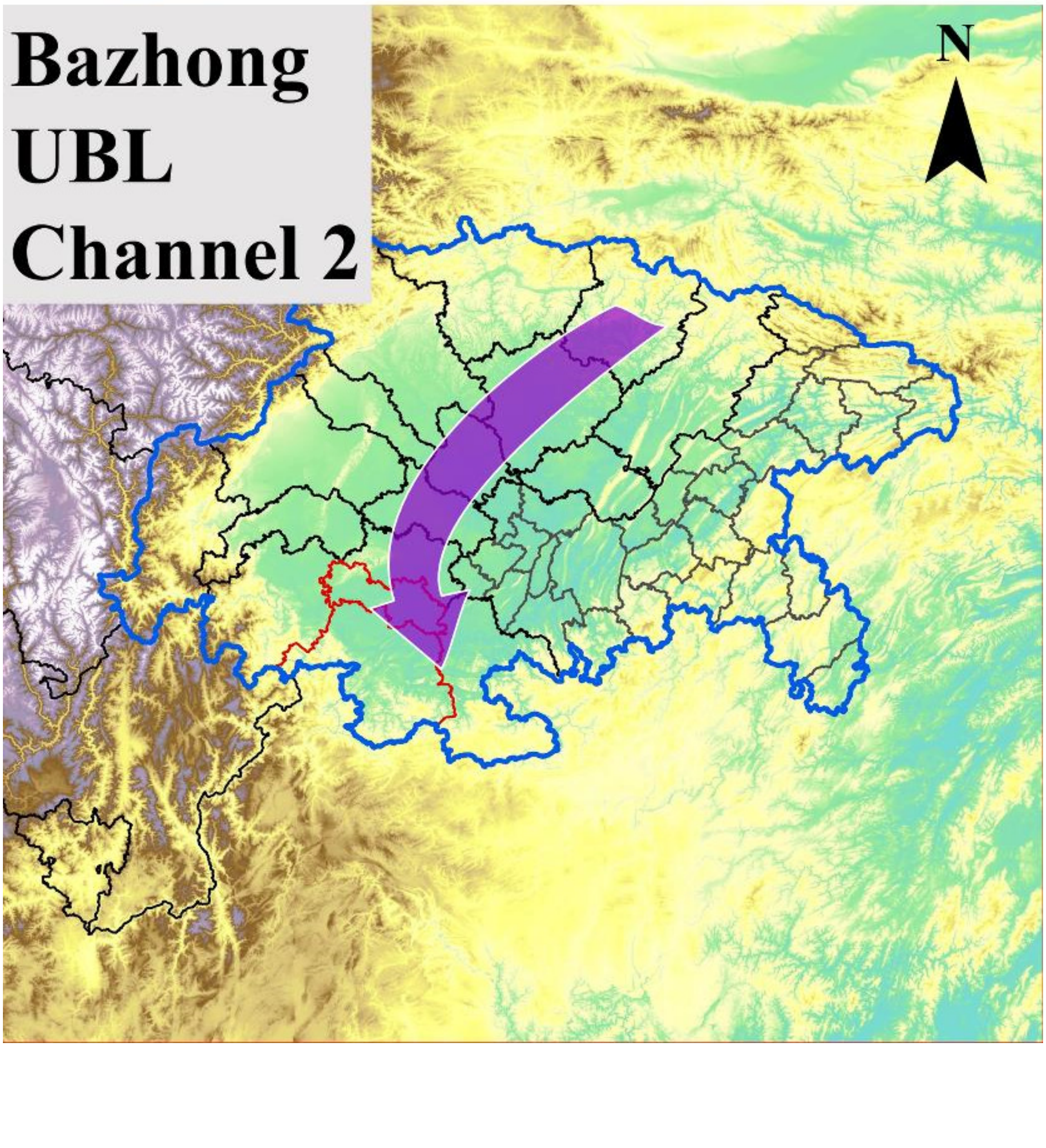
Bazhong
UBL
Channel 2
N

Figure S12 Channel 2 of Bazhong at UBL.

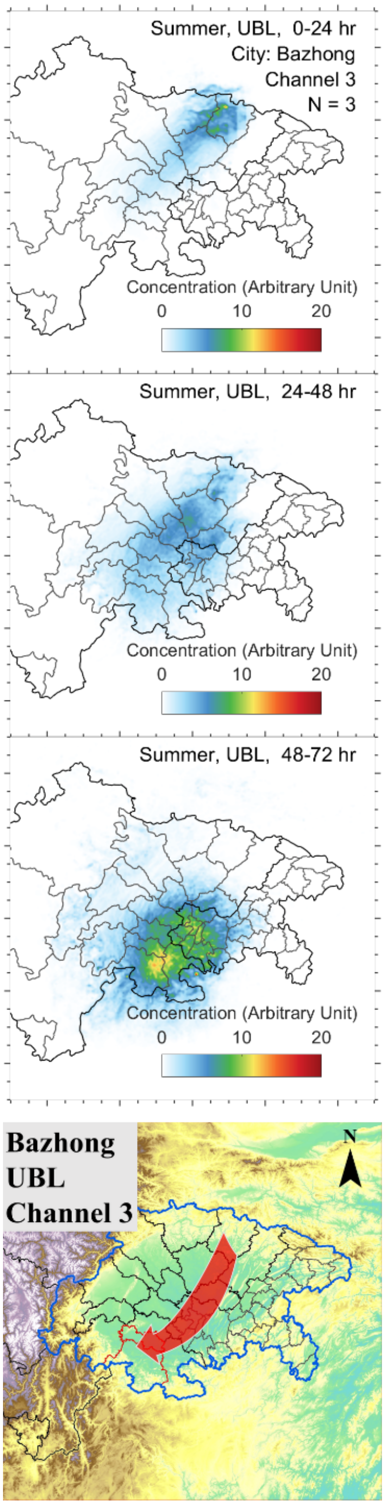

Figure S13 Channel 3 of Bazhong at UBL.

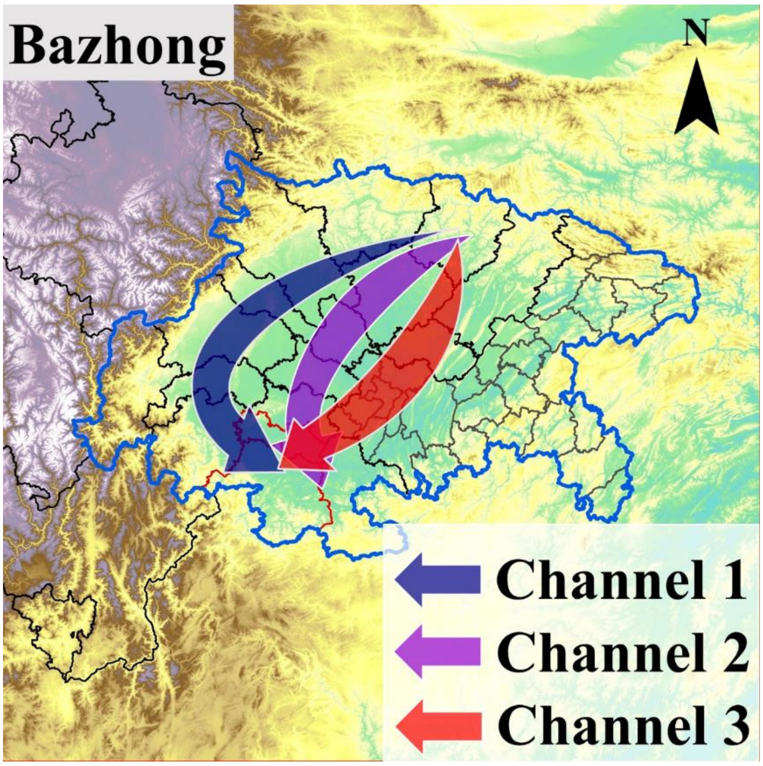

Figure S14 The identified 3 channels originating in Bazhong

Table S2 Occurrence frequencies of each channel originating in Bazhong in four seasons.

City	Channel	Layer	Season			
			autumn	spring	summer	winter
Bazhong	1	LBL	25.8%	13.3%	16.1%	32.3%
		UBL	22.6%	13.3%	12.9%	16.1%
	2	LBL	16.1%	16.7%	3.2%	16.1%
		UBL	9.7%	16.7%	6.5%	16.1%
	3	LBL			9.7%	
		UBL			9.7%	

1.3 Guangyuan

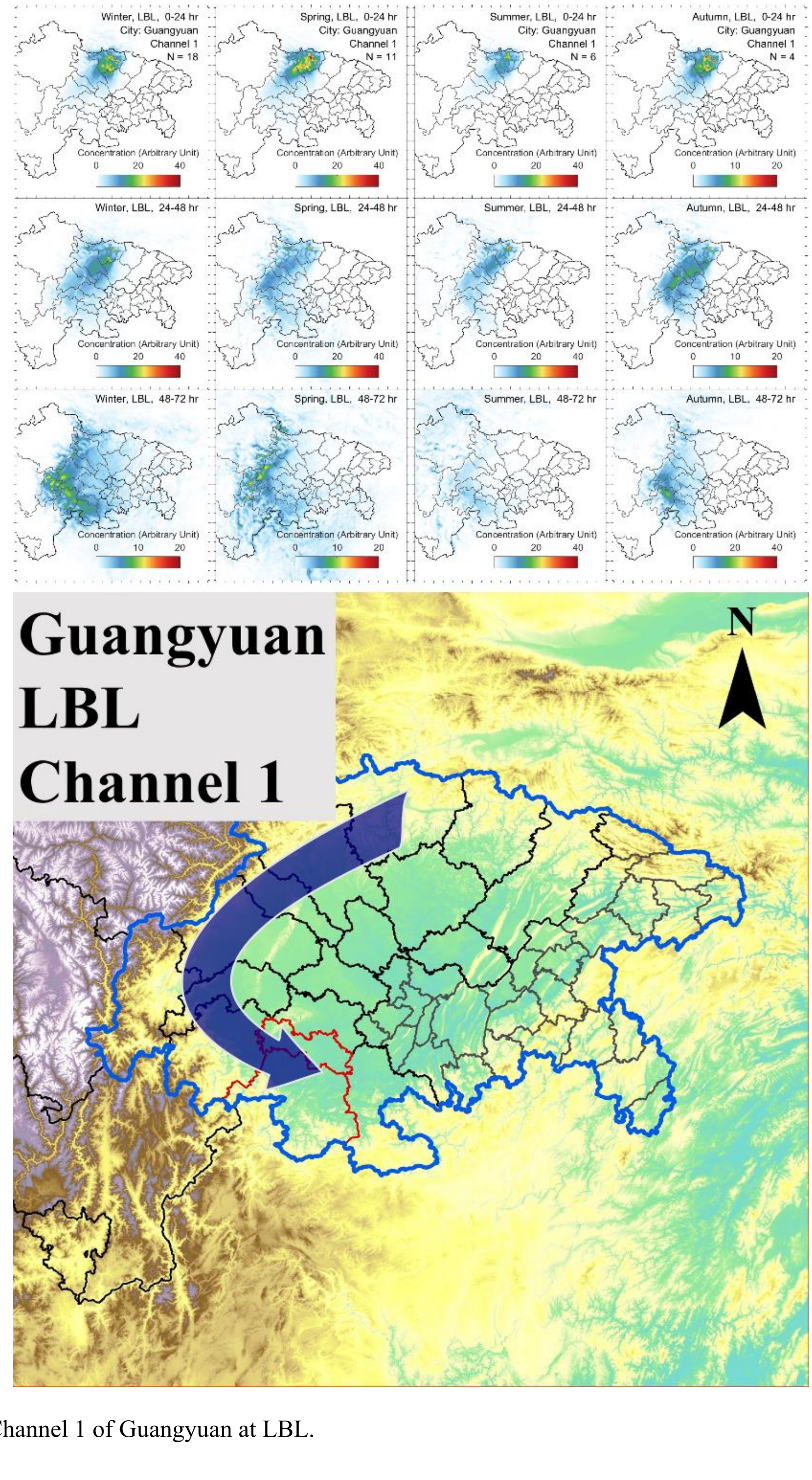

Figure S15 Channel 1 of Guangyuan at LBL.

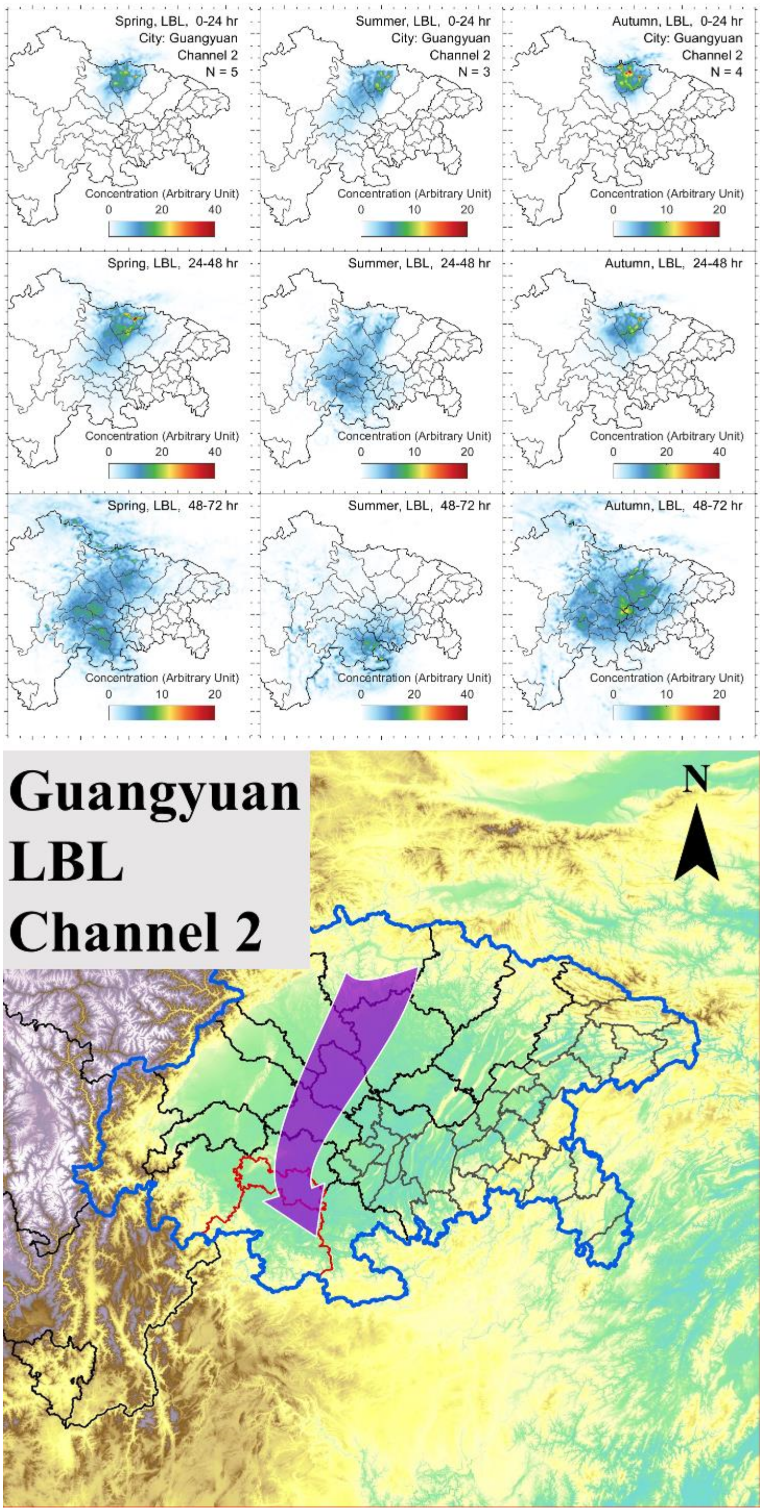

Figure S16 Channel 2 of Guangyuan at LBL.

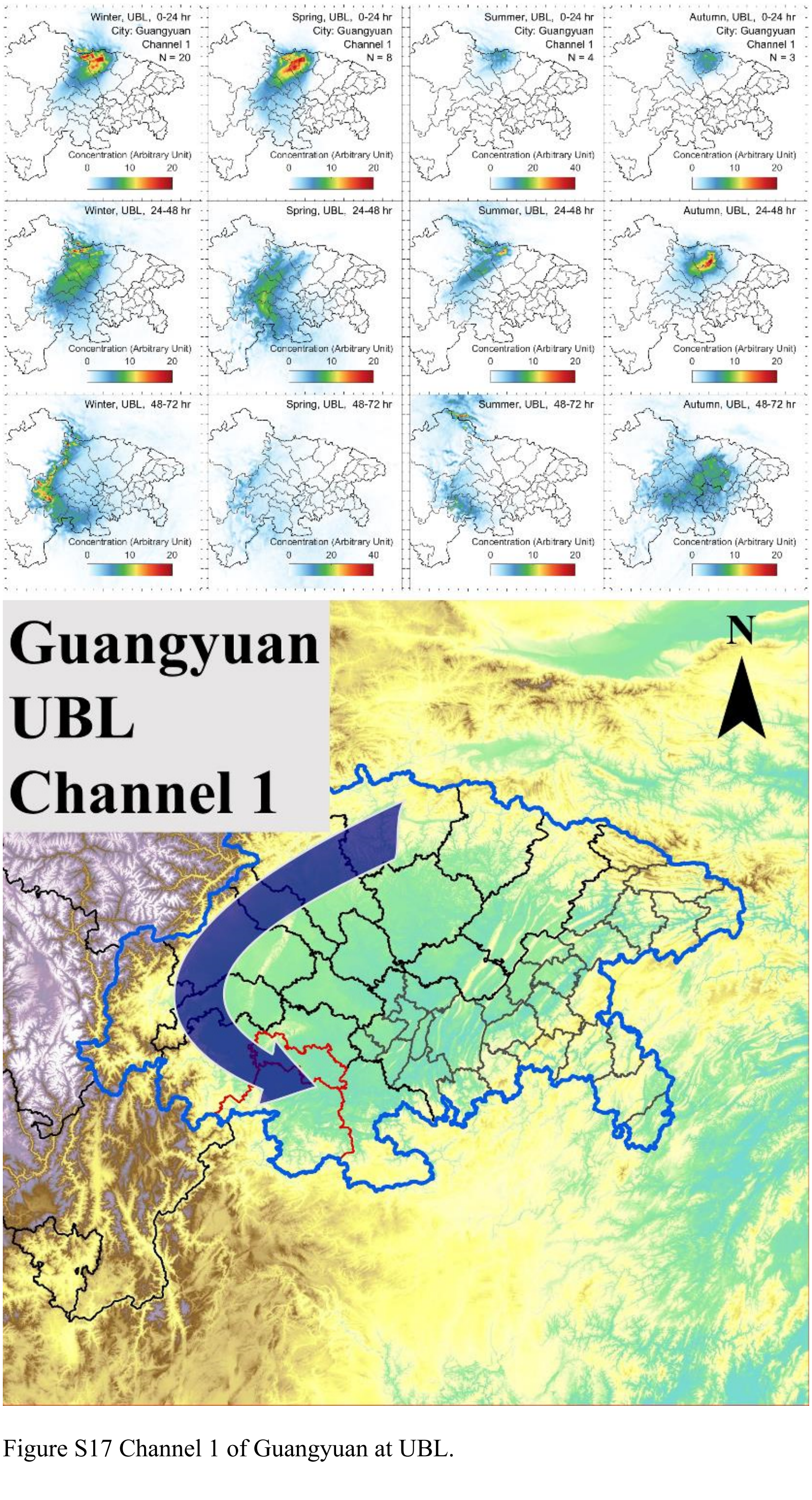

Figure S17 Channel 1 of Guangyuan at UBL.

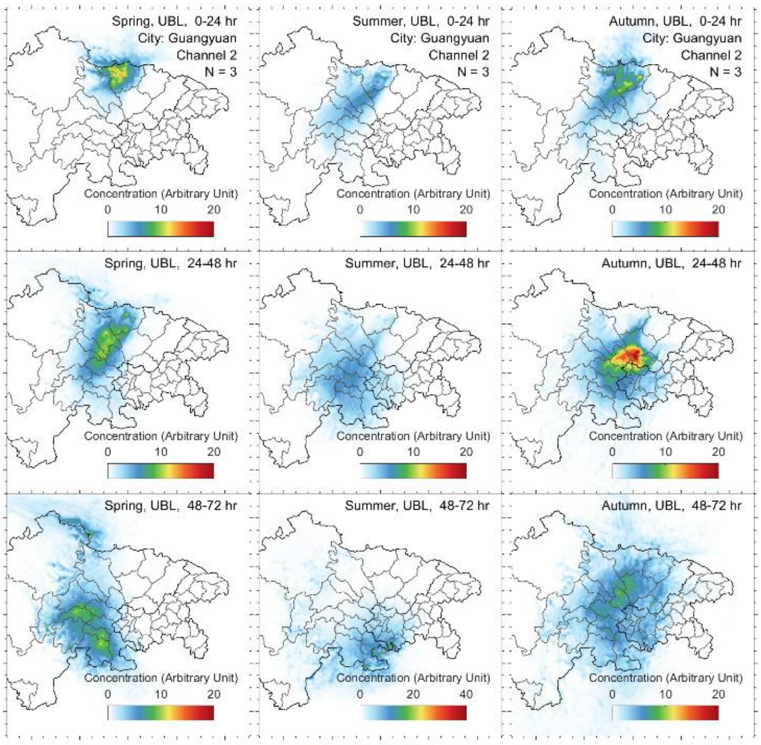

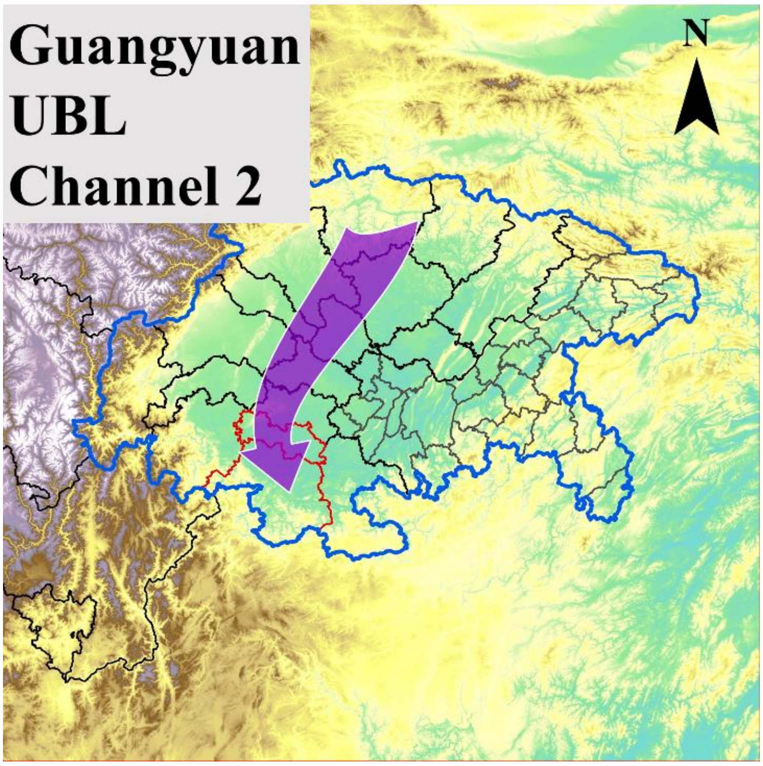

Figure S18 Channel 2 of Guangyuan at UBL.

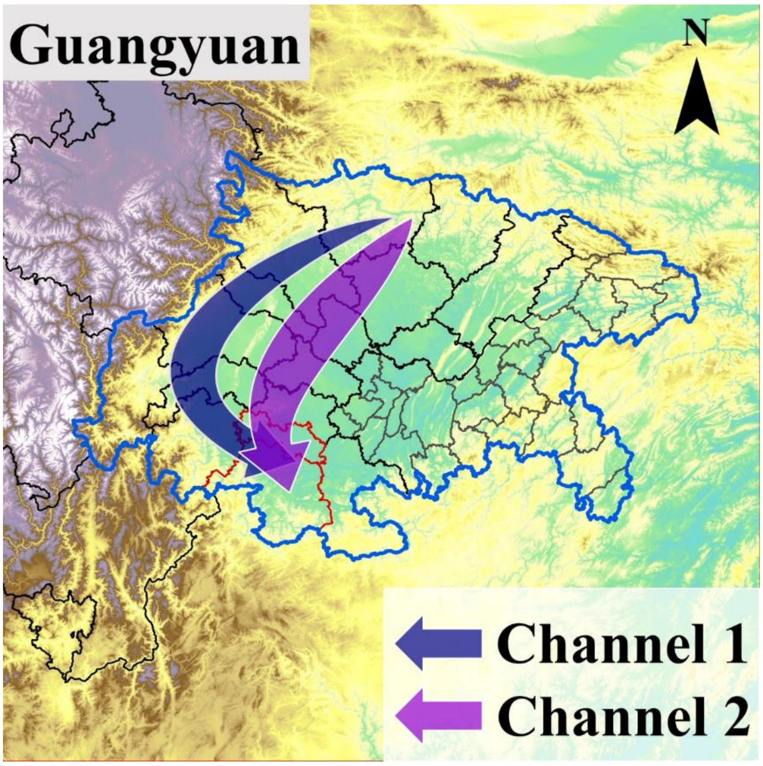

Figure S19 The identified 2 channels originating in Guangyuan.

Table S3 Occurrence frequencies of each channel originating in Guangyuan in four seasons.

City	Channel	Layer	Season			
			autumn	spring	summer	winter
Guangyuan	1	LBL	12.9%	36.7%	19.4%	58.1%
		UBL	9.7%	26.7%	12.9%	64.5%
	2	LBL	12.9%	16.7%	9.7%	
		UBL	9.7%	10.0%	9.7%	

1.4 Nanchong

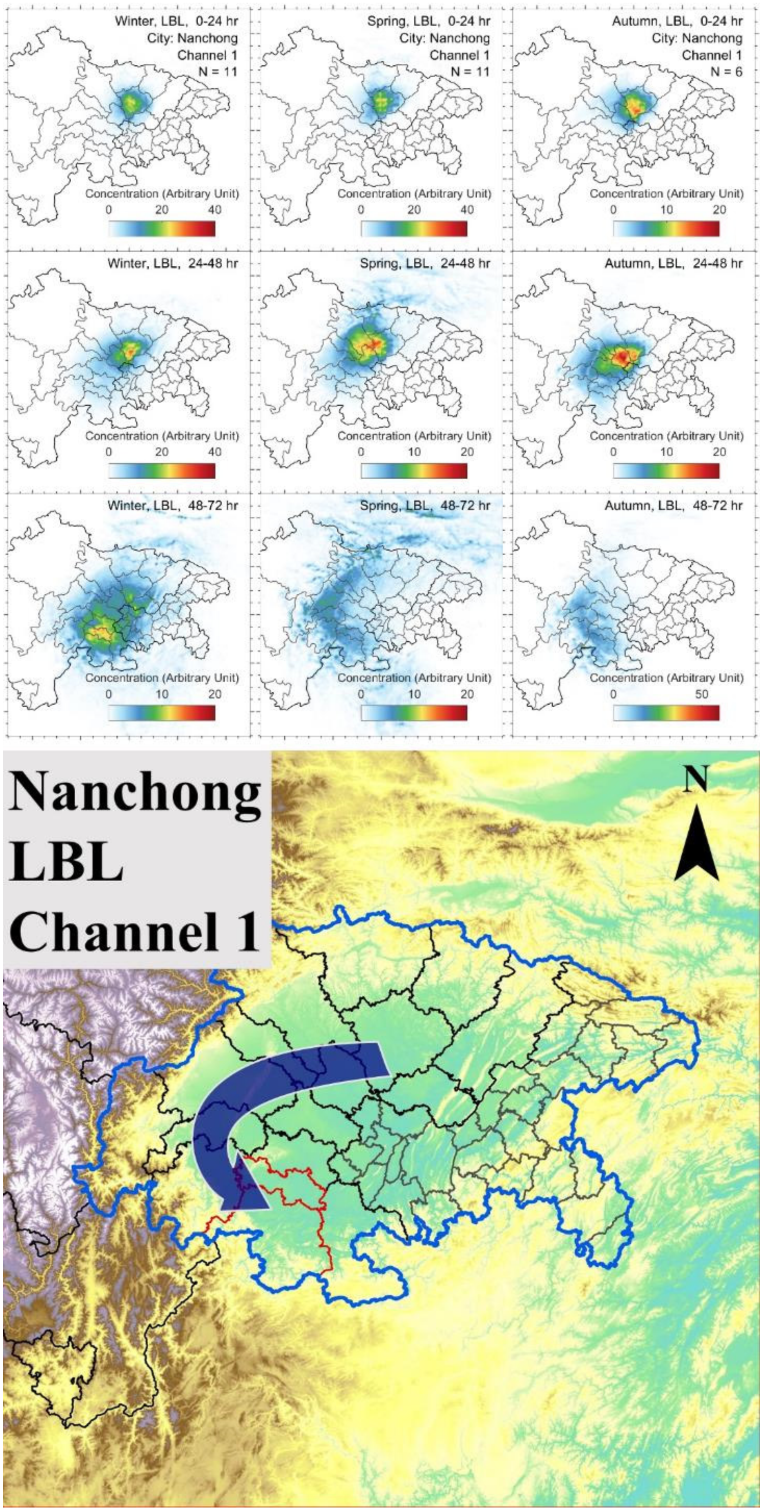

Figure S20 Channel 1 of Nanchong at LBL.

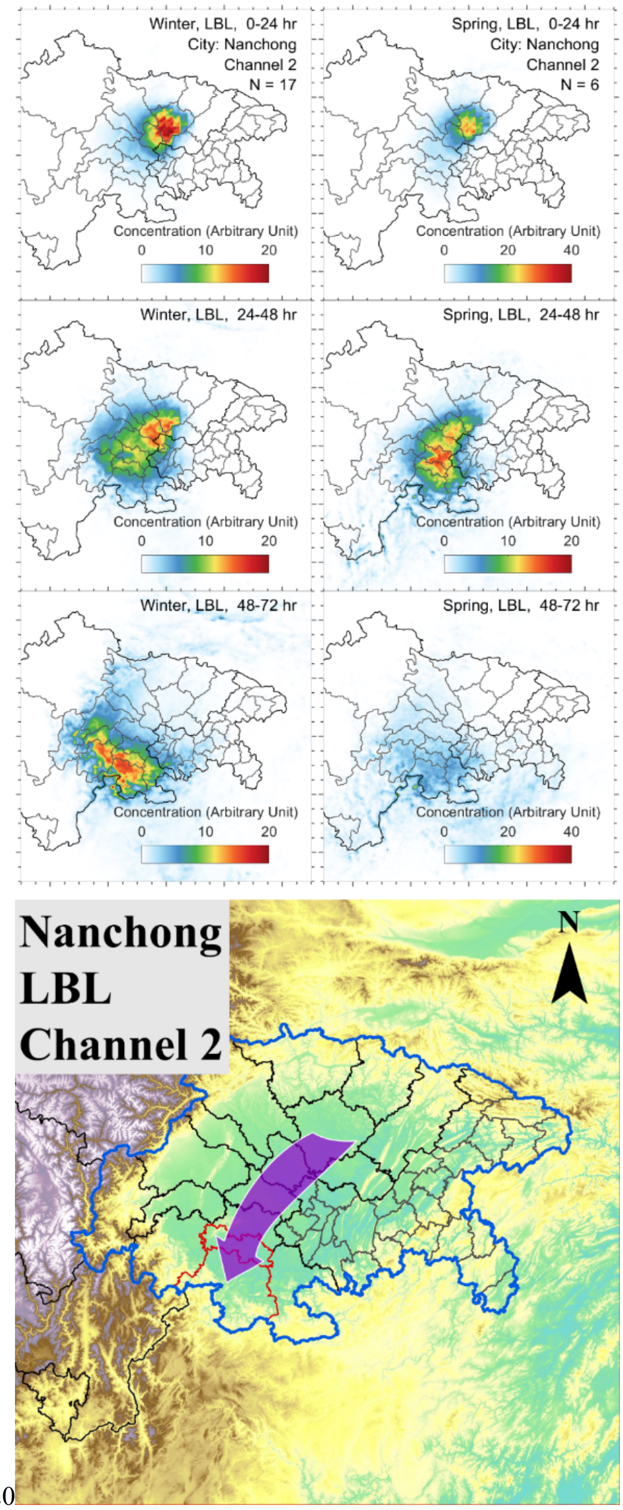

Figure S21 Channel 2 of Nanchong at LBL.

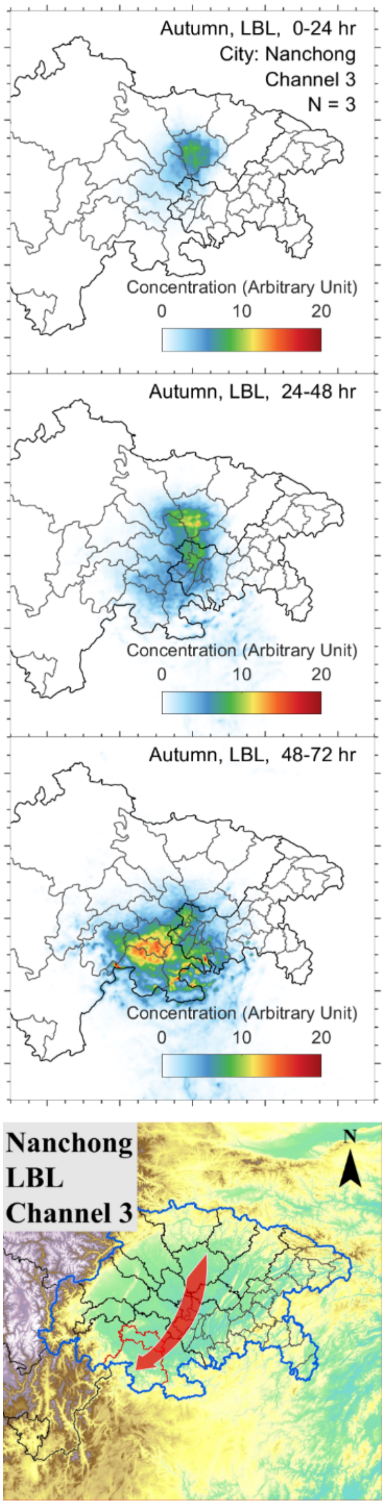

Figure S22 Channel 3 of Nanchong at LBL.

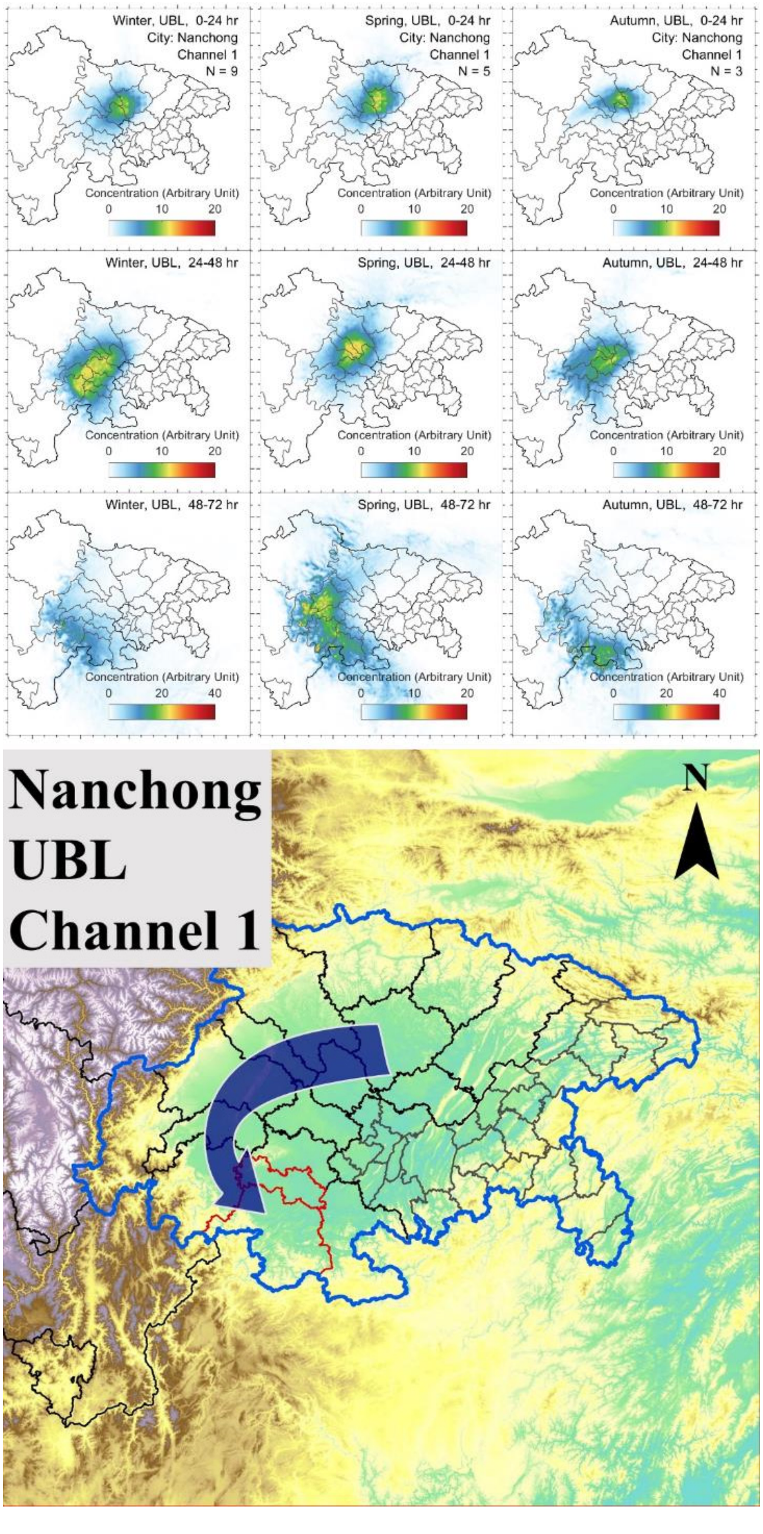

Figure S23 Channel 1 of Nanchong at UBL.

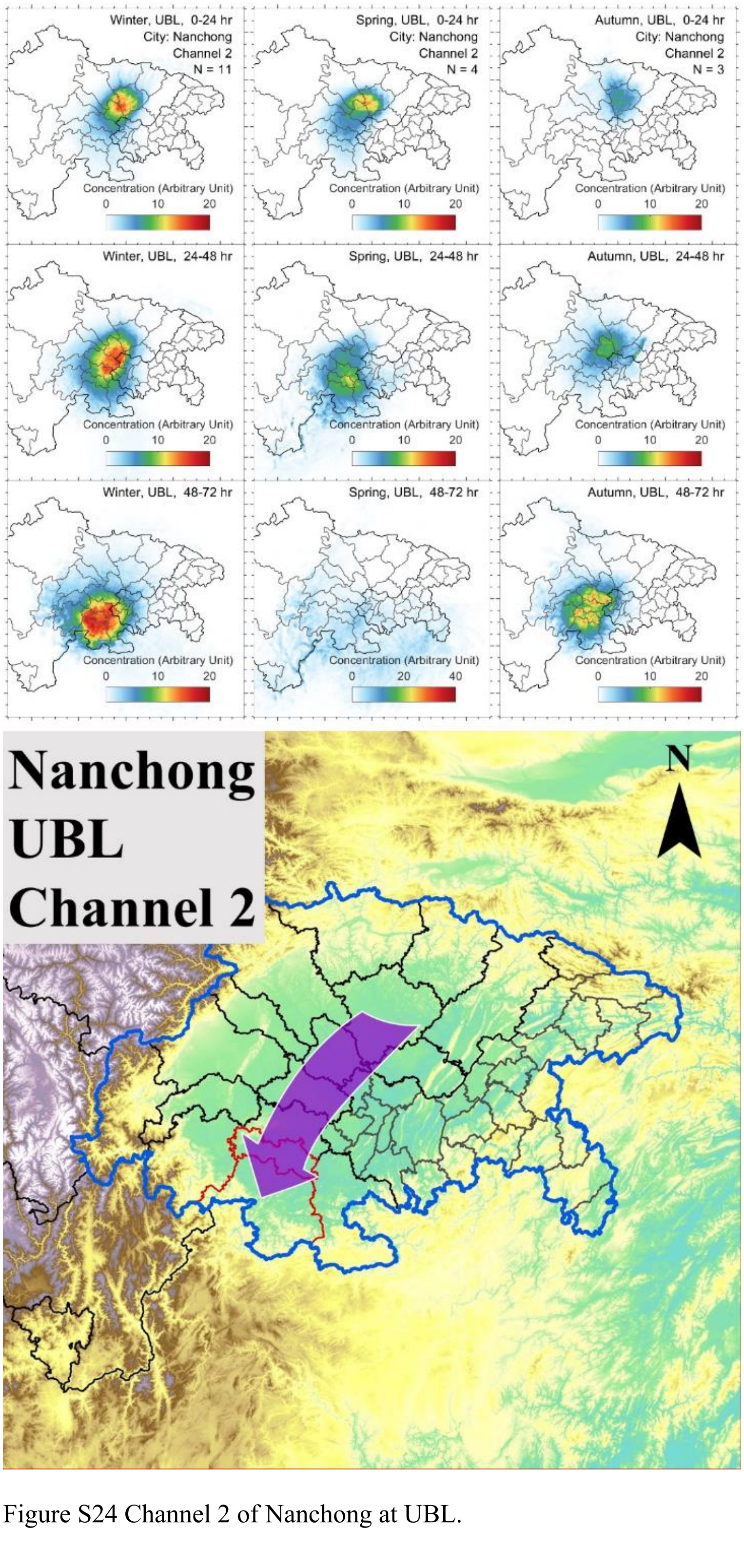

Figure S24 Channel 2 of Nanchong at UBL.

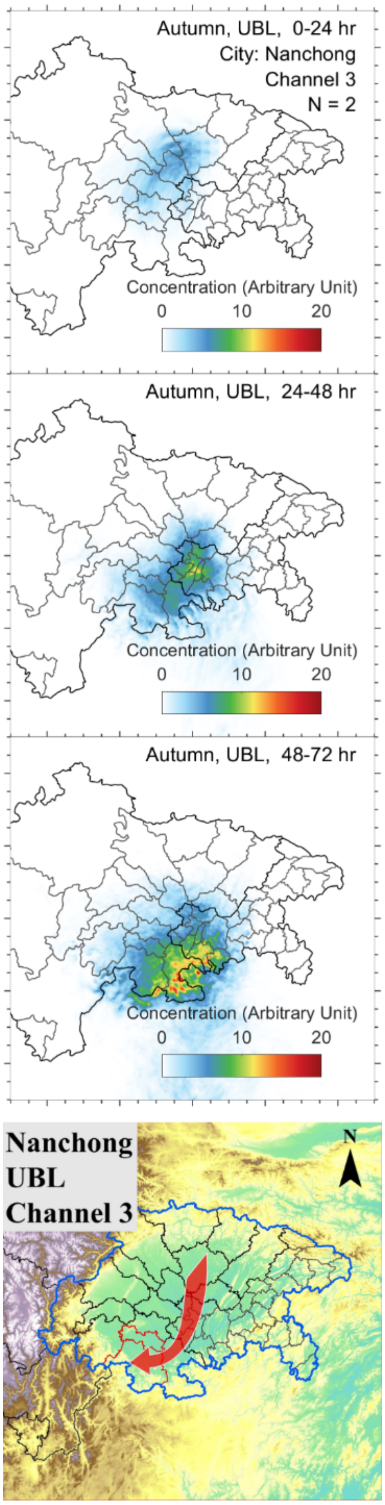

Figure S25 Channel 3 of Nanchong at UBL.

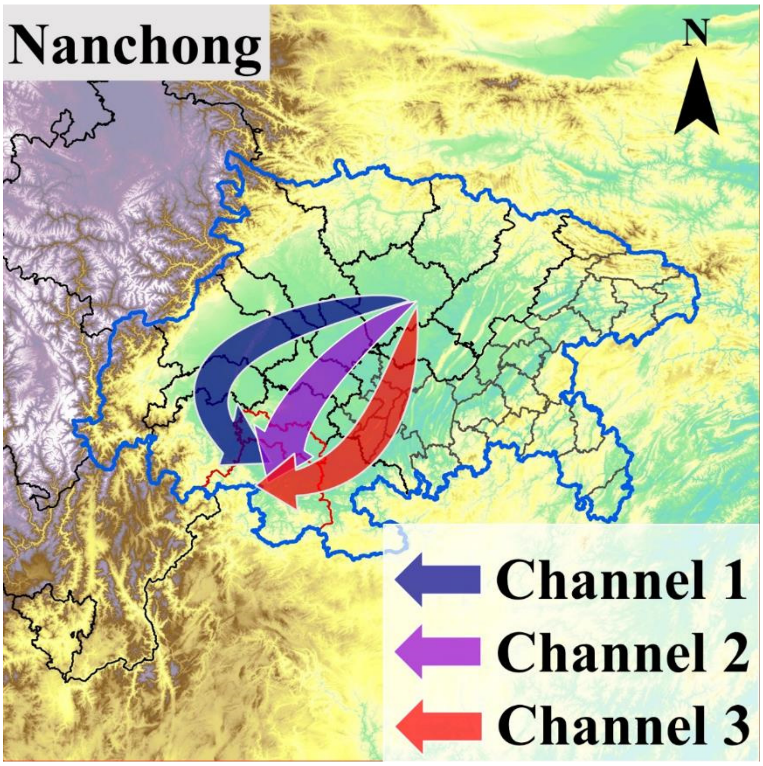

Figure S26 The identified 3 channels originating in Nanchong.

Table S4 Occurrence frequencies of each channel originating in Nanchong in four seasons.

City	Channel	Layer	Season			
			autumn	spring	summer	winter
Nanchong	1	LBL	19.4%	36.7%		35.5%
		UBL	9.7%	16.7%		29.0%
	2	LBL		20.0%		54.8%
		UBL	9.7%	13.3%		35.5%
	3	LBL	9.7%			
		UBL	6.5%			

1.5 Guang'an

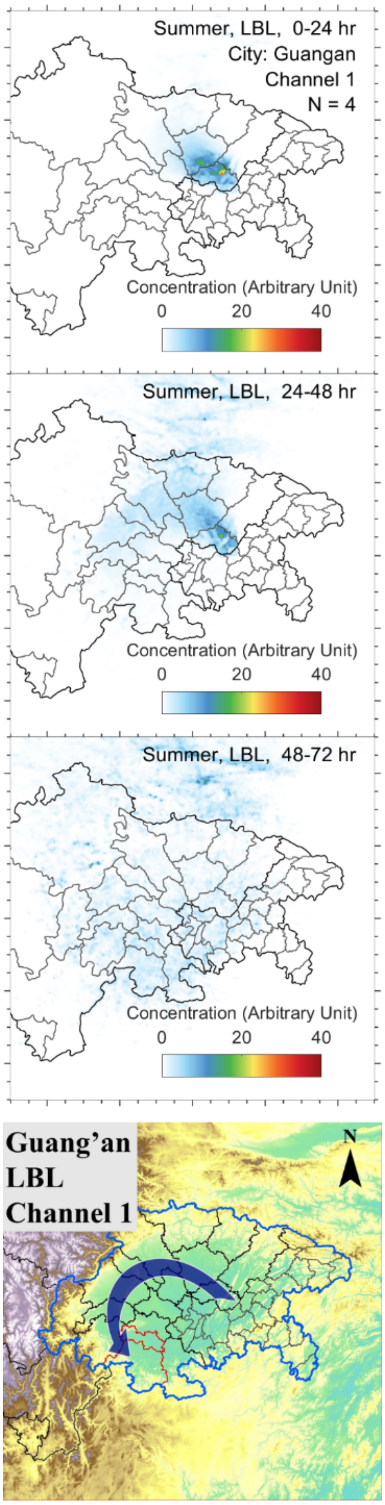

Figure S27 Channel 1 of Guang'an at LBL.

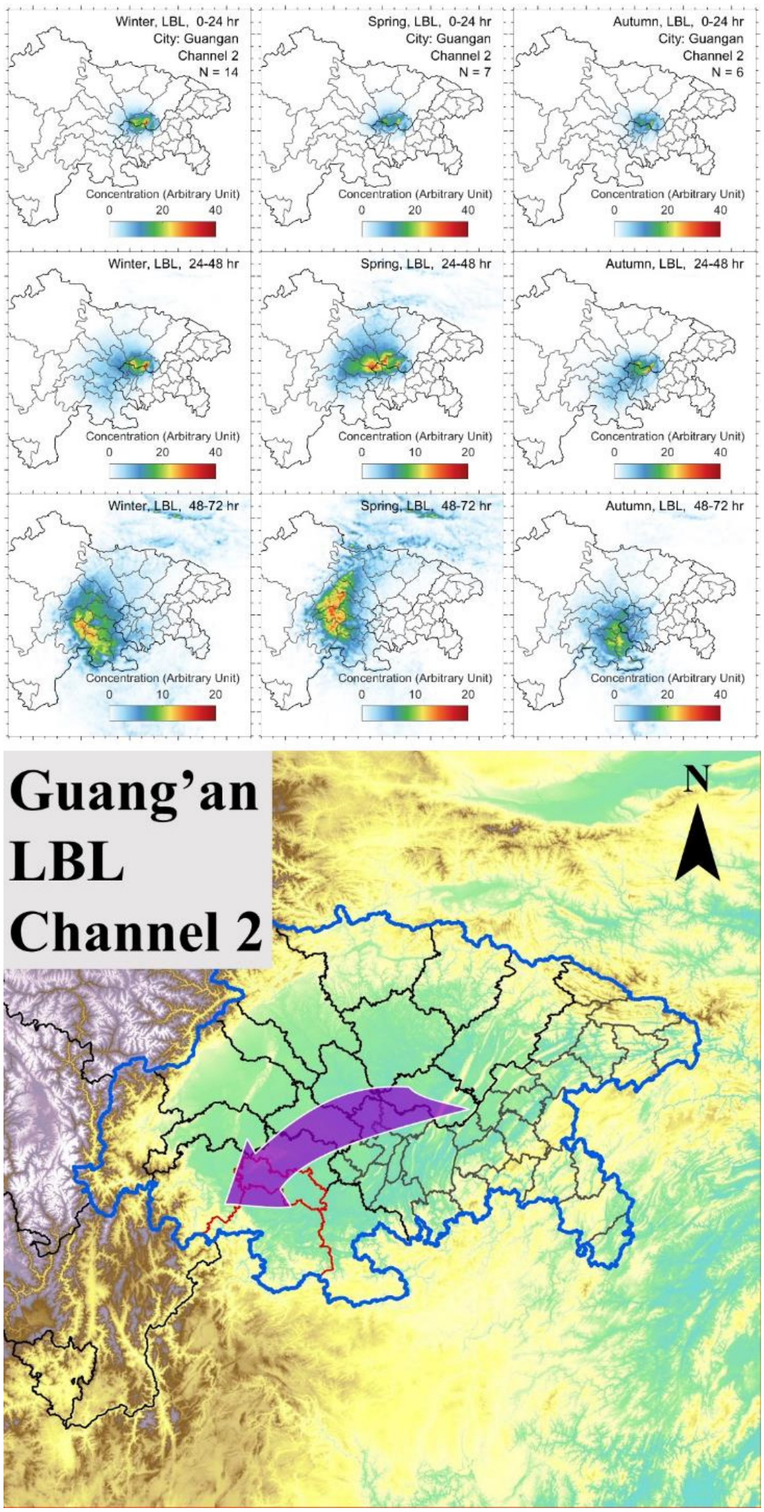

Figure S28 Channel 2 of Guang’an at LBL.

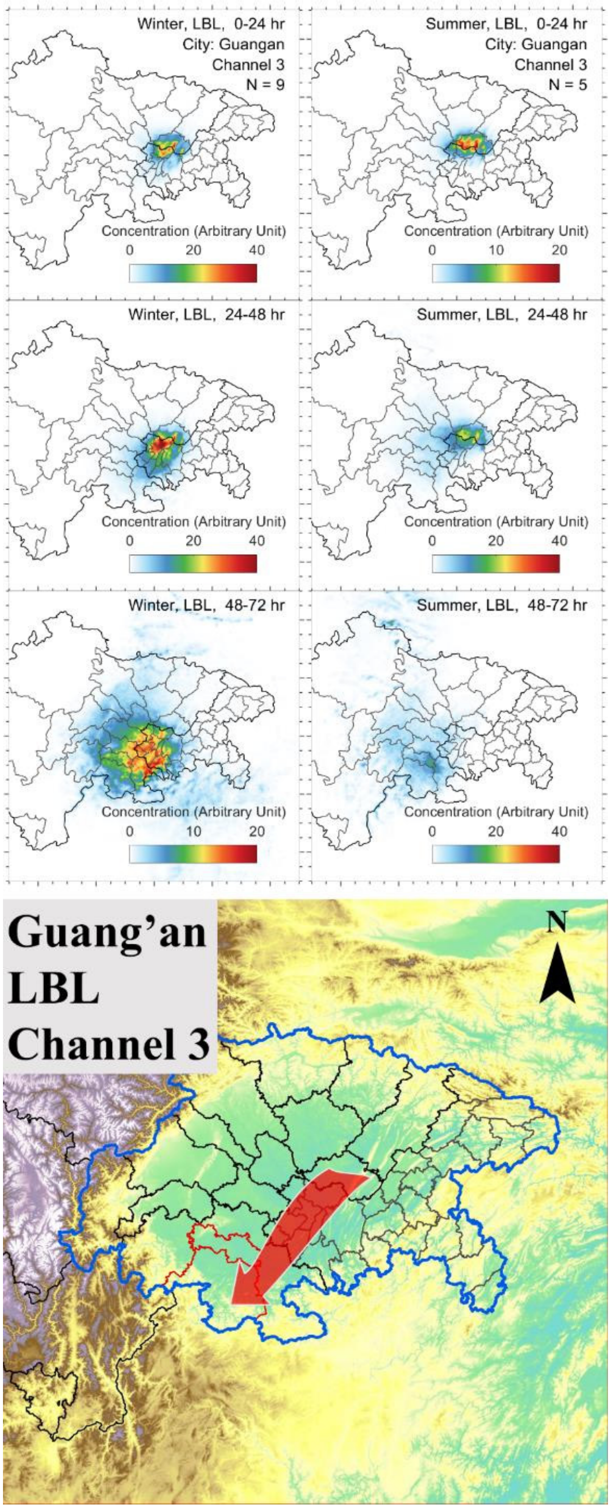

Figure S29 Channel 3 of Guang'an at LBL.

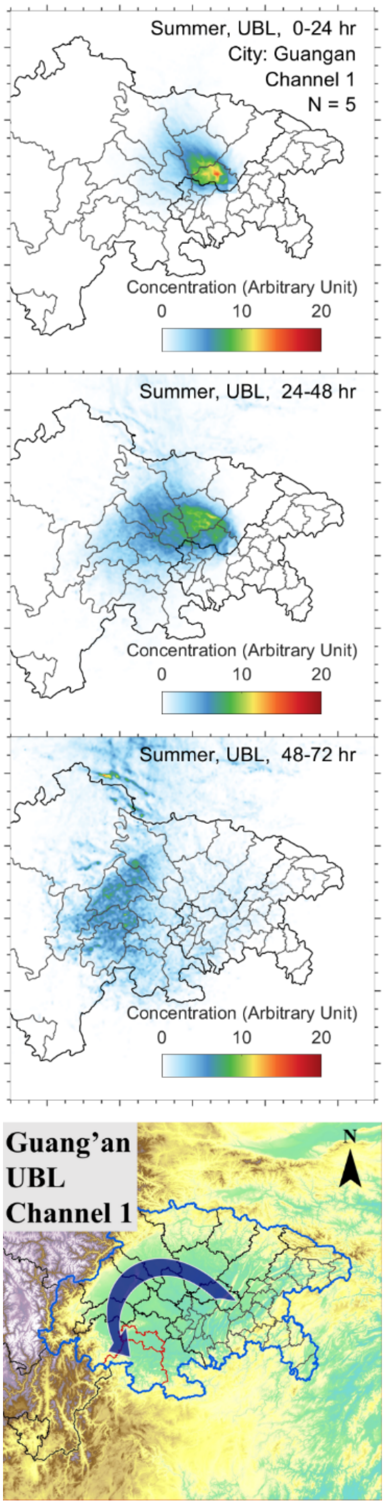

Figure S30 Channel 1 of Guang'an at UBL.

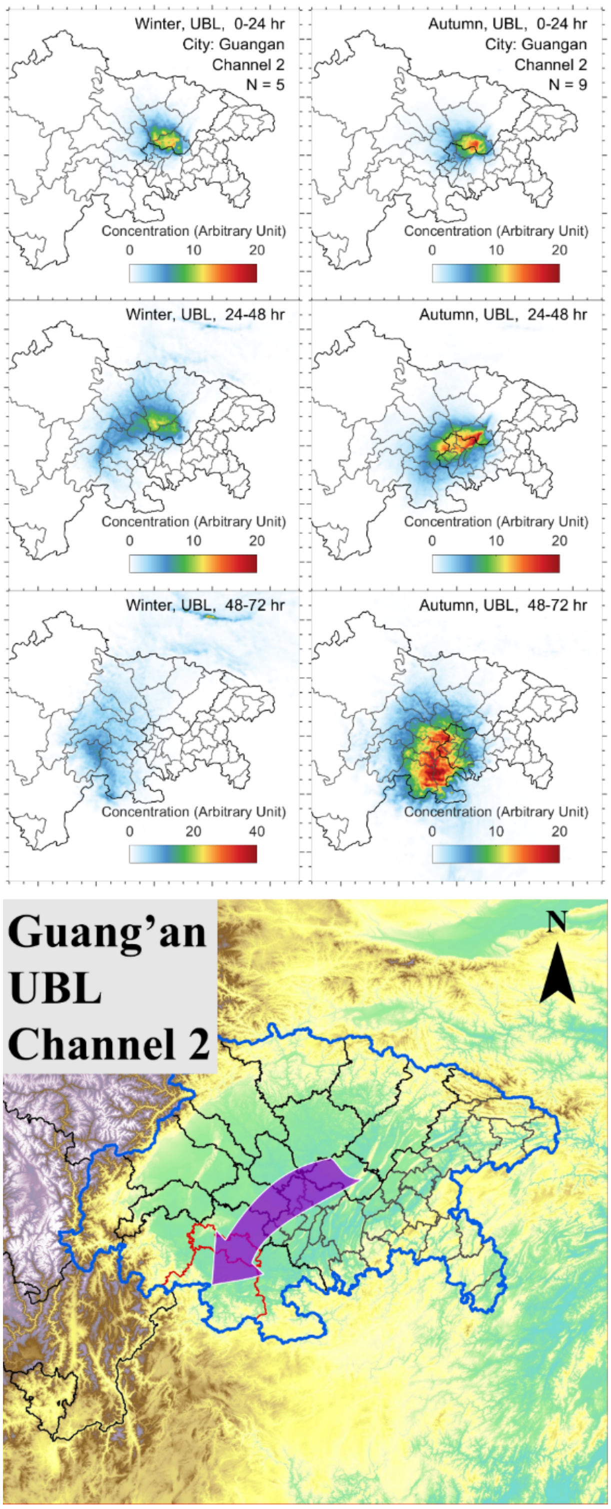

Figure S31 Channel 2 of Guang'an at UBL.

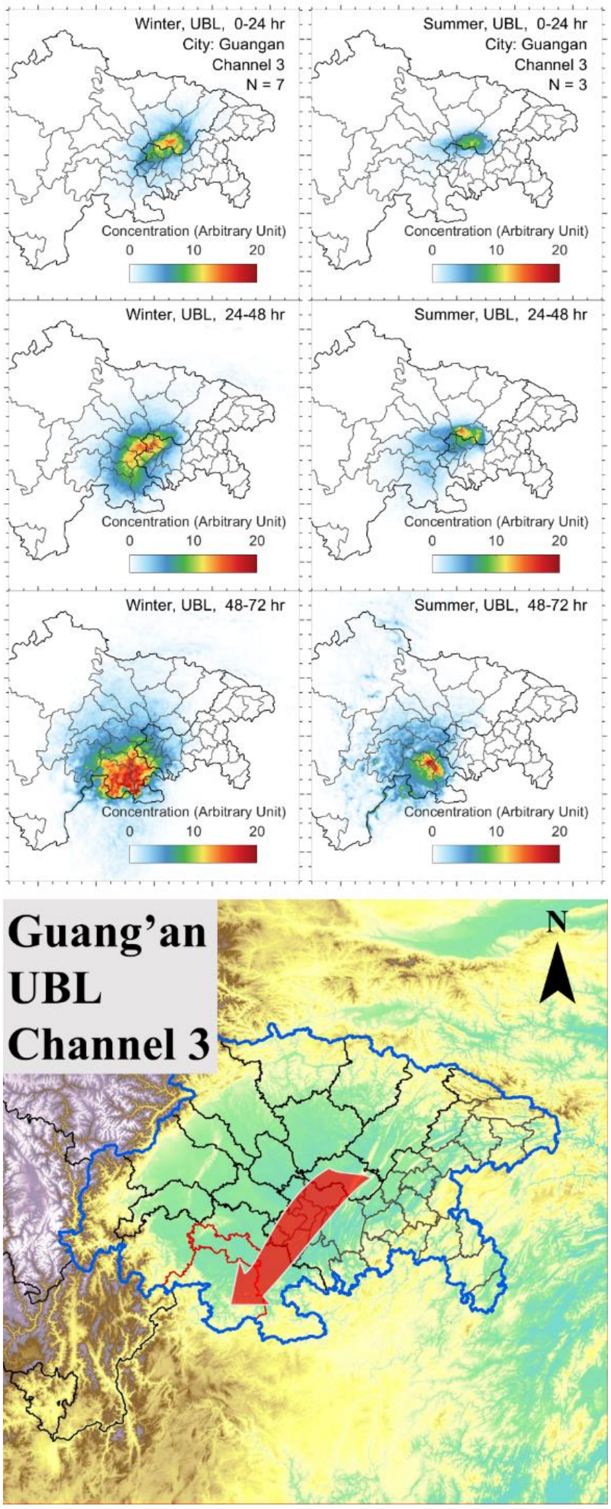

Figure S32 Channel 3 of Guang'an at UBL.

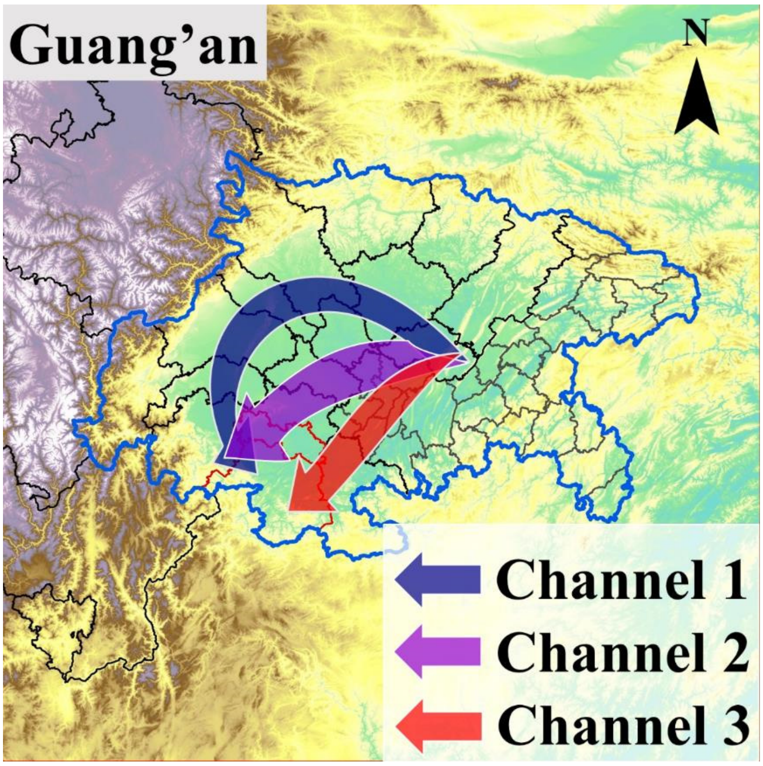

Figure S33 The identified 3 channels originating in Guang’an.

Table S5 Occurrence frequencies of each channel originating in Guang'an in four seasons.

City	Channel	Layer	Season			
			autumn	spring	summer	winter
Guang'an	1	LBL			12.9%	
		UBL			16.1%	
	2	LBL	19.4%	23.3%		45.2%
		UBL	29.0%			16.1%
	3	LBL			16.1%	29.0%
		UBL			9.7%	22.6%

2. Chengdu Plain Urban Agglomeration

2.1 Mianyang

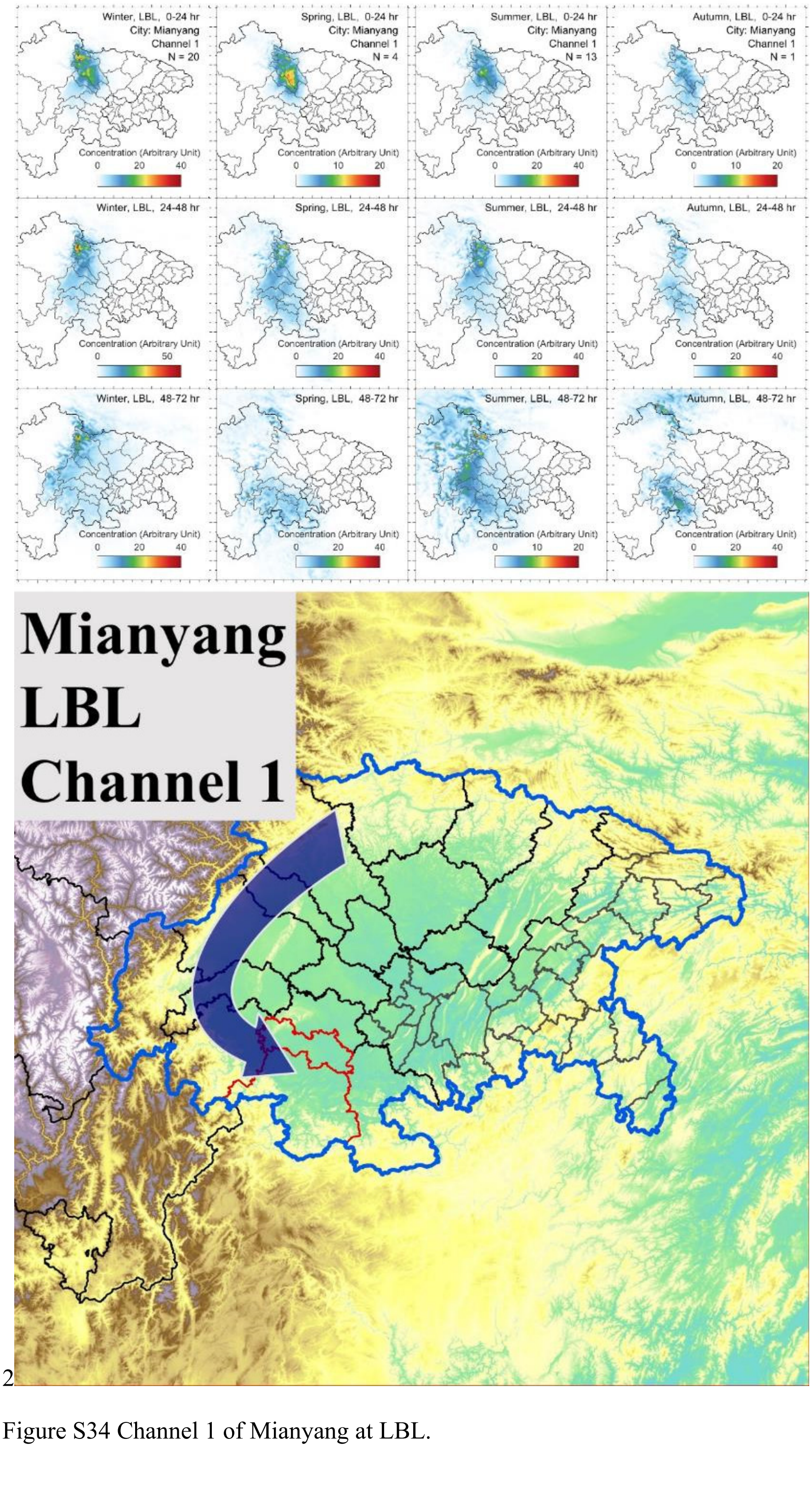

Figure S34 Channel 1 of Mianyang at LBL.

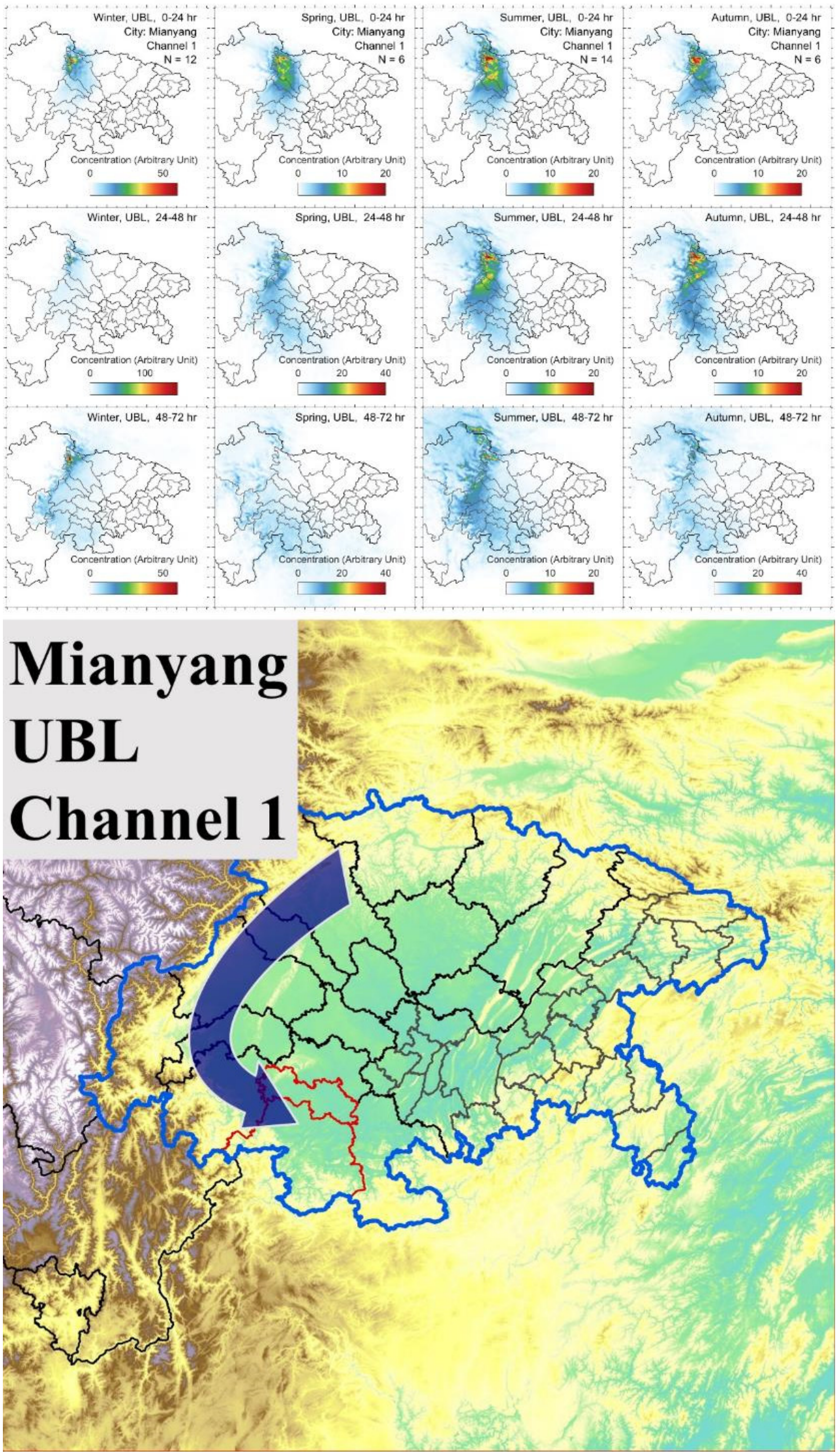

Figure S35 Channel 1 of Mianyang at UBL.

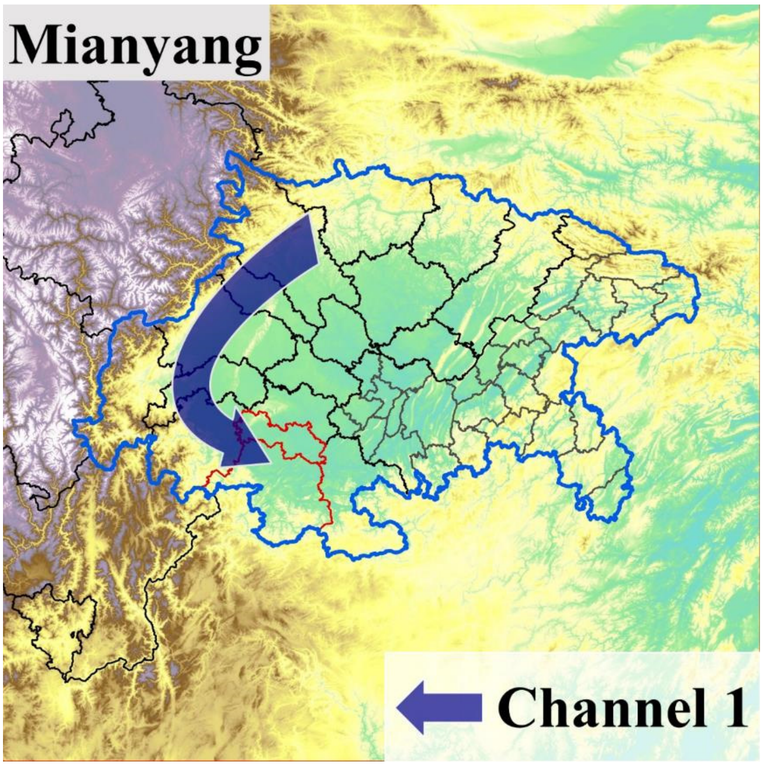

Figure S36 The identified channel originating in Mianyang.

Table S6 Occurrence frequency of the channel originating in Mianyang in four seasons.

City	Channel	Layer	Season			
			autumn	spring	summer	winter
Mianyang	1	LBL	3.2%	13.3%	41.9%	64.5%
		UBL	19.4%	20.0%	45.2%	38.7%

2.2 Deyang

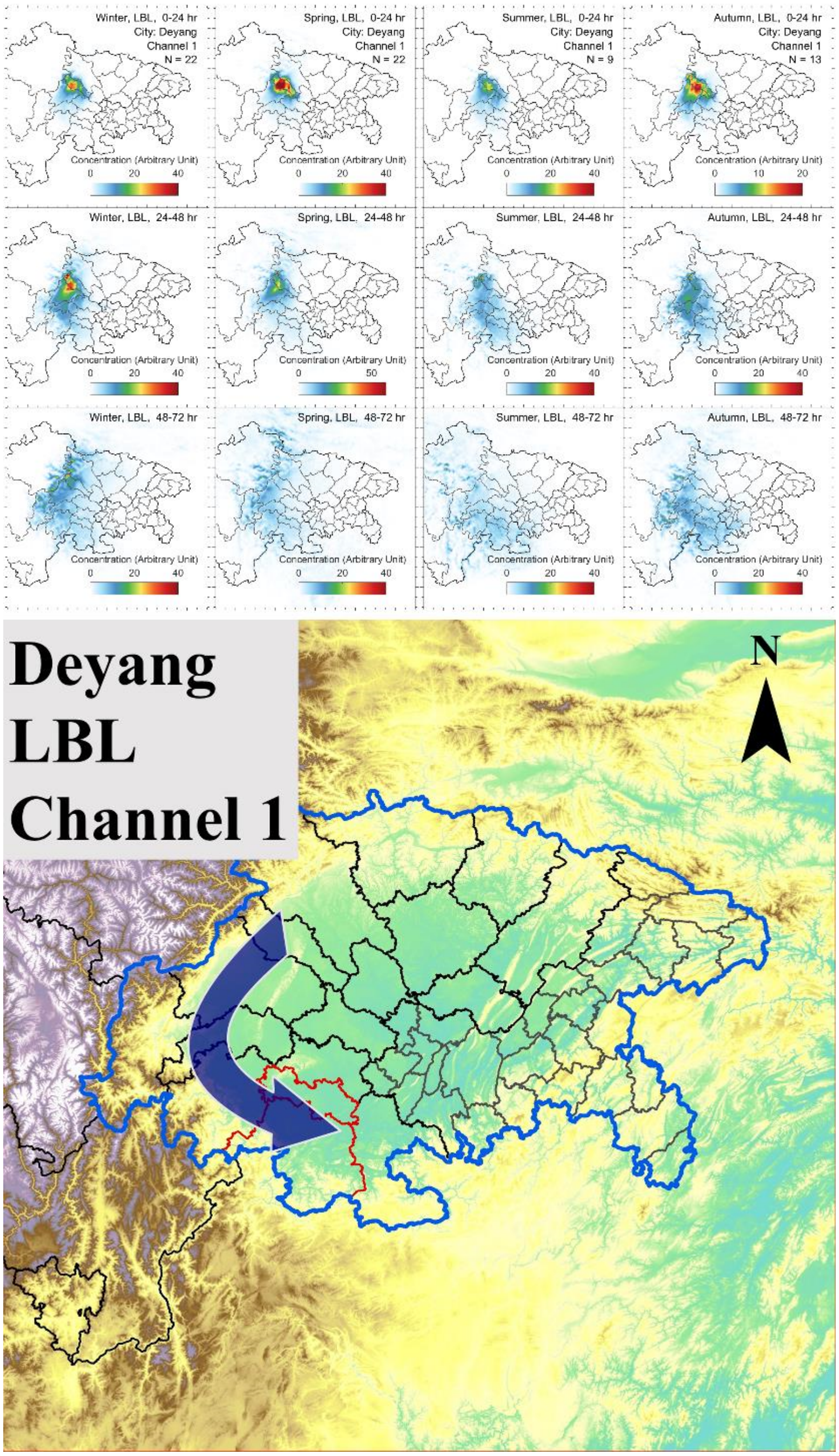

Figure S37 Channel 1 of Deyang at LBL.

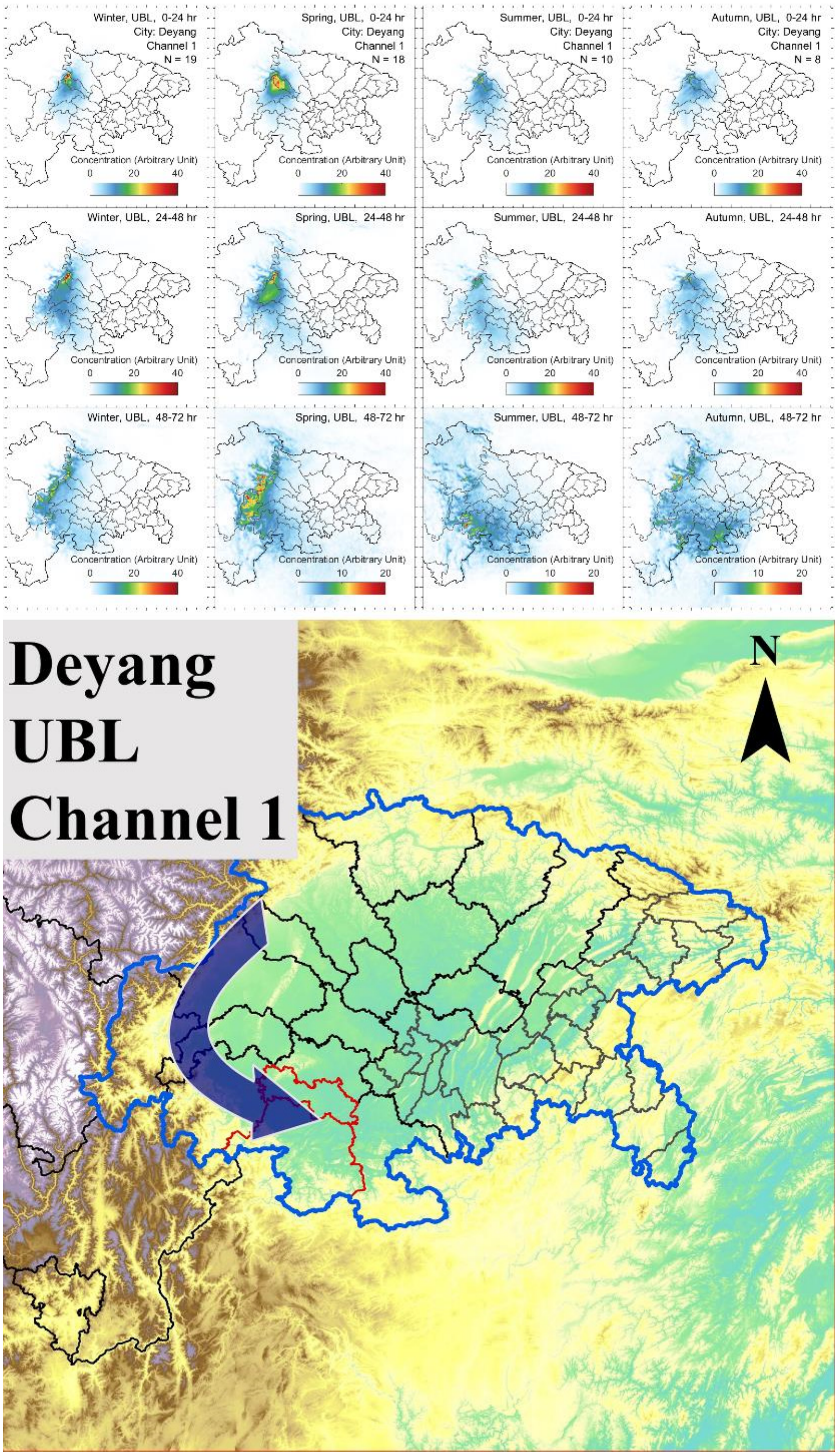

Figure S38 Channel 1 of Deyang at UBL.

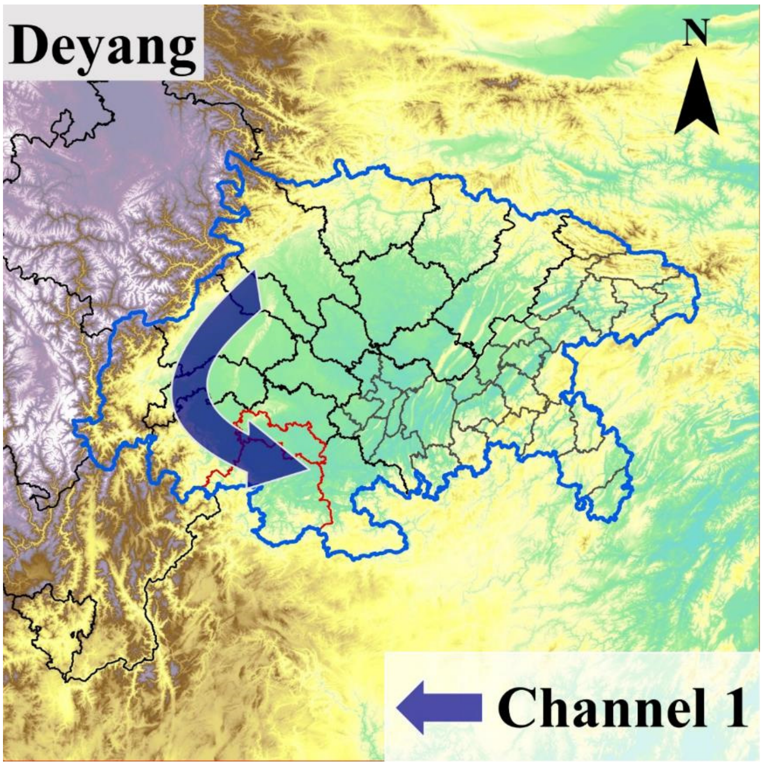

Figure S39 The identified channel originating in Deyang.

Table S7 Occurrence frequency of the channel originating in Deyang in four seasons.

City	Channel	Layer	Season			
			autumn	spring	summer	winter
Deyang	1	LBL	41.9%	73.3%	29.0%	71.0%
		UBL	25.8%	60.0%	32.3%	61.3%

2.3 Suining

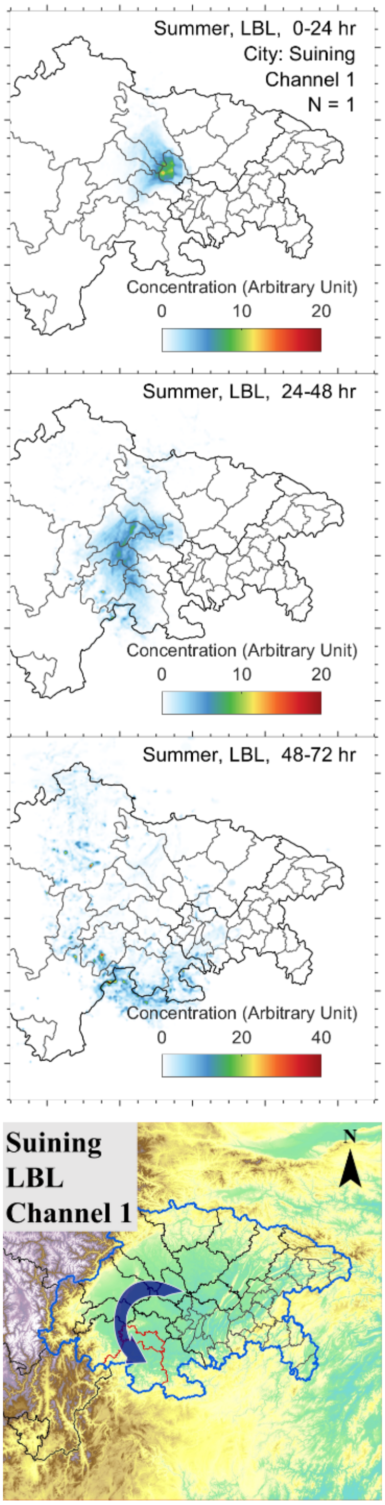

Figure S40 Channel 1 of Suining at LBL.

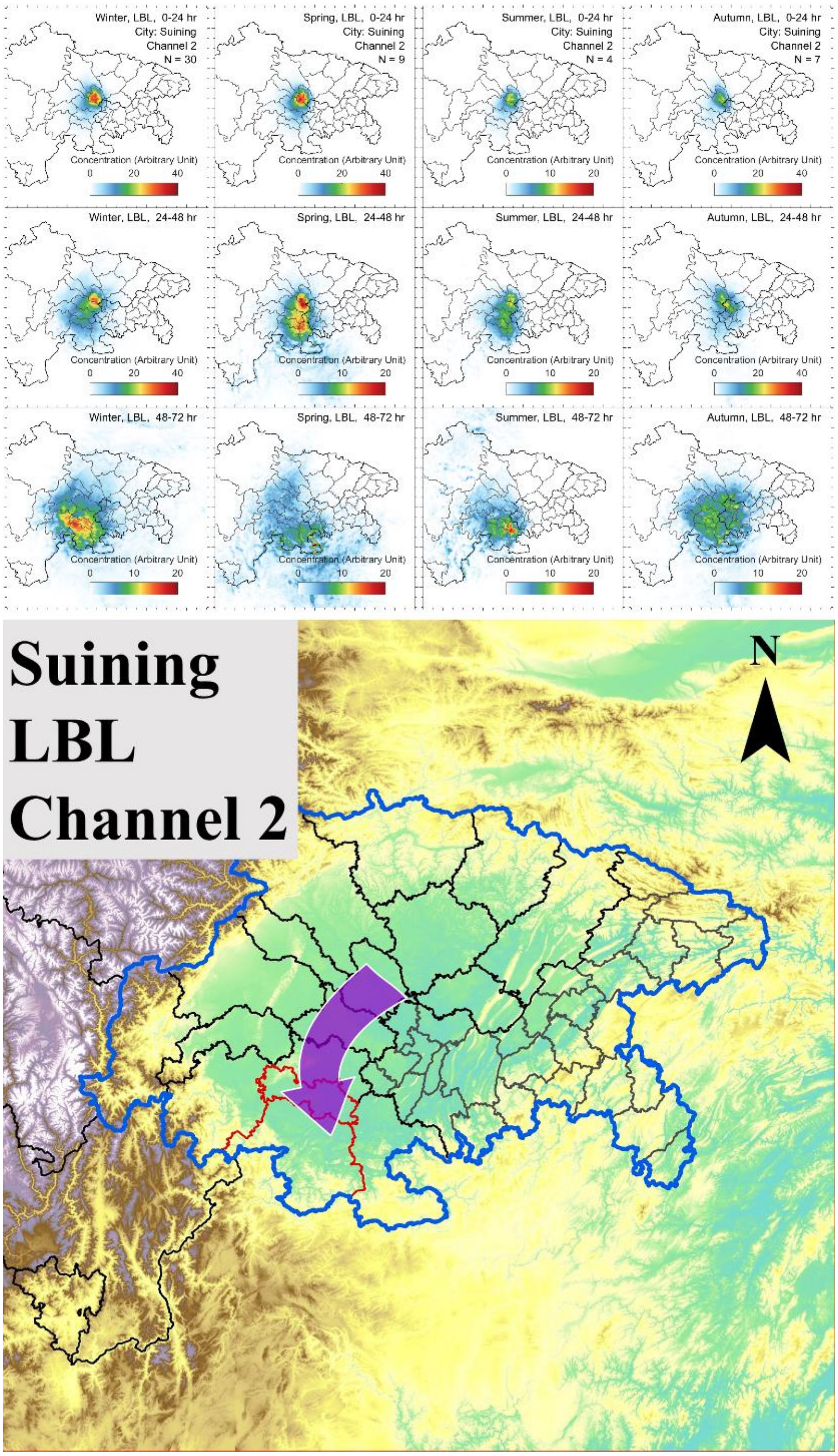

Figure S41 Channel 2 of Suining at LBL.

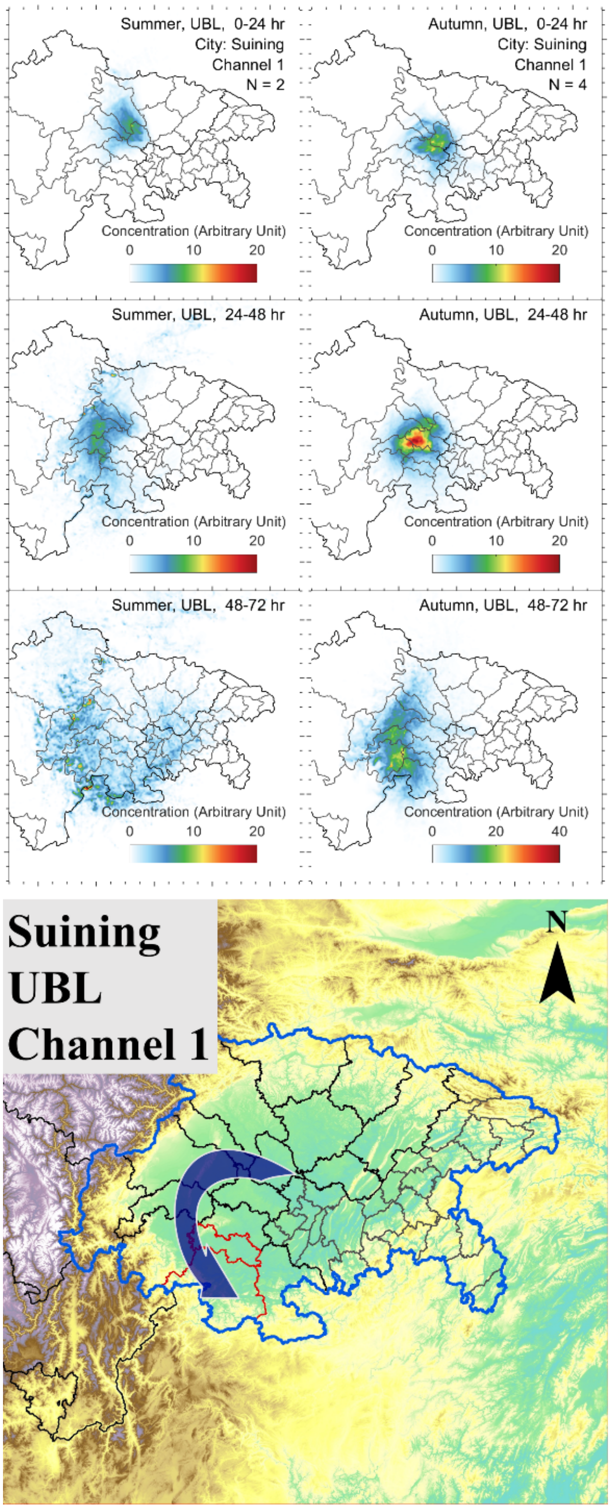

Figure S42 Channel 1 of Suining at UBL.

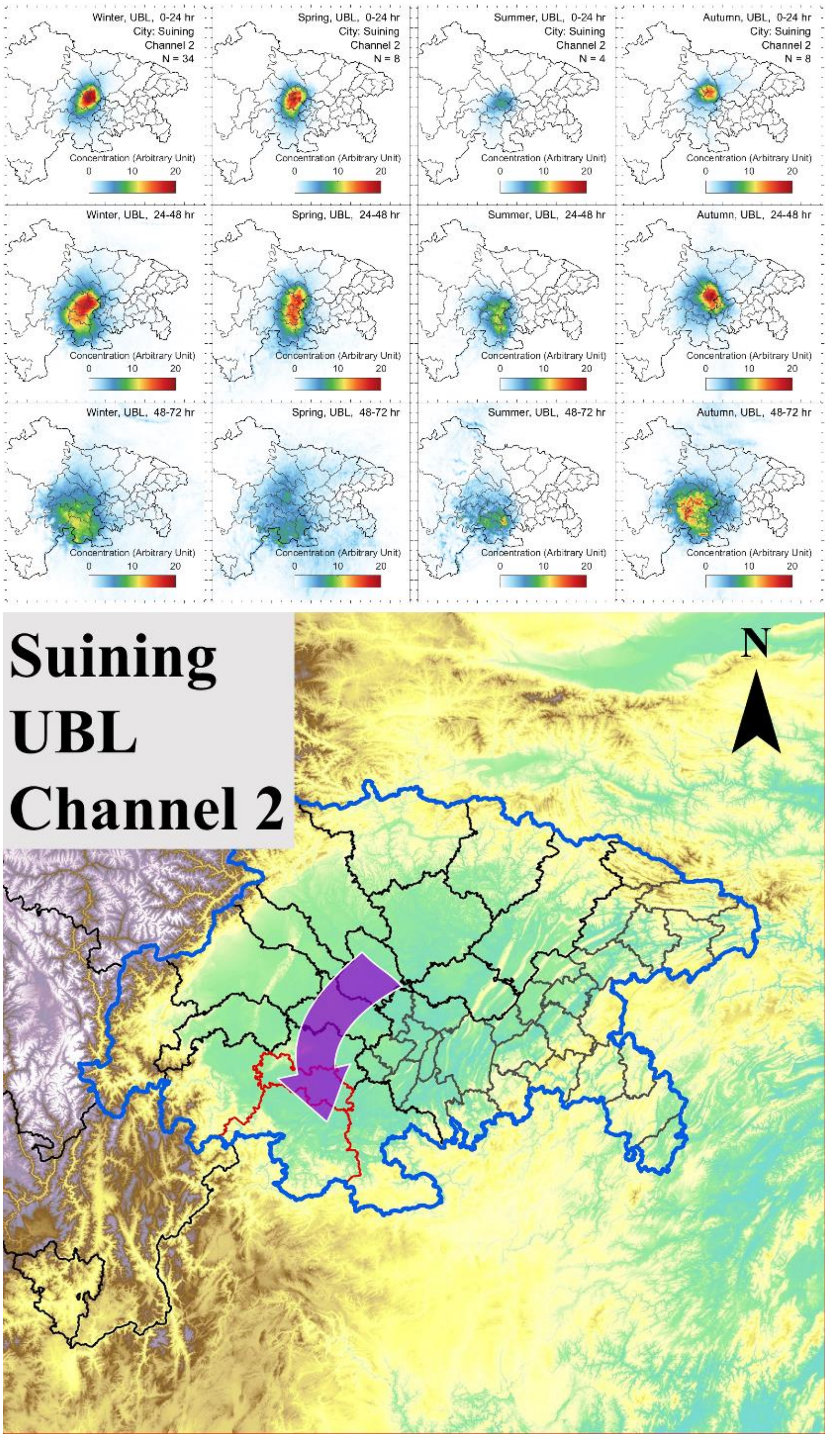

Figure S43 Channel 2 of Suining at UBL.

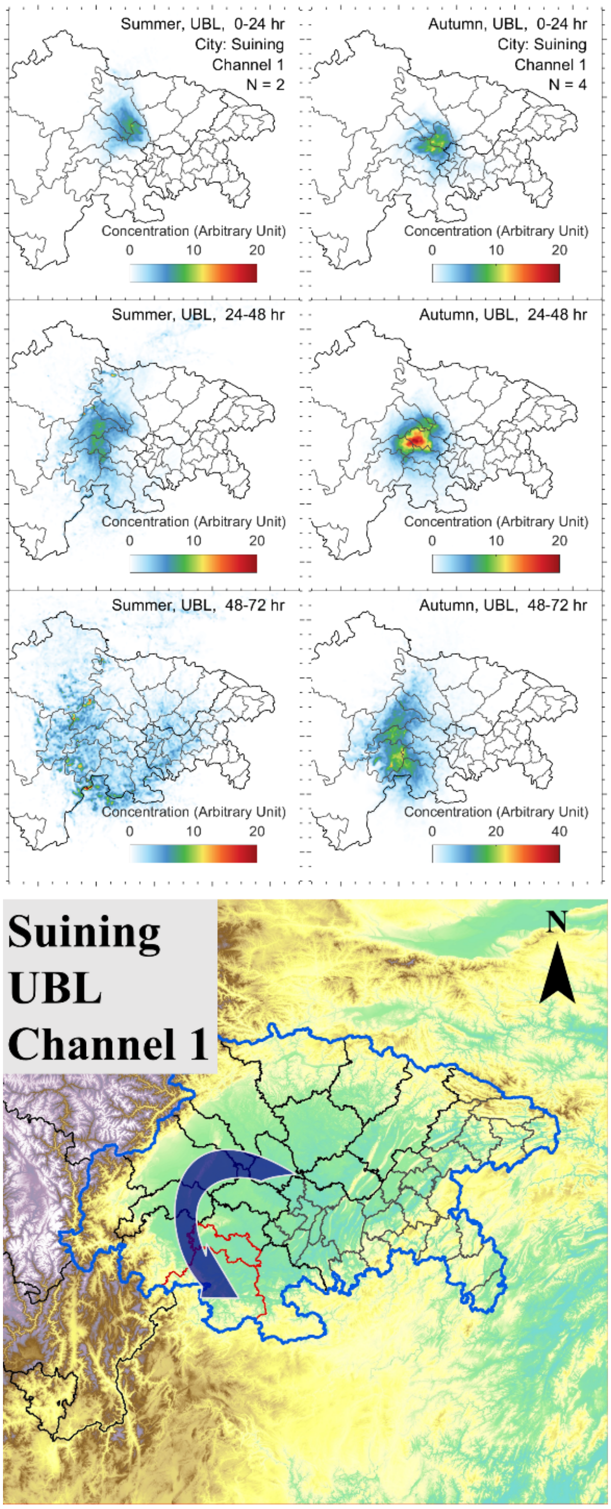

Figure S44 Channel 1 of Suining at UBL.

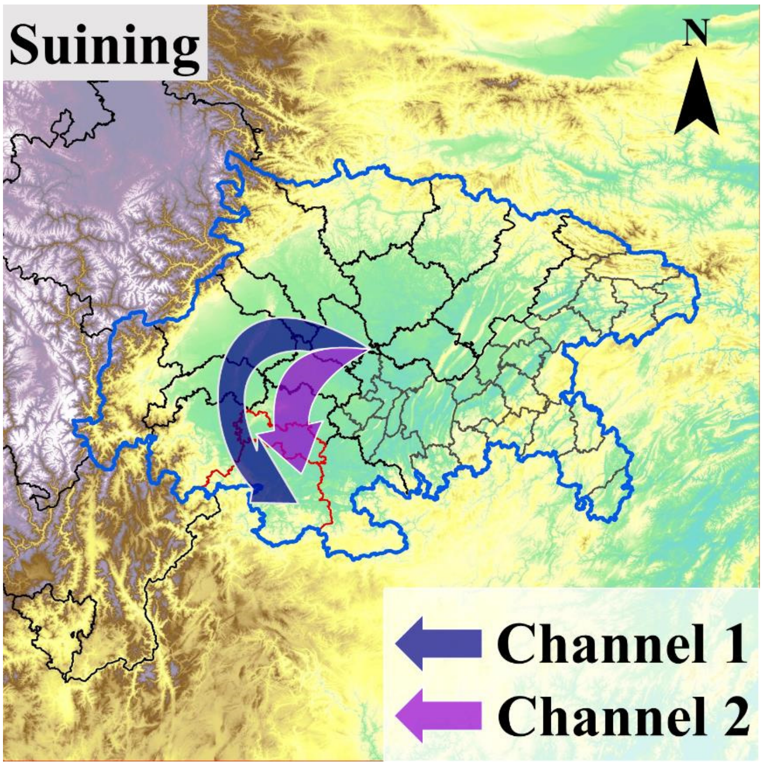

Figure S45 The identified 2 channels originating in.

Table S8 Occurrence frequencies of each channel originating in Suining in four seasons.

City	Channel	Layer	Season			
			autumn	spring	summer	winter
Suining	1	LBL			3.2%	
		UBL	12.9%		6.5%	
	2	LBL	22.6%	30.0%	12.9%	96.8%
		UBL	25.8%	26.7%	12.9%	109.7%

2.4 Chengdu

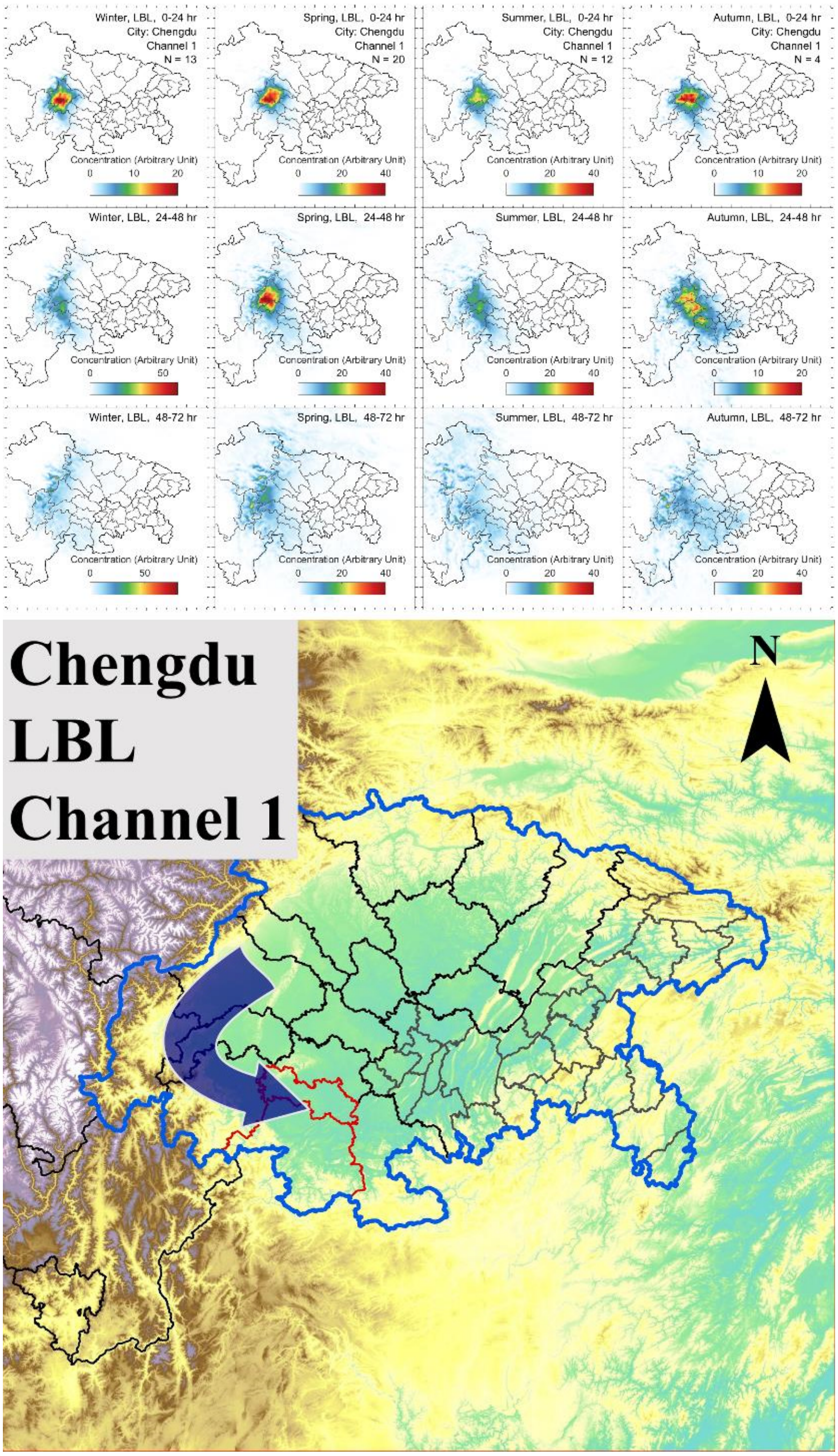

Figure S46 Channel 1 of Suining at LBL.

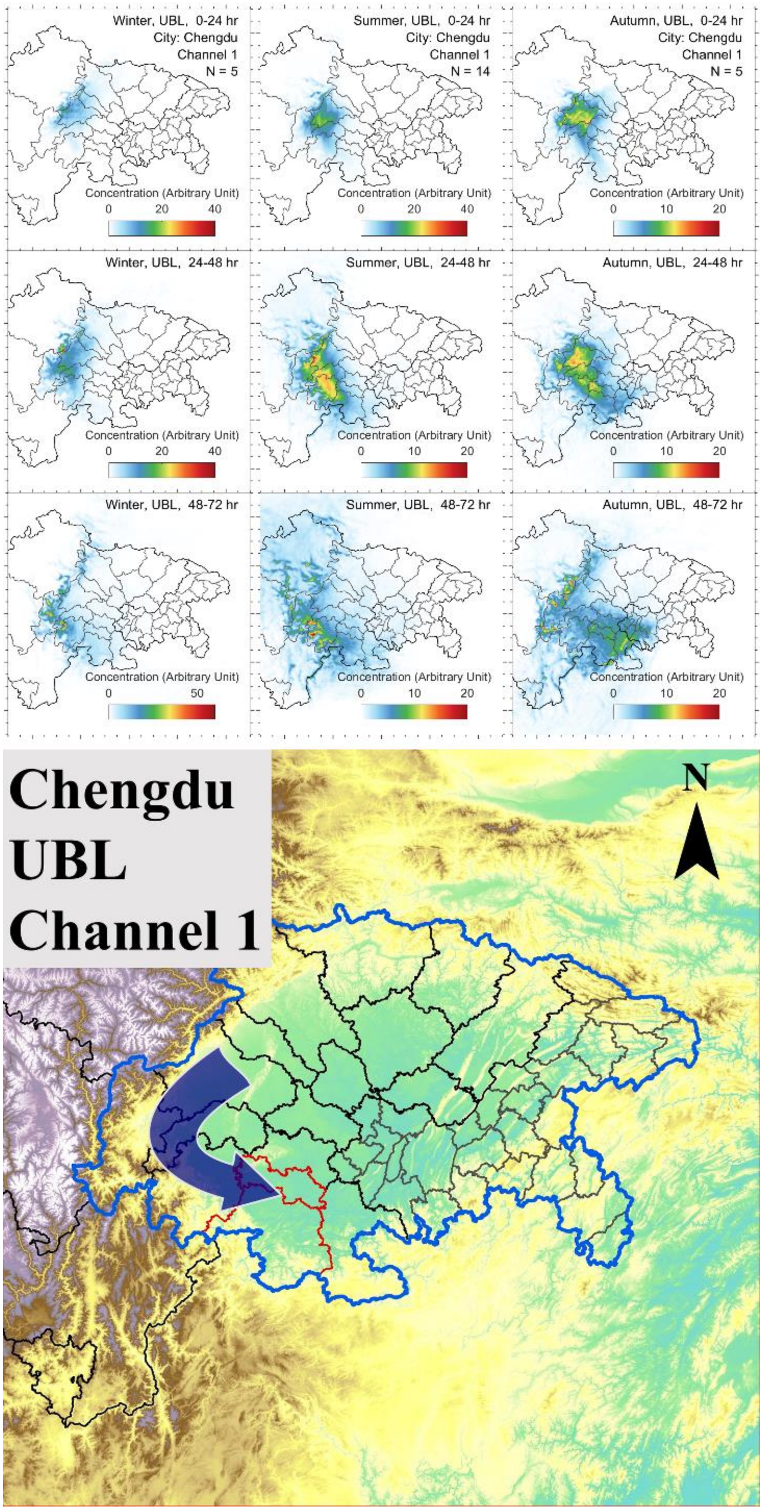

Figure S47 Channel 1 of Suining at UBL.

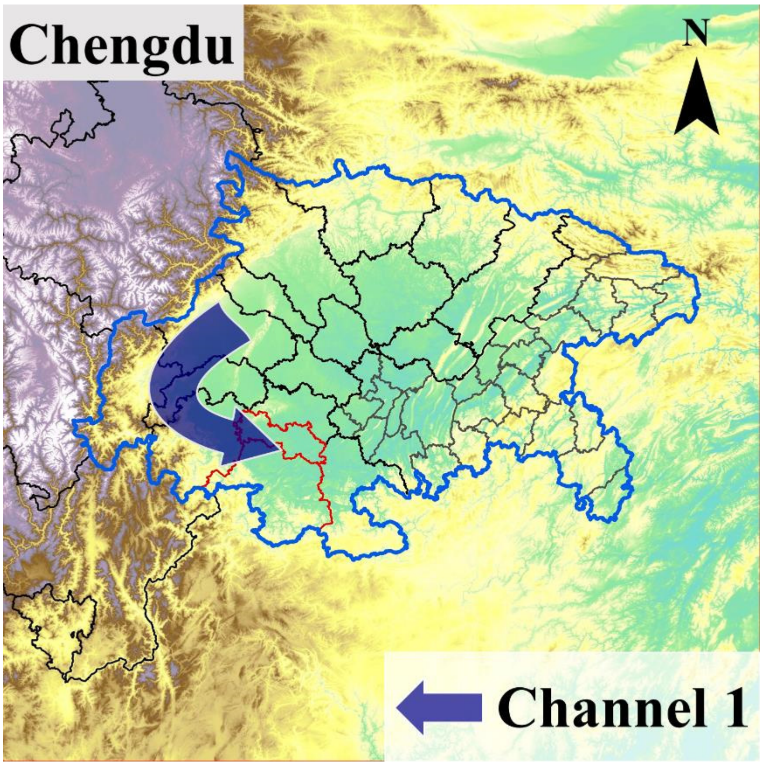

Figure S48 The identified channel originating in Chengdu.

Table S9 Occurrence frequency of the channel originating in Chengdu in four seasons.

City	Channel	Layer	Season			
			autumn	spring	summer	winter
Chengdu	1	LBL	12.9%	66.7%	38.7%	41.9%
		UBL	16.1%		45.2%	16.1%

2.5 Ziyang

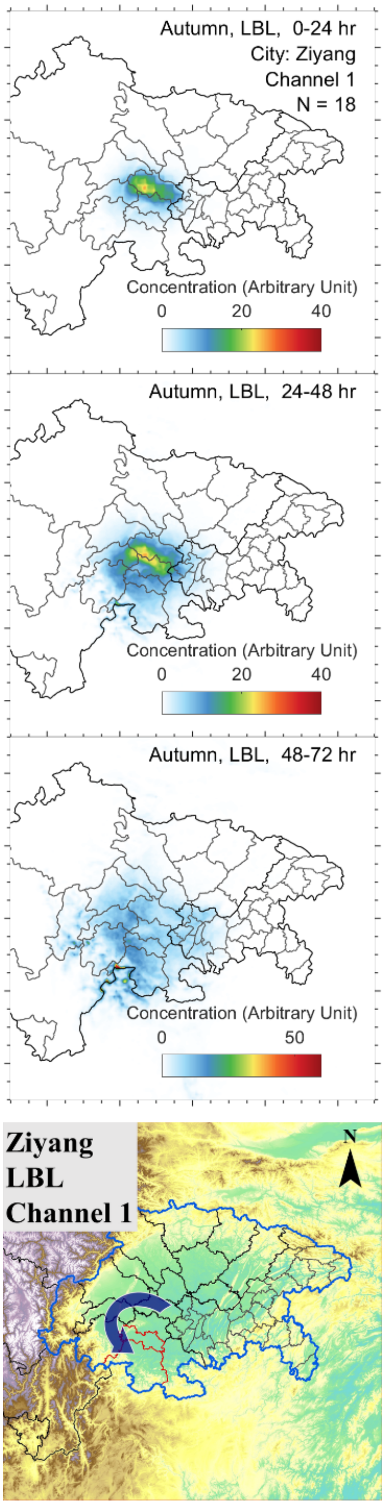

Figure S49 Channel 1 of Ziyang at LBL.

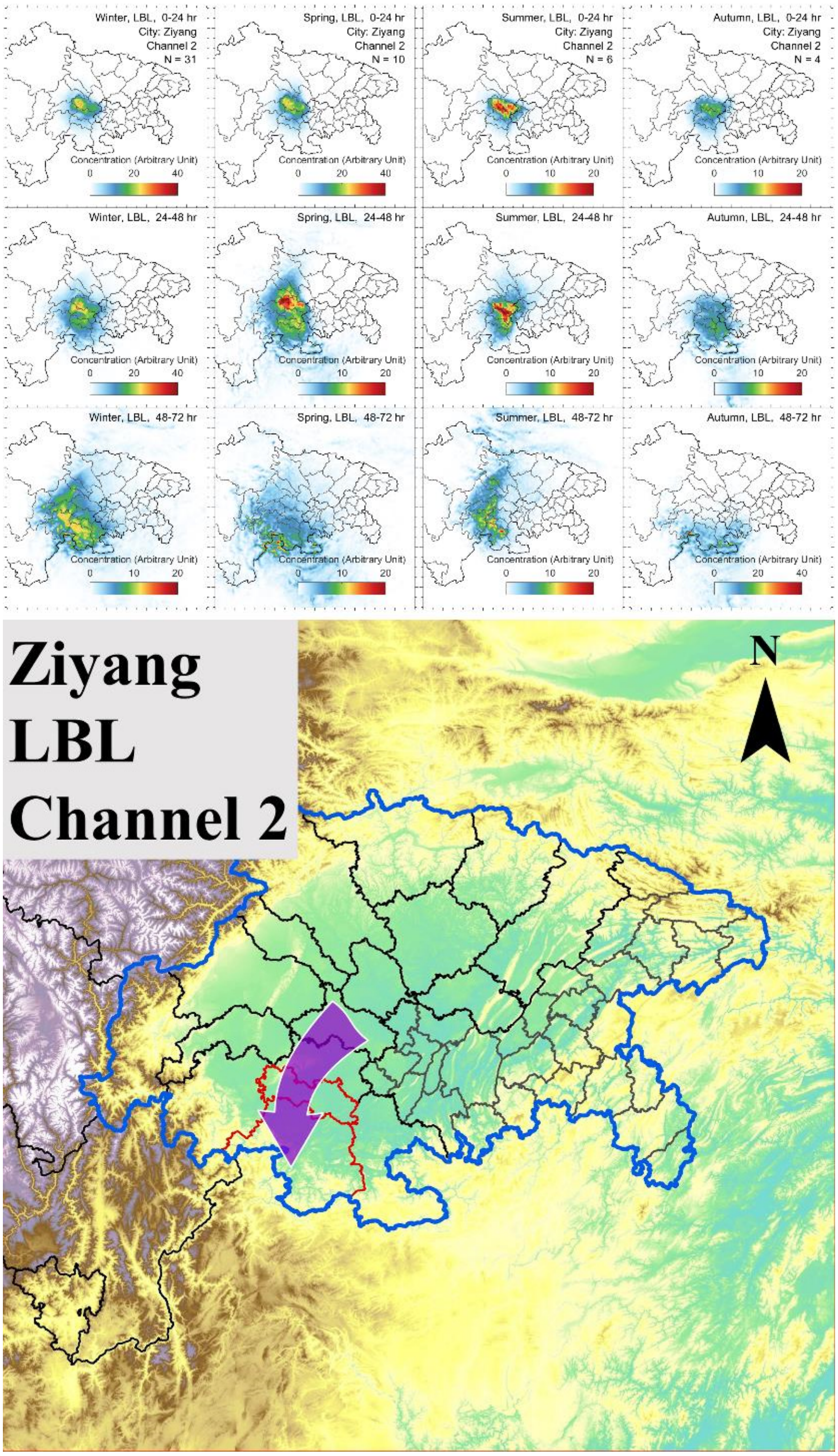

Figure S50 Channel 2 of Ziyang at LBL.

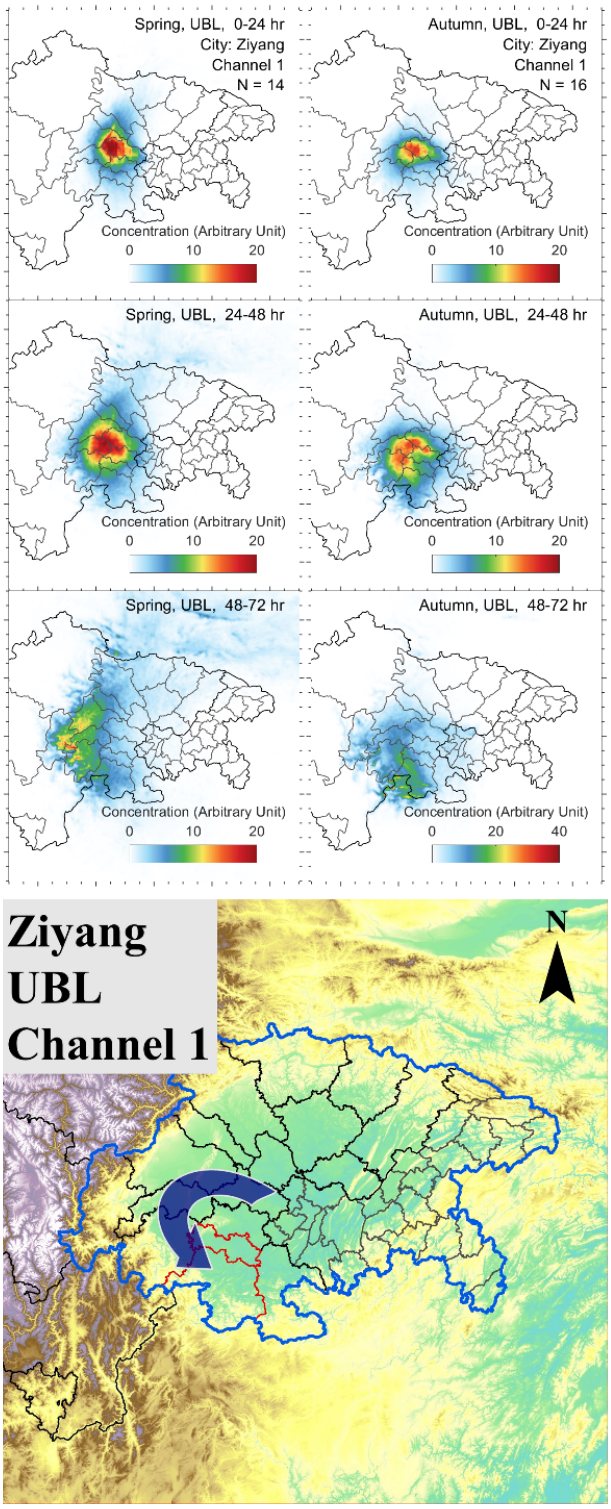

Figure S51 Channel 1 of Ziyang at UBL.

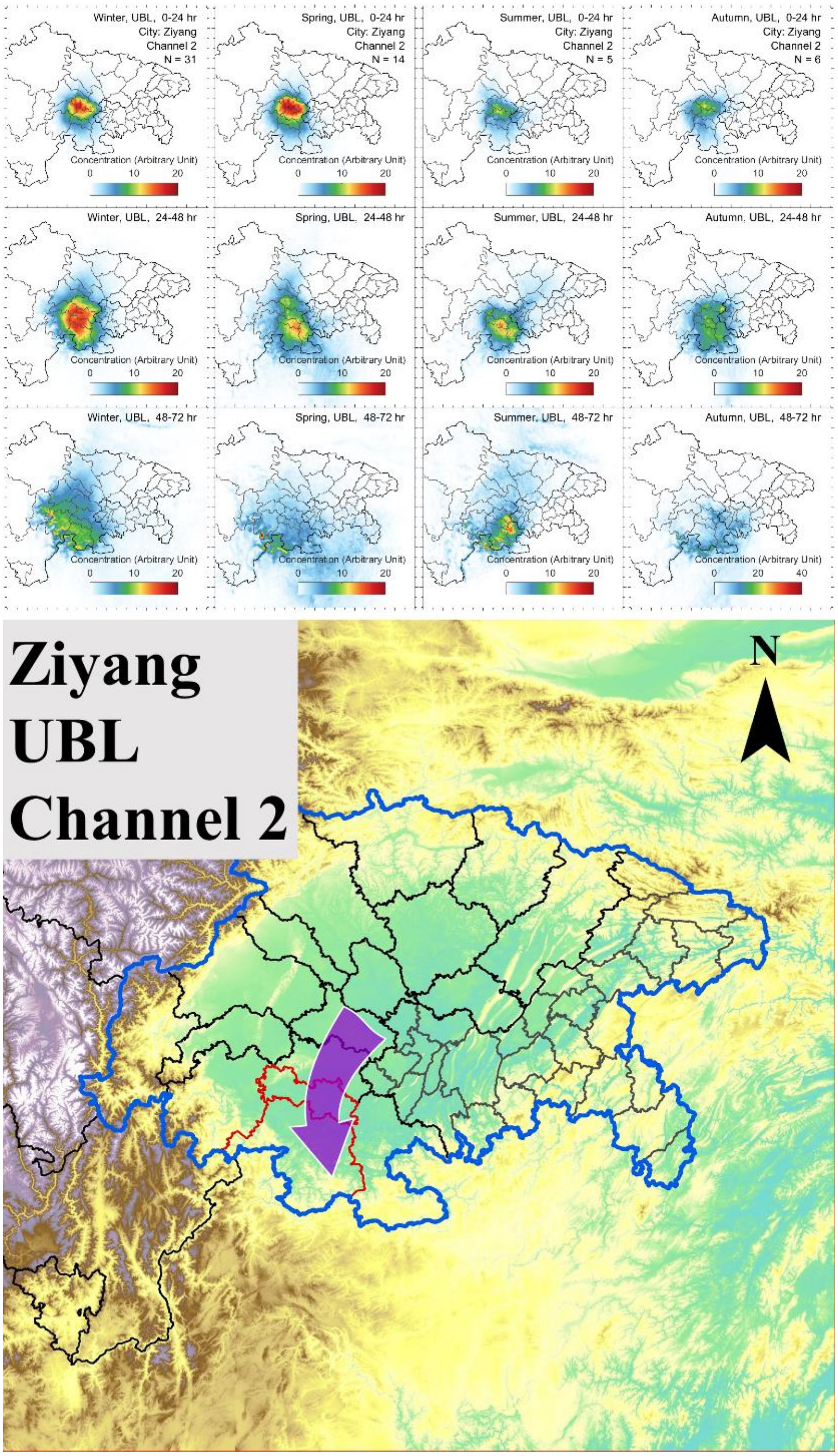

Figure S52 Channel 2 of Ziyang at UBL.

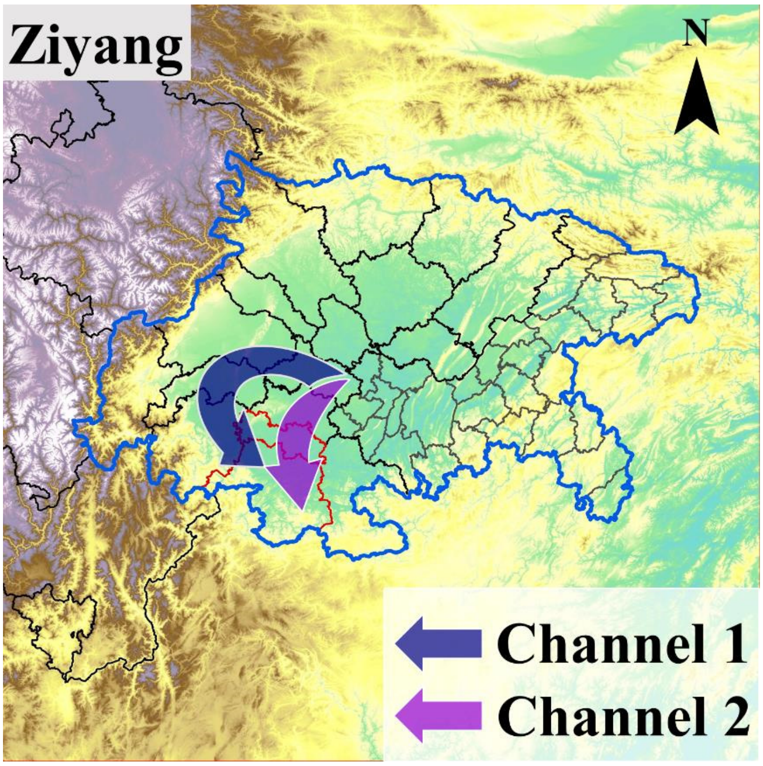

Figure S53 The identified 2 channels originating in Ziyang.

Table S10 Occurrence frequencies of each channel originating in Ziyang in four seasons.

City	Channel	Layer	Season			
			autumn	spring	summer	winter
Ziyang	1	LBL	58.1%			
		UBL	51.6%	46.7%		
	2	LBL	12.9%	33.3%	19.4%	100.0%
		UBL	19.4%	46.7%	16.1%	100.0%

2.6 Meishan

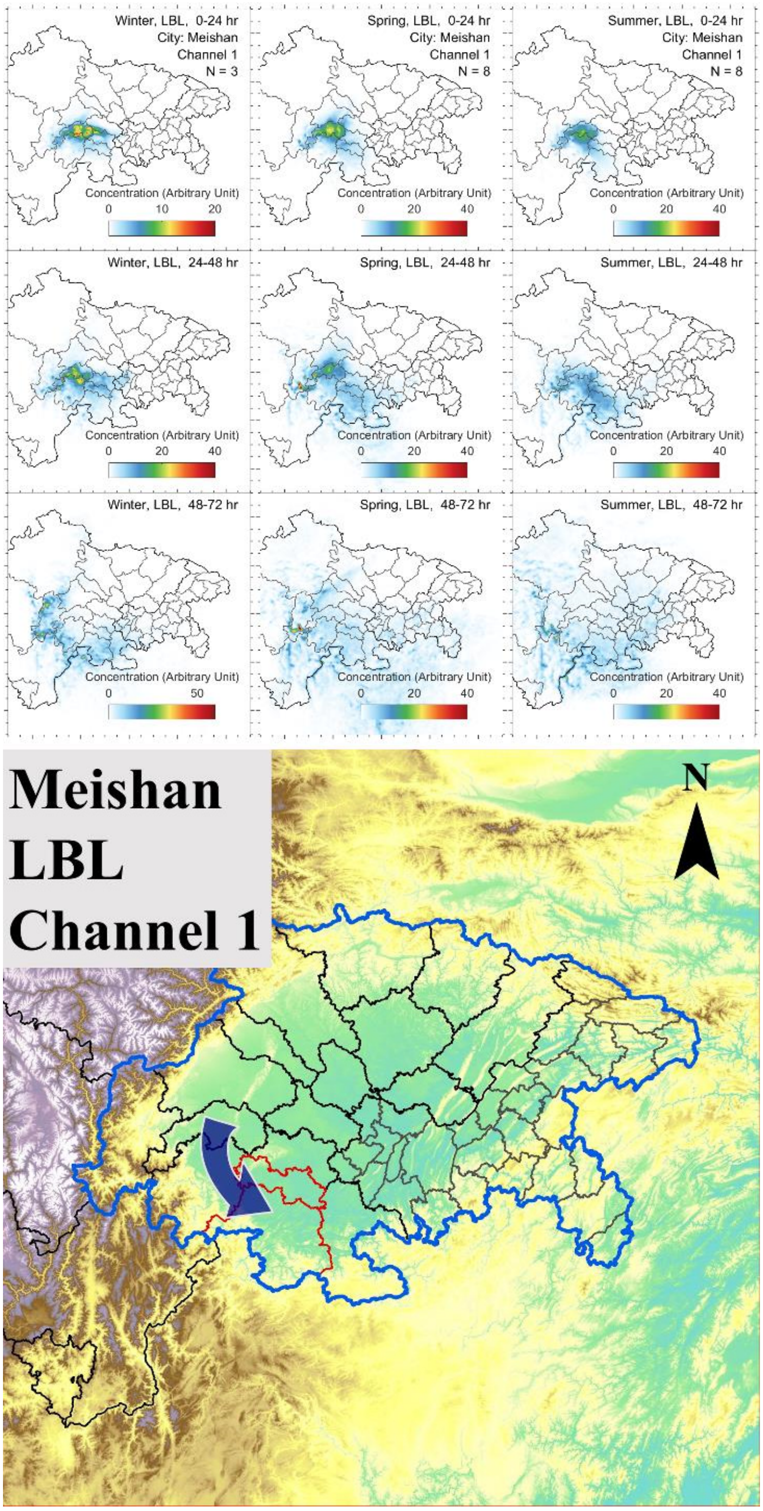

Figure S54 Channel 1 of Meishan at LBL.

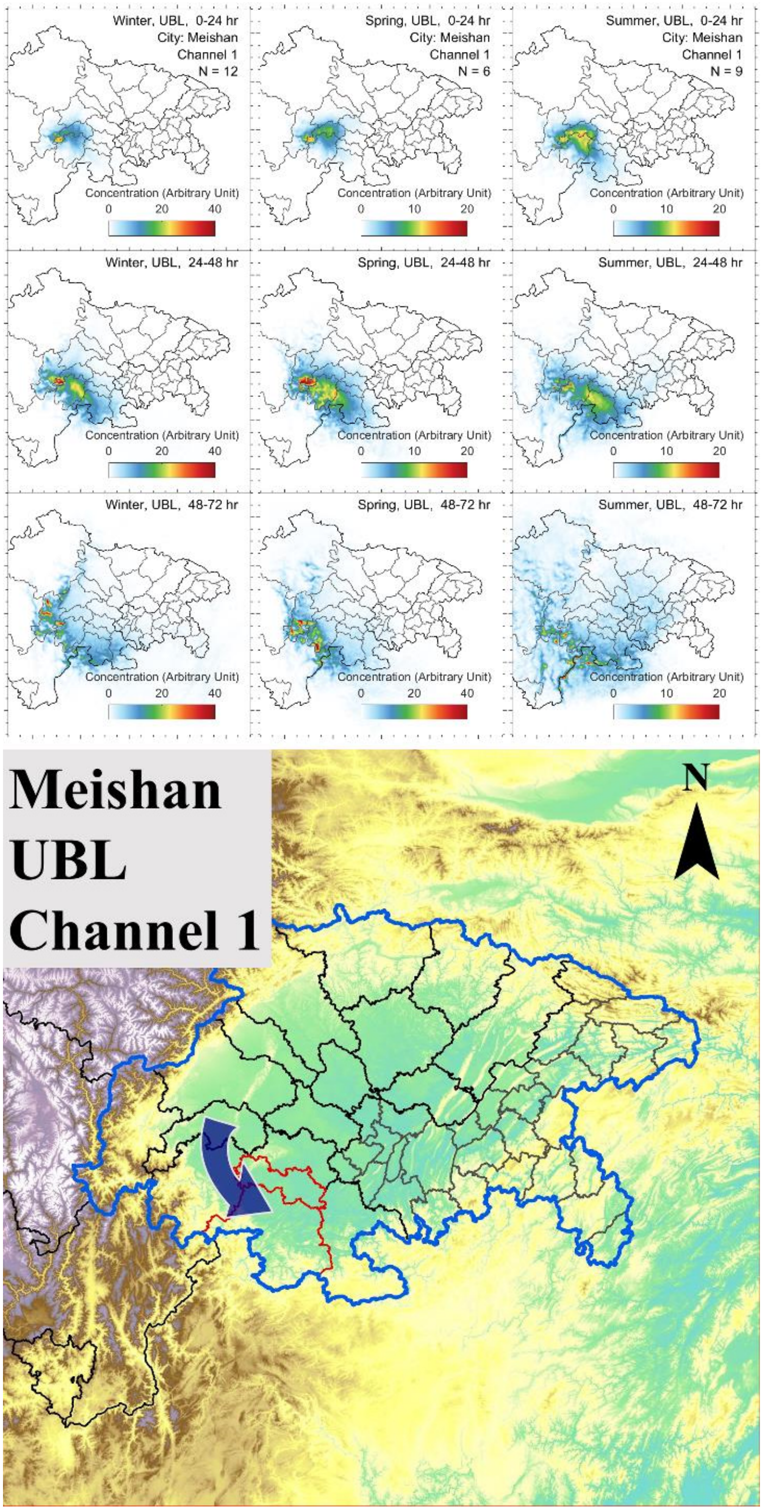

Figure S55 Channel 1 of Meishan at UBL.

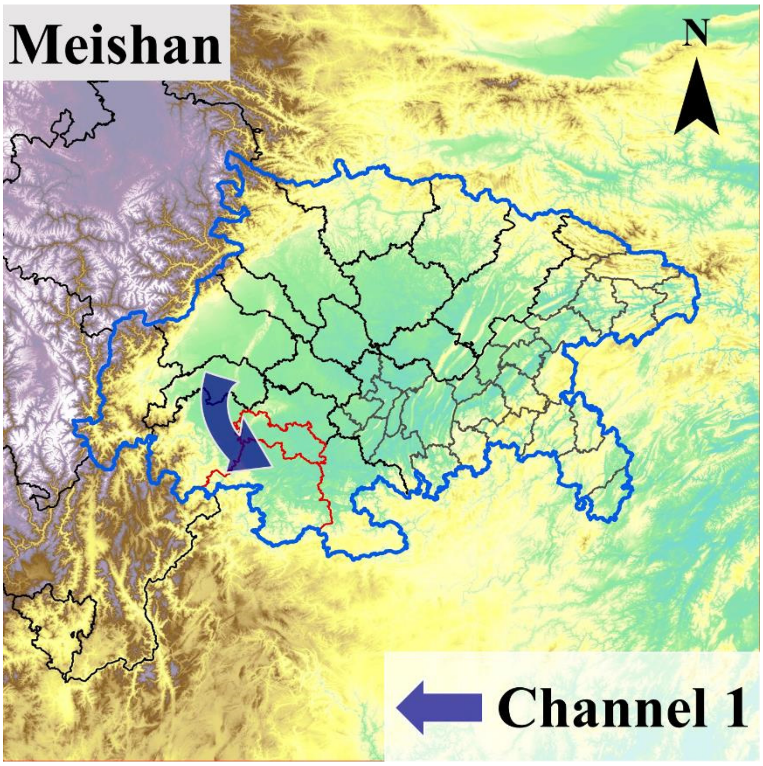

Figure S56 The identified channel originating in Meishan.

Table S11 Occurrence frequency of the channel originating in Meishan in four seasons.

City	Channel	Layer	Season			
			autumn	spring	summer	winter
Meishan	1	LBL		26.7%	25.8%	9.7%
		UBL		20.0%	29.0%	38.7%

2.7 Leshan

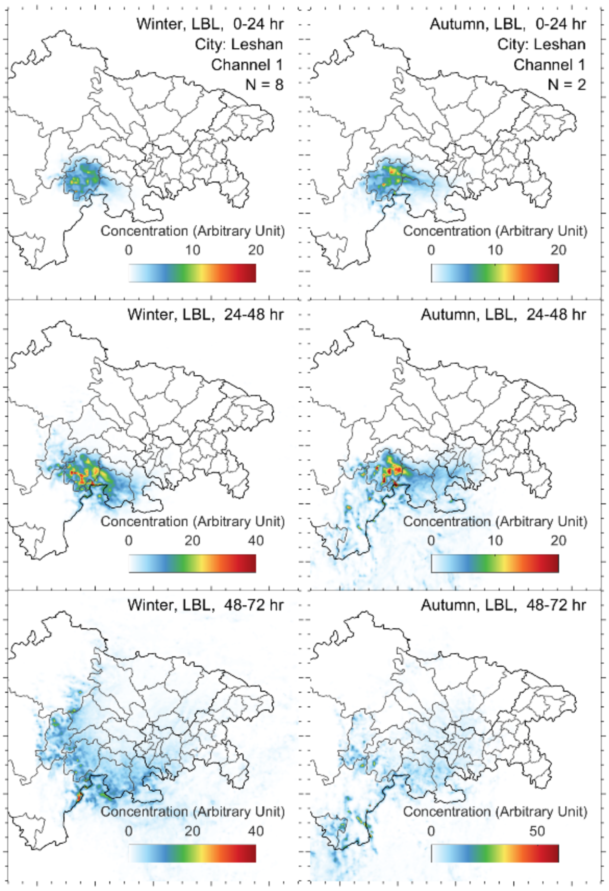

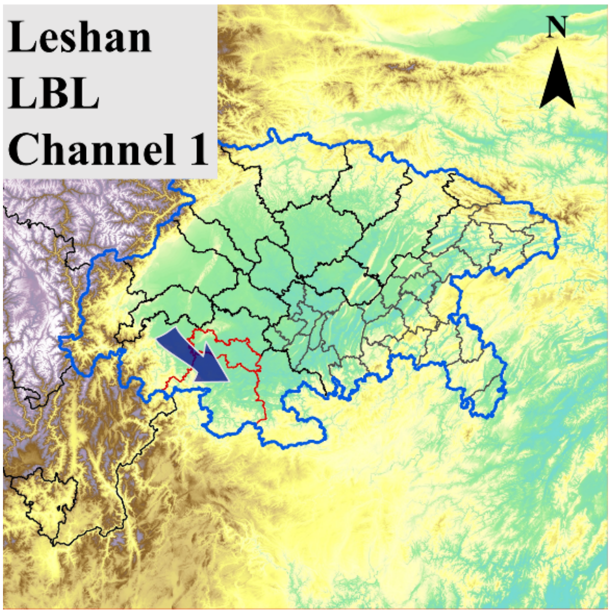

Figure S57 Channel 1 of Leshan at LBL.

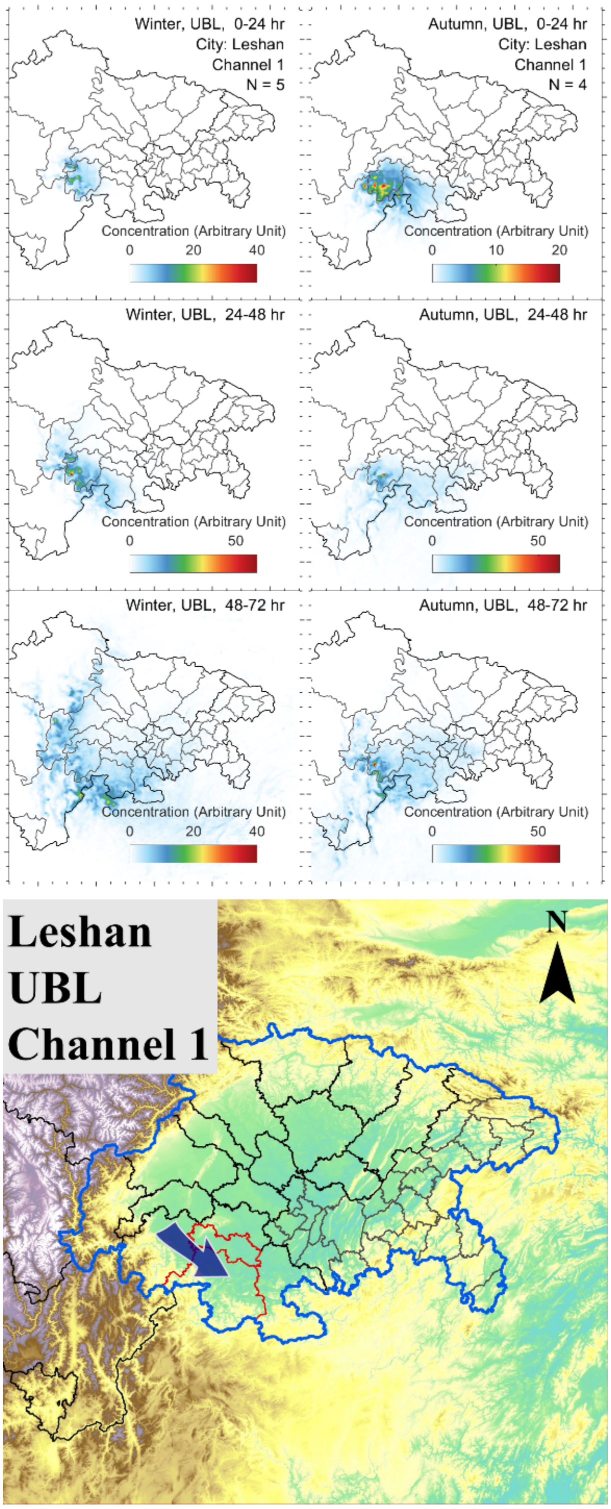

Figure S58 Channel 1 of Leshan at UBL.

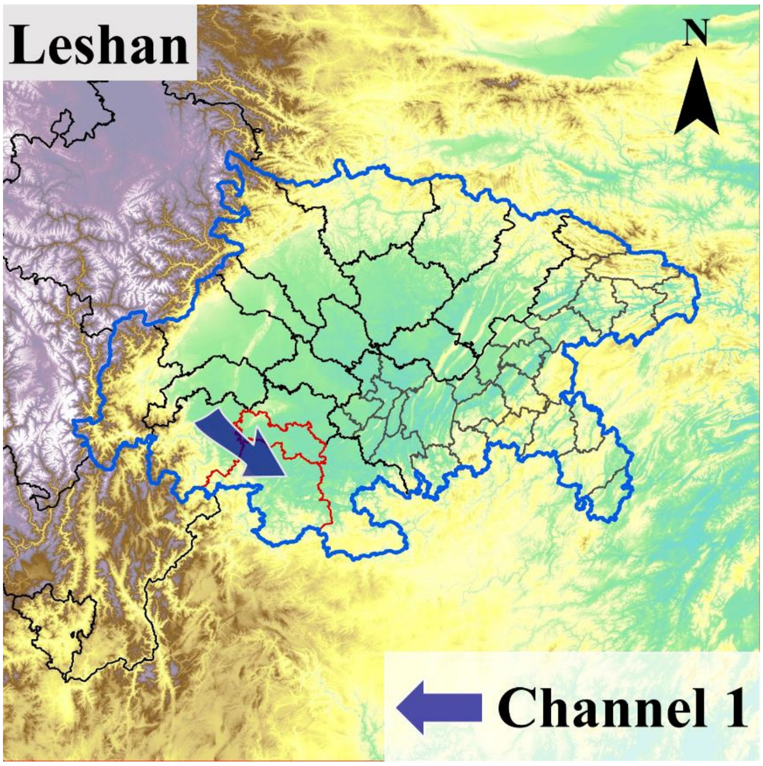

Figure S59 The identified channel originating in Leshan.

Table S12 Occurrence frequency of the channel originating in Leshan in four seasons.

City	Channel	Layer	Season			
			autumn	spring	summer	winter
Leshan	1	LBL	6.5%			25.8%
		UBL	12.9%			16.1%

3. Southern Sichuan Urban Agglomeration

3.1 Neijiang

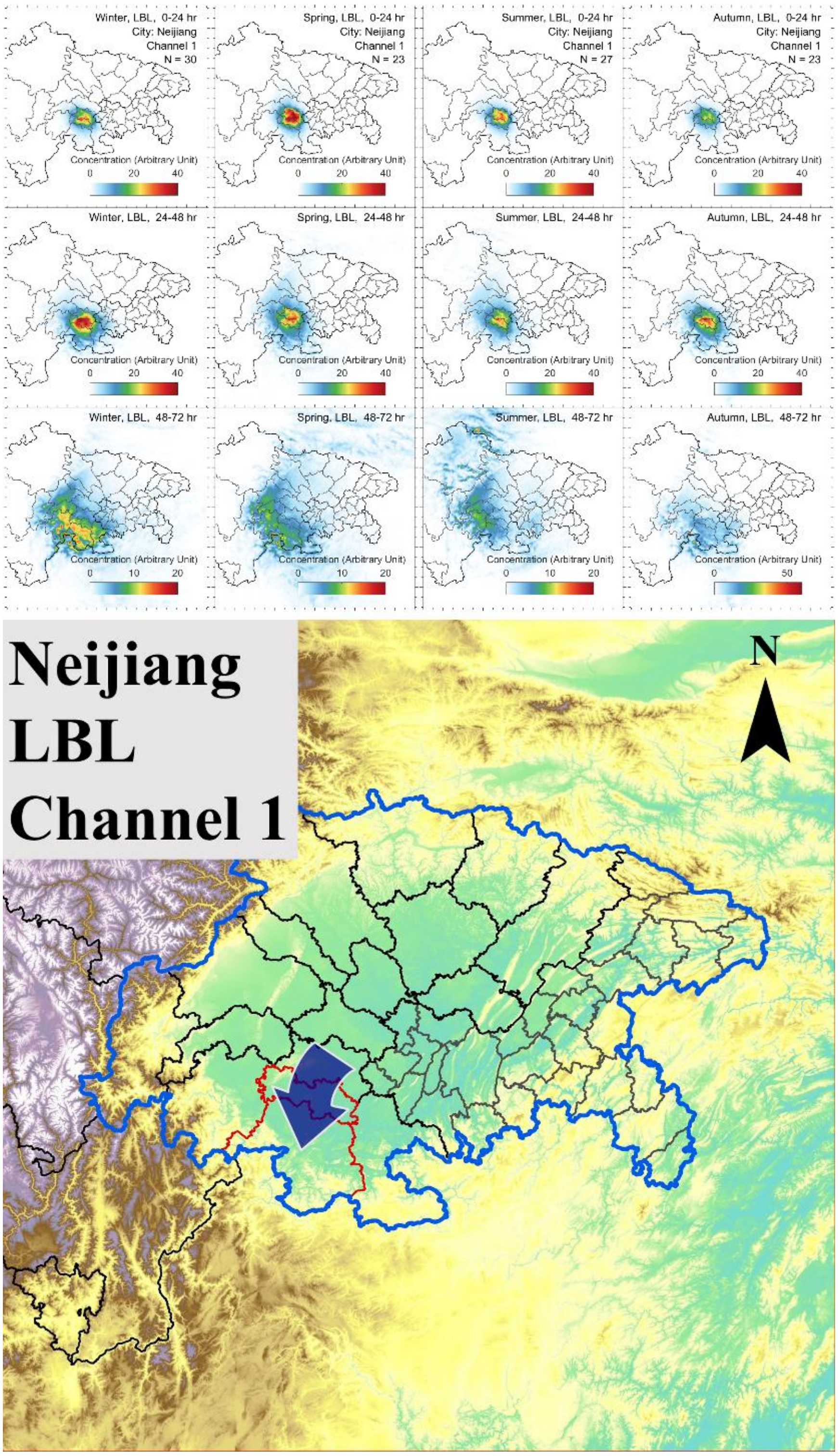

Figure S60 Channel 1 of Neijiang at LBL.

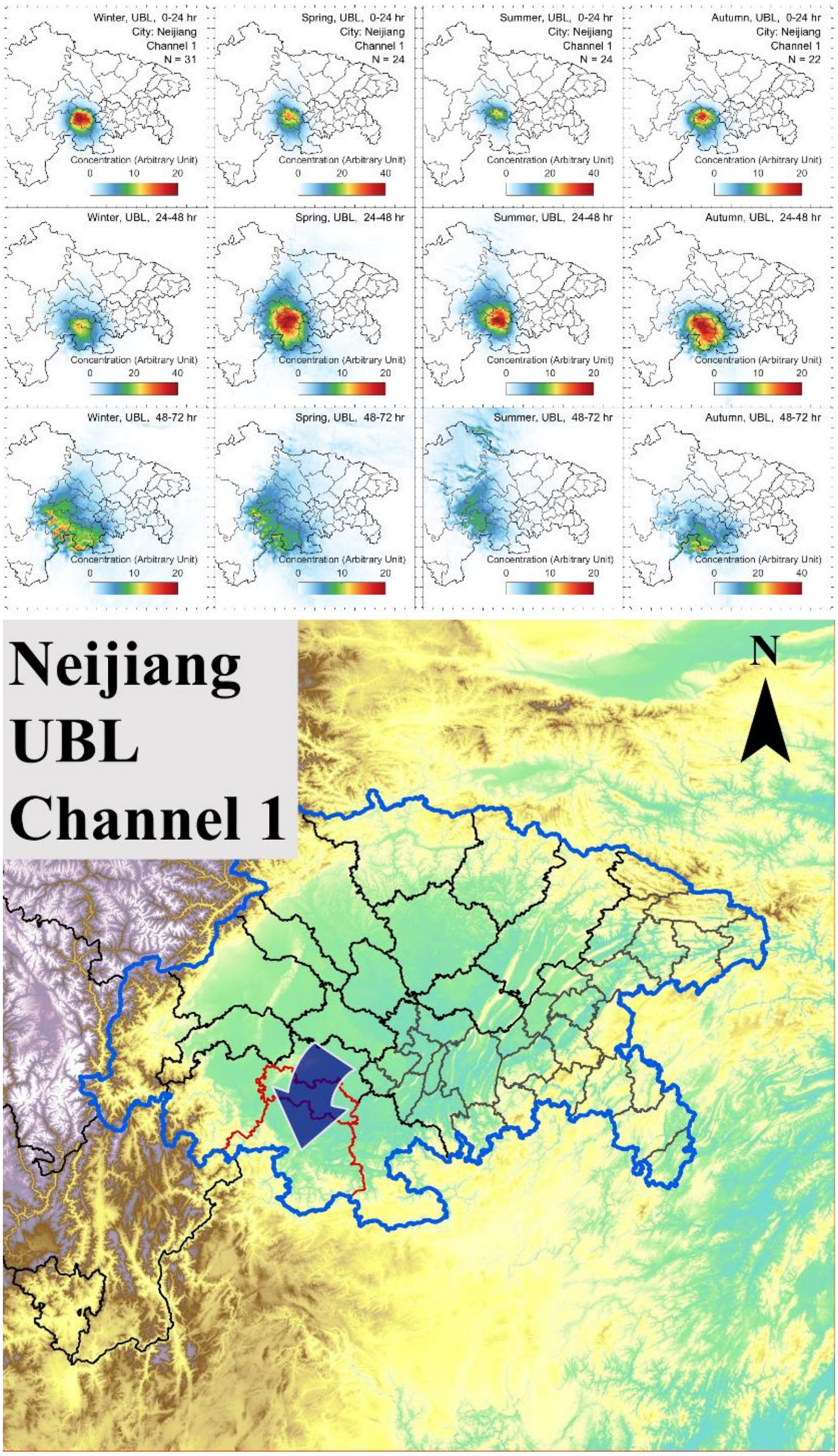

Figure S61 Channel 1 of Neijiang at UBL.

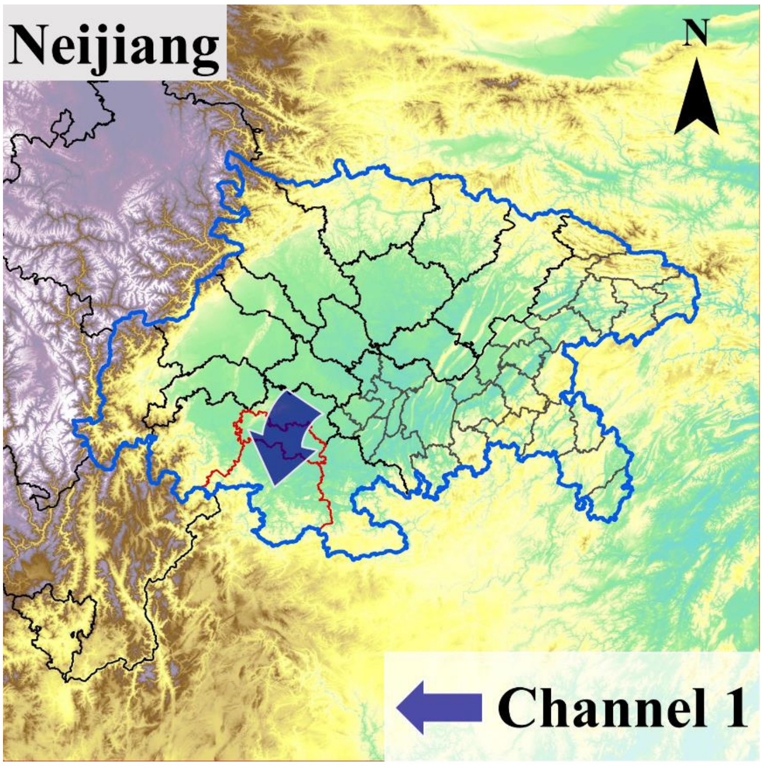

Figure S62 The identified channel originating in Neijiang.

Table S13 Occurrence frequency of the channel originating in Neijiang in four seasons.

City	Channel	Layer	Season			
			autumn	spring	summer	winter
Neijiang	1	LBL	74.2%	76.7%	87.1%	96.8%
		UBL	71.0%	80.0%	77.4%	100.0%

3.2 Luzhou

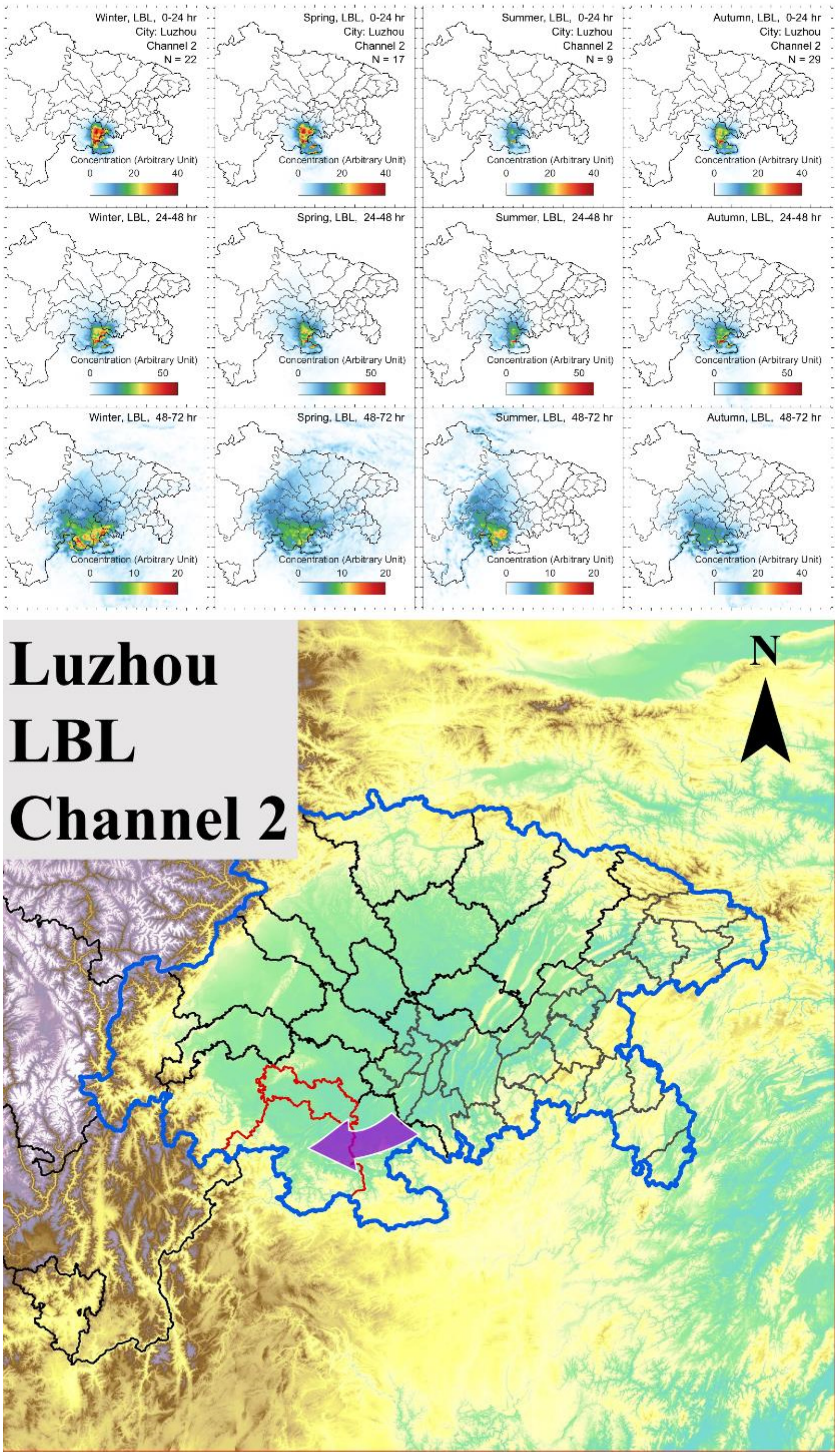

Figure S63 Channel 1 of Luzhou at LBL.

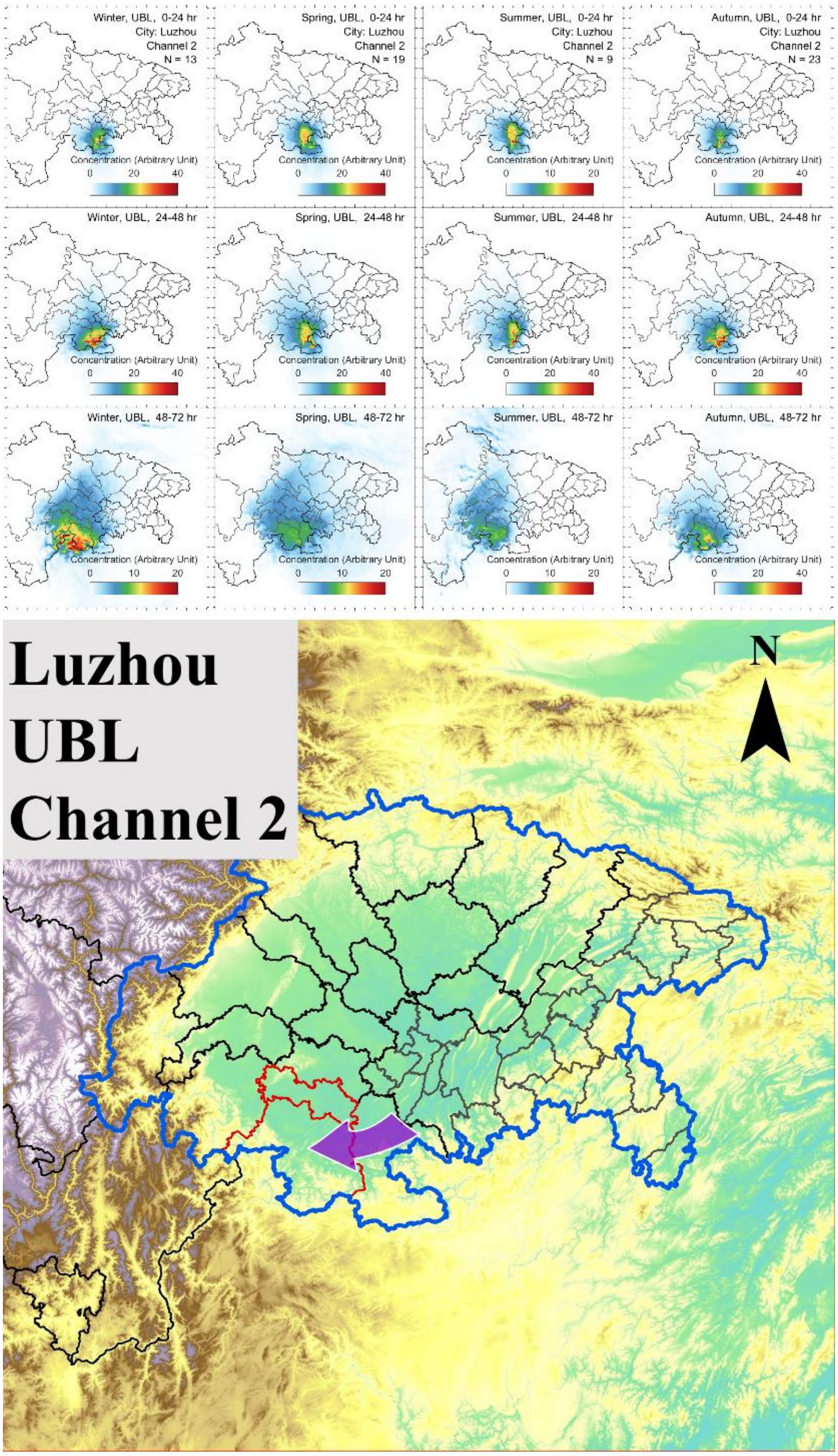

Figure S64 Channel 1 of Luzhou at UBL.

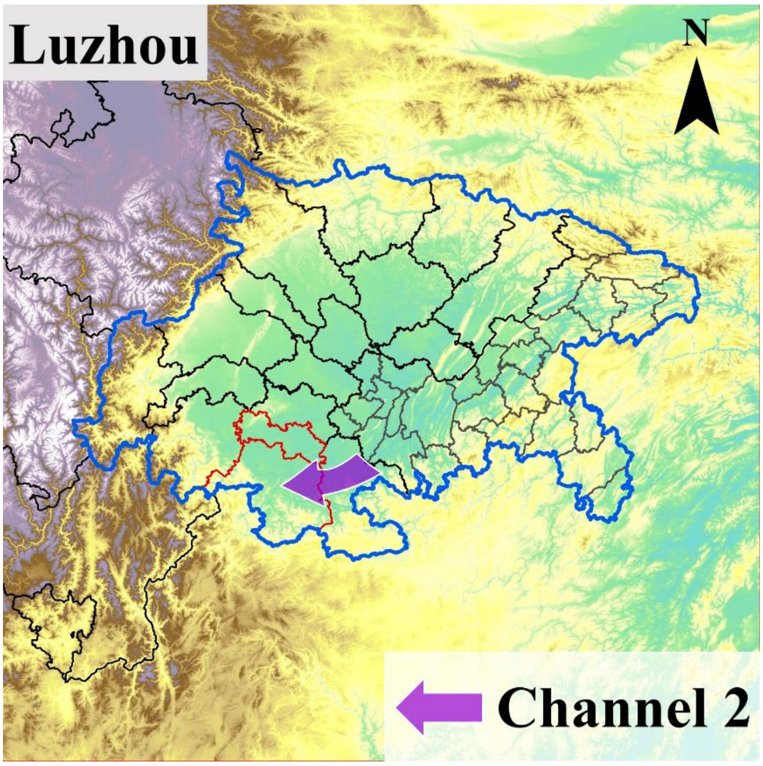

Figure S65 The identified channel originating in Luzhou.

Table S14 Occurrence frequency of the channel originating in Luzhou in four seasons.

City	Channel	Layer	Season			
			autumn	spring	summer	winter
Luzhou	2	LBL	93.5%	56.7%	29.0%	71.0%
		UBL	74.2%	63.3%	29.0%	41.9%

4. Northwest Chongqing Urban Agglomeration

4.1 Hechuan

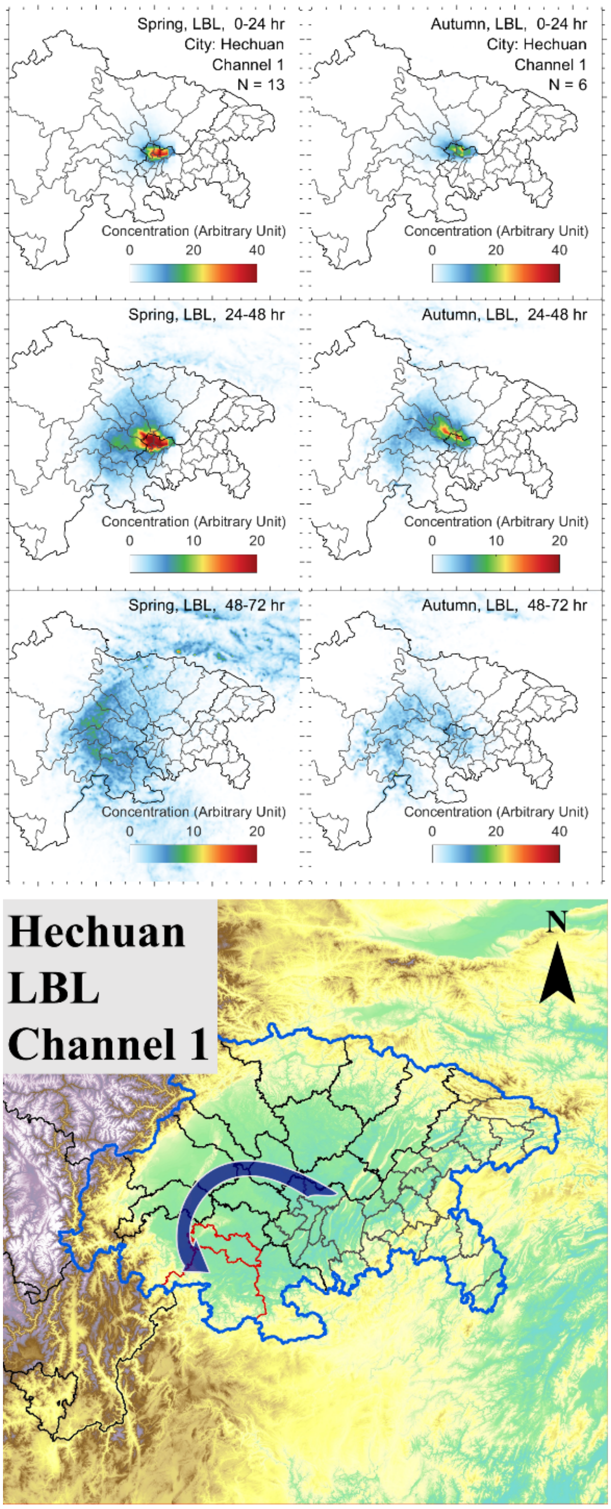

Figure S66 Channel 1 of Hechuan at LBL.

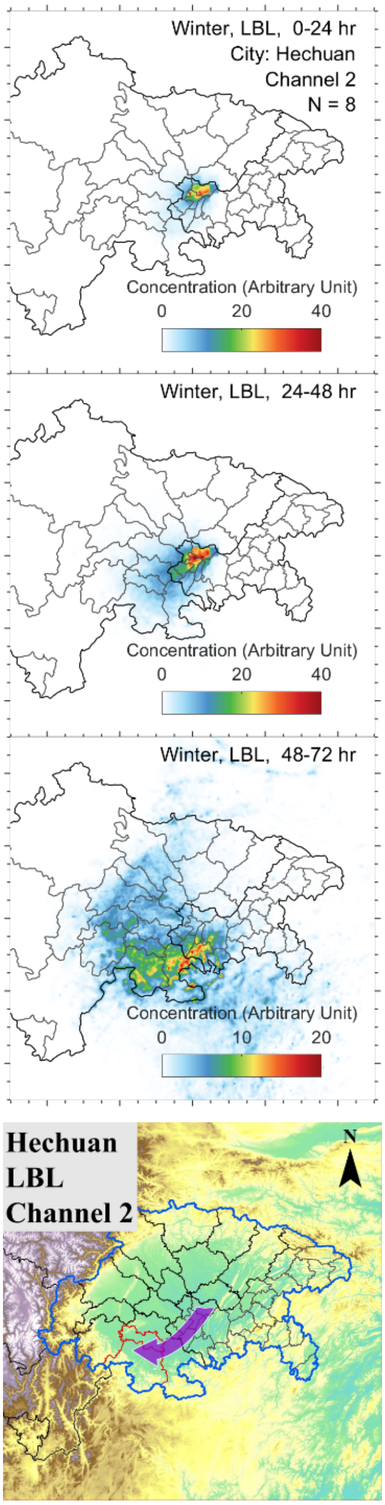

Figure S67 Channel 2 of Hechuan at LBL.

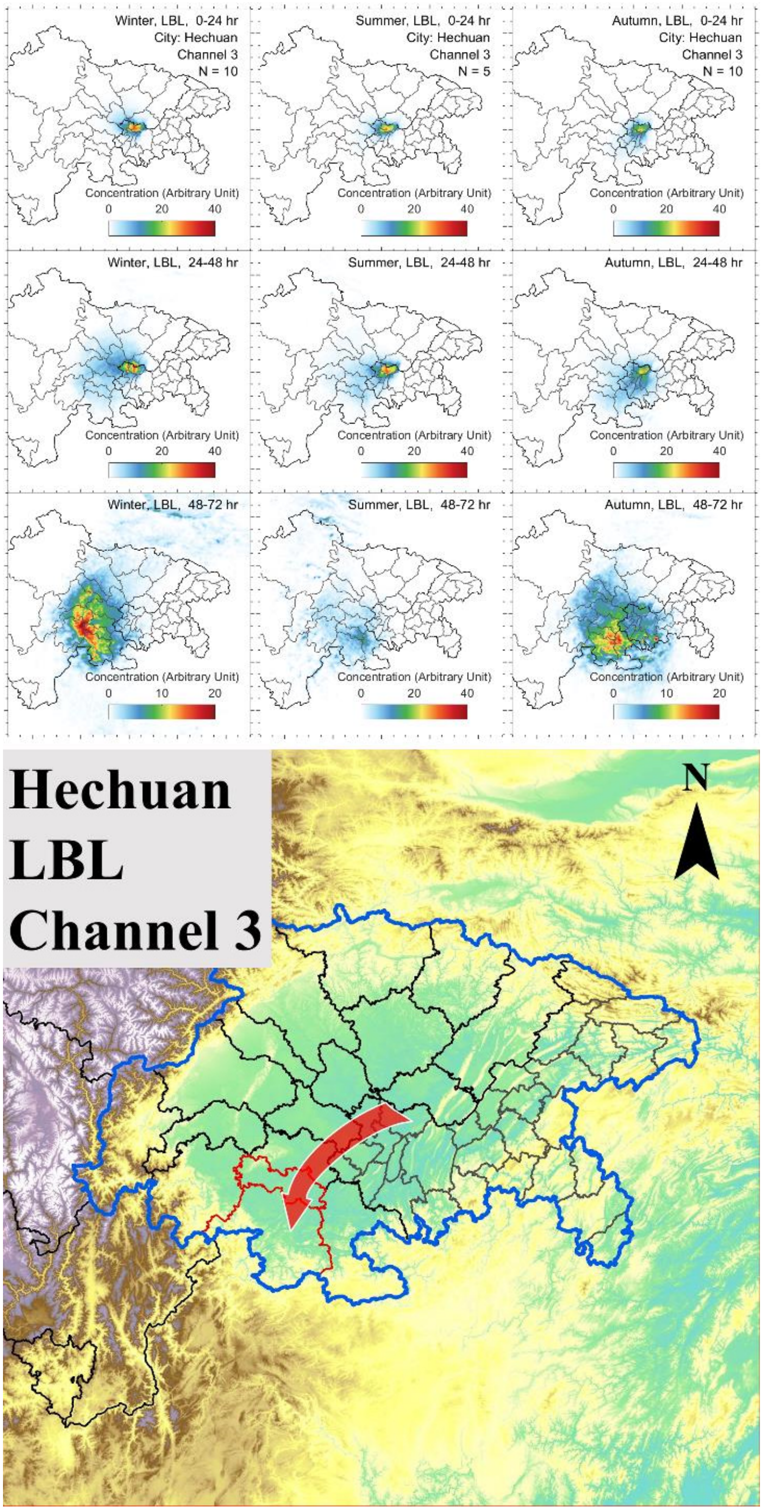

Figure S68 Channel 3 of Hechuan at LBL.

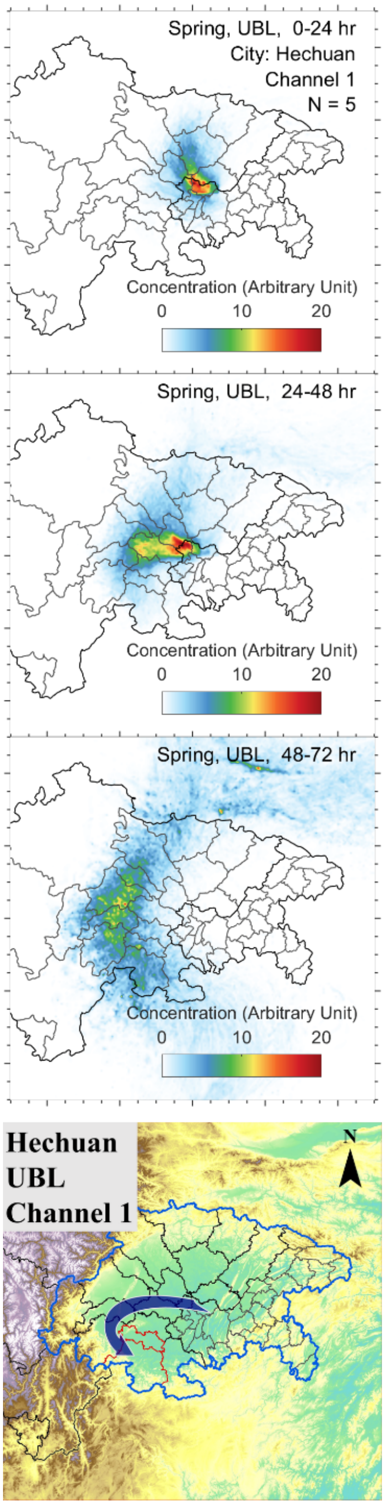

Figure S69 Channel 1 of Hechuan at UBL.

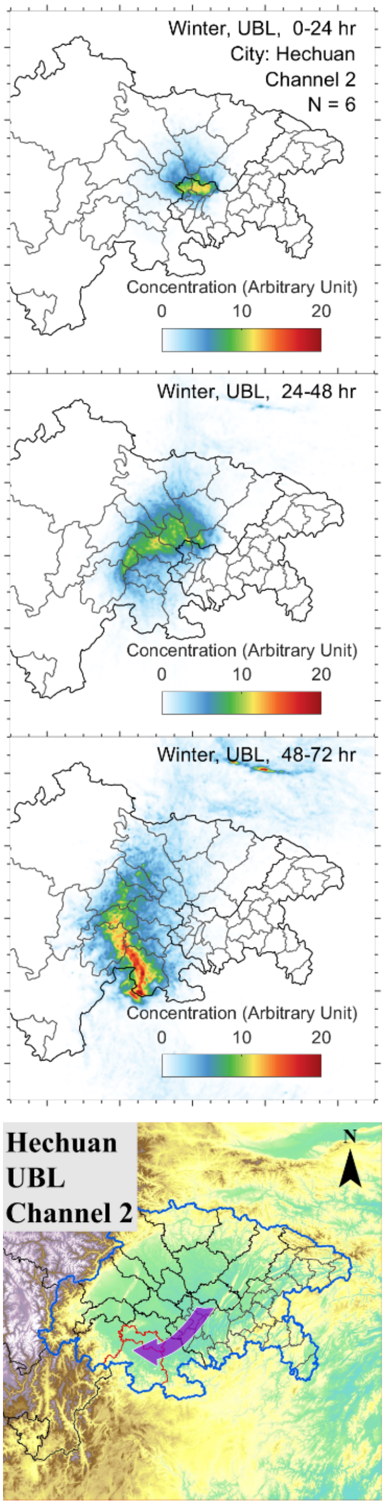

Figure S70 Channel 2 of Hechuan at UBL.

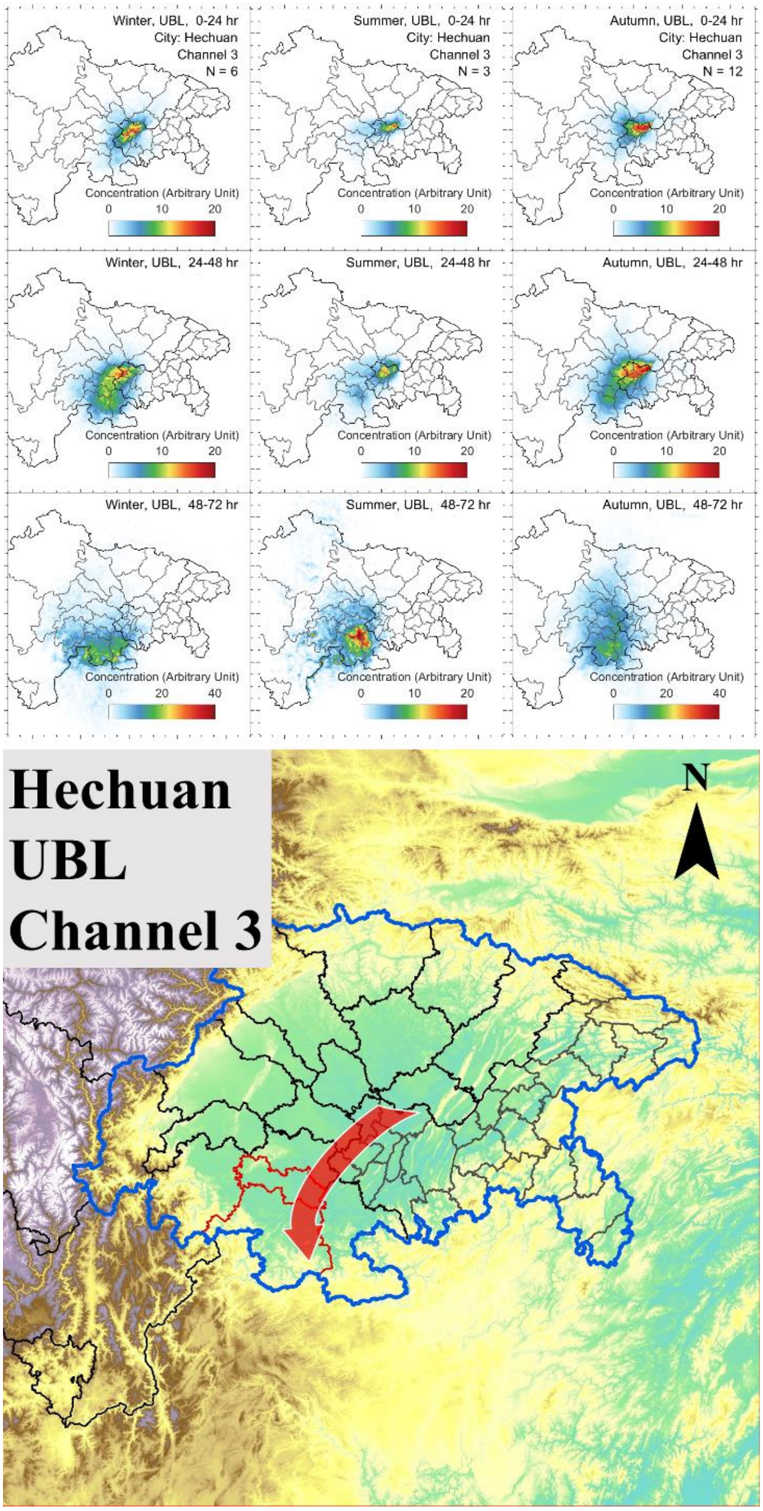

Figure S71 Channel 3 of Hechuan at UBL.

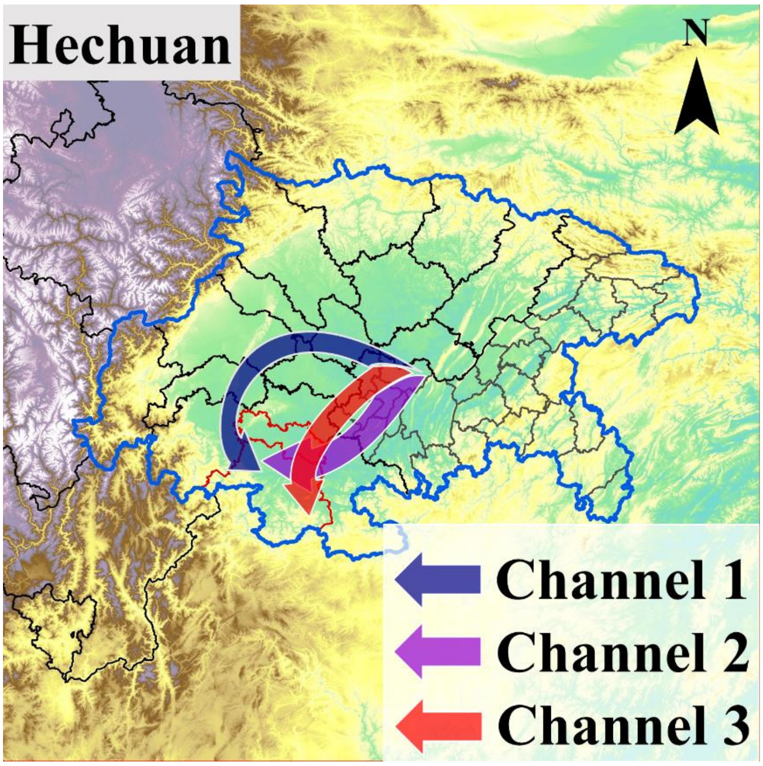

Figure S72 The identified 3 channels originating in Hechuan.

Table S15 Occurrence frequencies of each channel originating in Hechuan in four seasons.

City	Channel	Layer	Season			
			autumn	spring	summer	winter
Hechuan	1	LBL	19.4%	43.3%		
		UBL		16.7%		
	2	LBL				25.8%
		UBL				19.4%
	3	LBL	32.3%		16.1%	32.3%
		UBL	38.7%		9.7%	19.4%

4.2 Tongnan

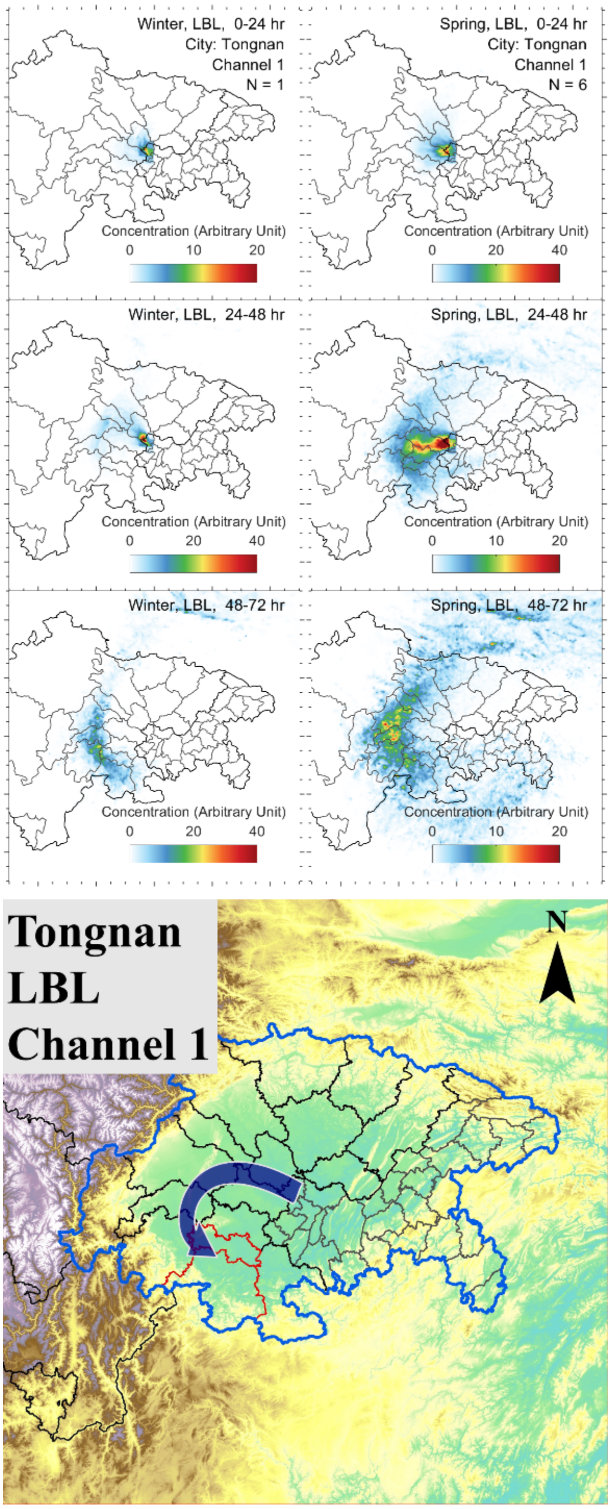

Figure S73 Channel 1 of Tongnan at LBL.

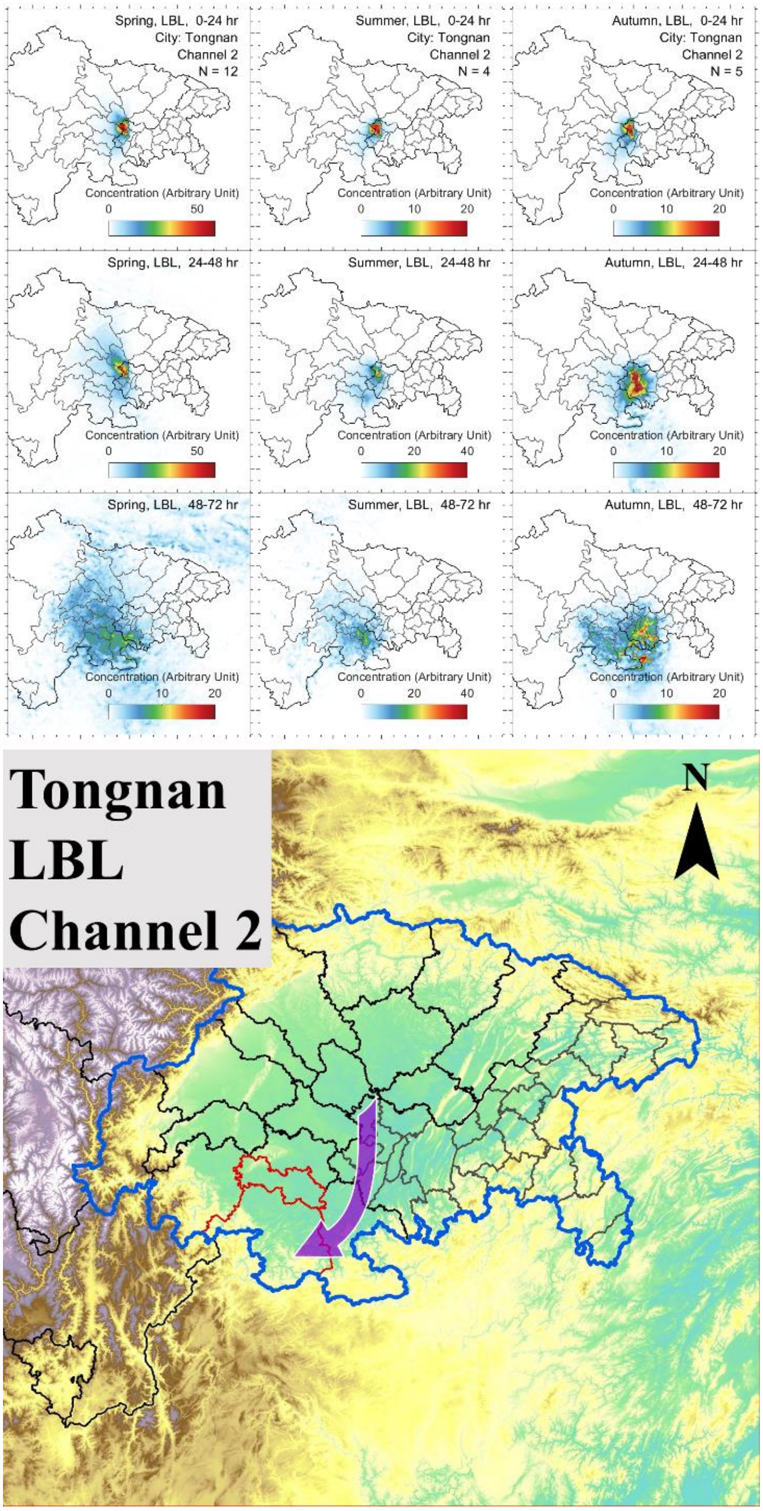

Figure S74 Channel 2 of Tongnan at LBL.

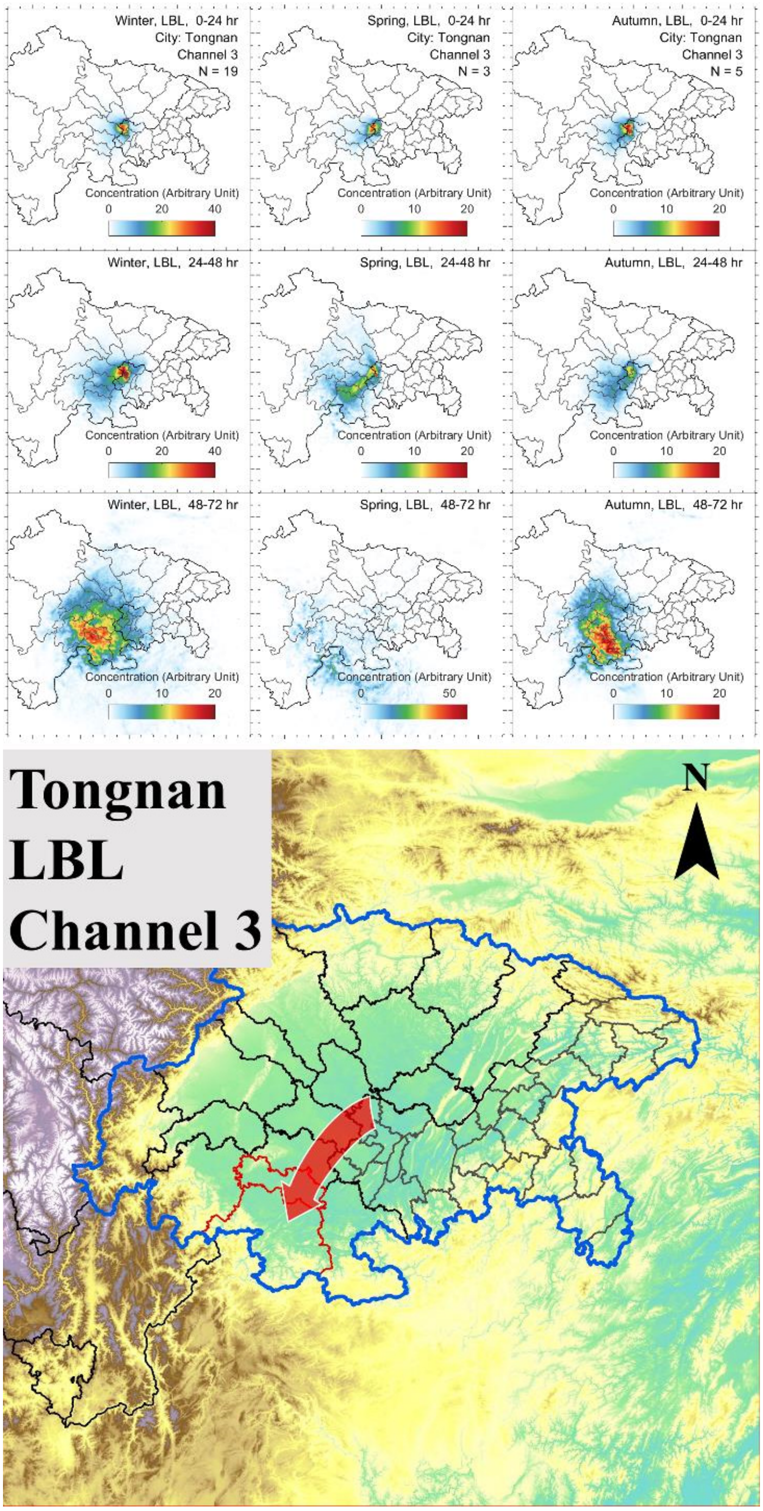

Figure S75 Channel 3 of Tongnan at LBL.

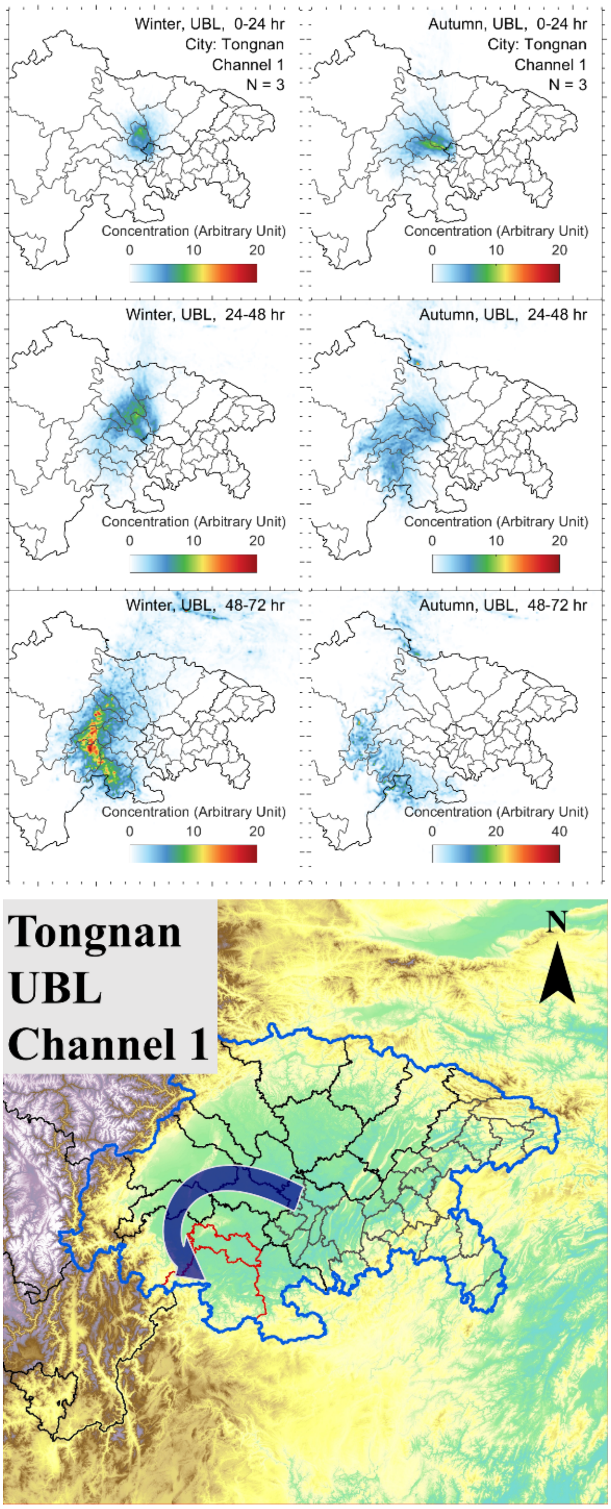

Figure S76 Channel 1 of Tongnan at UBL.

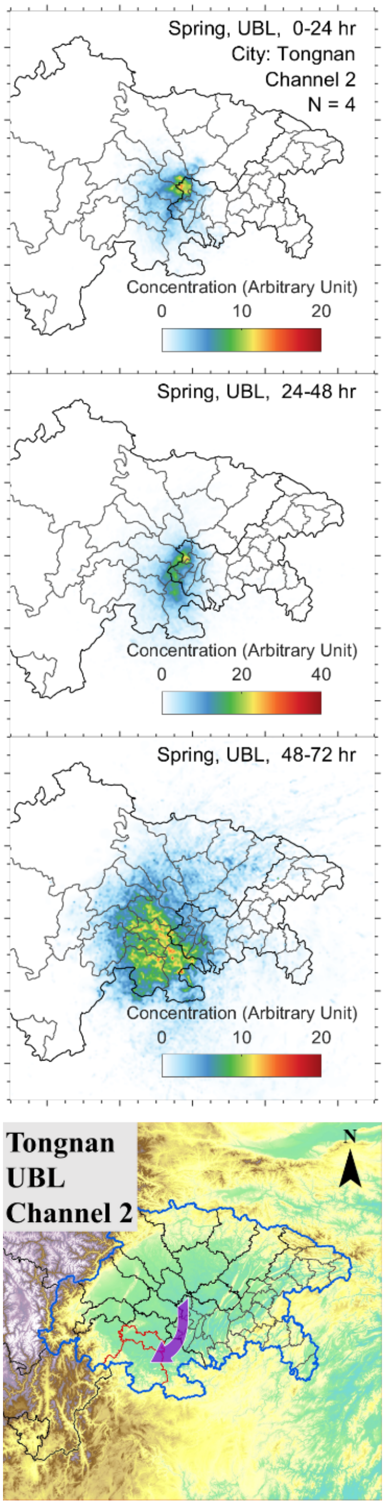

Figure S77 Channel 2 of Tongnan at UBL.

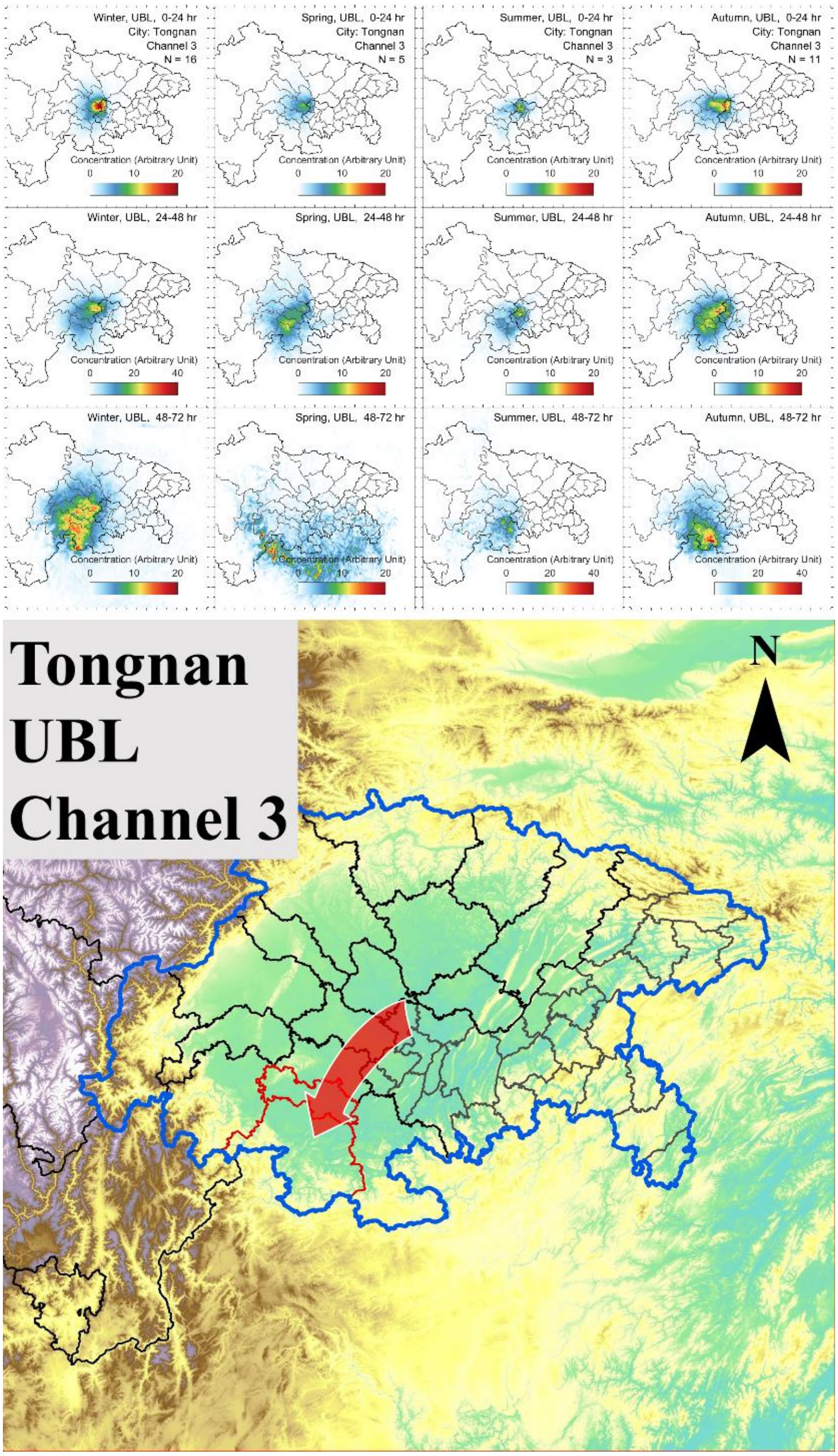

Figure S78 Channel 3 of Tongnan at UBL.

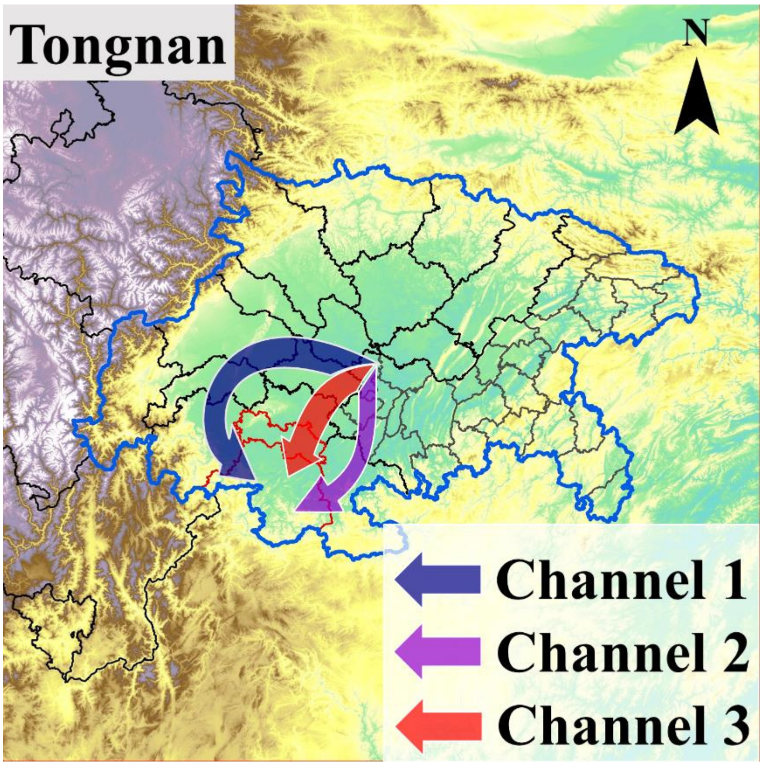

Figure S79 The identified 3 channels originating in Tongnan.

Table S16 Occurrence frequencies of each channel originating in Tongnan in four seasons.

City	Channel	Layer	Season			
			autumn	spring	summer	winter
Tongnan	1	LBL		20.0%		3.2%
		UBL	9.7%			9.7%
	2	LBL	16.1%	40.0%	12.9%	
		UBL		13.3%		
	3	LBL	16.1%	10.0%		61.3%
		UBL	35.5%	16.7%	9.7%	51.6%

4.3 Tongliang

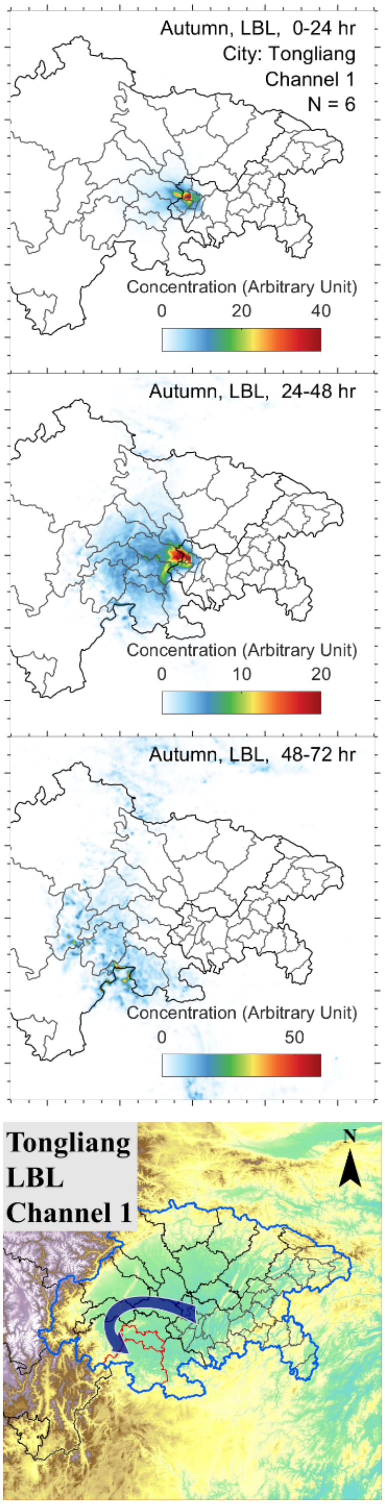

Figure S80 Channel 1 of Tongliang at LBL.

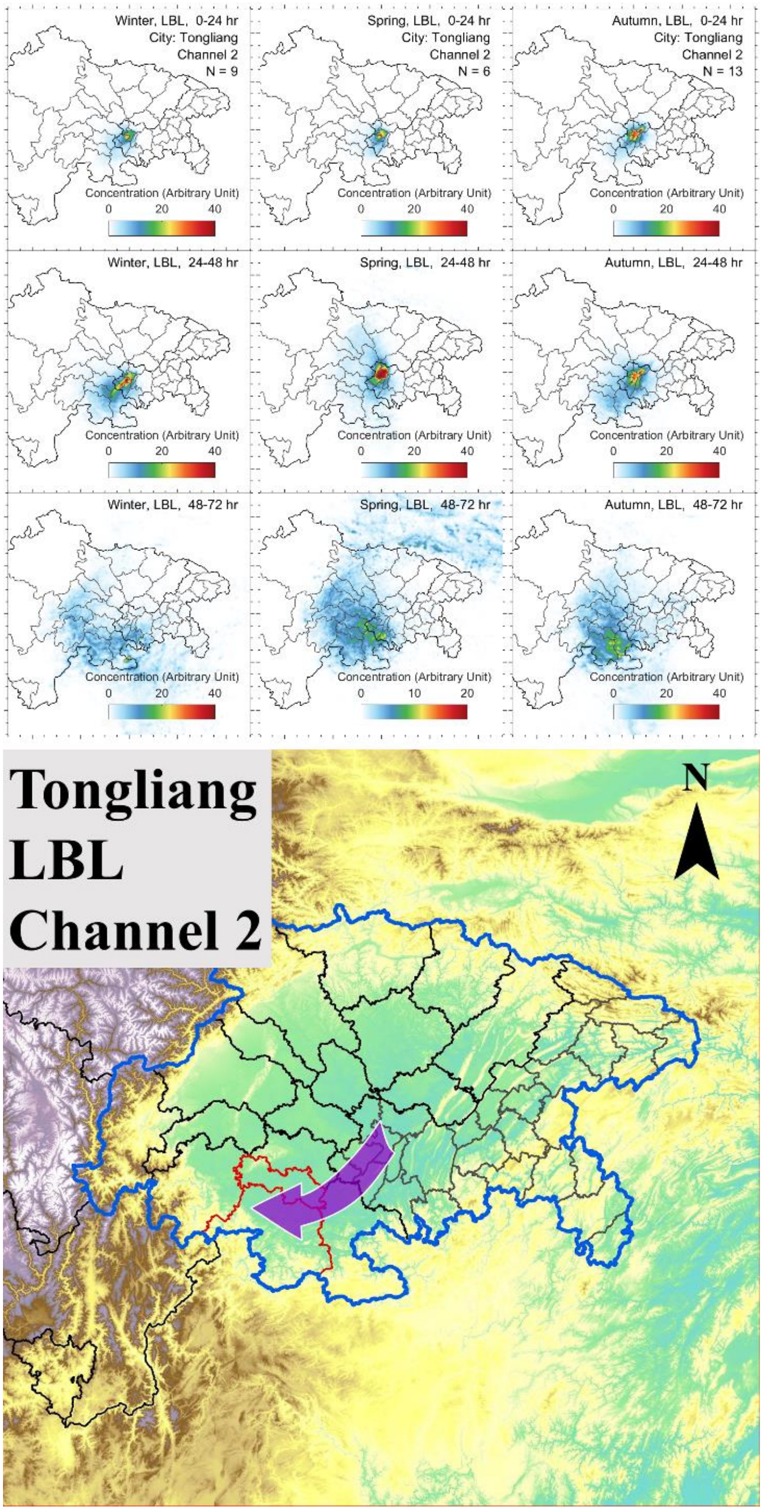

Figure S81 Channel 2 of Tongliang at LBL.

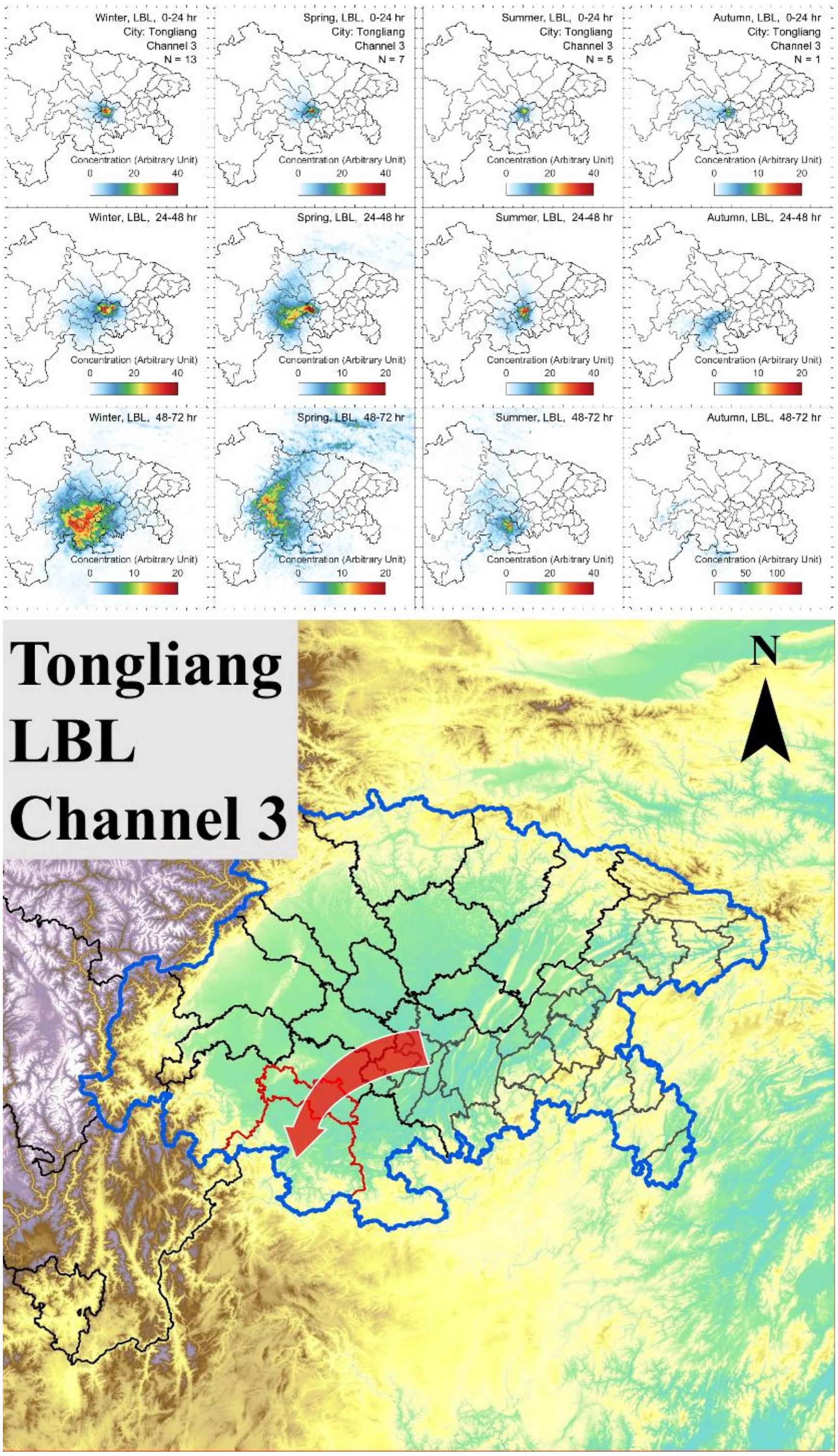

Figure S82 Channel 3 of Tongliang at LBL.

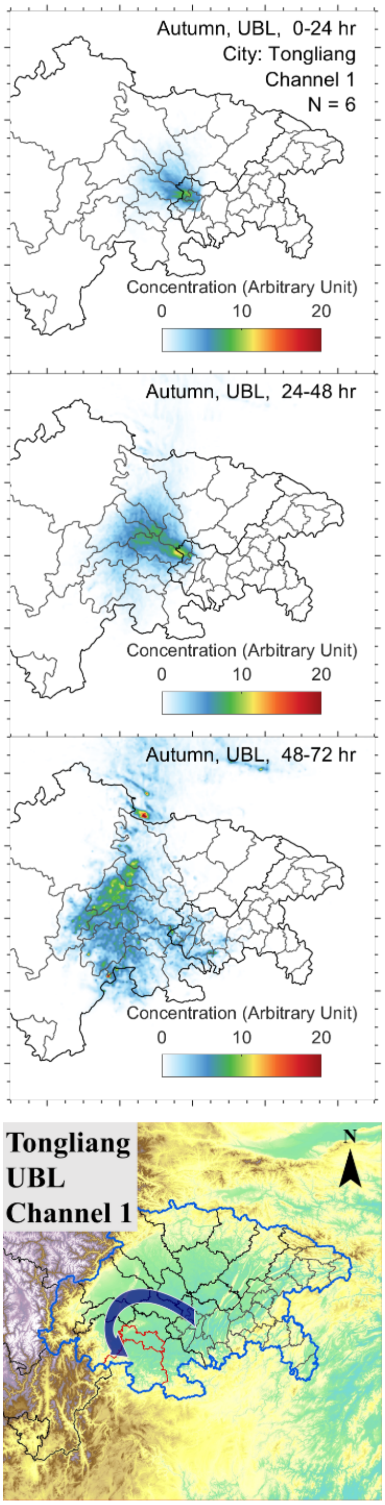

Figure S83 Channel 1 of Tongliang at UBL.

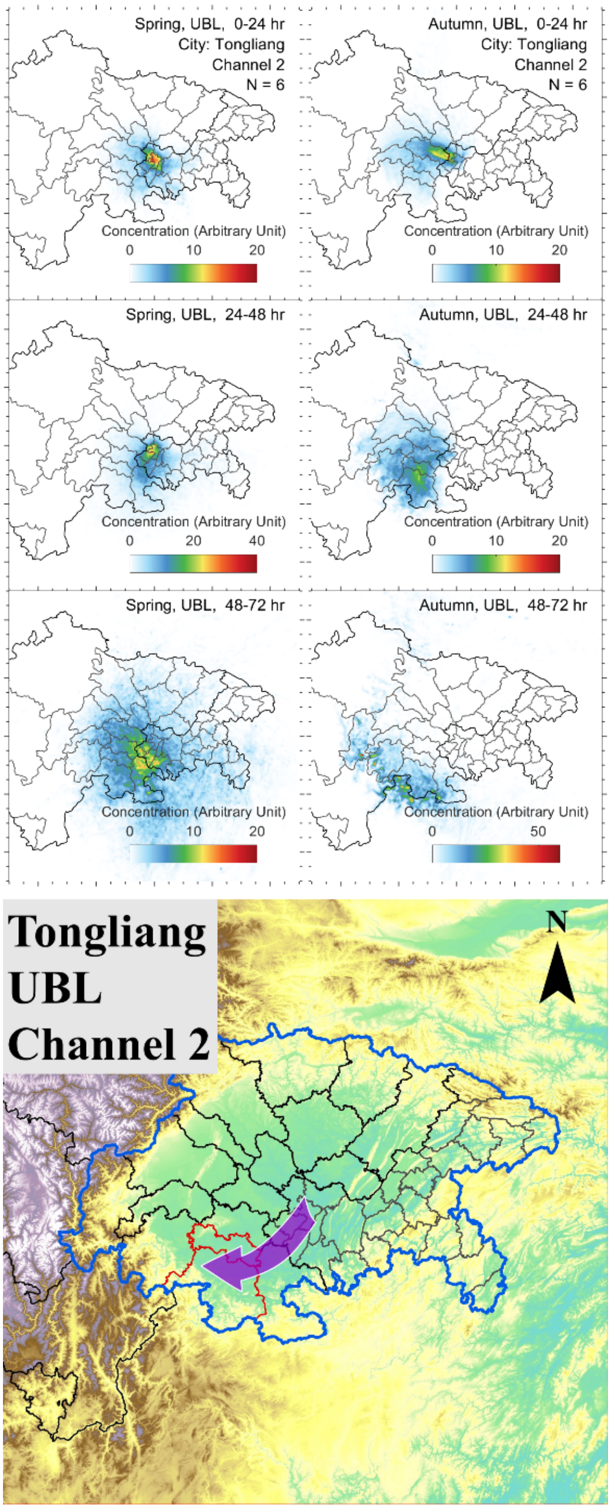

Figure S84 Channel 2 of Tongliang at UBL.

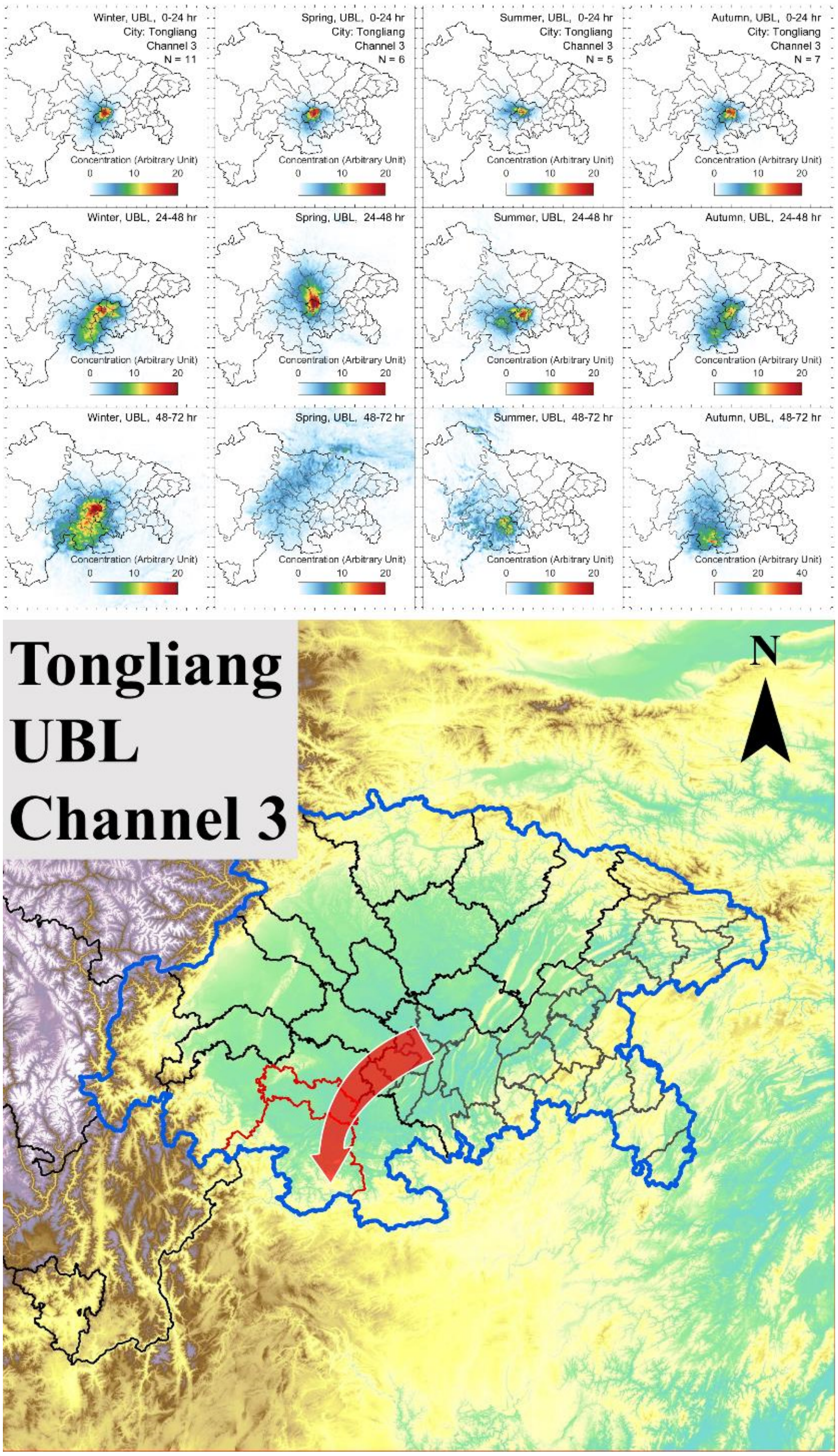

Figure S85 Channel 3 of Tongliang at UBL.

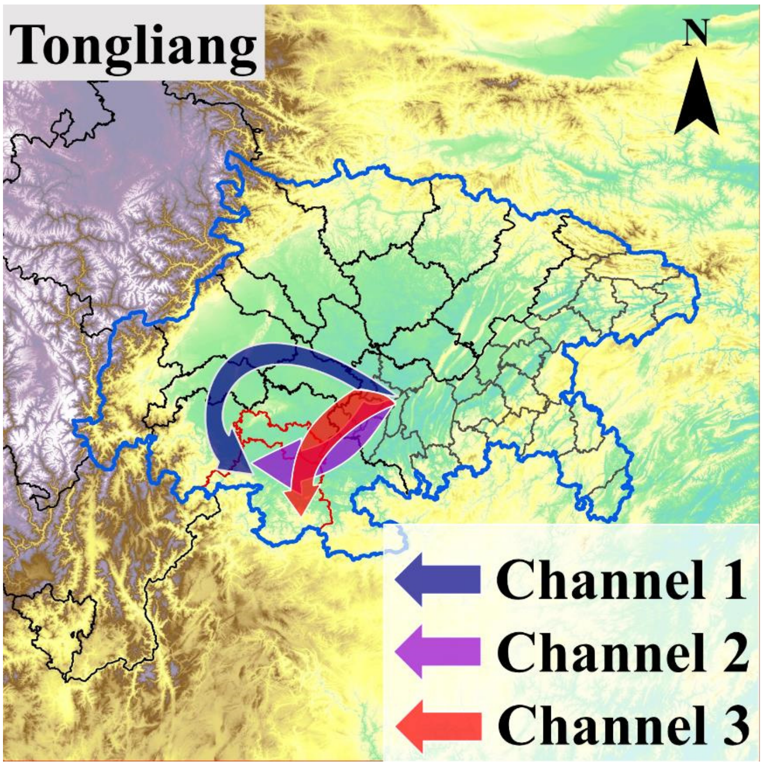

Figure S86 The identified 3 channels originating in Tongliang.

Table S17 Occurrence frequencies of each channel originating in Tongliang in four seasons.

City	Channel	Layer	Season			
			autumn	spring	summer	winter
Tongliang	1	LBL	19.4%			
		UBL	19.4%			
	2	LBL	41.9%	20.0%		29.0%
		UBL	19.4%	20.0%		
	3	LBL	3.2%	23.3%	16.1%	41.9%
		UBL	22.6%	20.0%	16.1%	35.5%

4.4 Dazu

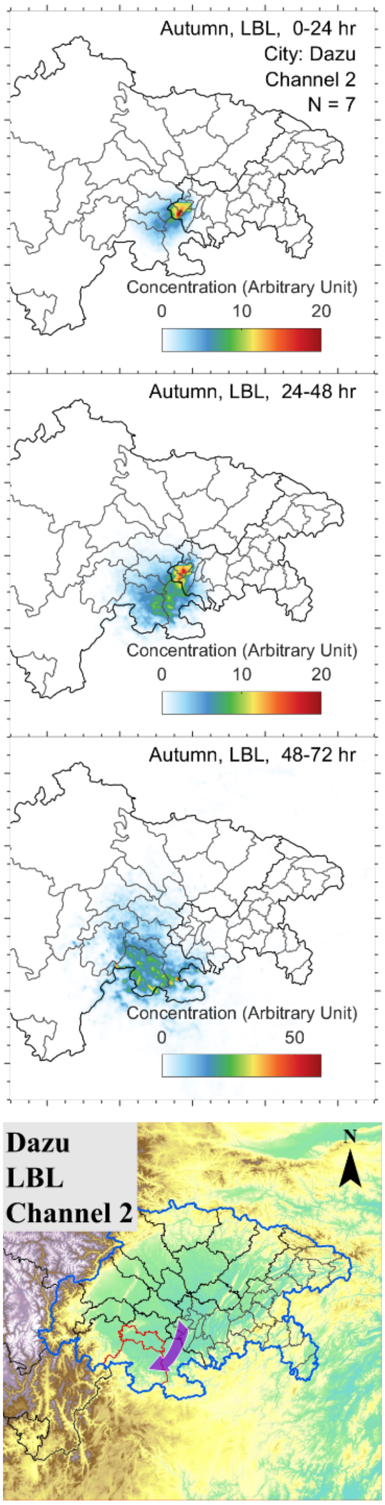

Figure S87 Channel 2 of Dazu at LBL.

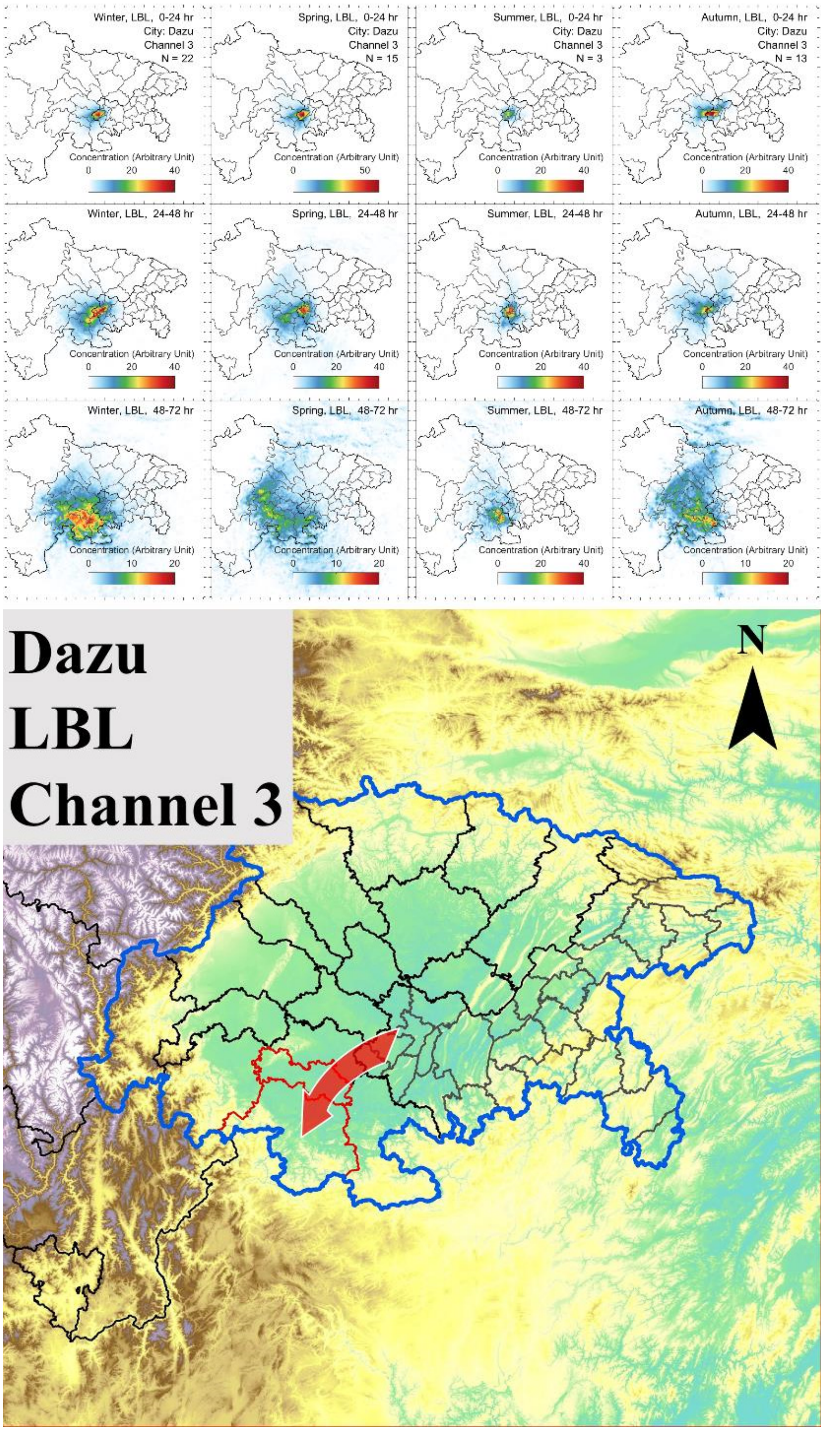

Figure S88 Channel 3 of Dazu at LBL.

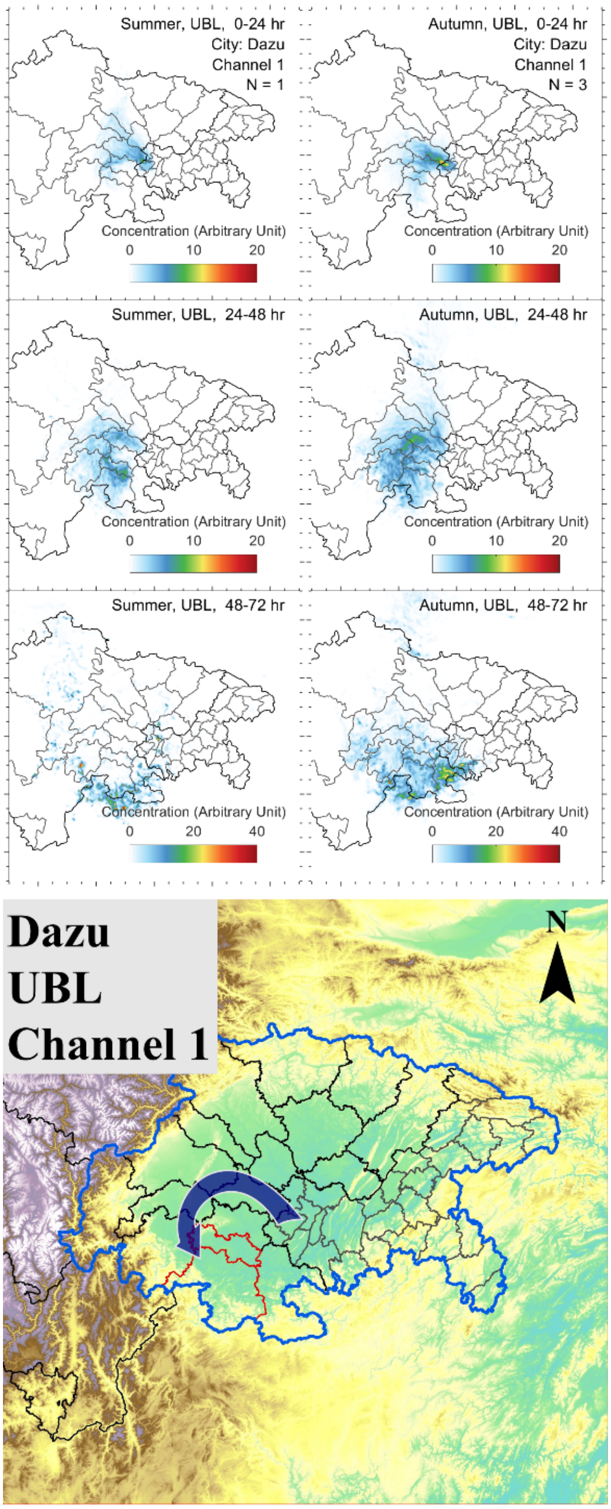

Figure S89 Channel 1 of Dazu at UBL.

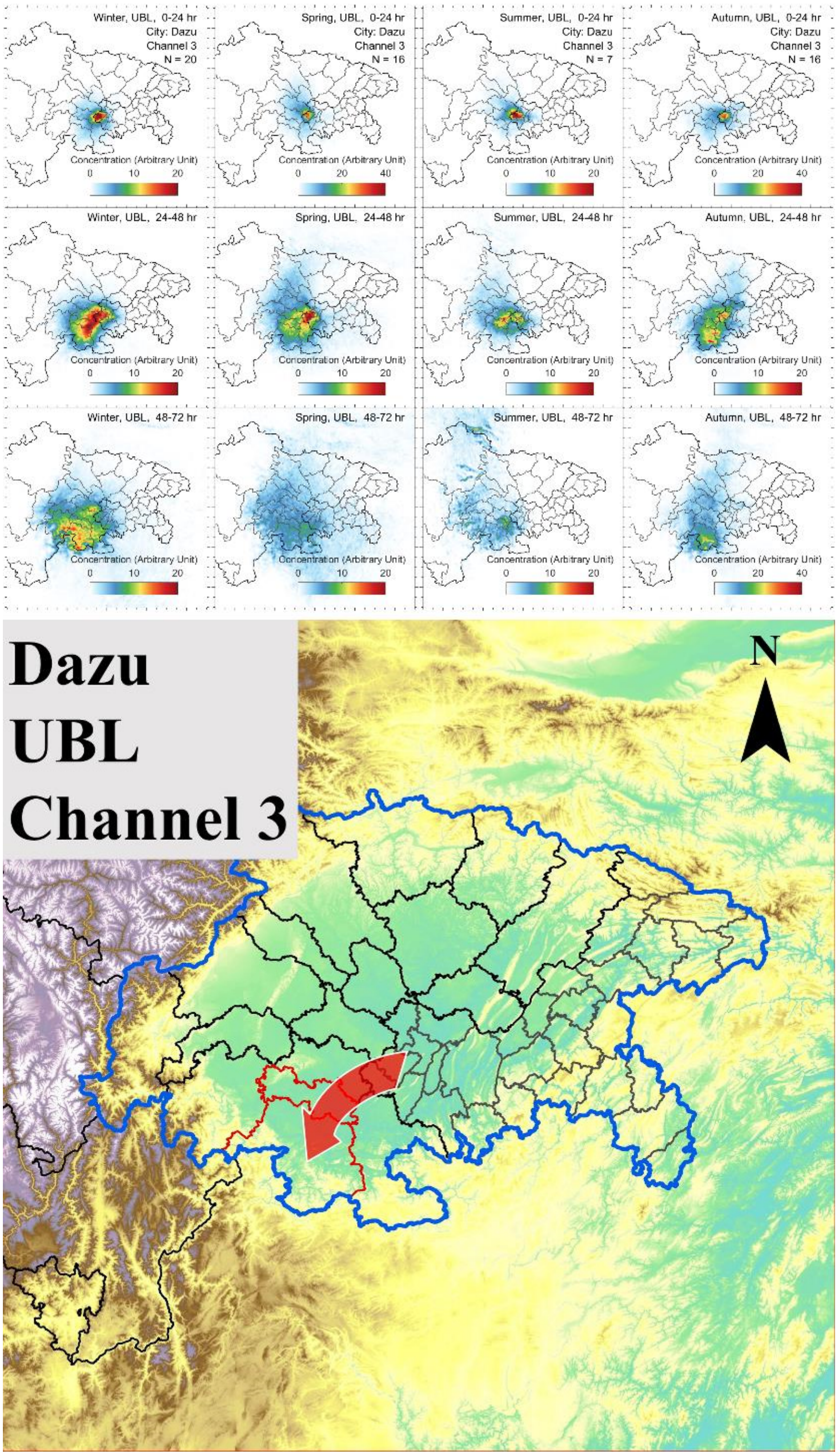

Figure S90 Channel 3 of Dazu at UBL.

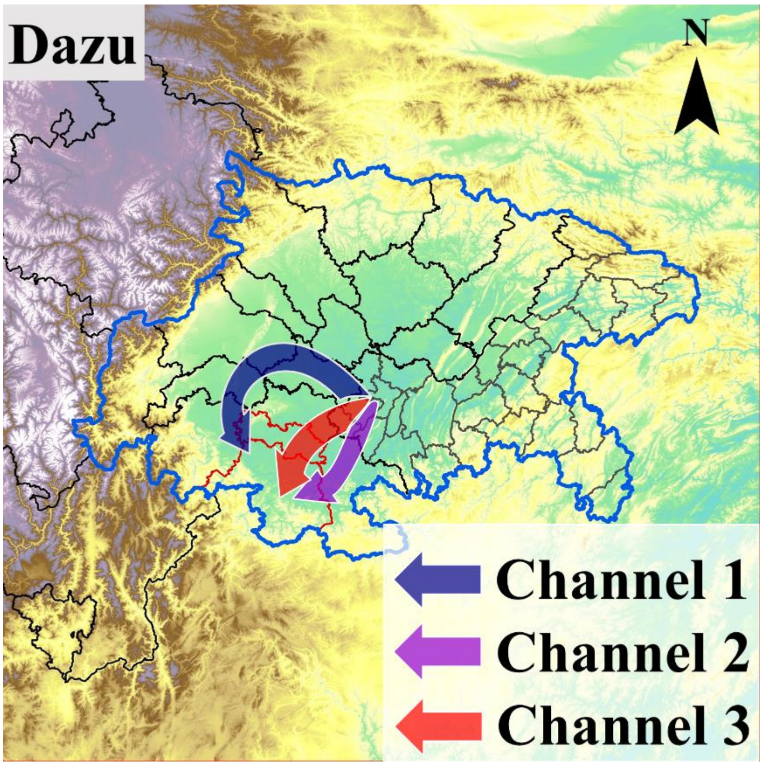

Figure S91 The identified 2 channels originating in Dazu.

Table S18 Occurrence frequencies of each channel originating in Dazu in four seasons.

City	Channel	Layer	Season			
			autumn	spring	summer	winter
Dazu	1	UBL	9.7%		3.2%	
	2	LBL	22.6%			
	3	LBL	41.9%	50.0%	9.7%	71.0%
		UBL	51.6%	53.3%	22.6%	64.5%

4.5 Rongchang

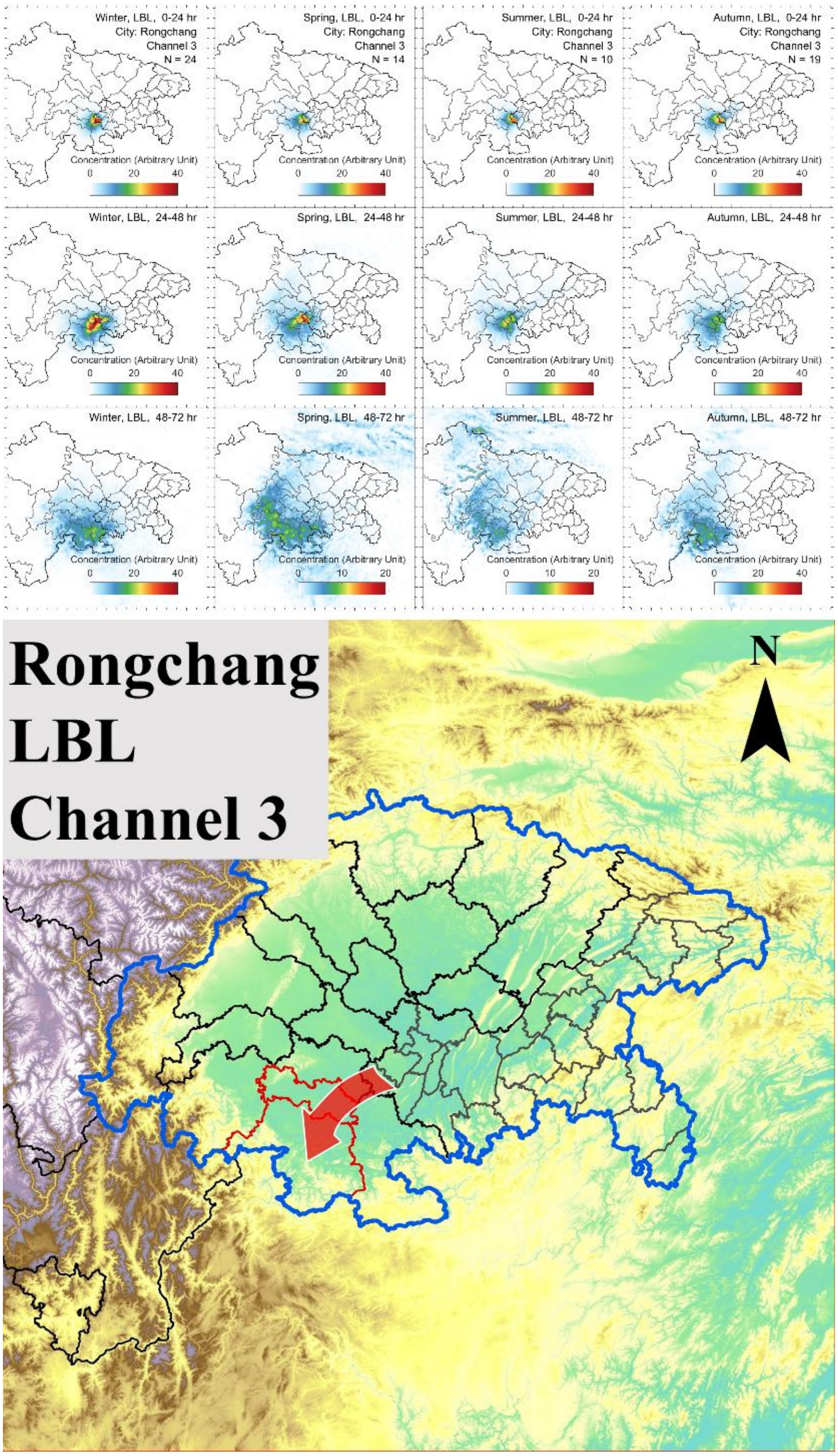

Figure S92 Channel 3 of Rongchang at LBL.

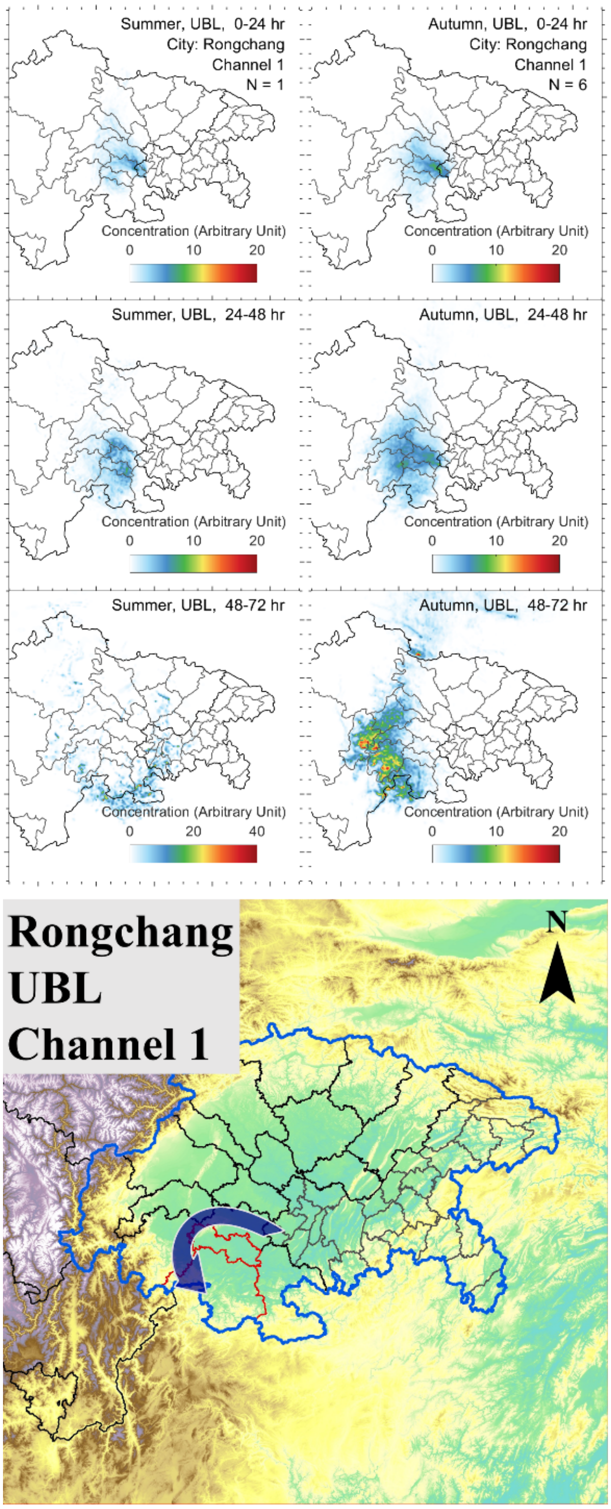

Figure S93 Channel 1 of Rongchang at UBL.

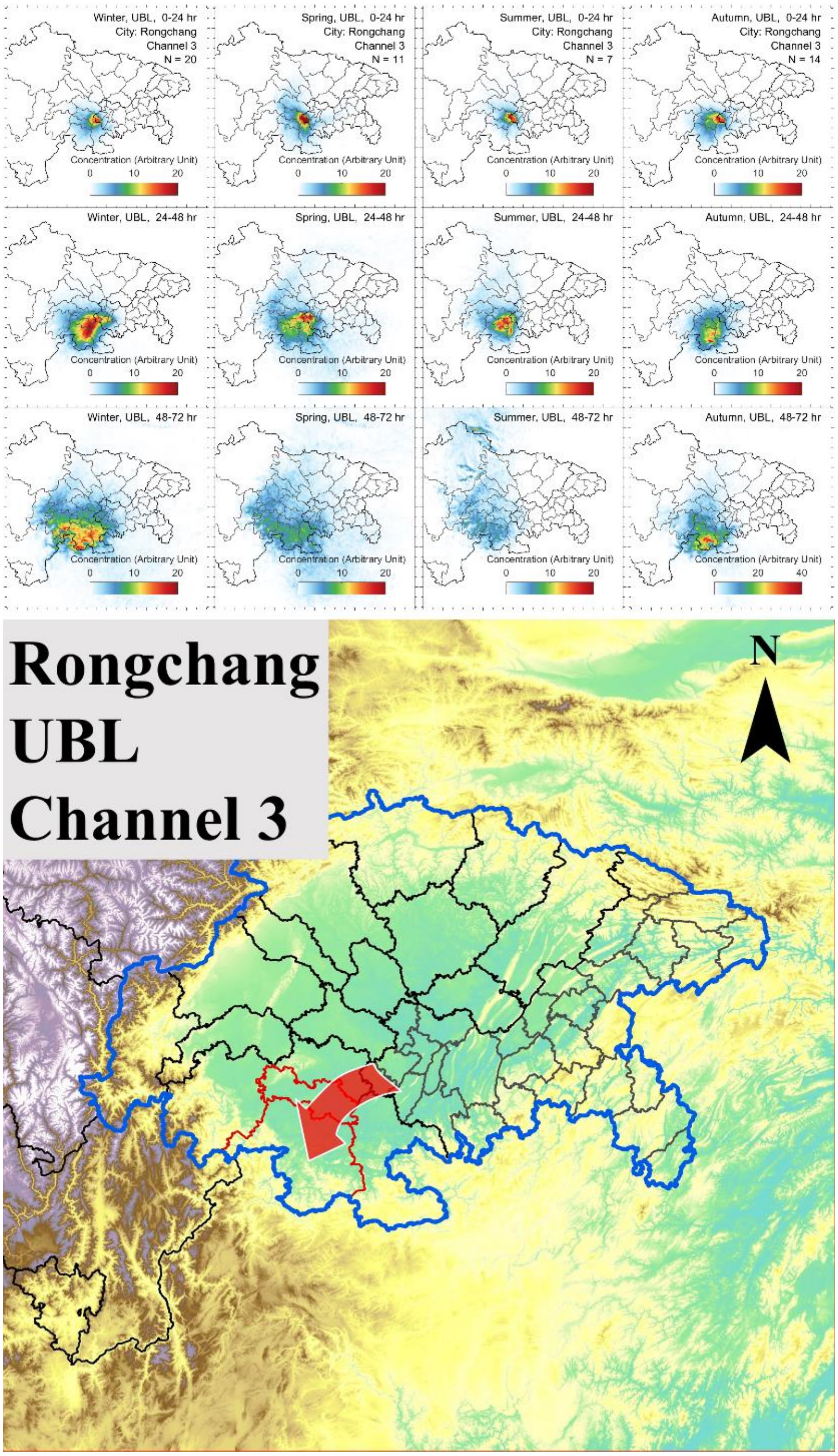

`

Figure S94 Channel 3 of Rongchang at UBL.

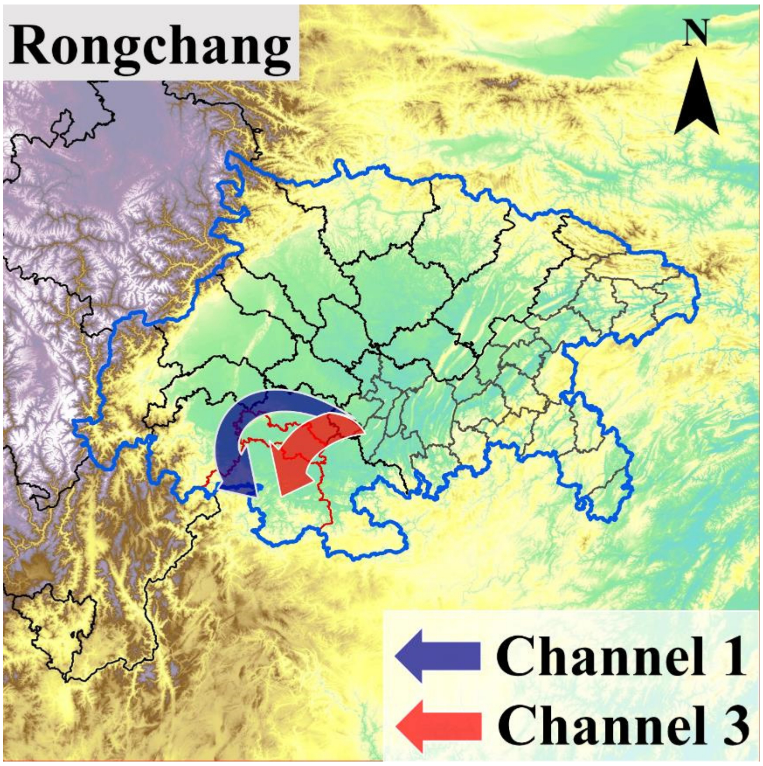

Figure S95 The identified 2 channels originating in Rongchang.

Table S19 Occurrence frequencies of each channel originating in Rongchang in four seasons.

City	Channel	Layer	Season			
			autumn	spring	summer	winter
Rongchang	1	UBL	19.4%		3.2%	
	3	LBL	61.3%	46.7%	32.3%	77.4%
		UBL	45.2%	36.7%	22.6%	64.5%

5. Southwest Chongqing Urban Agglomeration

5.1 Bishan

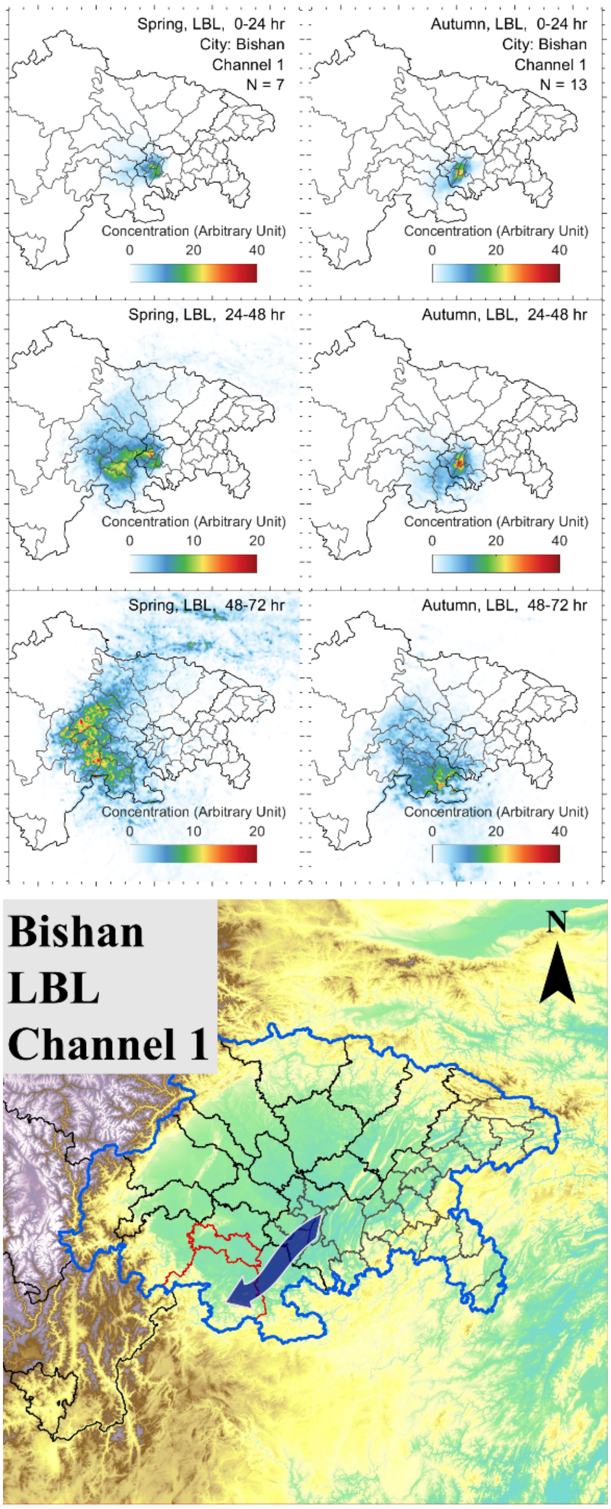

Figure S96 Channel 1 of Bishan at LBL.

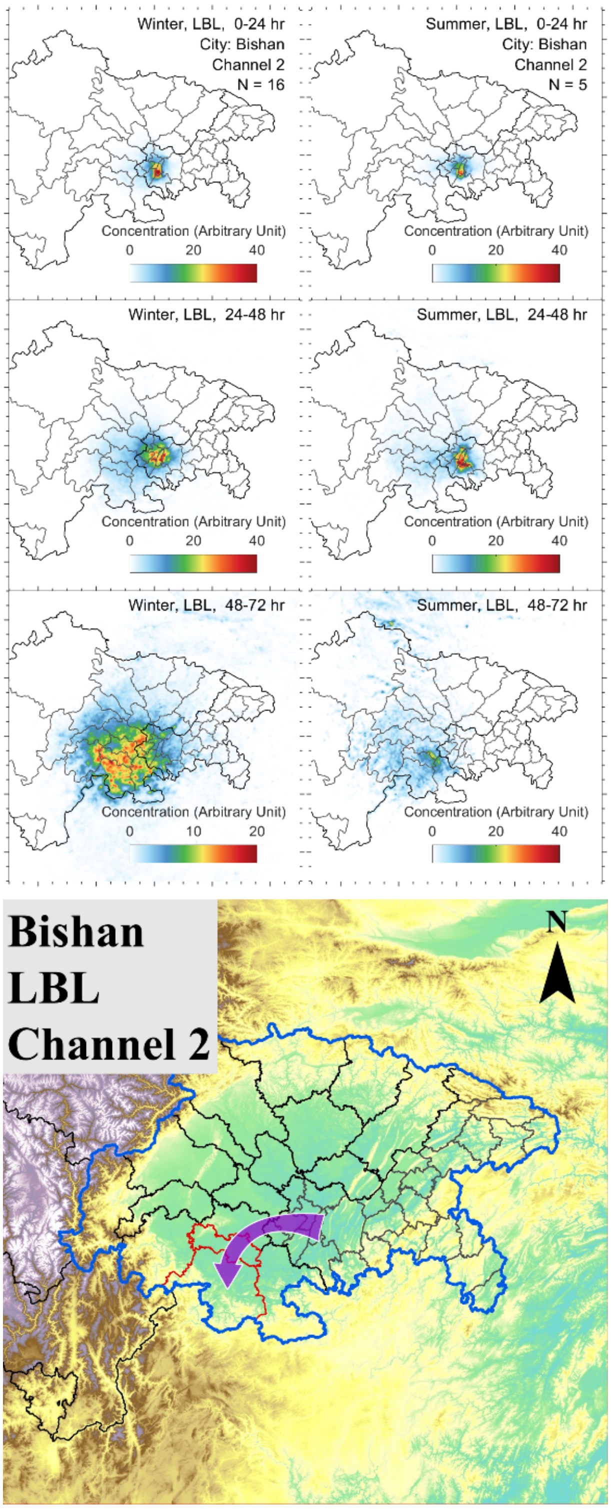

Figure S97 Channel 2 of Bishan at LBL.

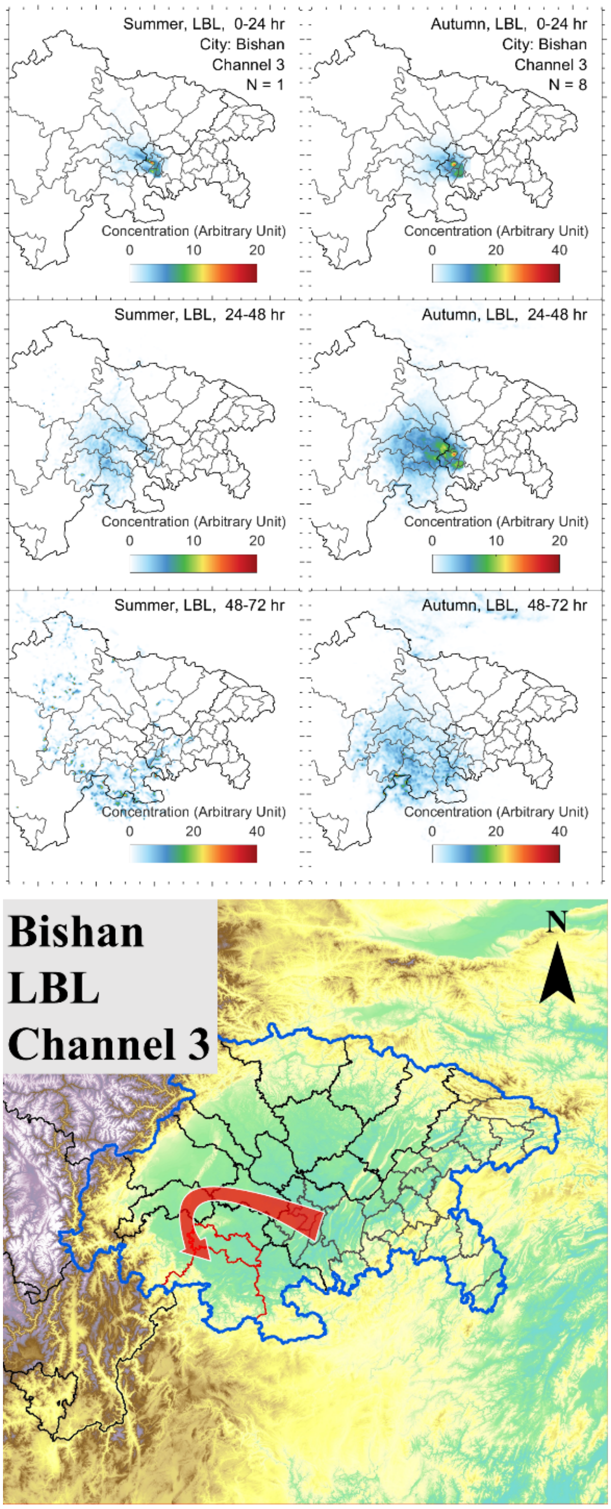

Figure S98 Channel 3 of Bishan at LBL.

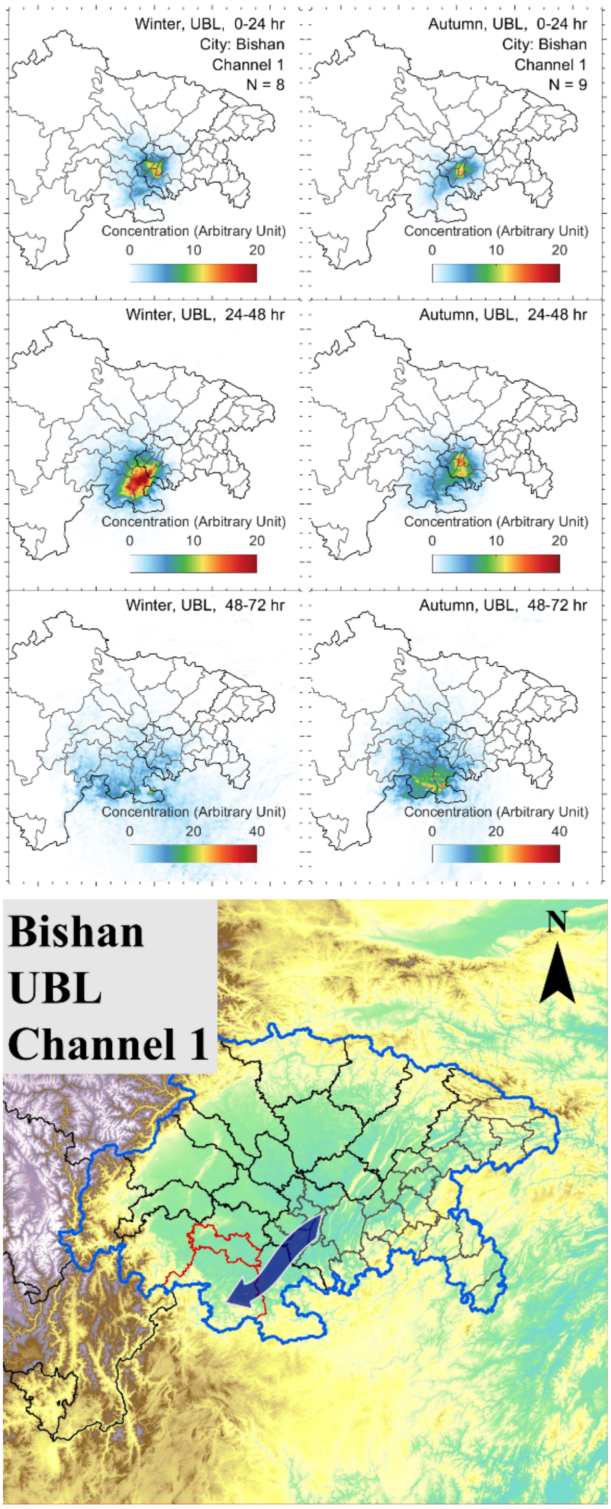

Figure S99 Channel 1 of Bishan at UBL.

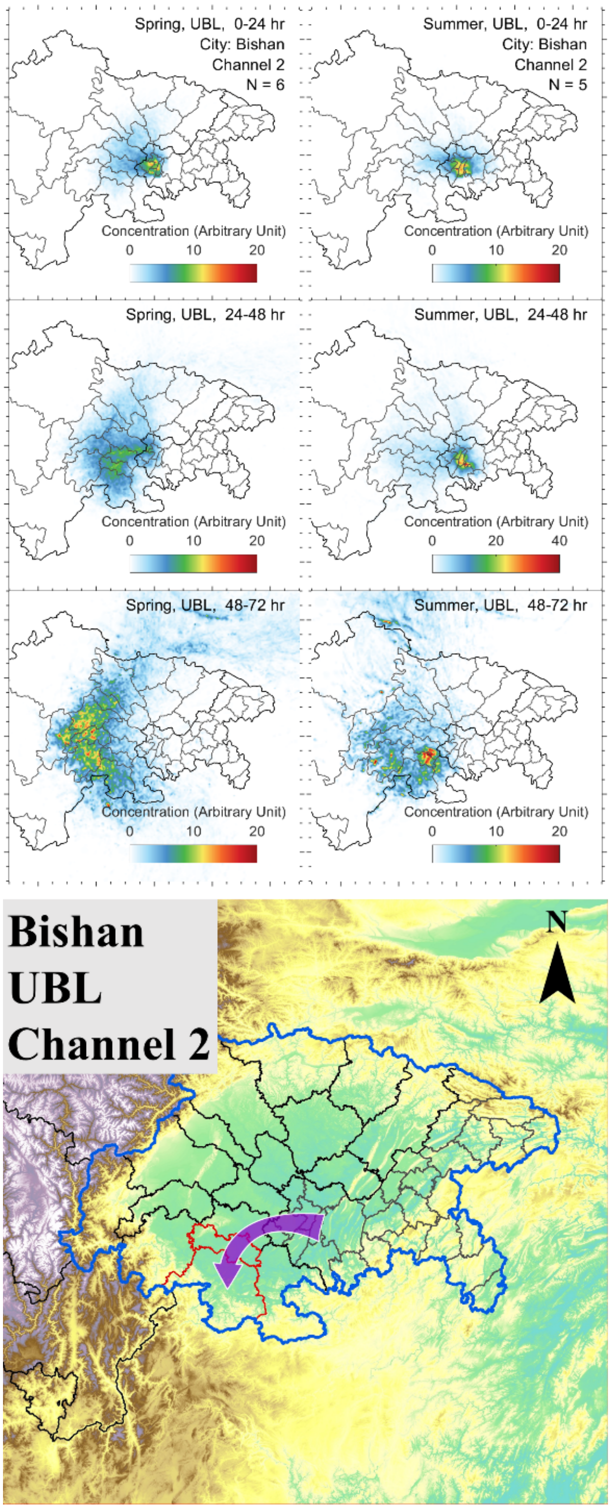

Figure S100 Channel 2 of Bishan at UBL.

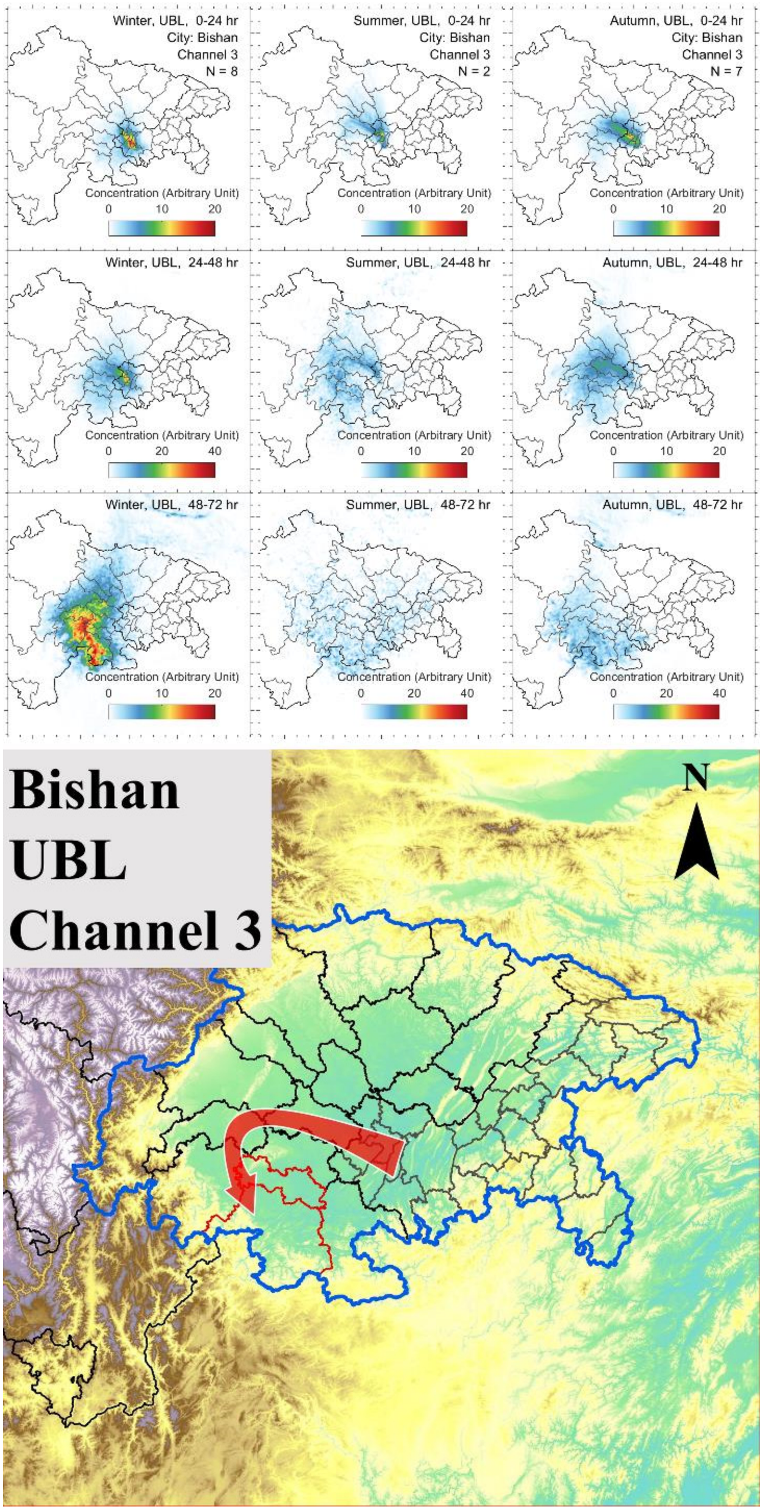

Figure S101 Channel 3 of Bishan at UBL.

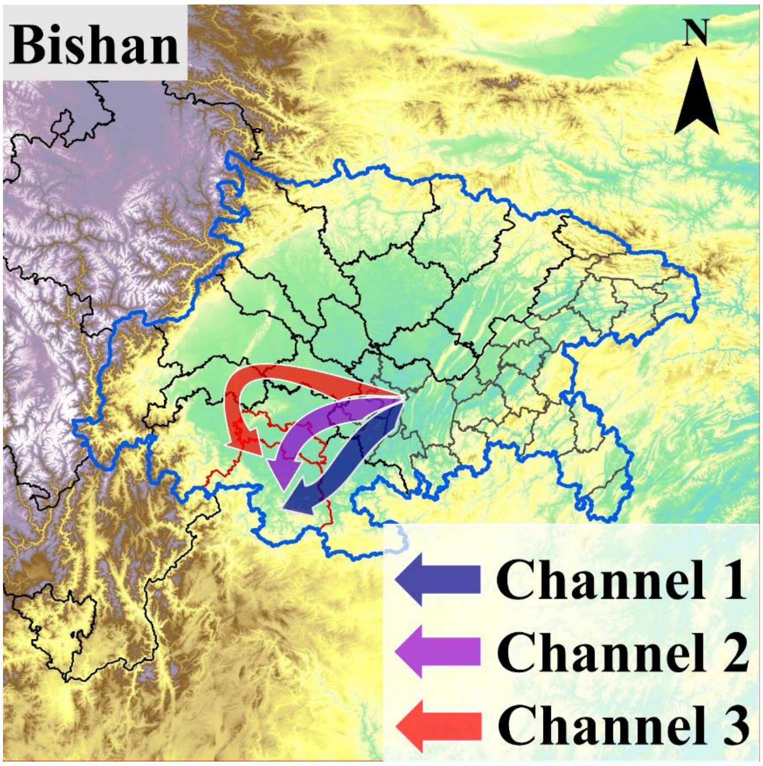

Figure S102 The identified 3 channels originating in Bishan.

Table 20 Occurrence frequencies of each channel originating in Bishan in four seasons.

City	Channel	Layer	Season			
			autumn	spring	summer	winter
Bishan	1	LBL	41.9%	23.3%		
		UBL	29.0%			25.8%
	2	LBL			16.1%	51.6%
		UBL		20.0%	16.1%	
	3	LBL	25.8%		3.2%	
		UBL	22.6%		6.5%	25.8%

5.2 Yongchuan

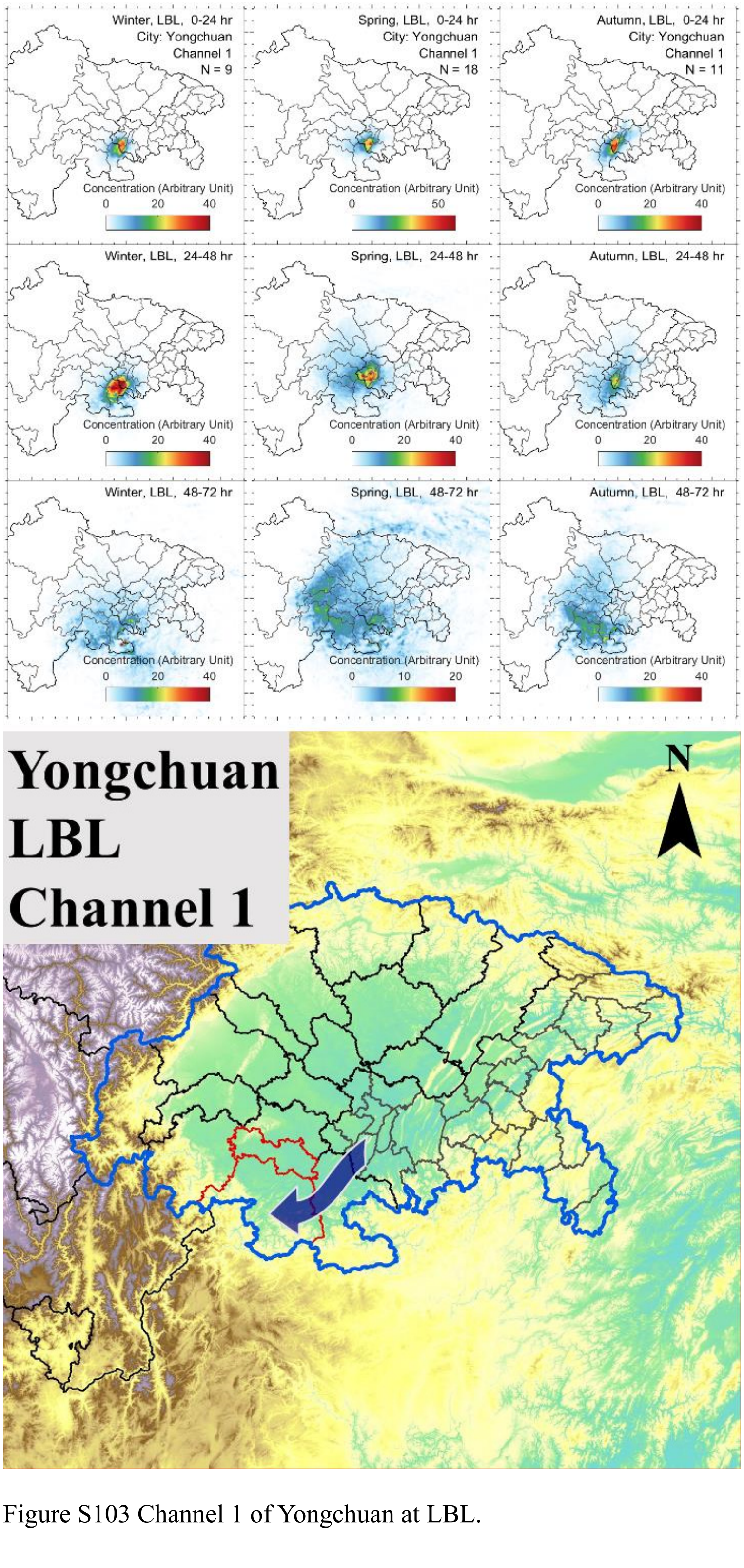

Figure S103 Channel 1 of Yongchuan at LBL.

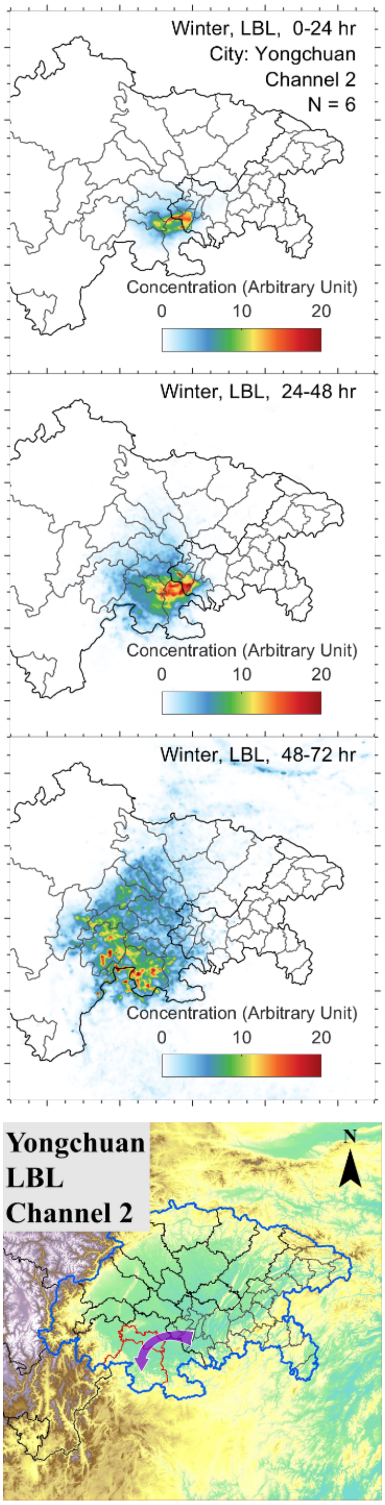

Figure S104 Channel 2 of Yongchuan at LBL.

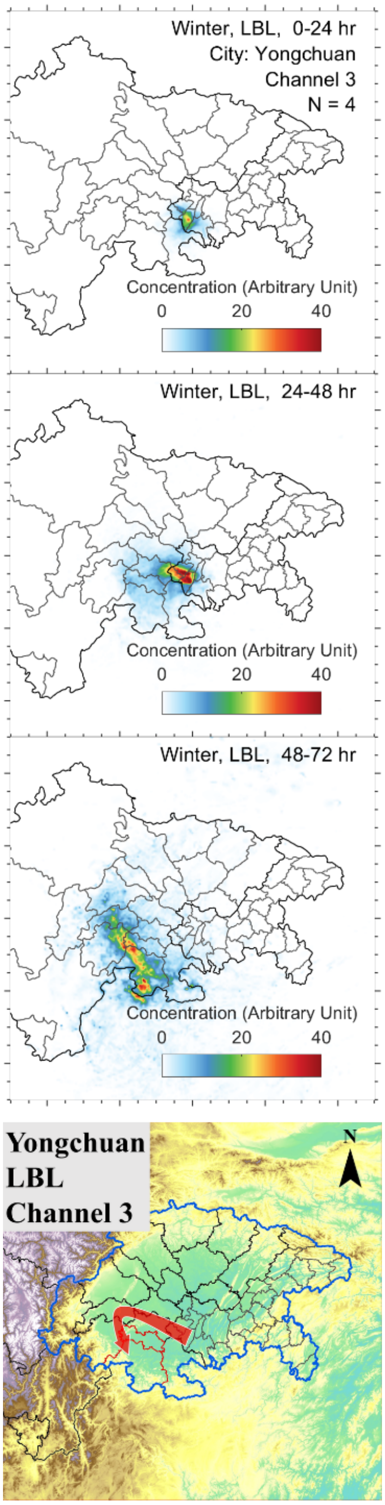

Figure S105 Channel 3 of Yongchuan at LBL.

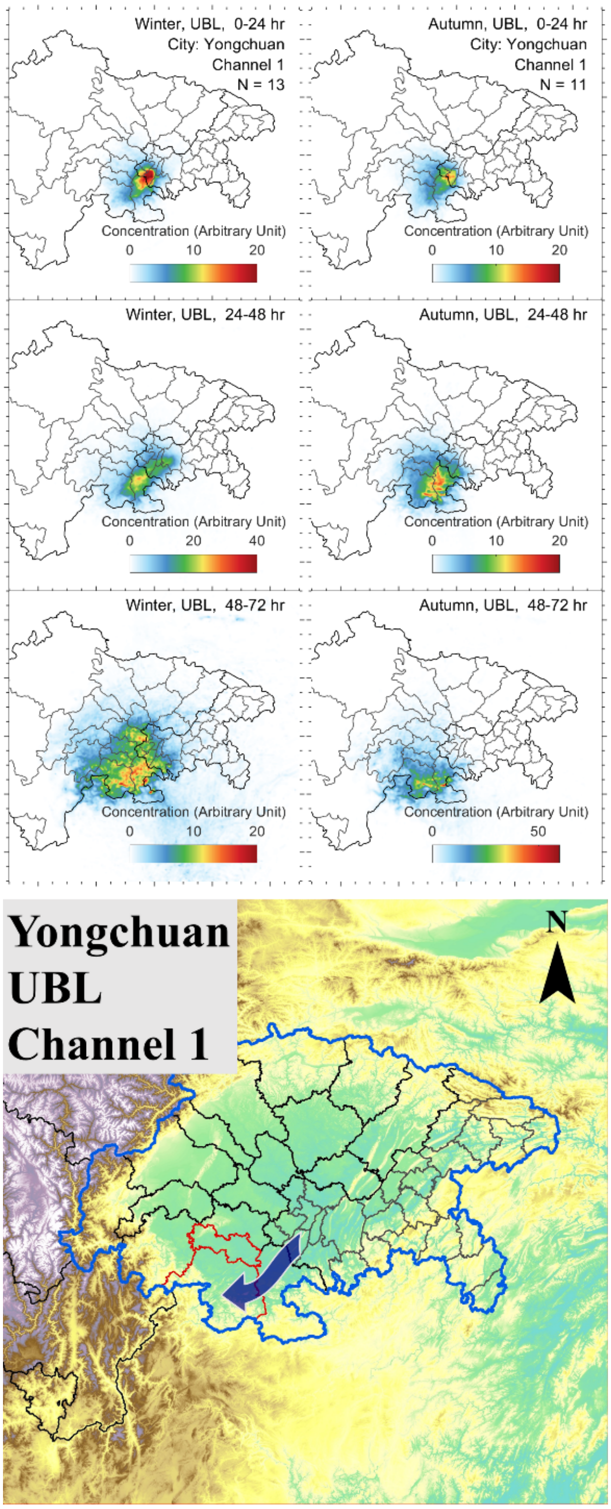

Figure S106 Channel 1 of Yongchuan at UBL.

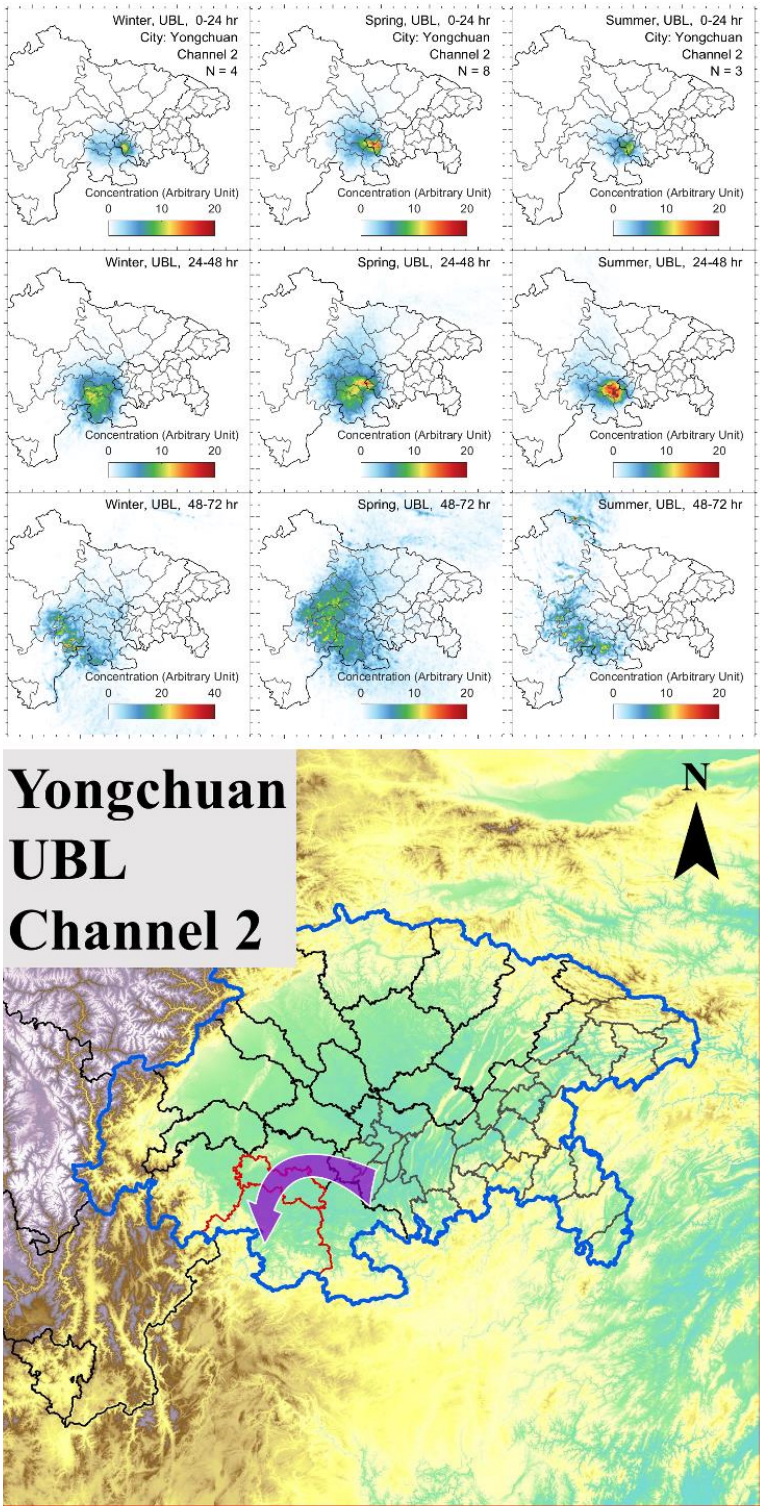

Figure S107 Channel 2 of Yongchuan at UBL.

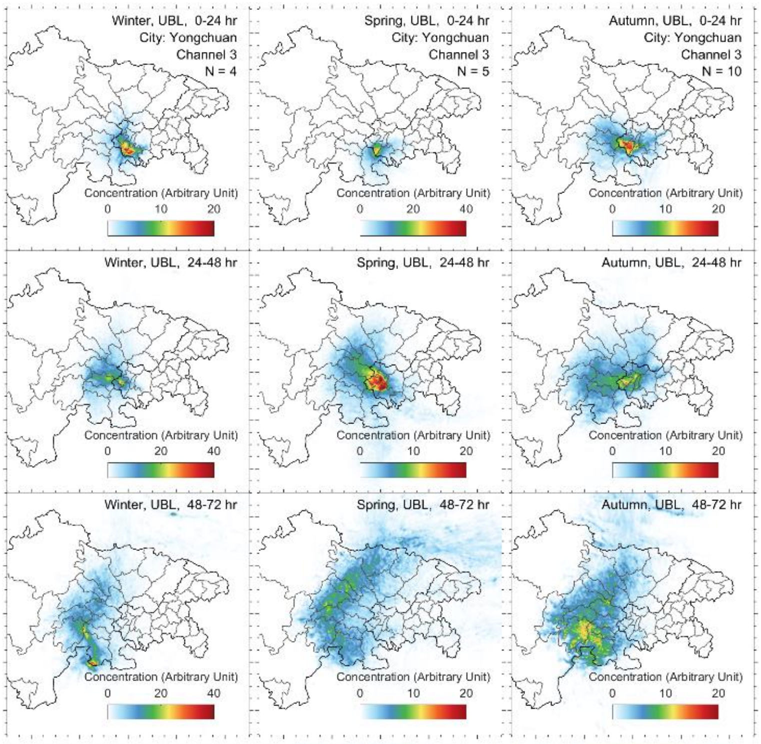

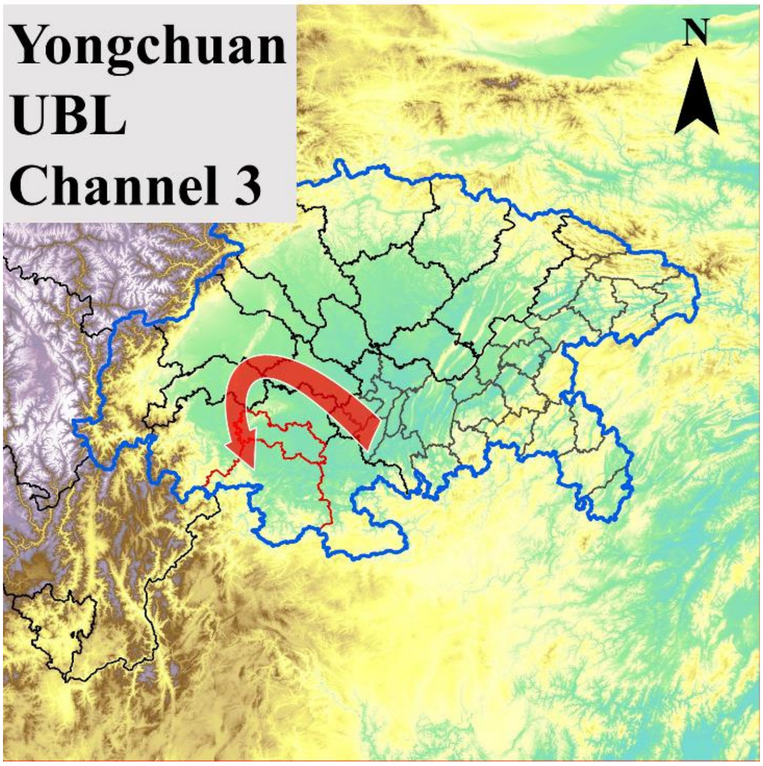

Figure S108 Channel 3 of Yongchuan at UBL.

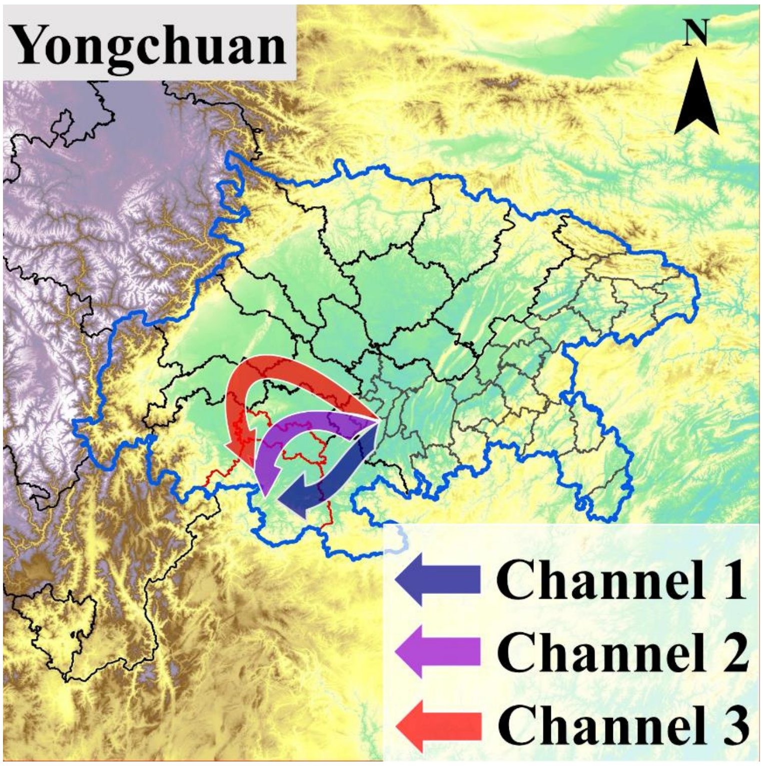

Figure S109 The identified 3 channels originating in Yongchuan.

Table S21 Occurrence frequencies of each channel originating in Yongchuan in four seasons.

City	Channel	Layer	Season			
			autumn	spring	summer	winter
Yongchuan	1	LBL	35.5%	60.0%		29.0%
		UBL	35.5%			41.9%
	2	LBL				19.4%
		UBL		26.7%	9.7%	12.9%
	3	LBL				12.9%
		UBL	32.3%	16.7%		12.9%

5.3 Jiangjin

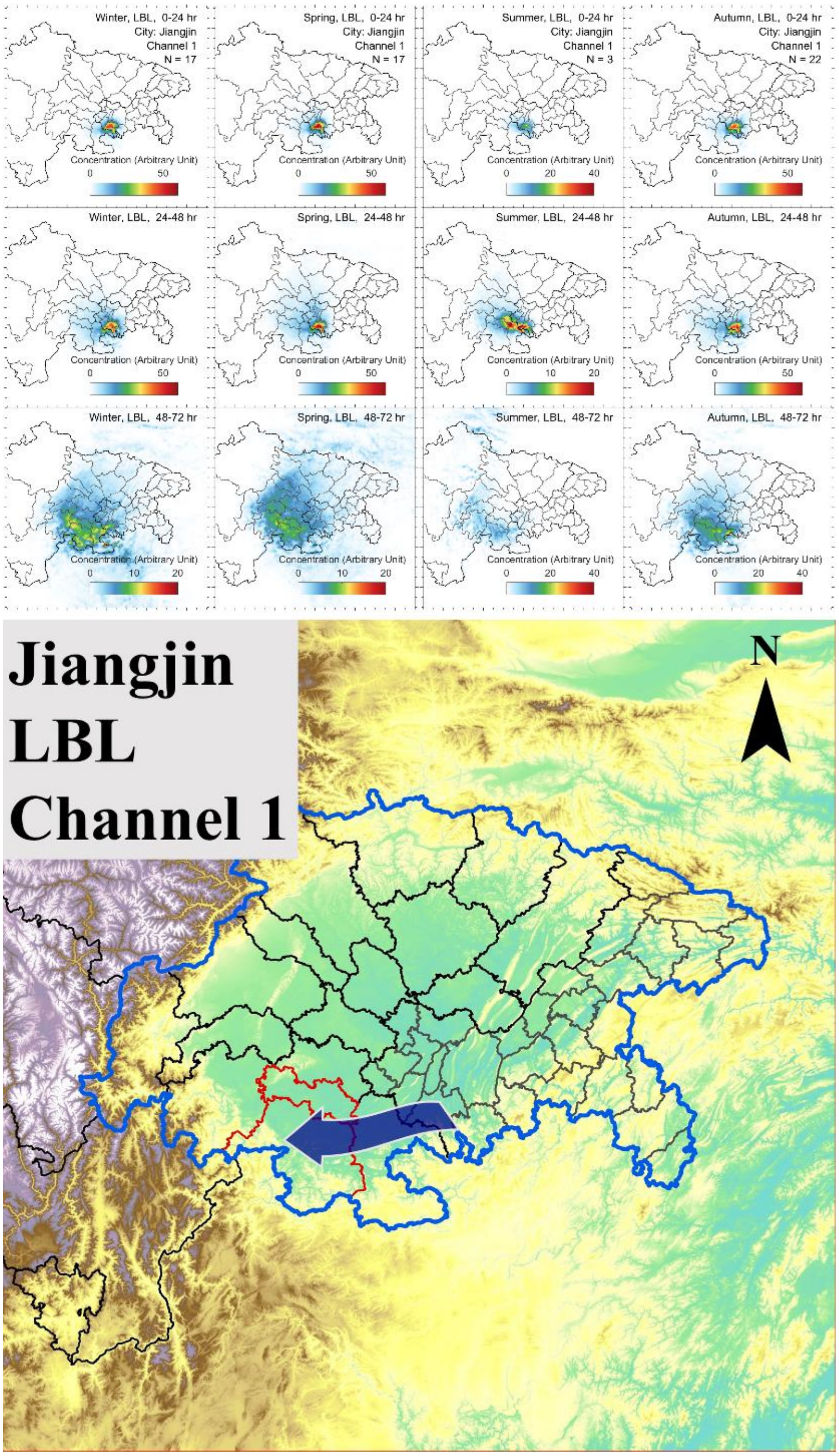

Figure S110 Channel 1 of Jiangjin at LBL.

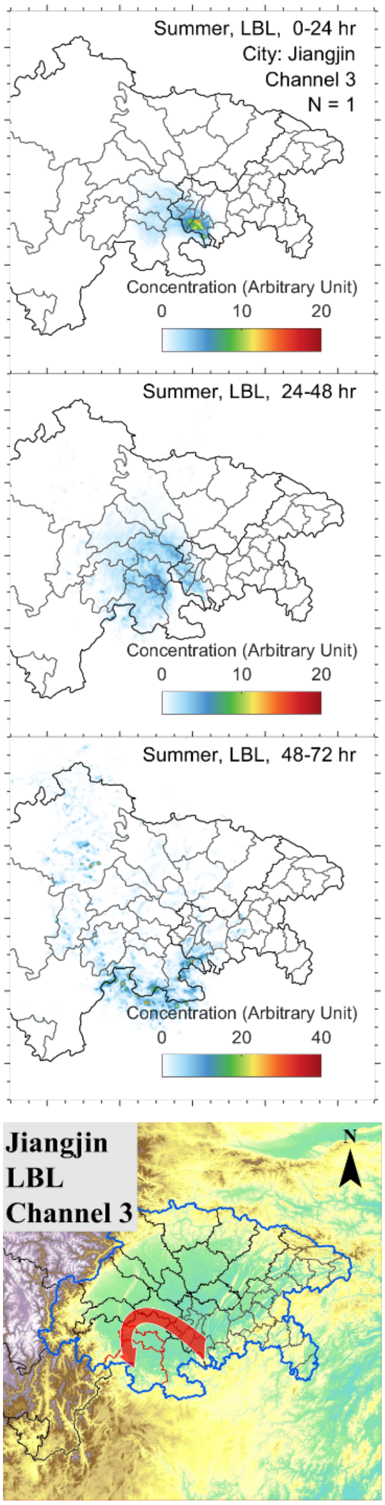

Figure S111 Channel 3 of Jiangjin at LBL.

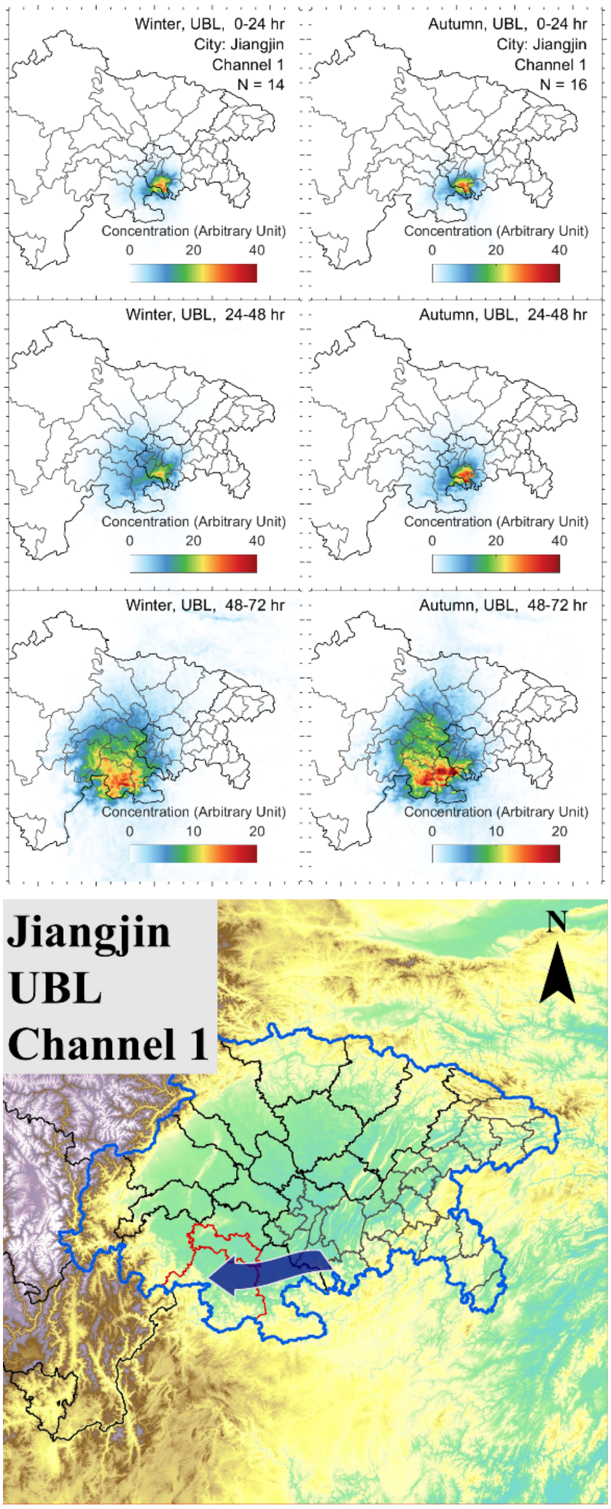

Figure S112 Channel 1 of Jiangjin at UBL.

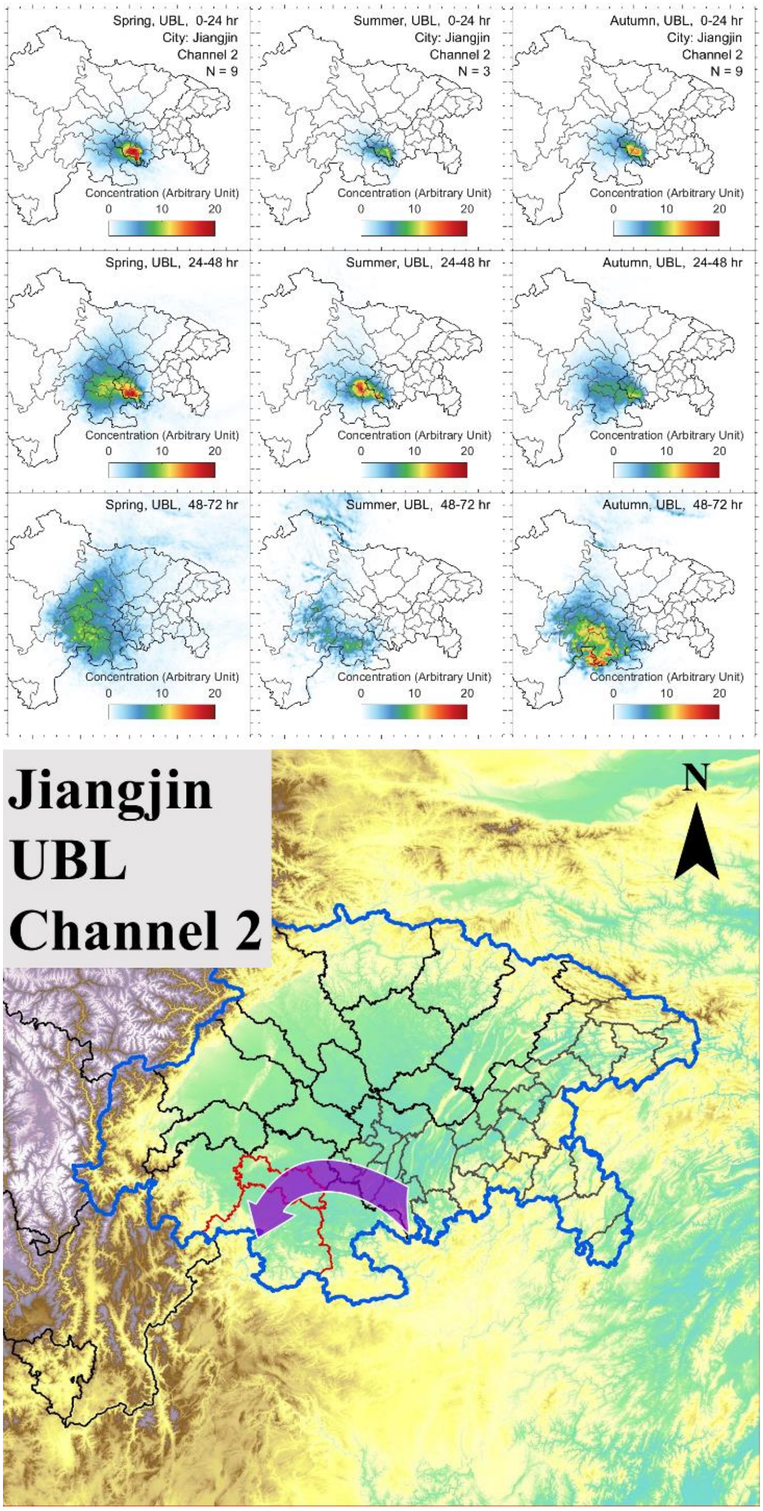

Figure S113 Channel 2 of Jiangjin at UBL.

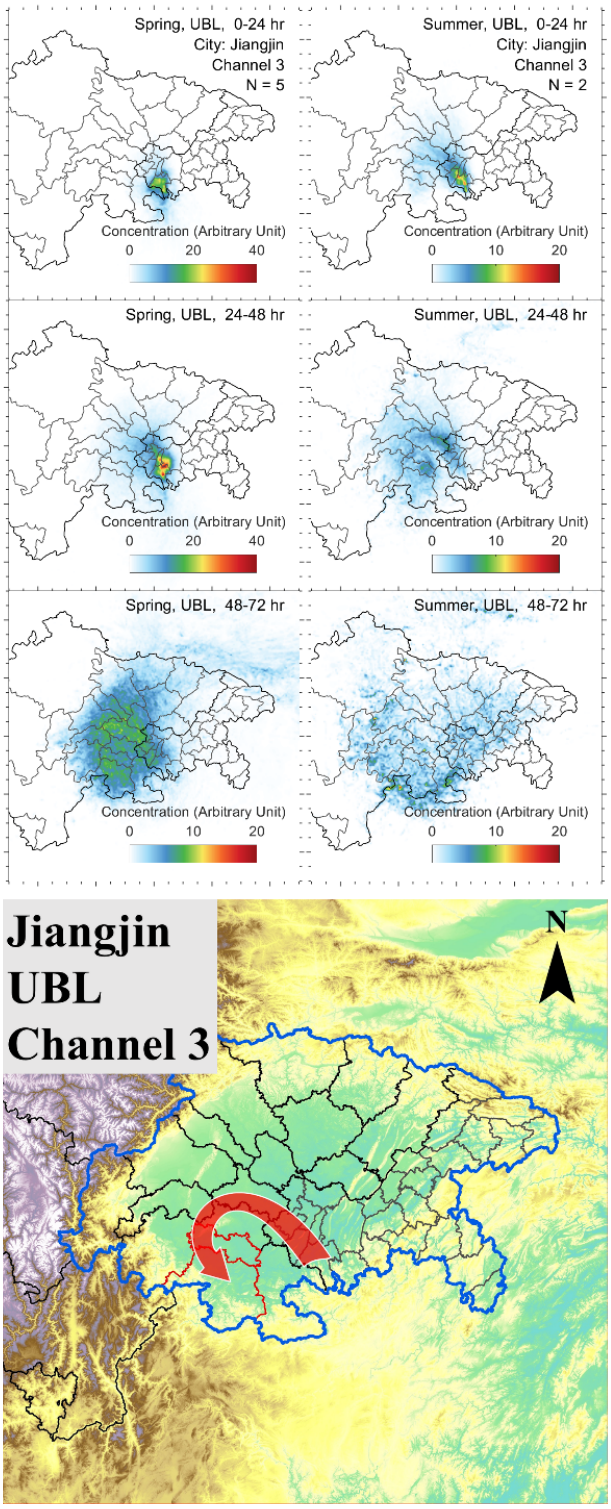

Figure S114 Channel 3 of Jiangjin at UBL.

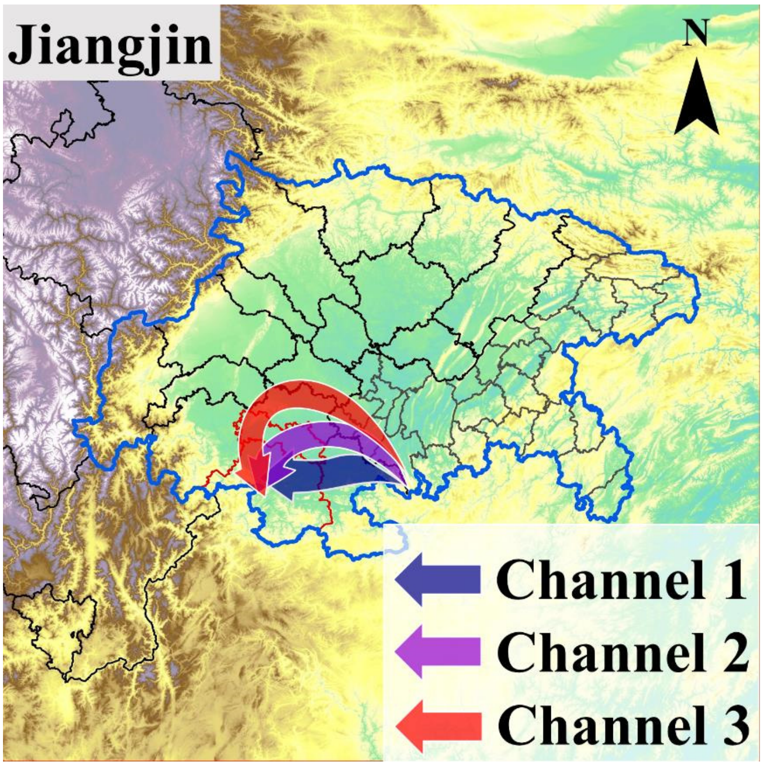

Figure S115 The identified 3 channels originating in Jiangjin.

Table S22 Occurrence frequencies of each channel originating in Jiangjin in four seasons.

City	Channel	Layer	Season			
			autumn	spring	summer	winter
Jiangjin	1	LBL	71.0%	56.7%	9.7%	54.8%
		UBL	51.6%			45.2%
	2	UBL	29.0%	30.0%	9.7%	
	3	LBL			3.2%	
		UBL		16.7%	6.5%	

5.4 Qijiang

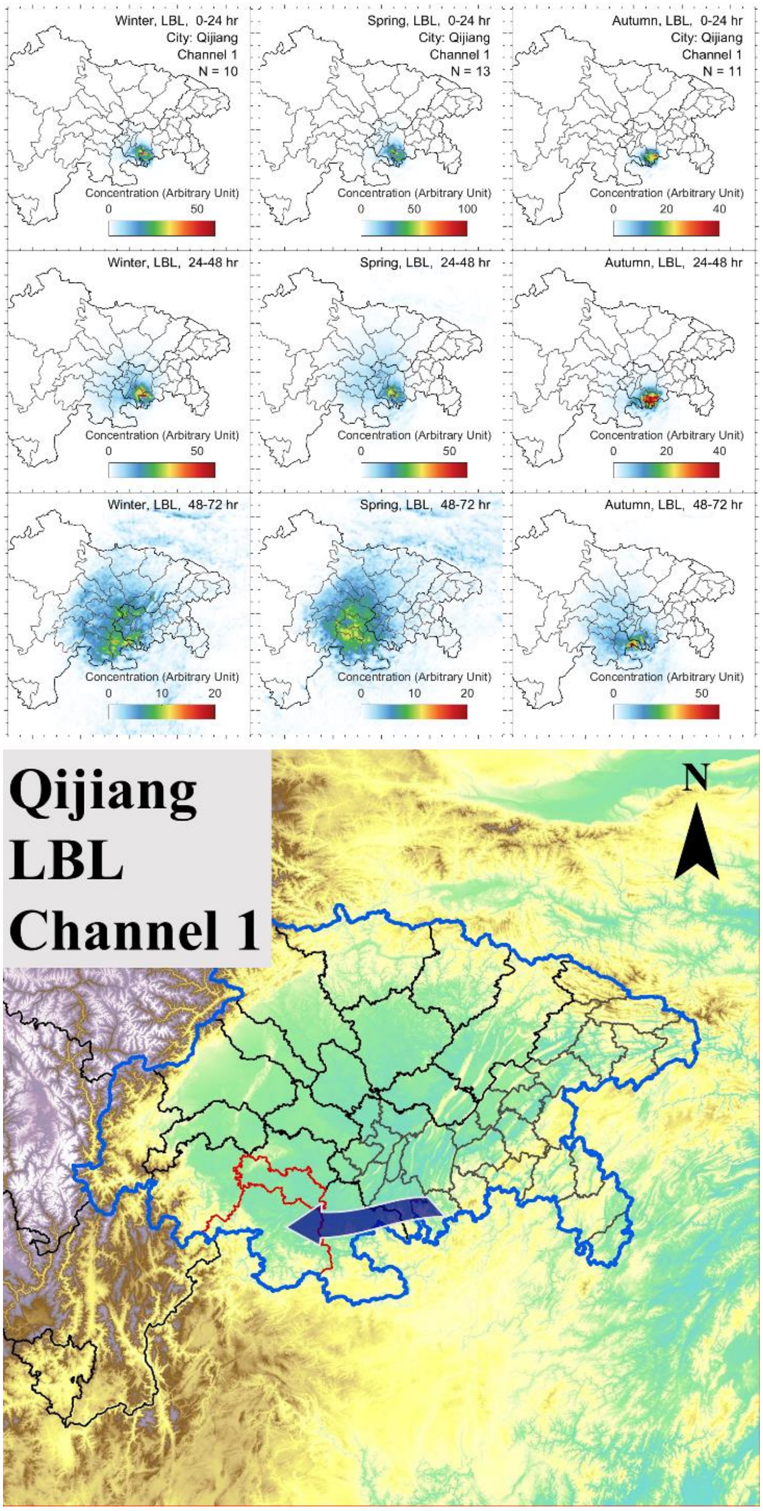

Figure S116 Channel 1 of Qijiang at LBL.

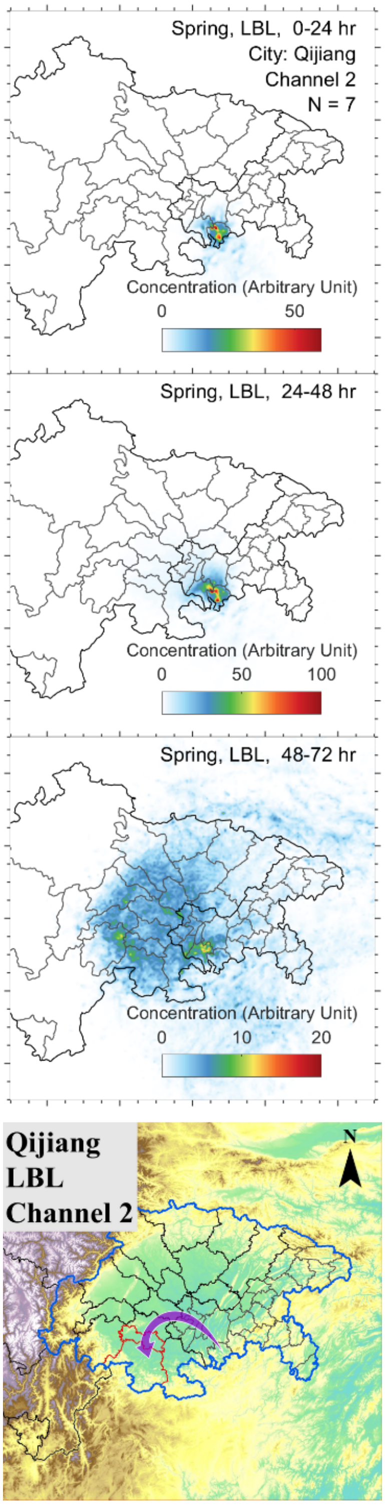

Figure S117 Channel 2 of Qijiang at LBL.

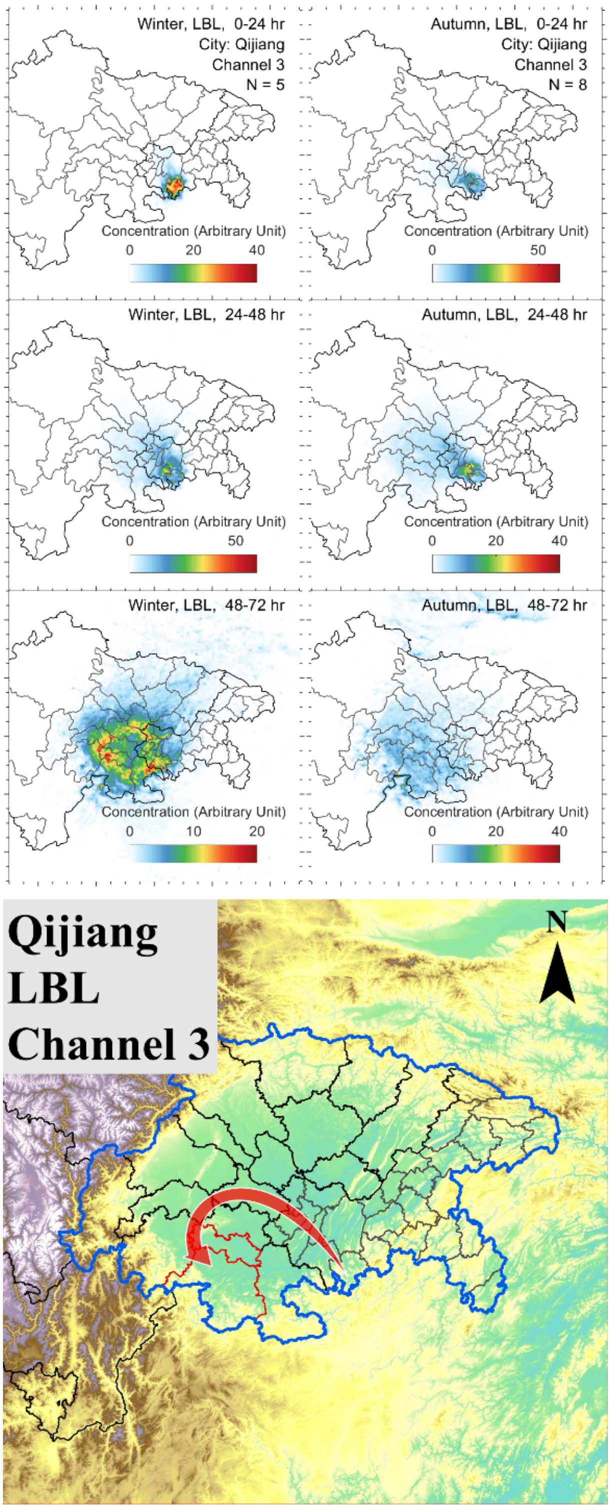

Figure S118 Channel 3 of Qijiang at LBL.

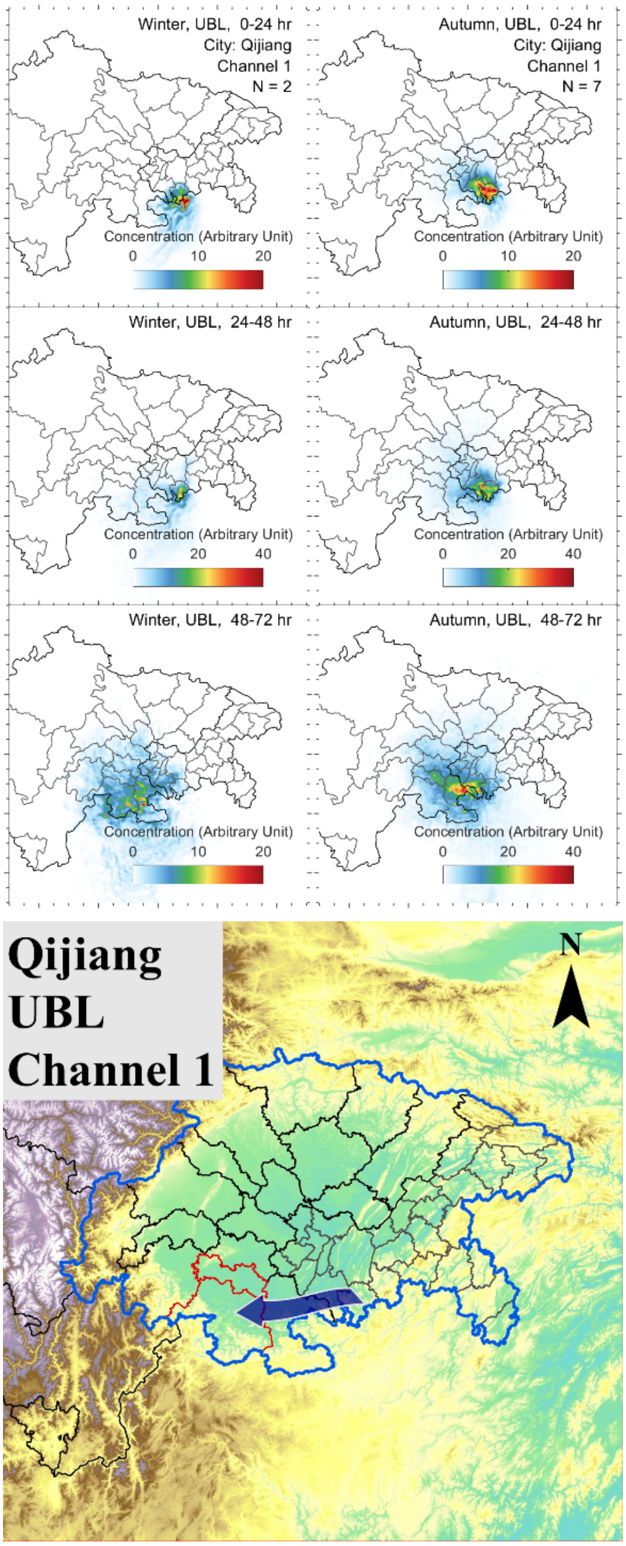

Figure S119 Channel 1 of Qijiang at UBL.

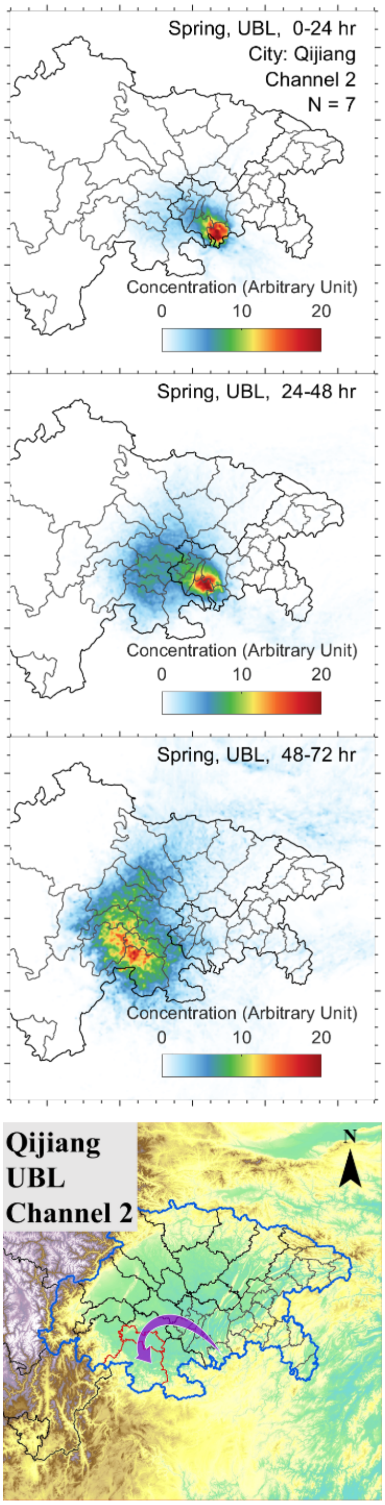

Figure S120 Channel 2 of Qijiang at UBL.

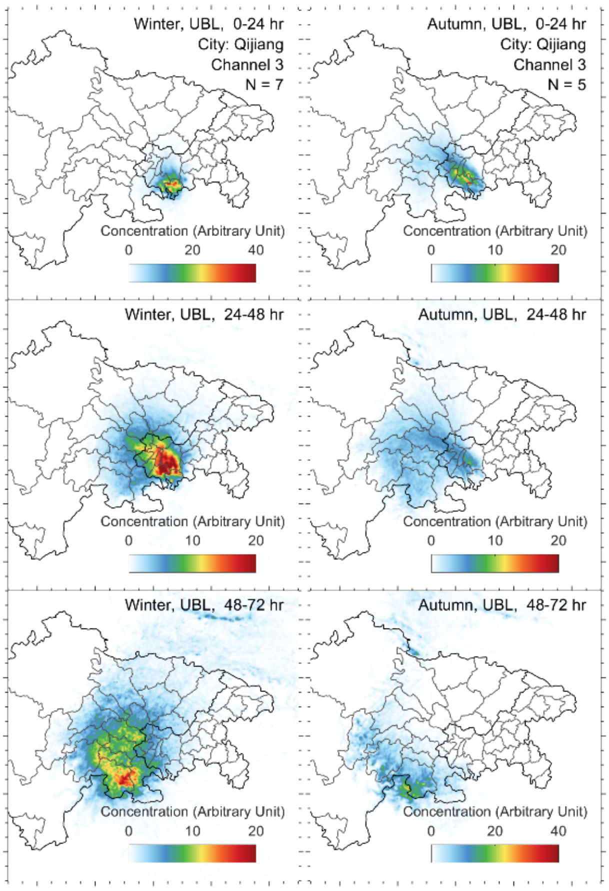

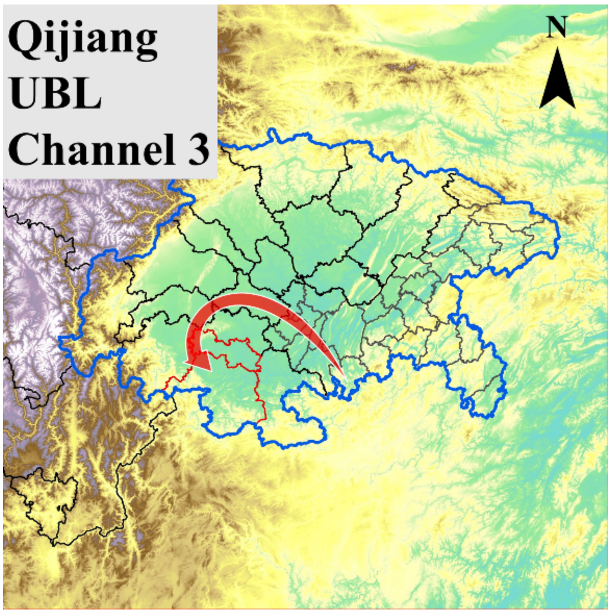

Figure S121 Channel 3 of Qijiang at UBL.

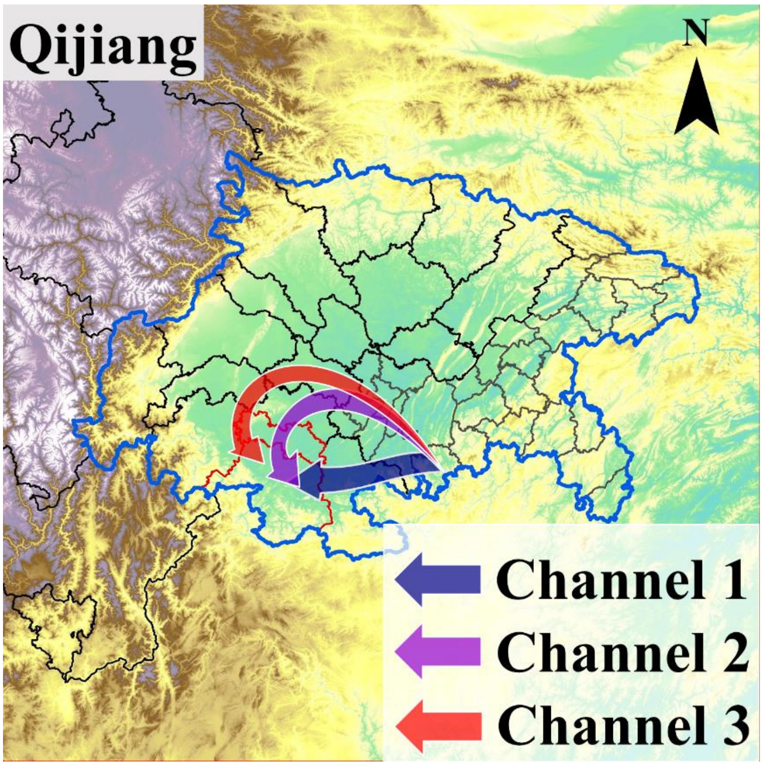

Figure S122 The identified 3 channels originating in Qijiang.

Table S23 Occurrence frequencies of each channel originating in Qijiang in four seasons.

City	Channel	Layer	Season			
			autumn	spring	summer	winter
Qijiang	1	LBL	35.5%	43.3%		32.3%
		UBL	22.6%			6.5%
	2	LBL		23.3%		
		UBL		23.3%		
	3	LBL	25.8%			16.1%
		UBL	16.1%			22.6%

6. Downtown Chongqing

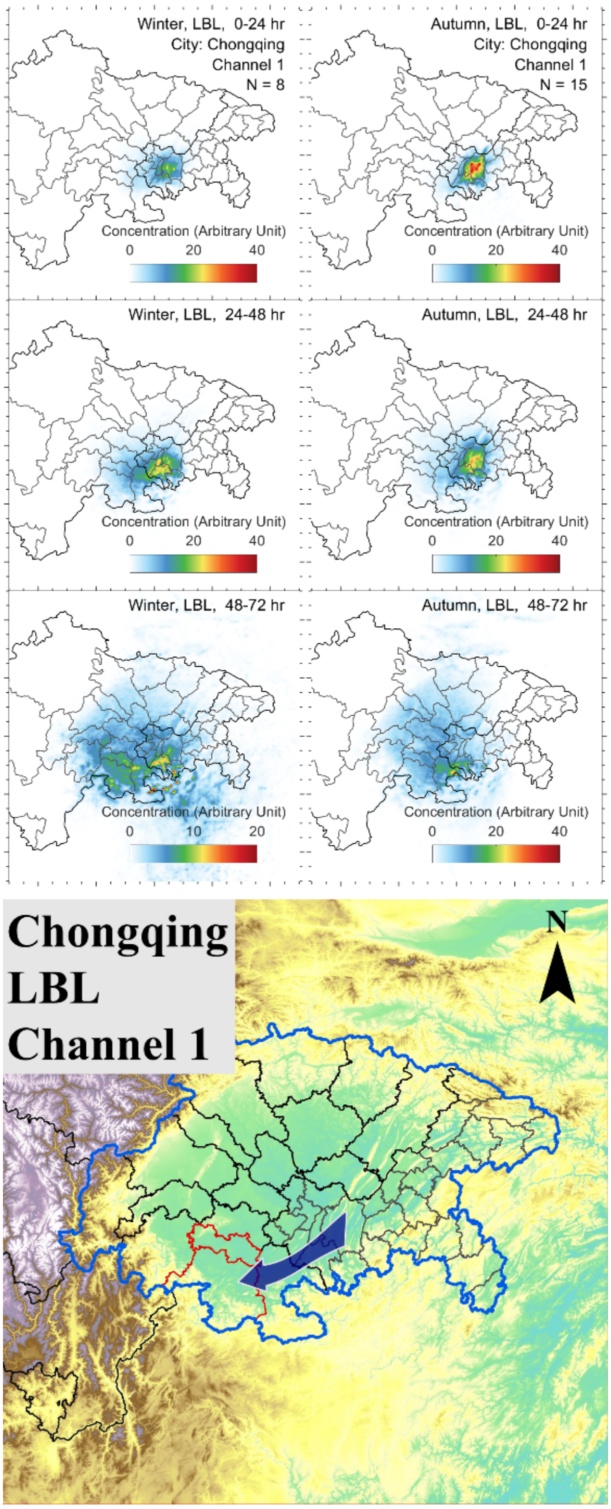

Figure S123 Channel 1 of Chongqing at LBL.

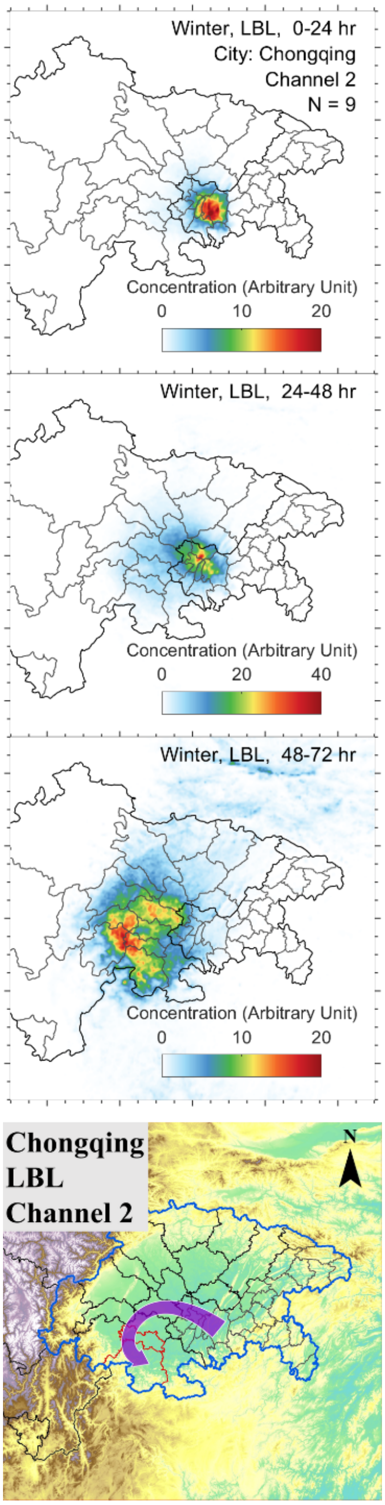

Figure S124 Channel 2 of Chongqing at LBL.

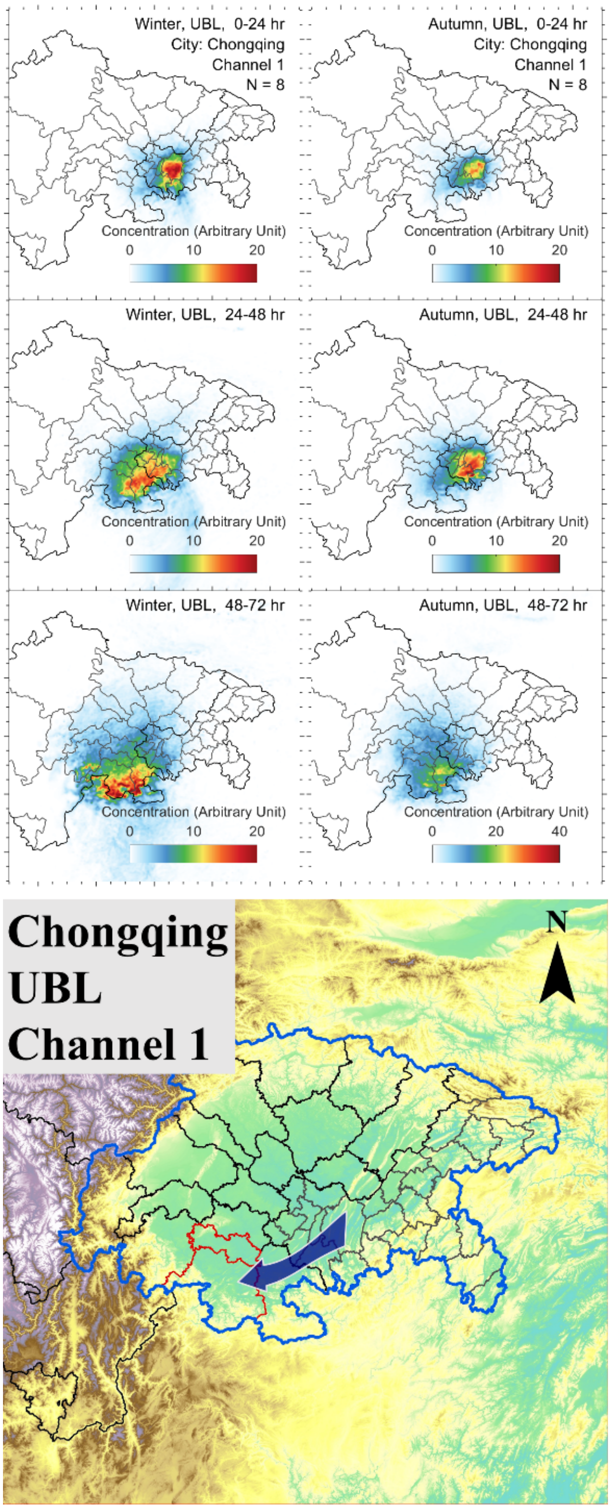

Figure S125 Channel 1 of Chongqing at UBL.

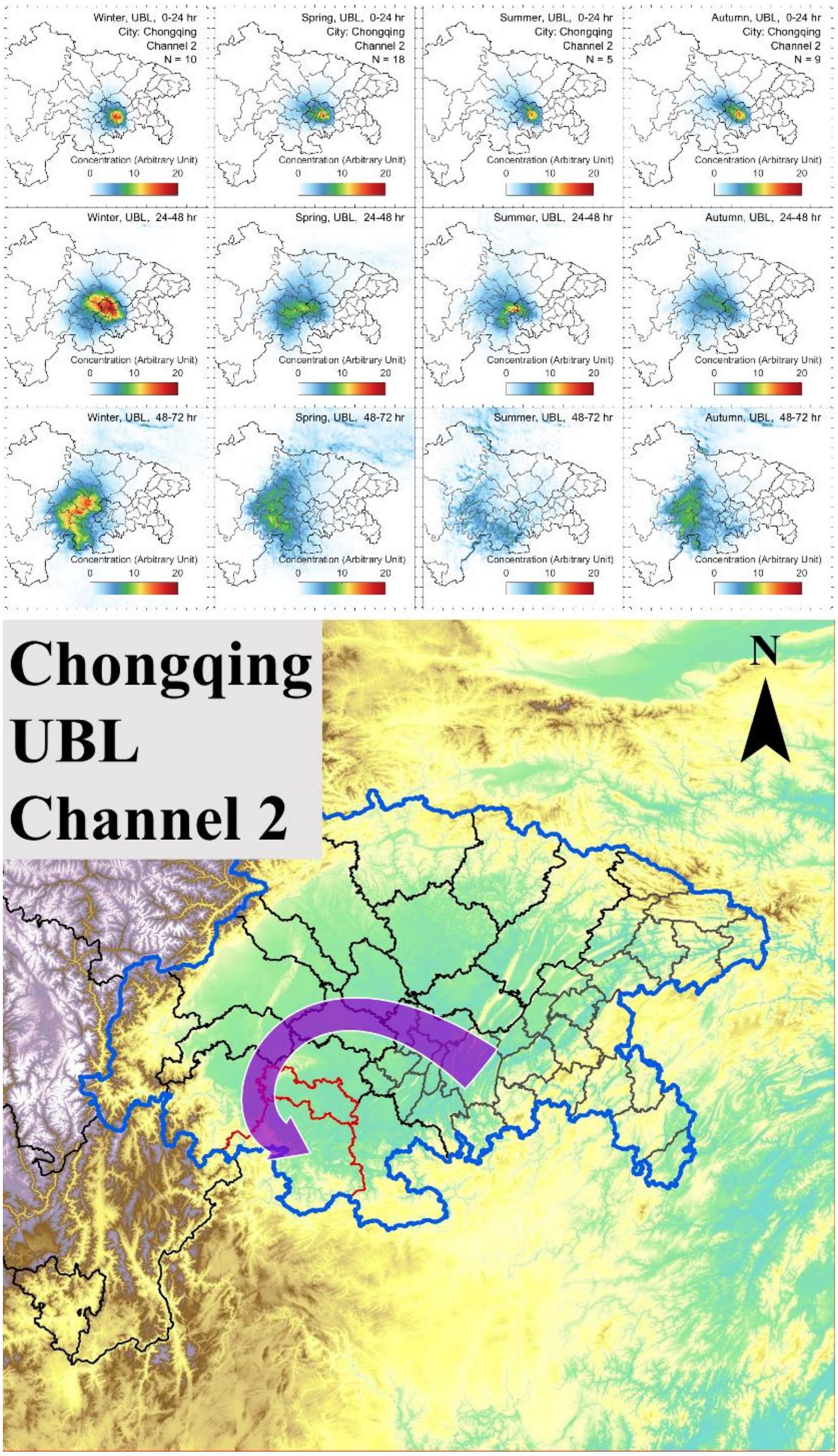

Figure S126 Channel 2 of Chongqing at UBL.

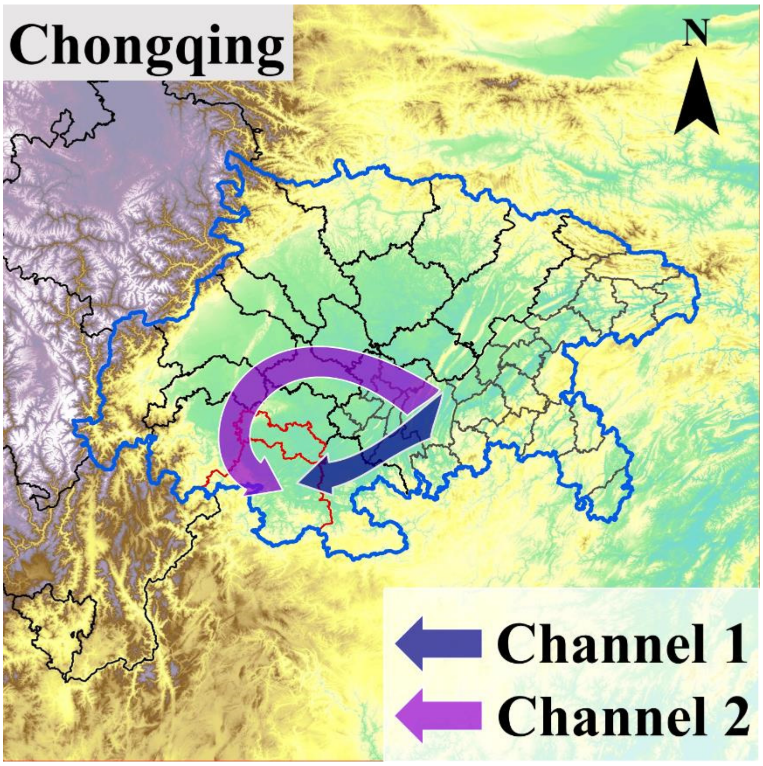

Figure S127 The identified 2 channels originating in Chongqing.

Table S24 Occurrence frequencies of each channel originating in MDC in four seasons.

City	Channel	Layer	Season			
			autumn	spring	summer	winter
MDC	1	LBL	48.4%			25.8%
		UBL	25.8%			25.8%
	2	LBL				29.0%
		UBL	29.0%	60.0%	16.1%	32.3%

7. Eastern Chongqing Urban Agglomeration

7.1 Changshou

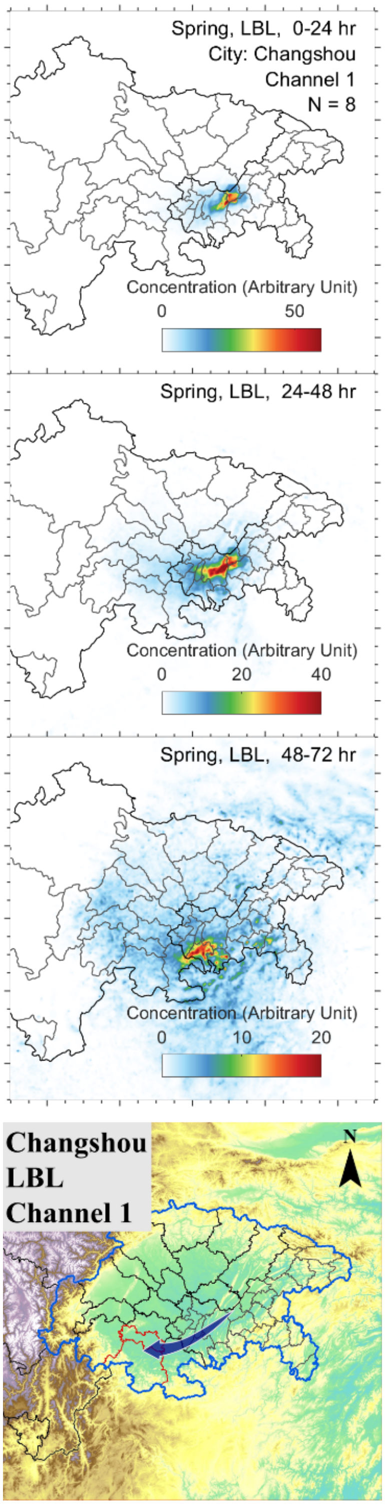

Figure S128 Channel 1 of Changshou at LBL.

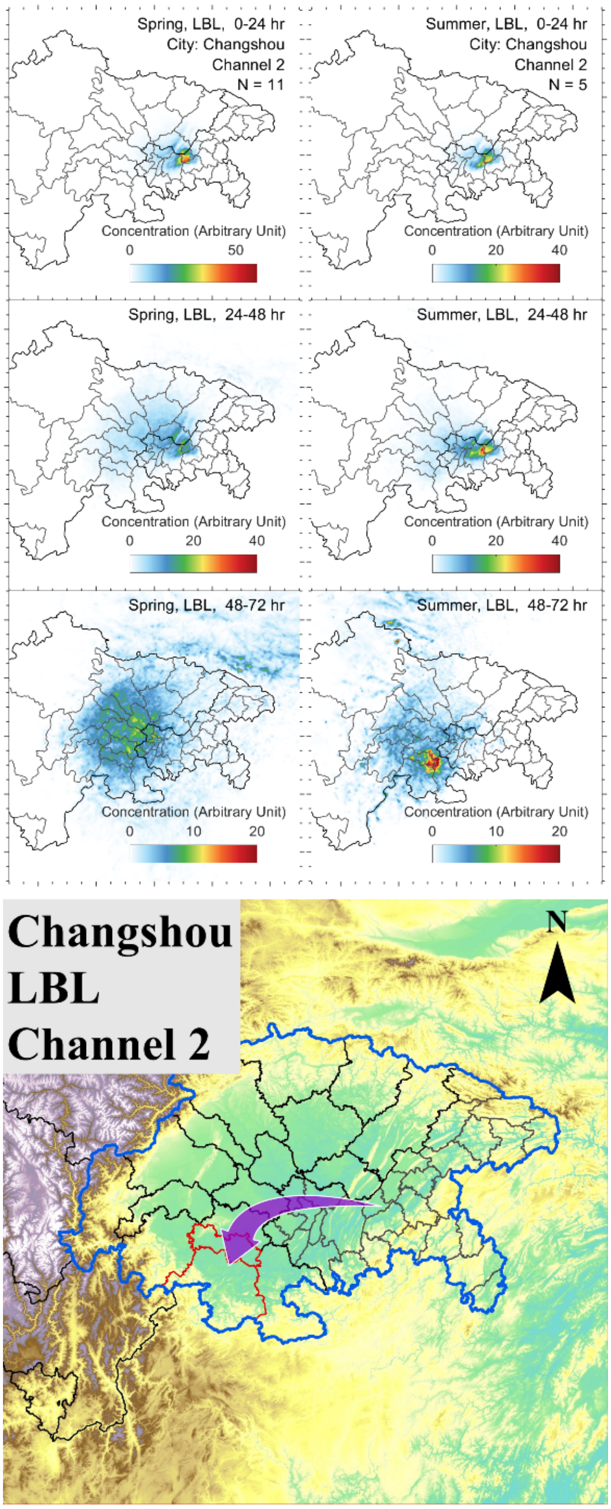

Figure S129 Channel 2 of Changshou at LBL.

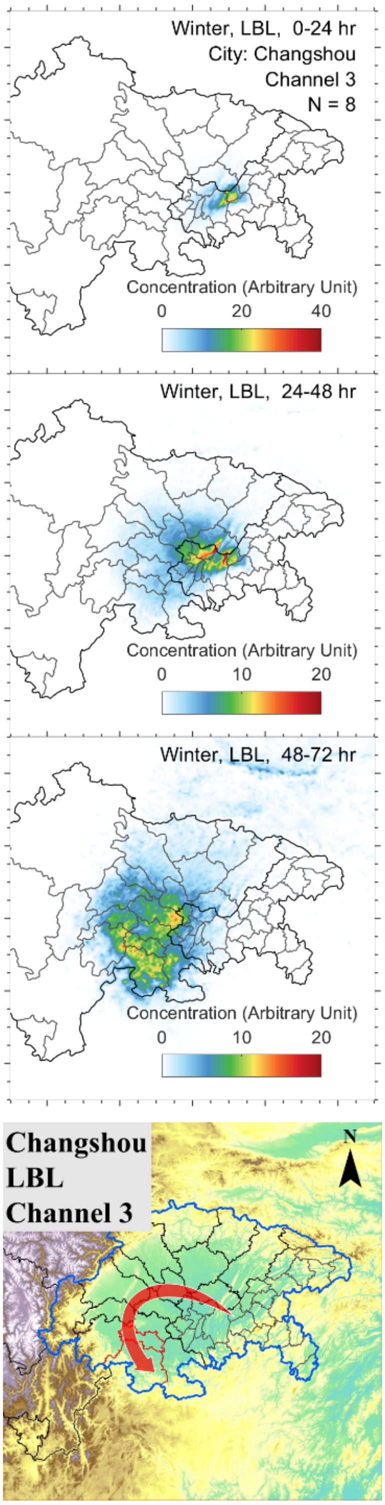

Figure S130 Channel 3 of Changshou at LBL.

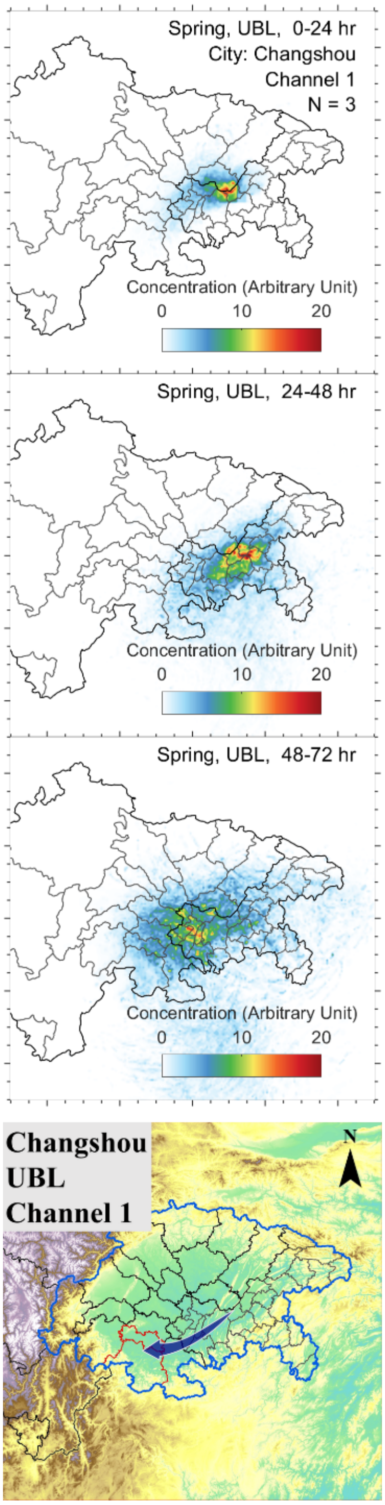

Figure S131 Channel 1 of Changshou at UBL.

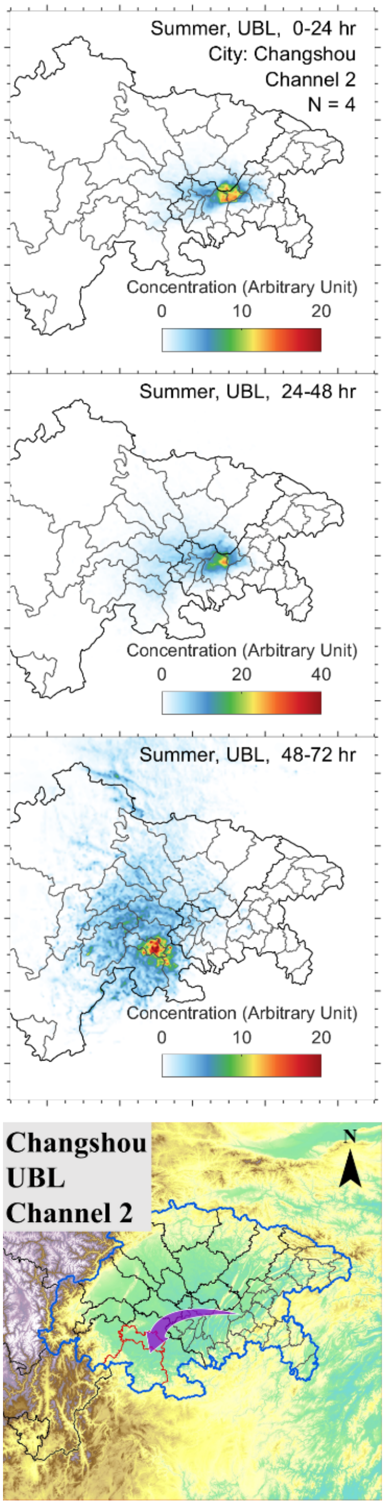

Figure S132 Channel 2 of Changshou at UBL.

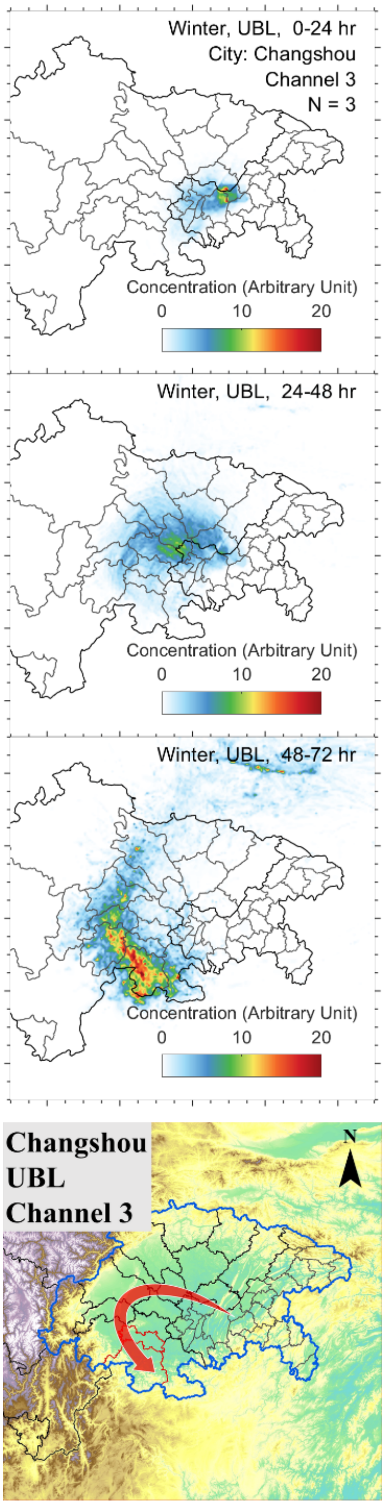

Figure S133 Channel 3 of Changshou at UBL.

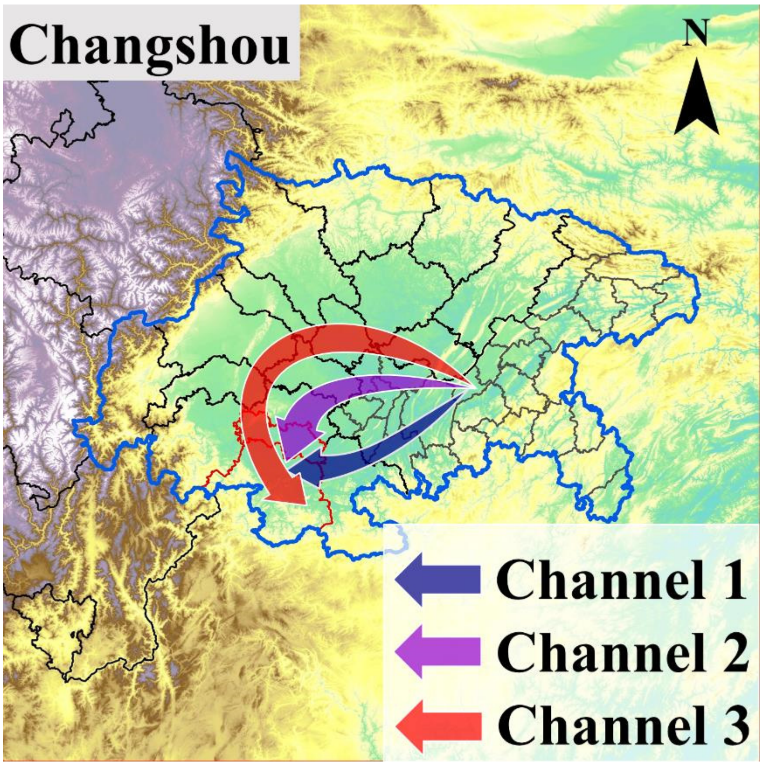

Figure S134 The identified 3 channels originating in Changshou.

Table S25 Occurrence frequencies of each channel originating in Changshou in four seasons.

City	Channel	Layer	Season			
			autumn	spring	summer	winter
Changshou	1	LBL		26.7%		
		UBL		10.0%		
	2	LBL		36.7%	16.1%	
		UBL			12.9%	
	3	LBL				25.8%
		UBL				9.7%

7.2 Dianjiang

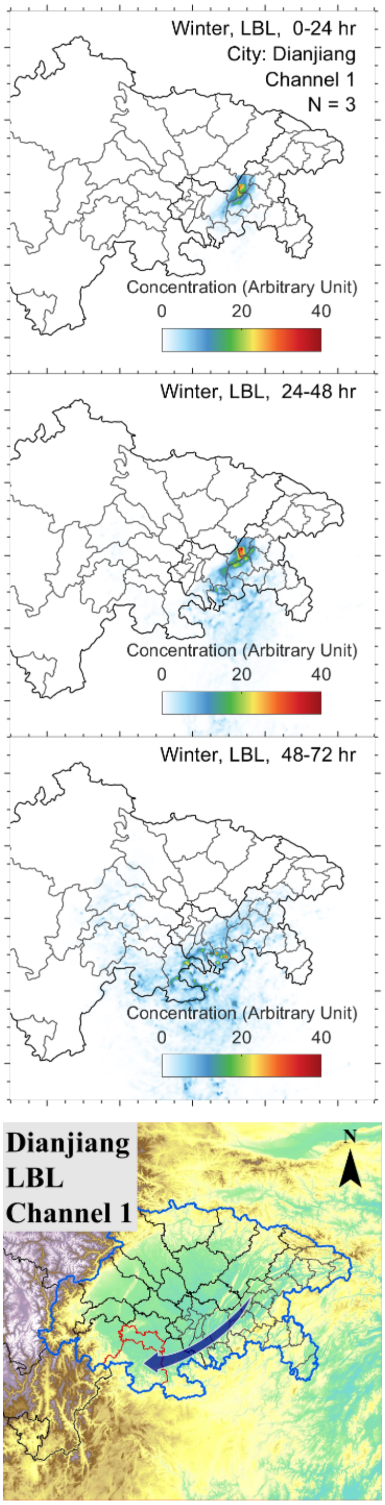

Figure S135 Channel 1 of Dianjiang at LBL.

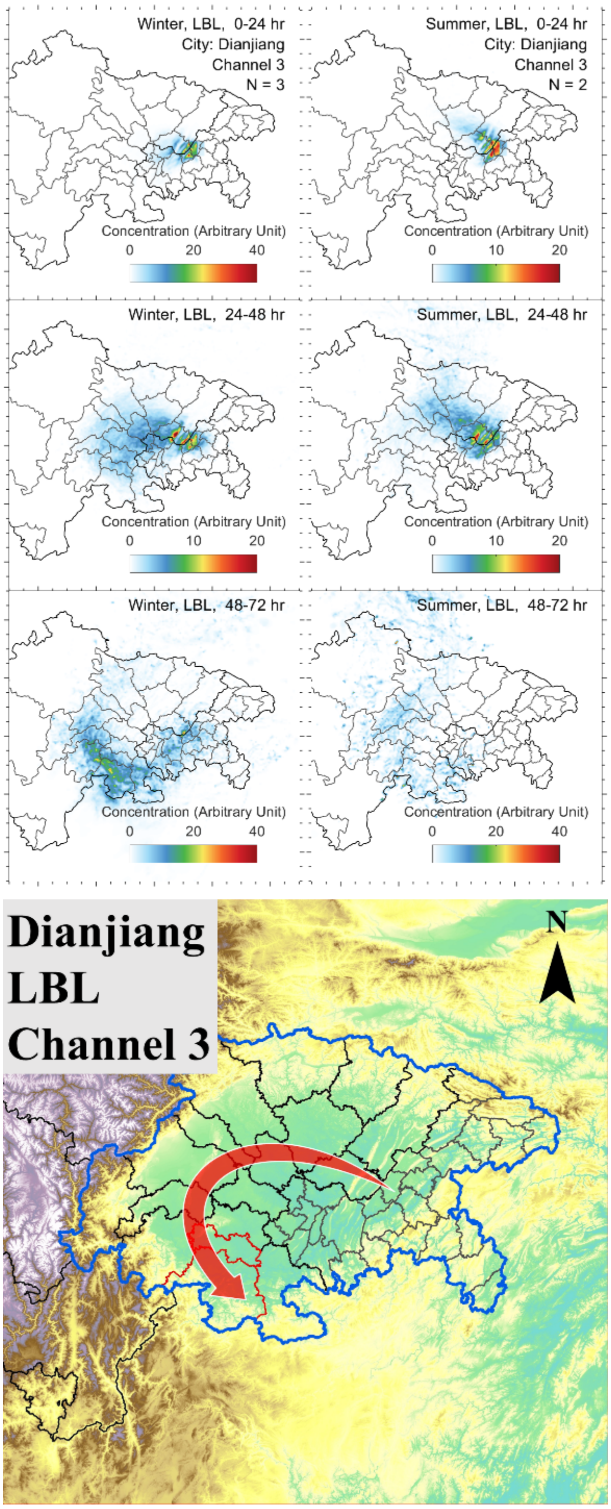

Figure S136 Channel 3 of Dianjiang at LBL.

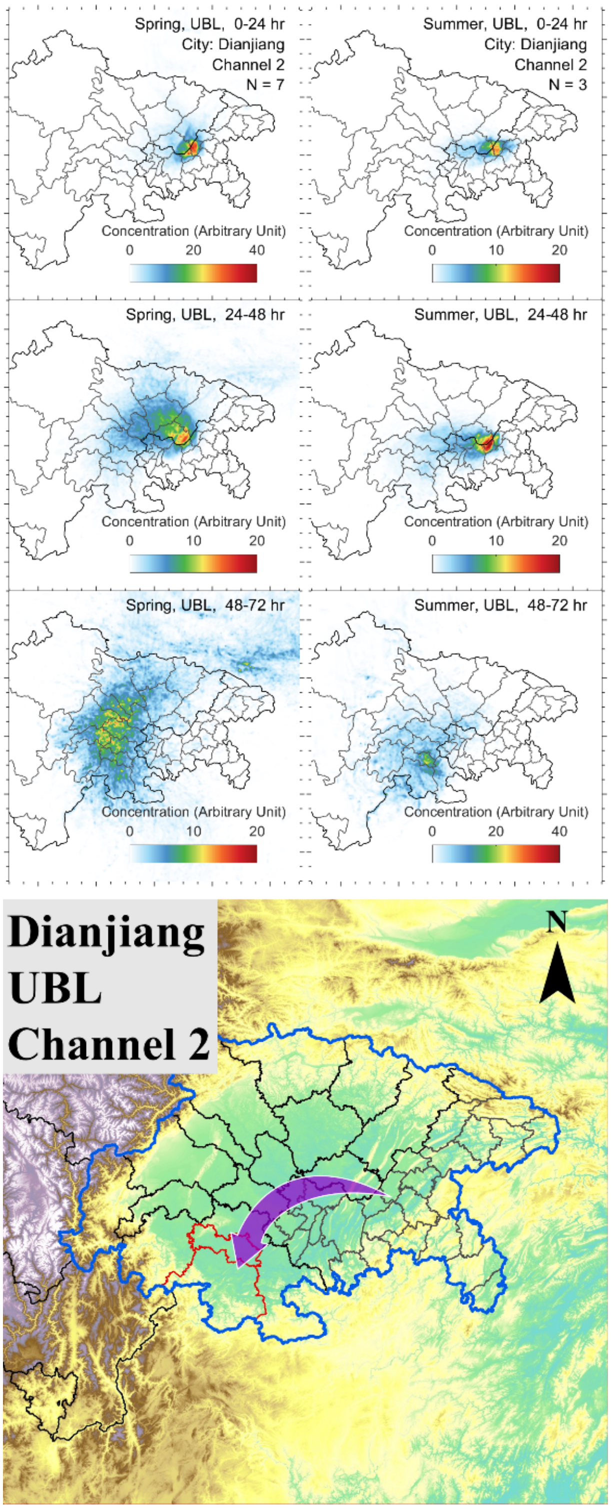

Figure S137 Channel 2 of Dianjiang at UBL.

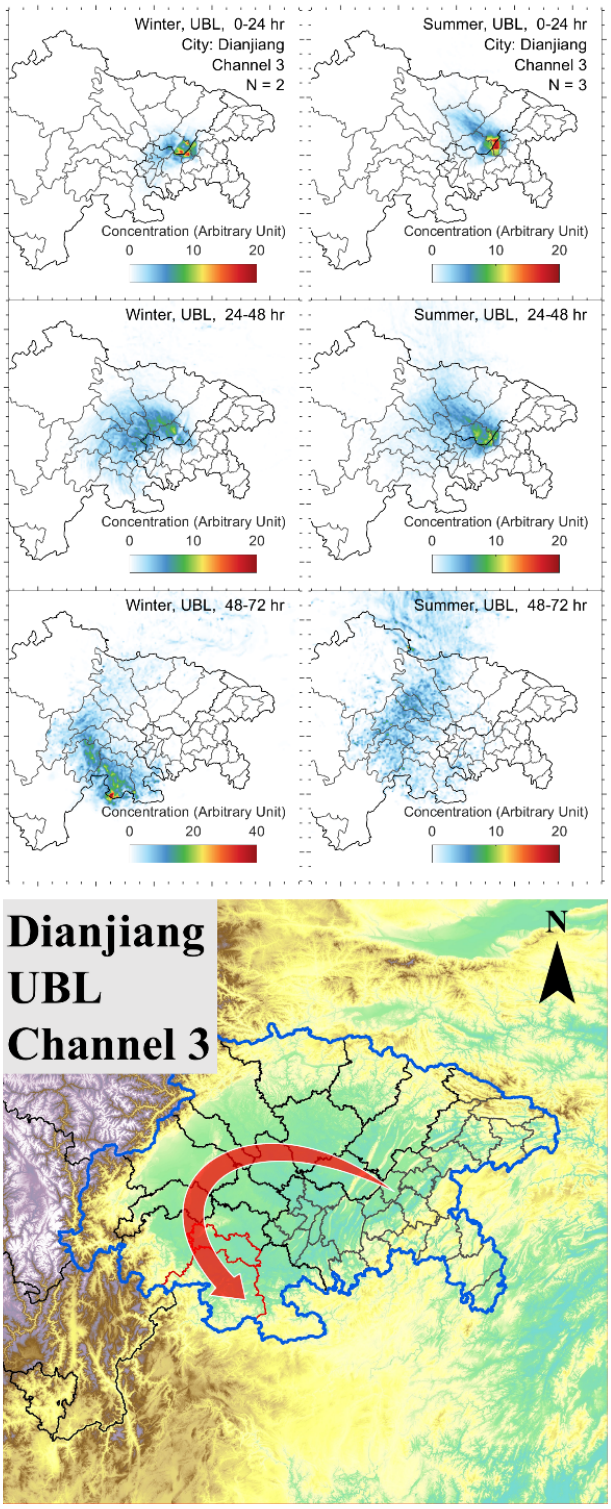

Figure S138 Channel 3 of Dianjiang at UBL.

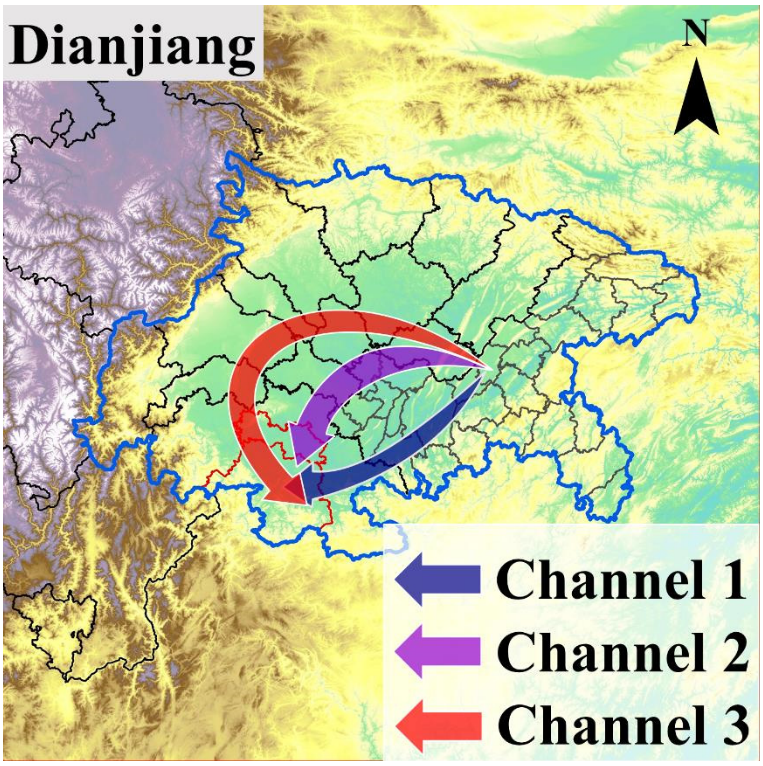

Figure S139 The identified 3 channels originating in Dianjiang.

Table 26 Occurrence frequencies of each channel originating in Dianjiang in four seasons.

City	Channel	Layer	Season			
			autumn	spring	summer	winter
Dianjiang	1	LBL				9.7%
	2	UBL		23.3%	9.7%	
	3	LBL			6.5%	9.7%
		UBL			9.7%	6.5%

7.3 Liangping

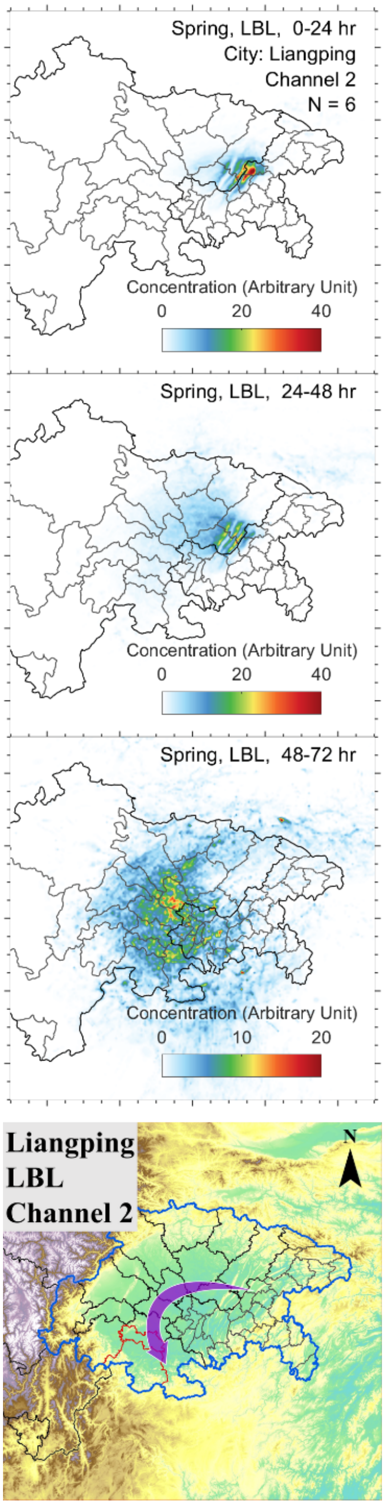

Figure S140 Channel 2 of Liangping at LBL.

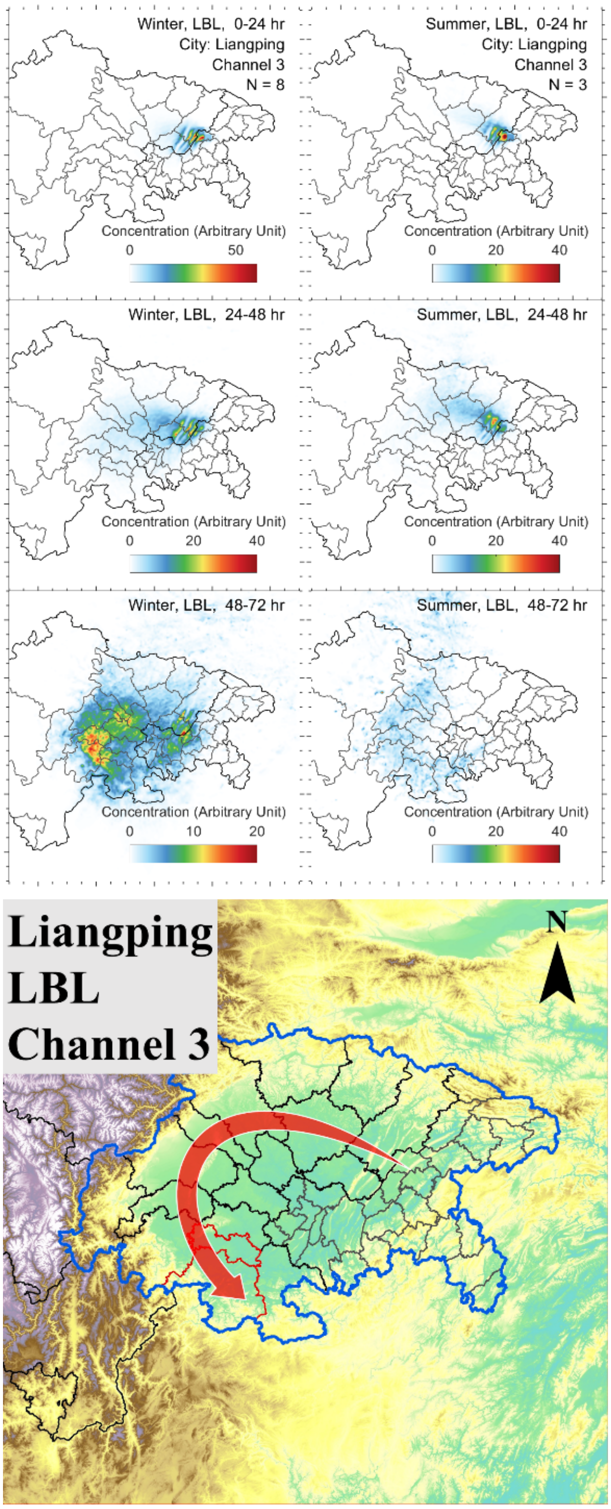

Figure S141 Channel 3 of Liangping at LBL.

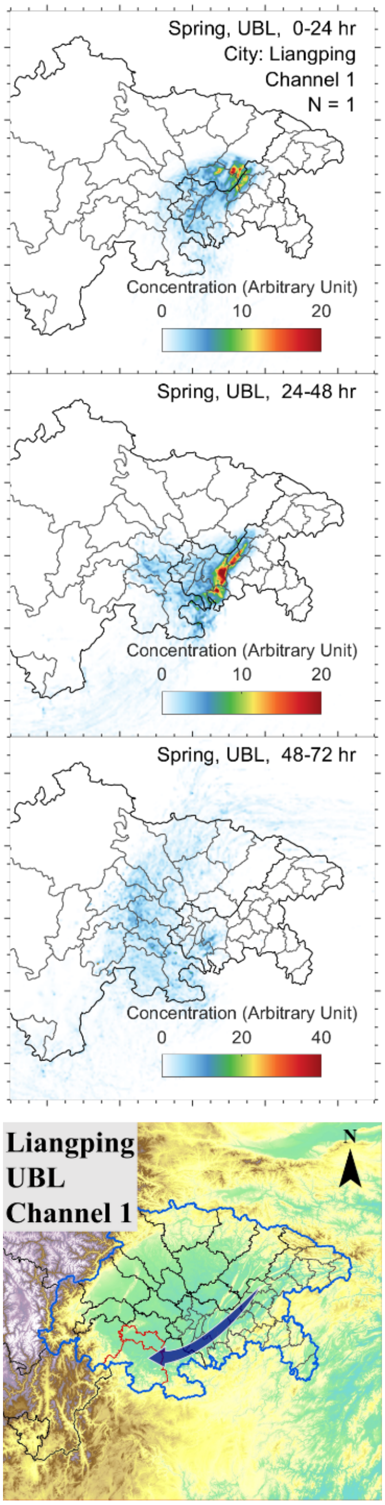

Figure S142 Channel 1 of Liangping at UBL.

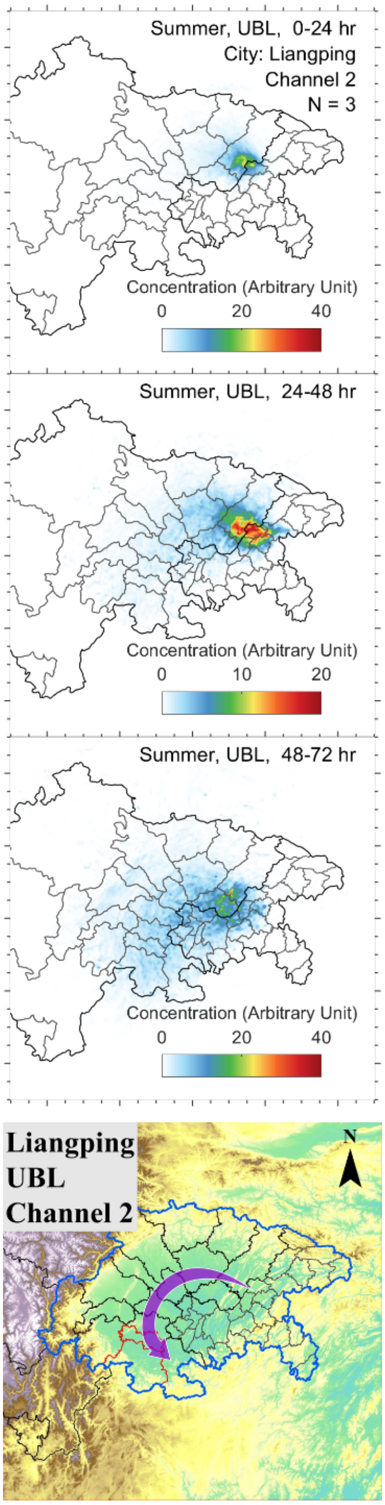

Figure S143 Channel 2 of Liangping at UBL.

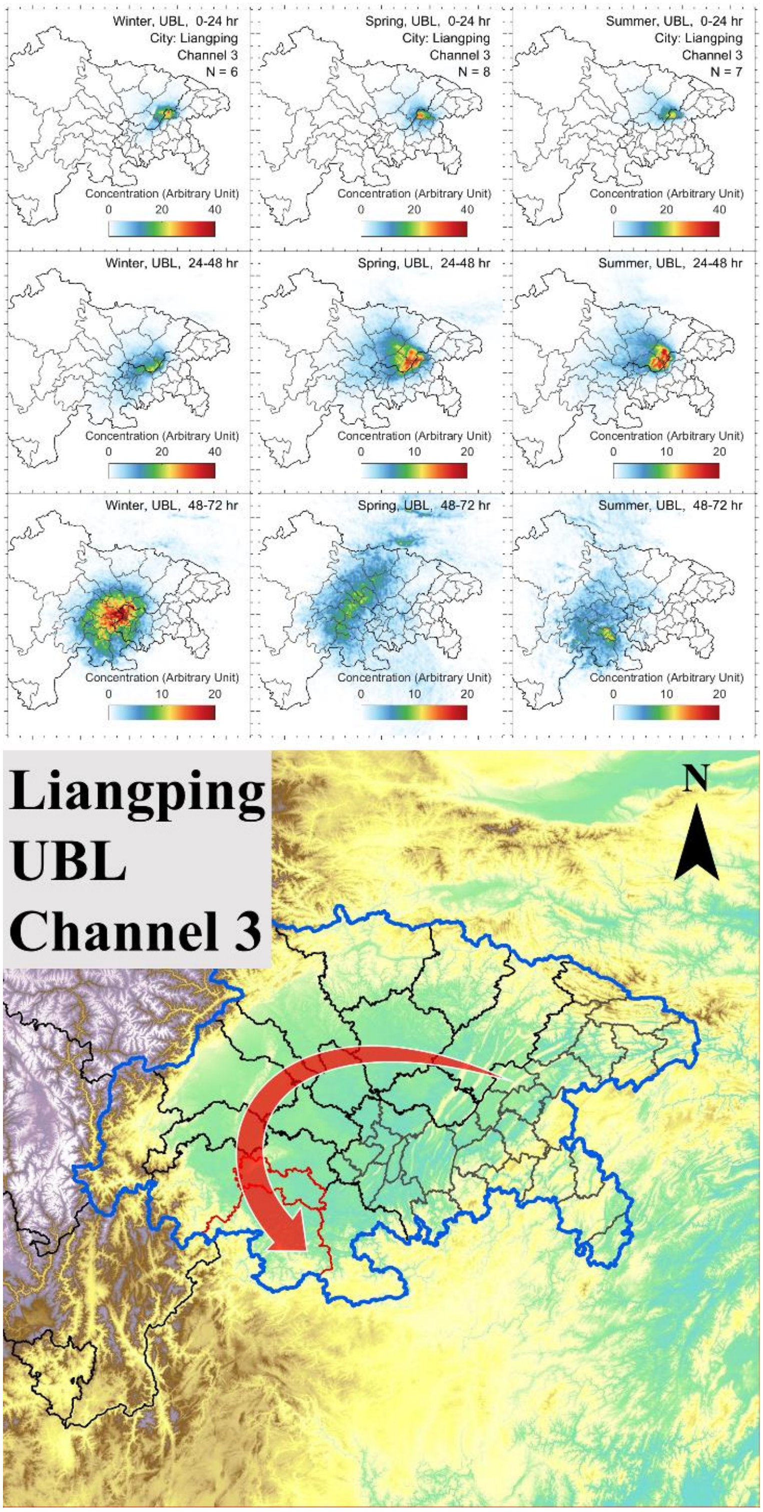

Figure S144 Channel 3 of Liangping at UBL.

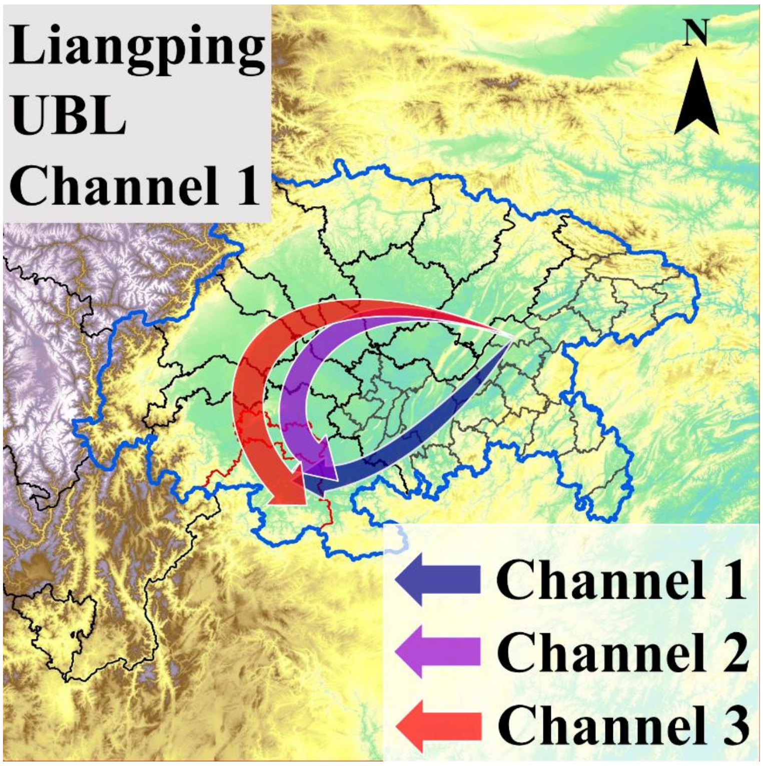

Figure S145 The identified 3 channels originating in Liangping.

Table S27 Occurrence frequencies of each channel originating in Liangping in four seasons.

City	Channel	Layer	Season			
			autumn	spring	summer	winter
Liangping	1	UBL		3.3%		
	2	LBL		20.0%		
		UBL			9.7%	
	3	LBL			9.7%	25.8%
		UBL		26.7%	22.6%	19.4%

7.4 Zhongxian

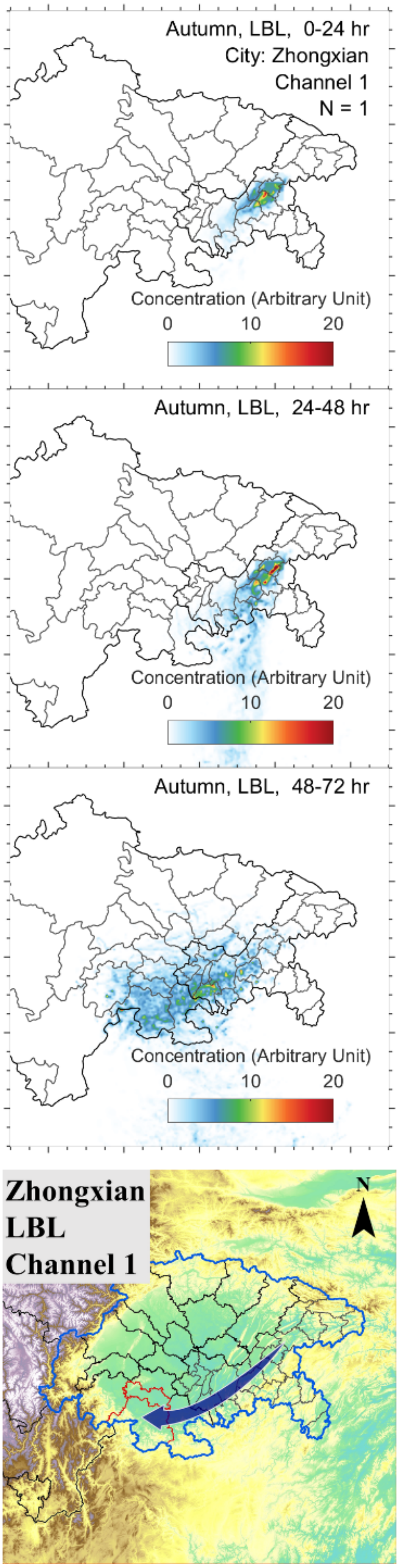

Autumn, LBL, 0-24 hr
City: Zhongxian
Channel 1
N = 1
Concentration (Arbitrary Unit)
0
10
20
Autumn, LBL, 24-48 hr
Concentration (Arbitrary Unit)
0
10
20
Autumn, LBL, 48-72 hr
Concentration (Arbitrary Unit)
0
10
20
Zhongxian
LBL
Channel 1
N

Figure S146 Channel 1 of Zhongxian at LBL.

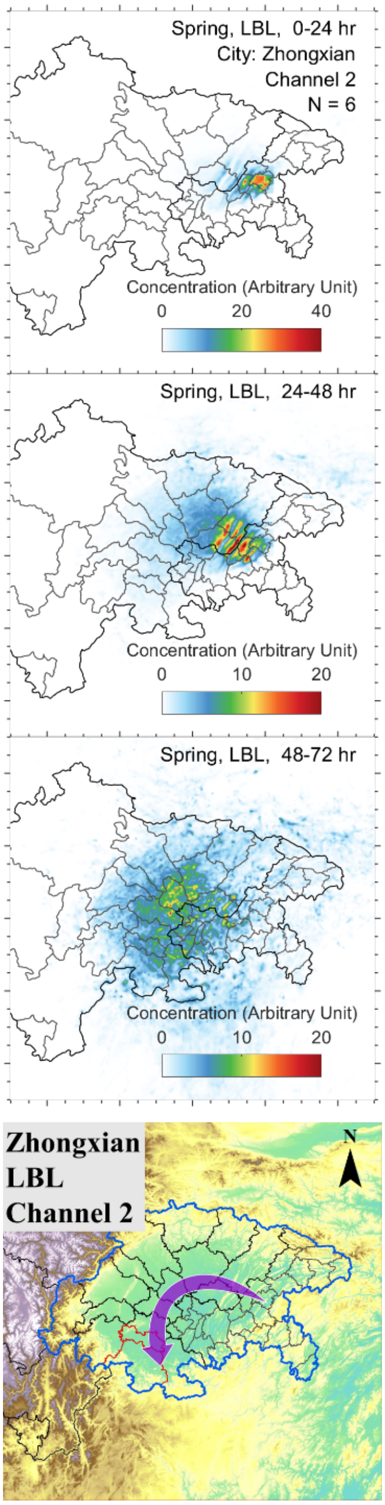

Figure S147 Channel 2 of Zhongxian at LBL.

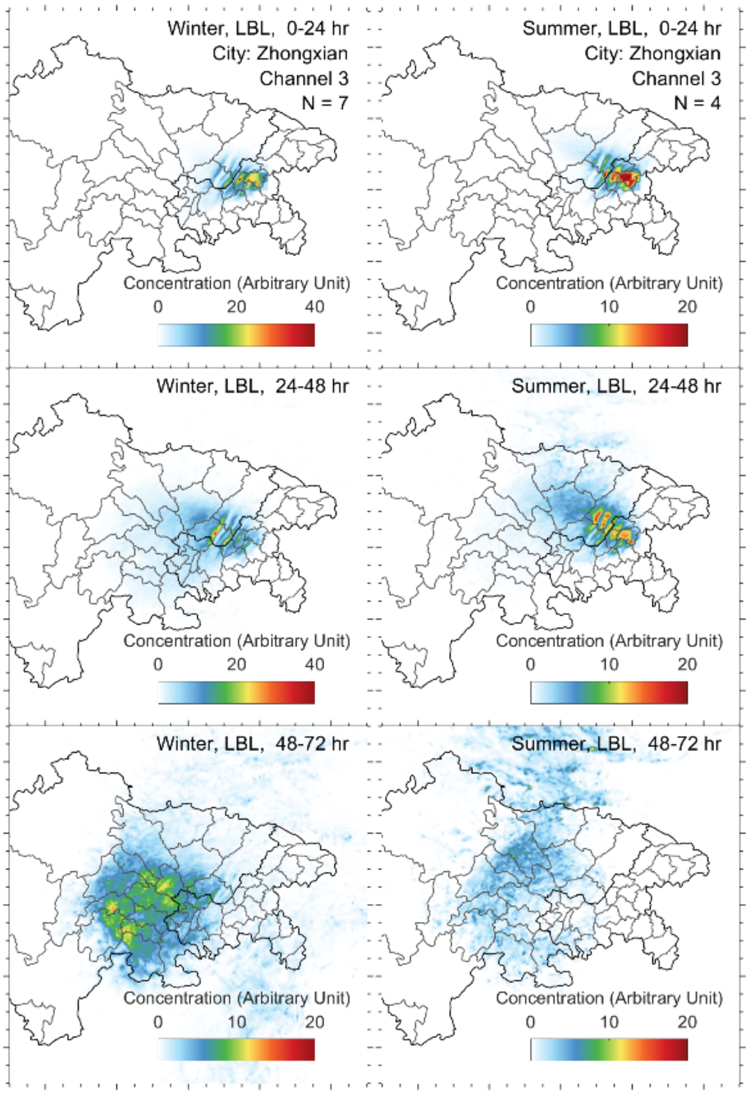

Winter, LBL, 0-24 hr
City: Zhongxian
Channel 3
N = 7
Concentration (Arbitrary Unit)
0 20 40
Summer, LBL, 0-24 hr
City: Zhongxian
Channel 3
N = 4
Concentration (Arbitrary Unit)
0 10 20
Winter, LBL, 24-48 hr
Concentration (Arbitrary Unit)
0 20 40
Summer, LBL, 24-48 hr
Concentration (Arbitrary Unit)
0 10 20
Winter, LBL, 48-72 hr
Concentration (Arbitrary Unit)
0 10 20
Summer, LBL, 48-72 hr
Concentration (Arbitrary Unit)
0 10 20

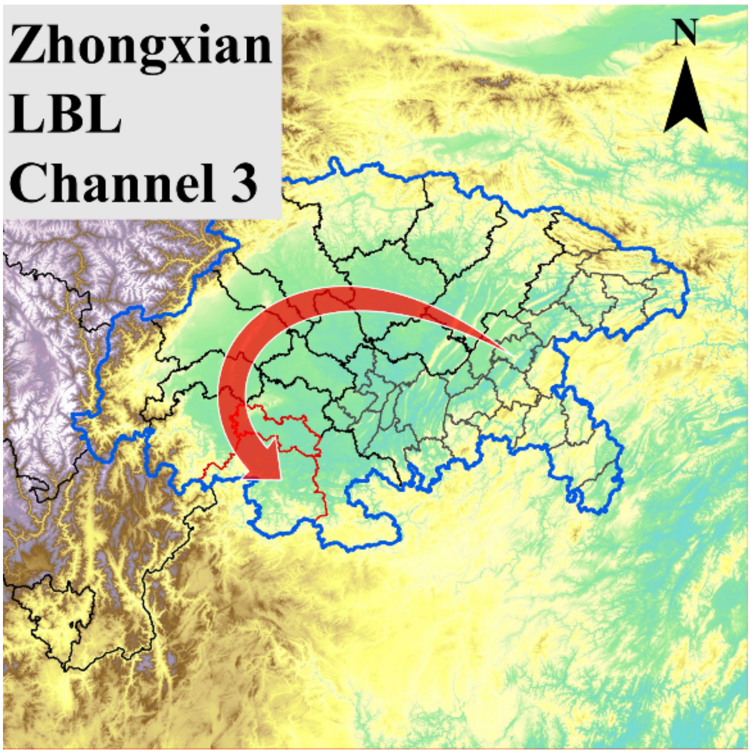

Zhongxian
LBL
Channel 3
N

Figure S148 Channel 3 of Zhongxian at LBL.

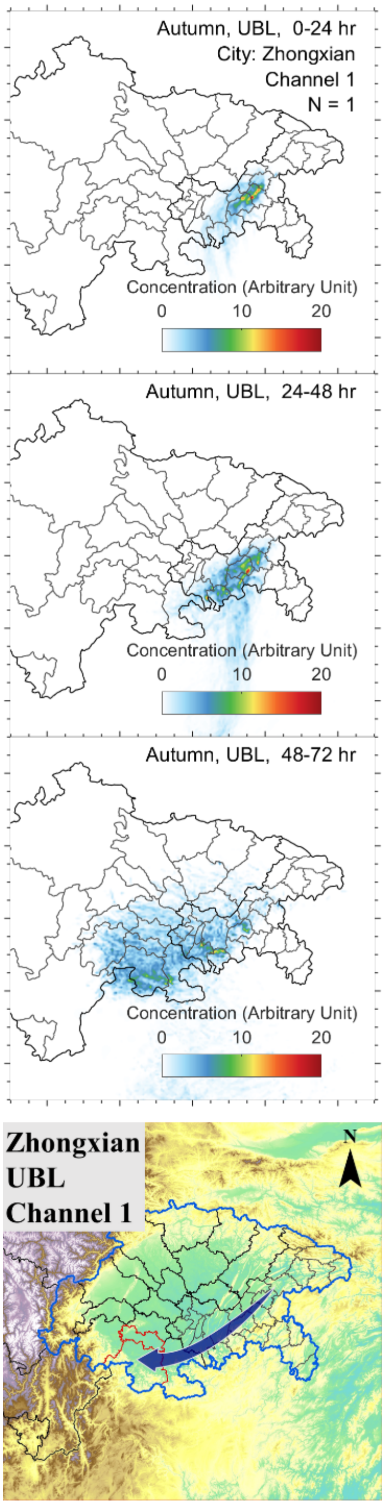

Figure S149 Channel 1 of Zhongxian at UBL.

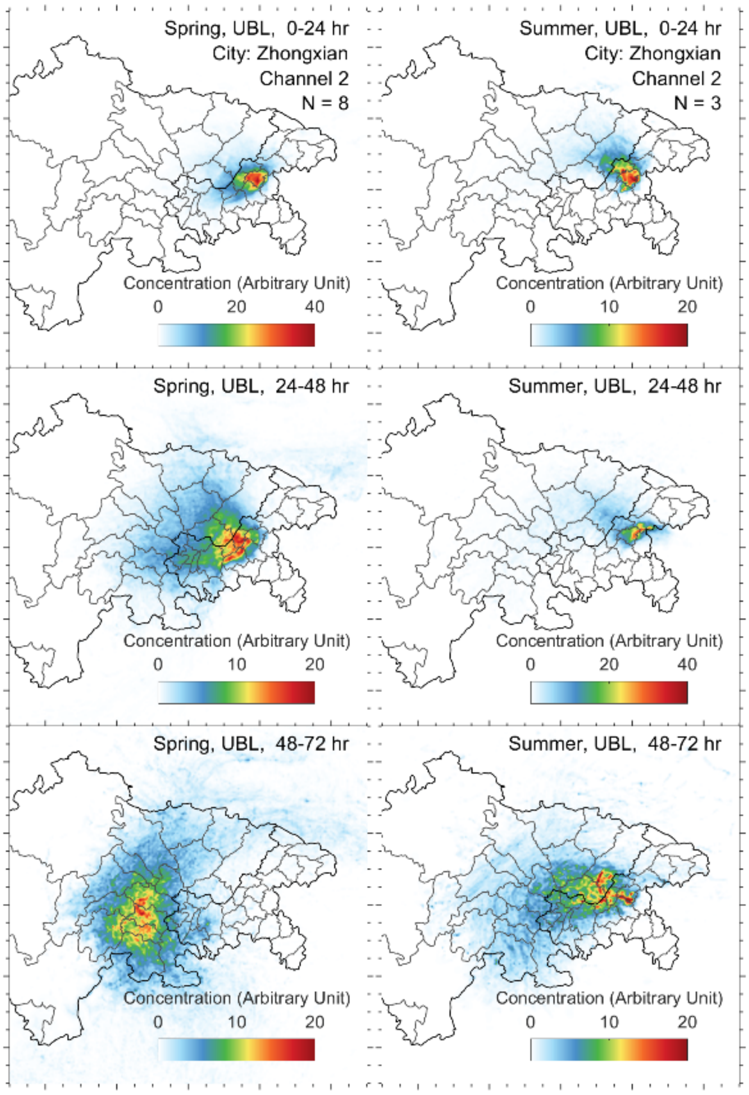
Spring, UBL, 0-24 hr
City: Zhongxian
Channel 2
N = 8
Concentration (Arbitrary Unit)
0 20 40
Summer, UBL, 0-24 hr
City: Zhongxian
Channel 2
N = 3
Concentration (Arbitrary Unit)
0 10 20
Spring, UBL, 24-48 hr
Concentration (Arbitrary Unit)
0 10 20
Summer, UBL, 24-48 hr
Concentration (Arbitrary Unit)
0 20 40
Spring, UBL, 48-72 hr
Concentration (Arbitrary Unit)
0 10 20
Summer, UBL, 48-72 hr
Concentration (Arbitrary Unit)
0 10 20

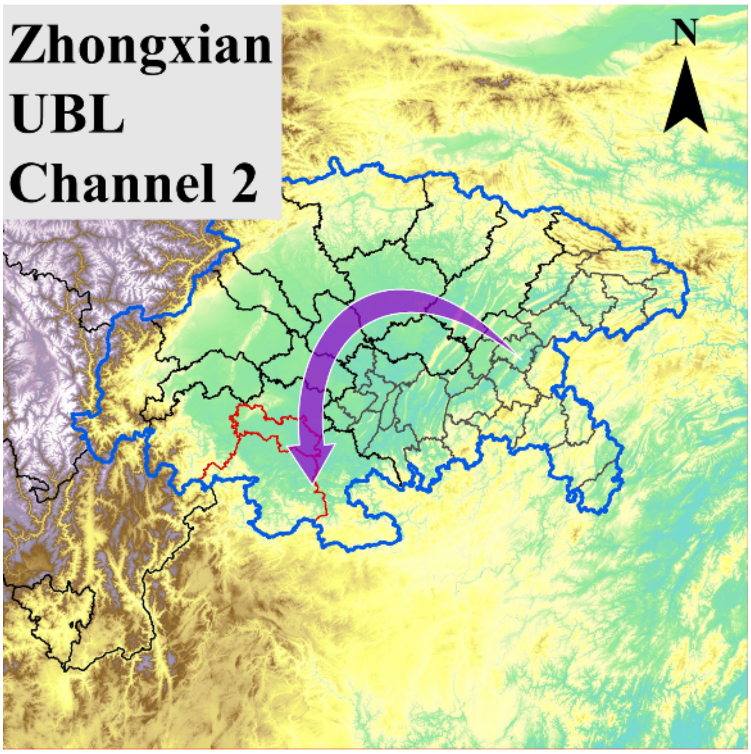
Zhongxian
UBL
Channel 2
N

Figure S150 Channel 2 of Zhongxian at UBL.

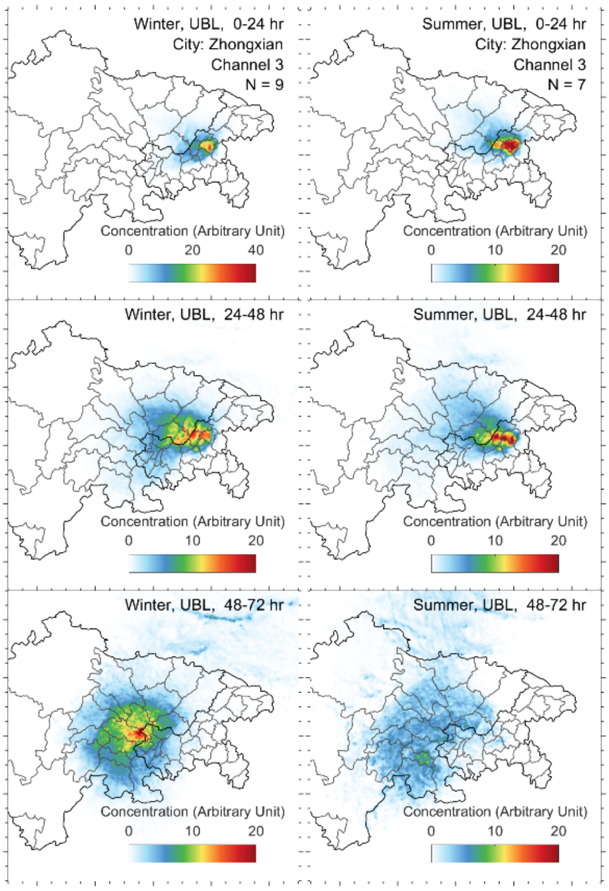

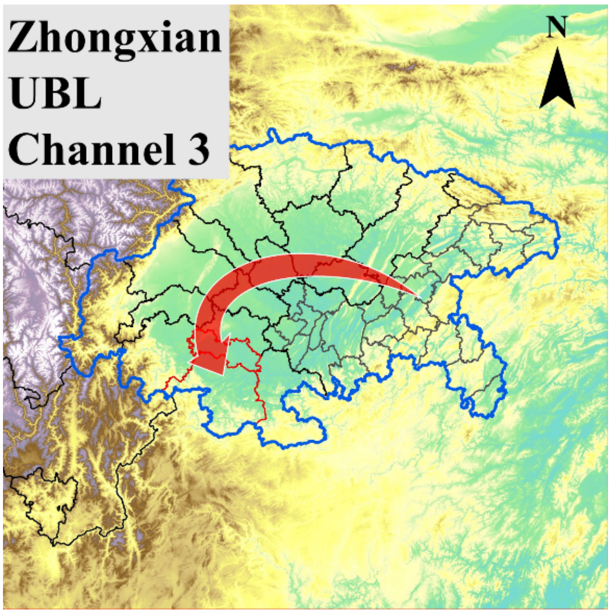

Figure S151 Channel 3 of Zhongxian at UBL.

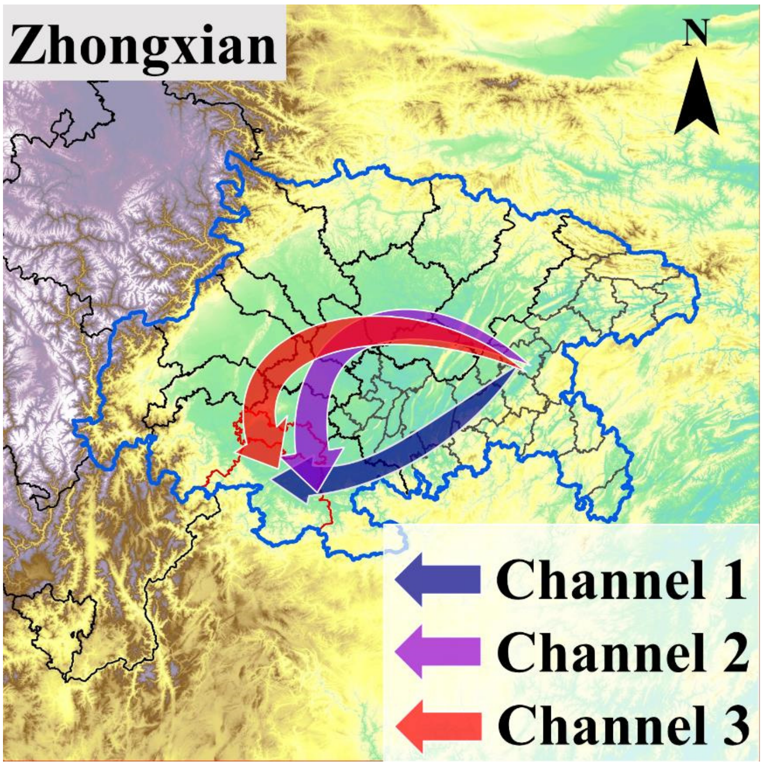

Figure S152 The identified 3 channels originating in.

Table S28 Occurrence frequencies of each channel originating in Zhongxian in four seasons.

City	Channel	Layer	Season			
			autumn	spring	summer	winter
Zhongxian	1	LBL	3.2%			
		UBL	3.2%			
	2	LBL		20.0%		
		UBL		26.7%	9.7%	
	3	LBL			12.9%	22.6%
		UBL			22.6%	29.0%

7.5 Fengdu

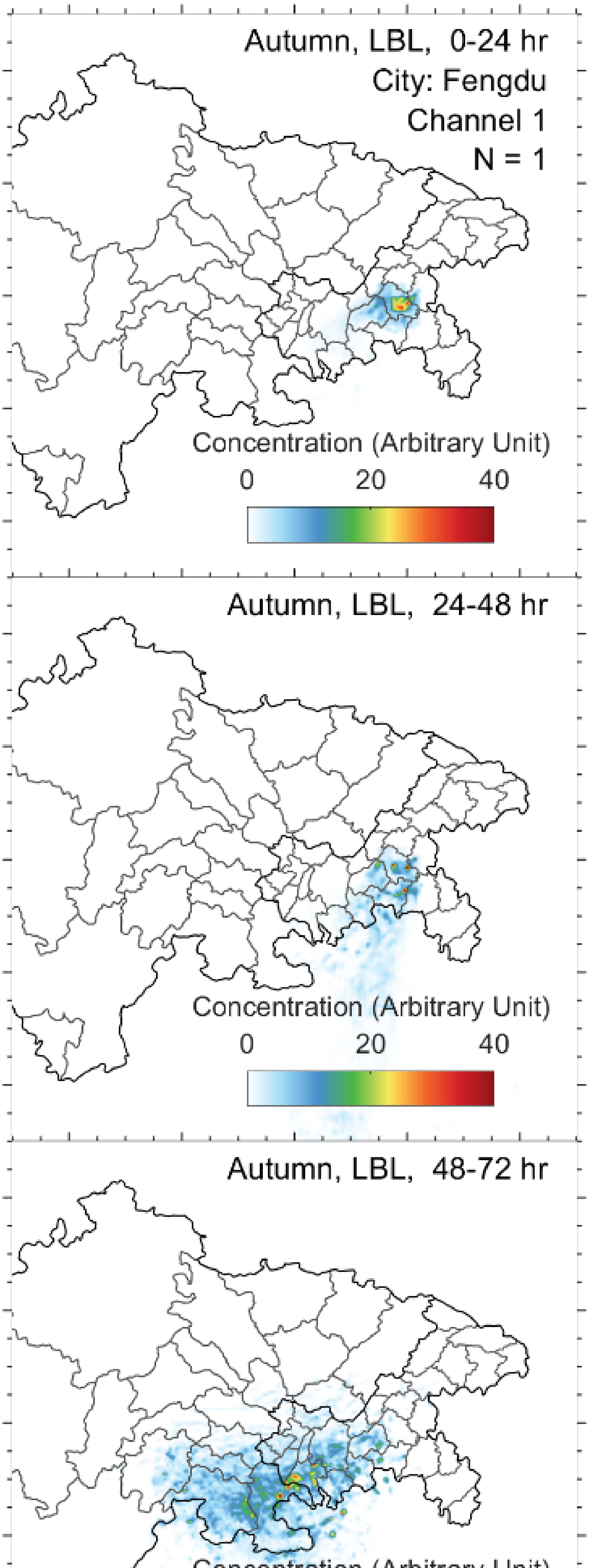
Autumn, LBL, 0-24 hr
City: Fengdu
Channel 1
N = 1
Concentration (Arbitrary Unit)
0
20
40
Autumn, LBL, 24-48 hr
Concentration (Arbitrary Unit)
0
20
40
Autumn, LBL, 48-72 hr

Figure S153 Channel 1 of Fengdu at LBL.

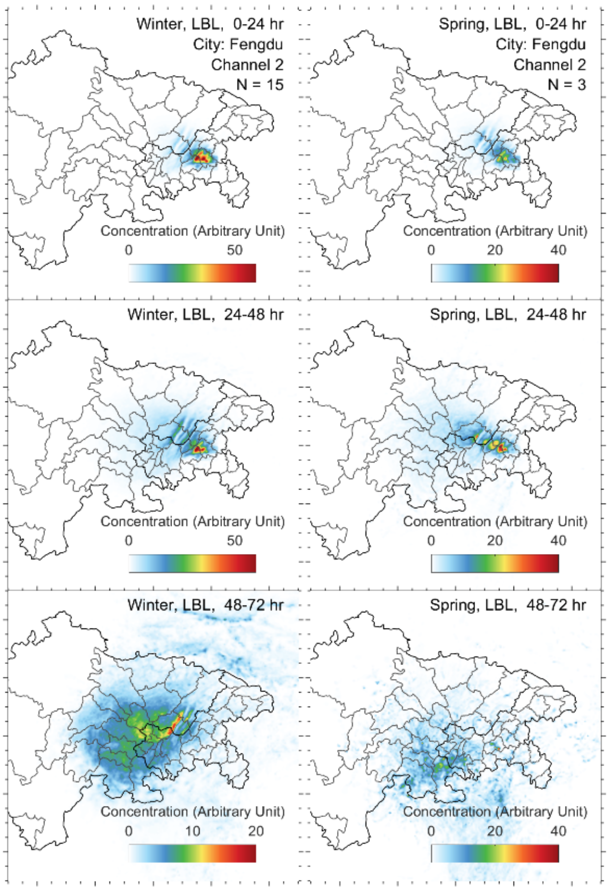

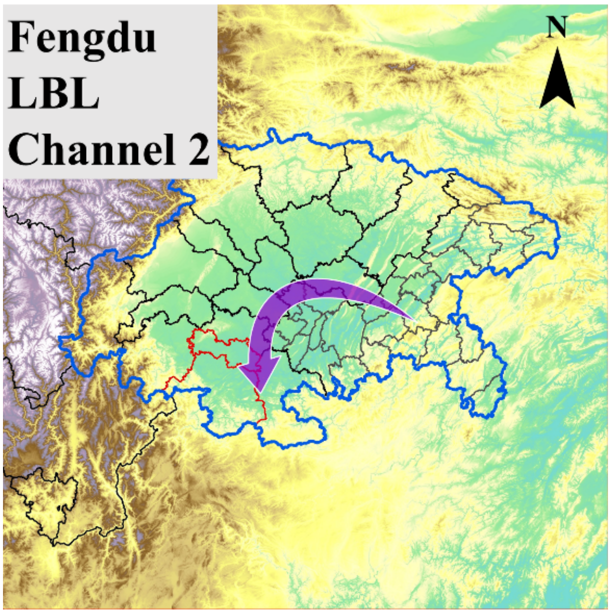

Figure S154 Channel 2 of Fengdu at LBL.

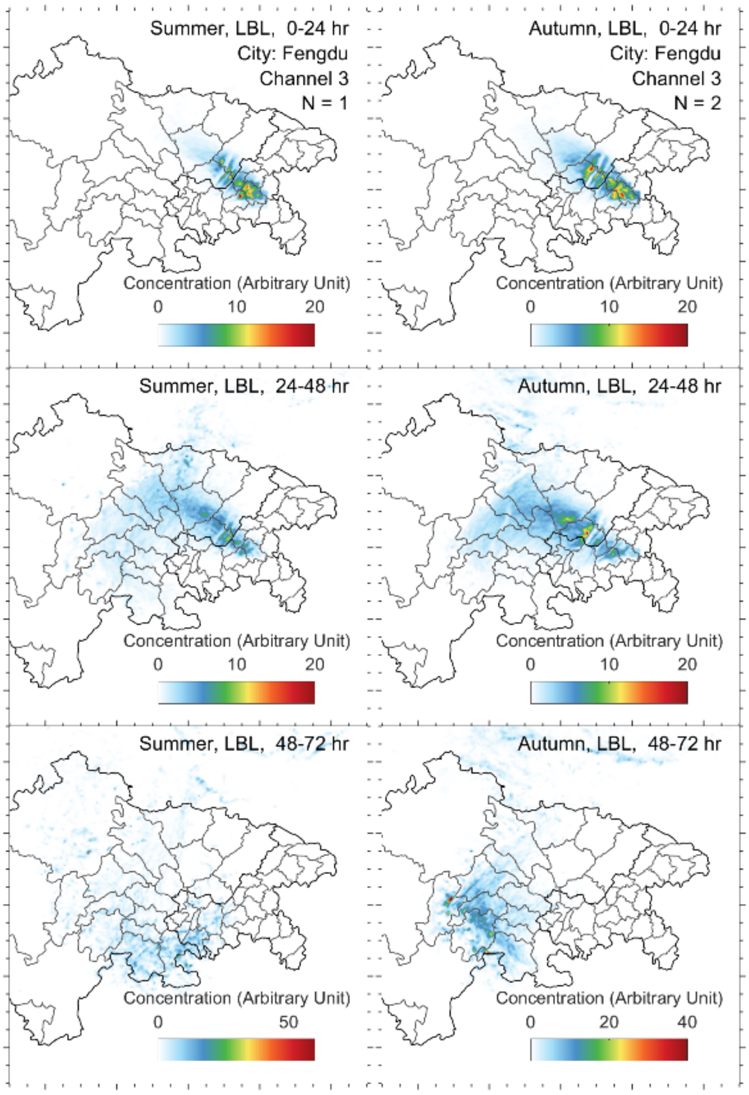
Summer, LBL, 0-24 hr
City: Fengdu
Channel 3
N = 1
Concentration (Arbitrary Unit)
0 10 20
Autumn, LBL, 0-24 hr
City: Fengdu
Channel 3
N = 2
Concentration (Arbitrary Unit)
0 10 20
Summer, LBL, 24-48 hr
Concentration (Arbitrary Unit)
0 10 20
Autumn, LBL, 24-48 hr
Concentration (Arbitrary Unit)
0 10 20
Summer, LBL, 48-72 hr
Concentration (Arbitrary Unit)
0 50
Autumn, LBL, 48-72 hr
Concentration (Arbitrary Unit)
0 20 40

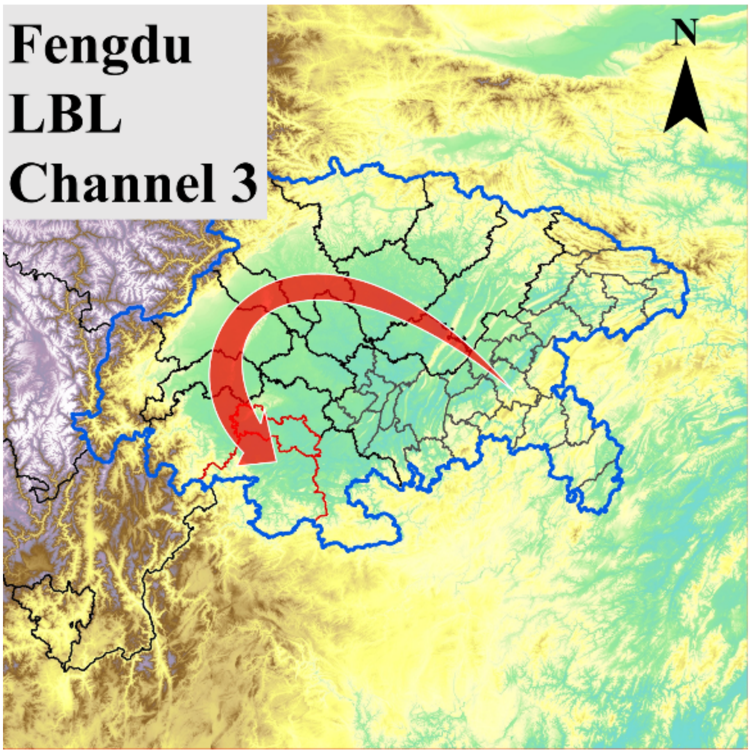
Fengdu
LBL
Channel 3
N

Figure S155 Channel 3 of Fengdu at LBL.

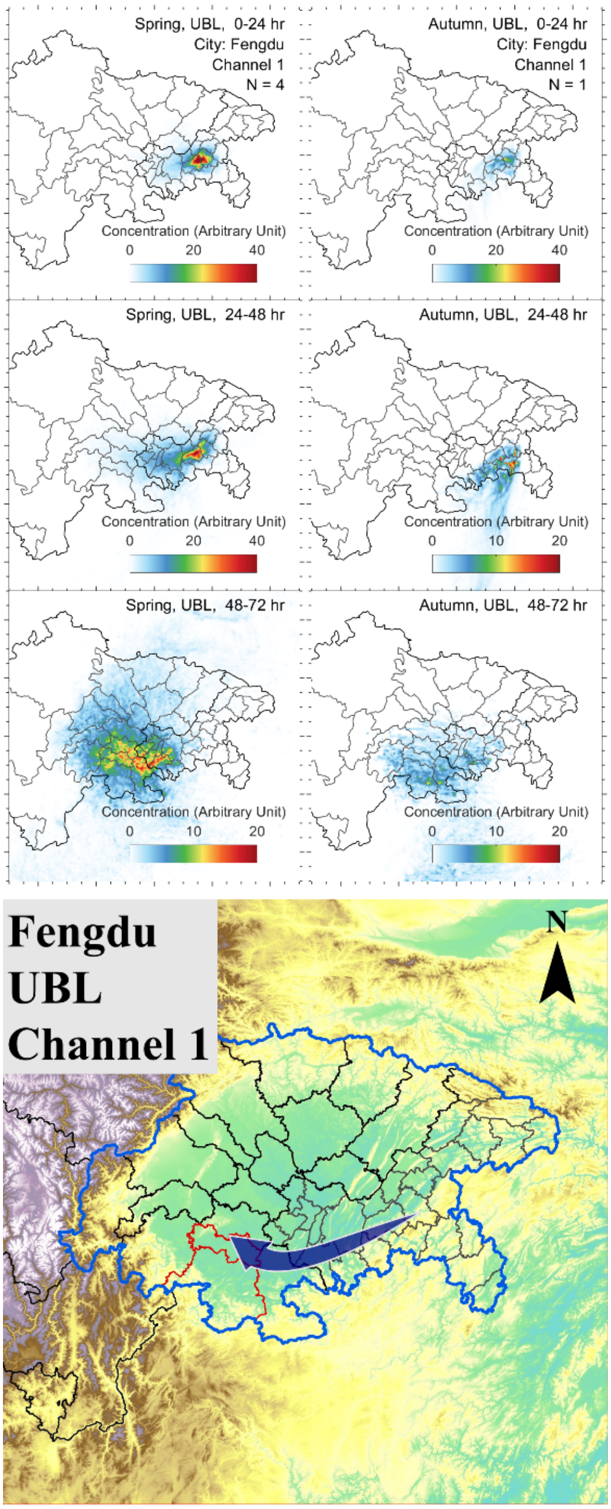

Figure S156 Channel 1 of Fengdu at UBL.

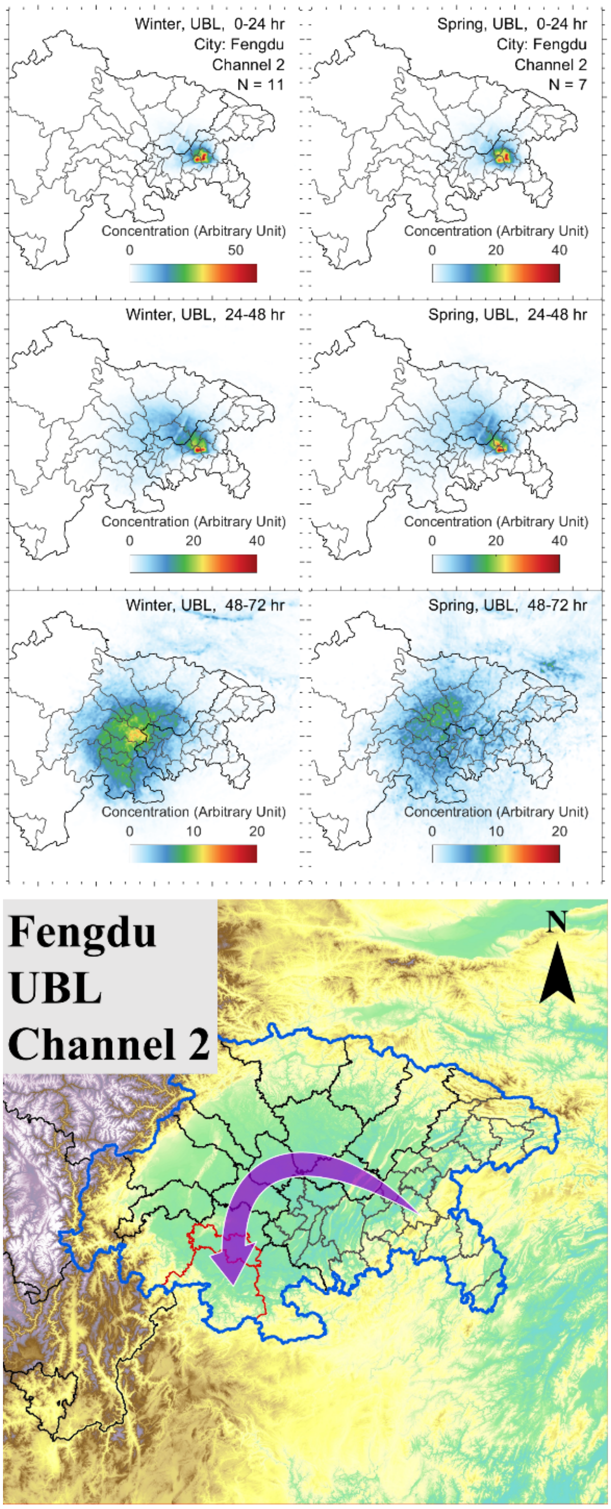

Figure S157 Channel 2 of Fengdu at UBL.

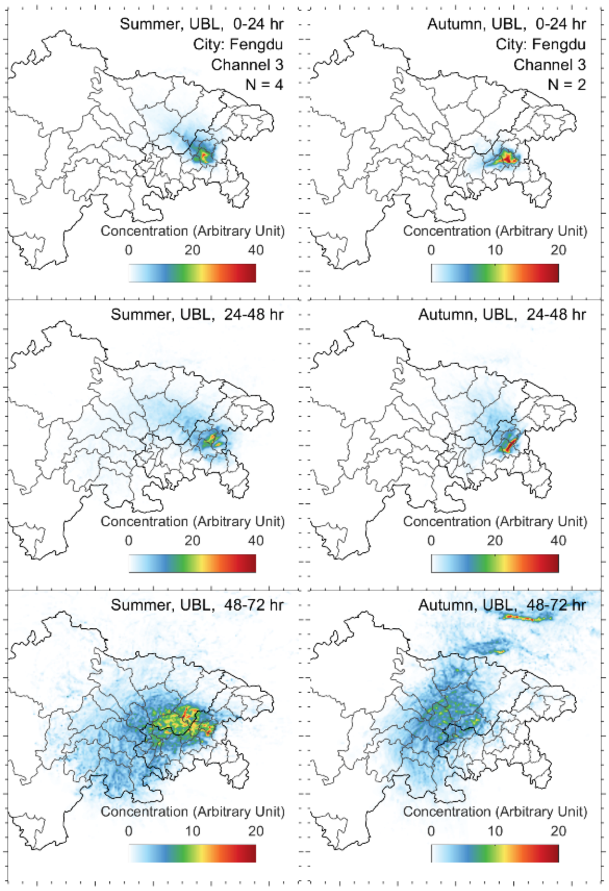

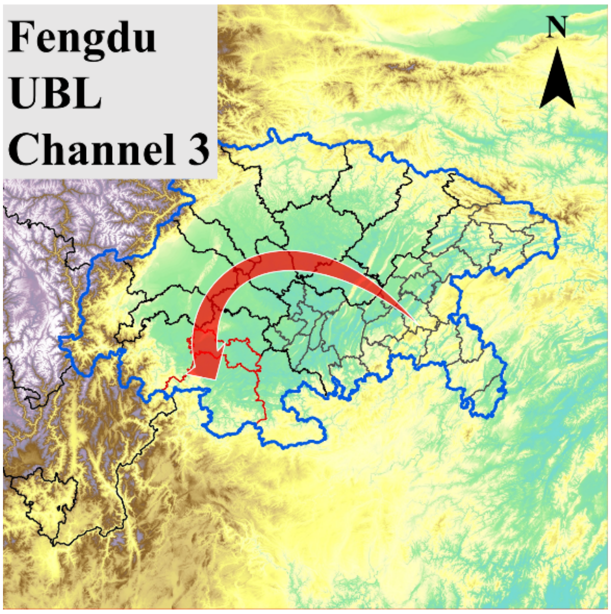

Figure S158 Channel 3 of Fengdu at UBL.

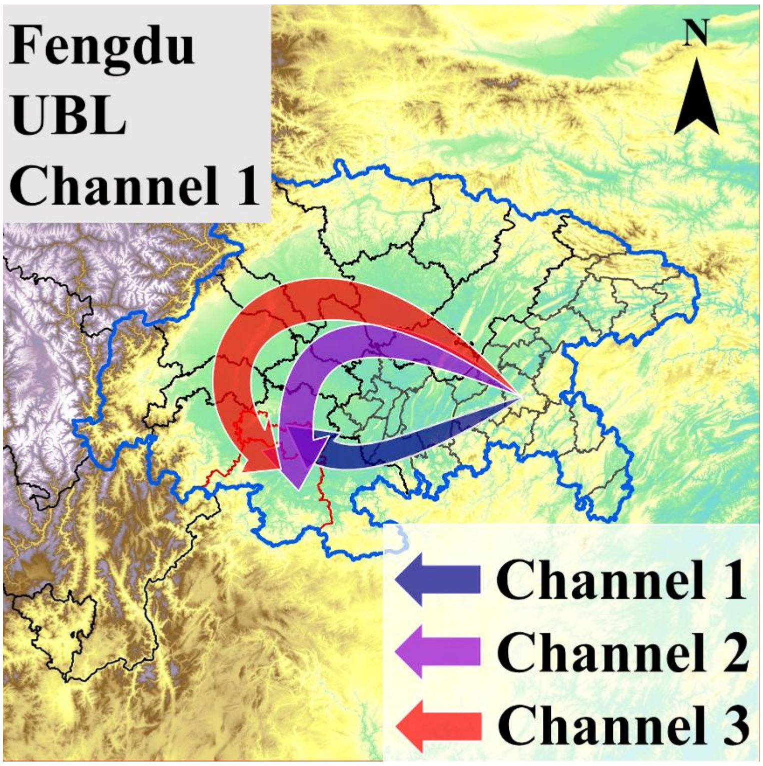

Figure S159 The identified 3 channels originating in Fengdu.

Table S29 Occurrence frequencies of each channel originating in Fengdu in four seasons.

City	Channel	Layer	Season			
			autumn	spring	summer	winter
Fengdu	1	LBL	3.2%			
		UBL	3.2%	13.3%		
	2	LBL		10.0%		48.4%
		UBL		23.3%		35.5%
	3	LBL	6.5%		3.2%	
		UBL	6.5%		12.9%	

7.6 Fuling

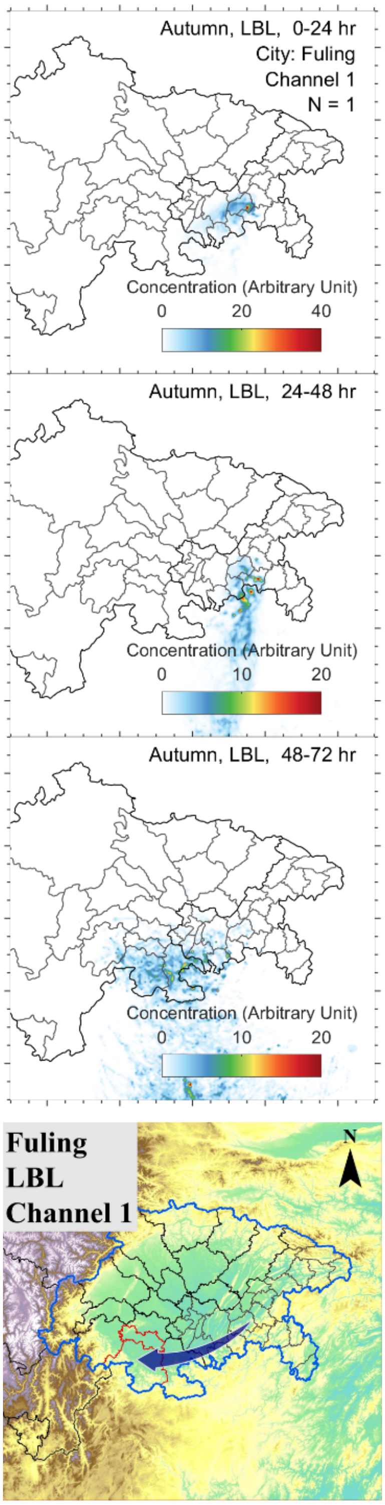

Figure S160 Channel 1 of Fuling at LBL.

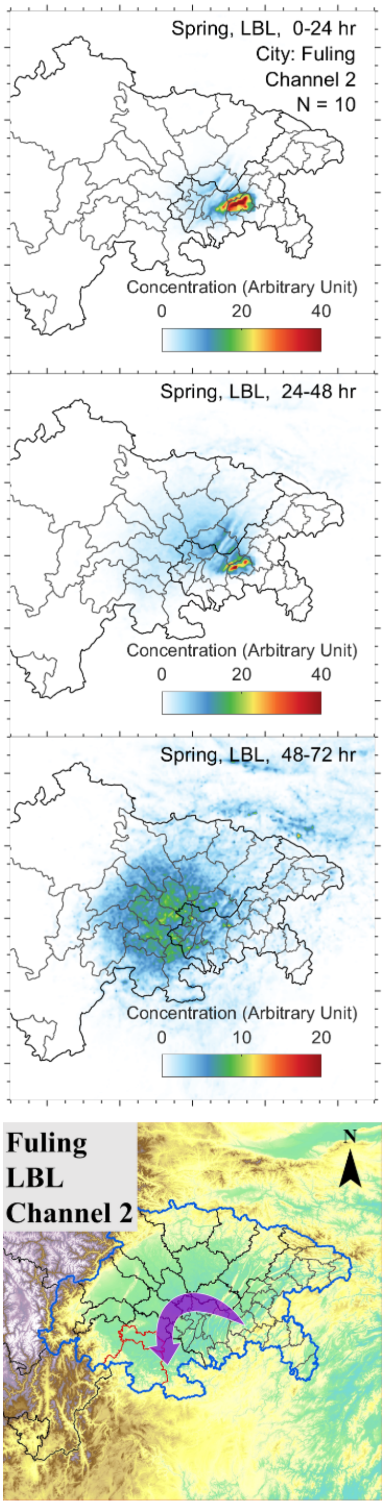

Figure S161 Channel 2 of Fuling at LBL.

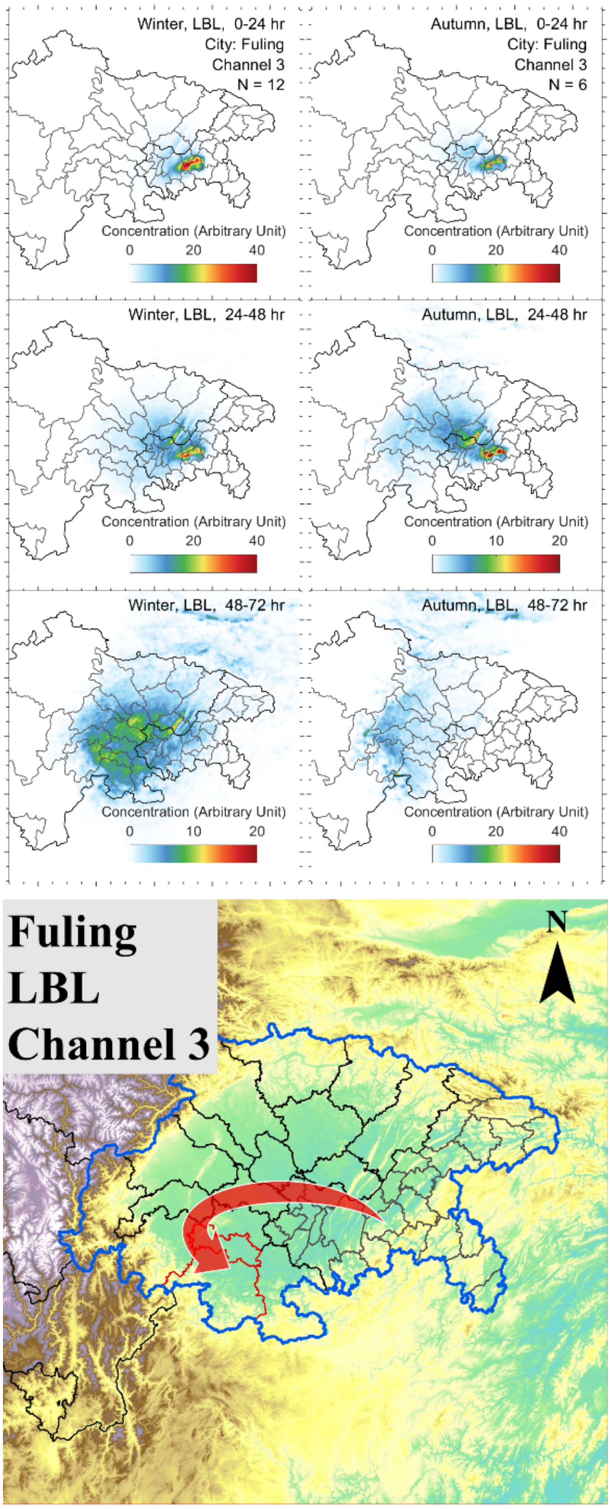

Figure S162 Channel 3 of Fuling at LBL.

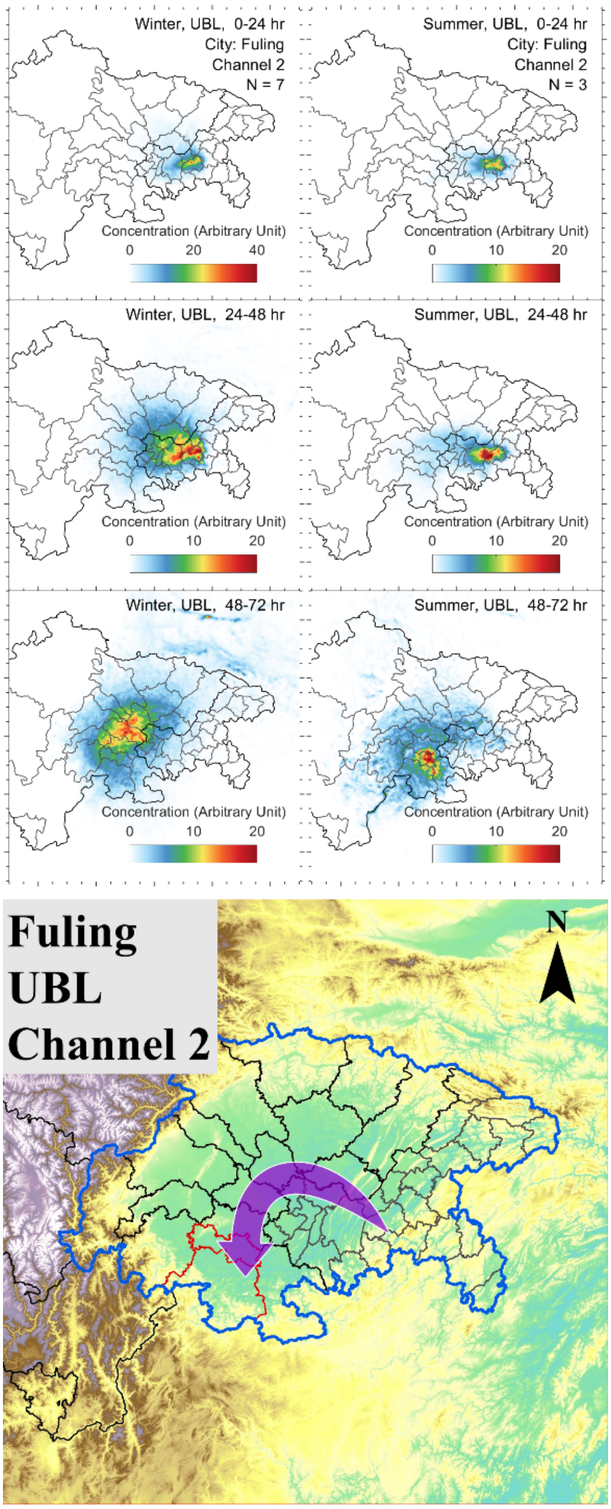

Figure S163 Channel 2 of Fuling at UBL.

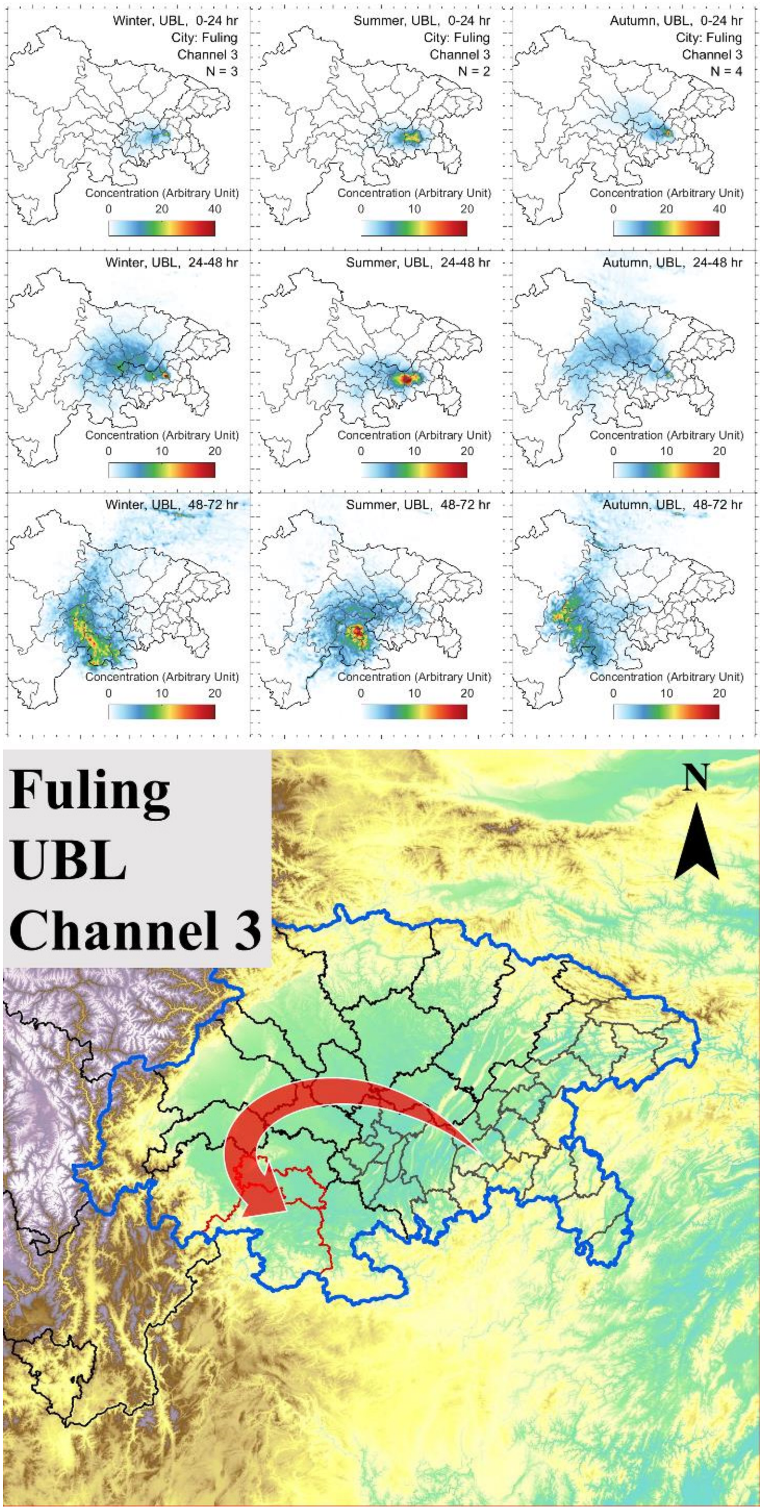

Figure S164 Channel 3 of Fuling at UBL.

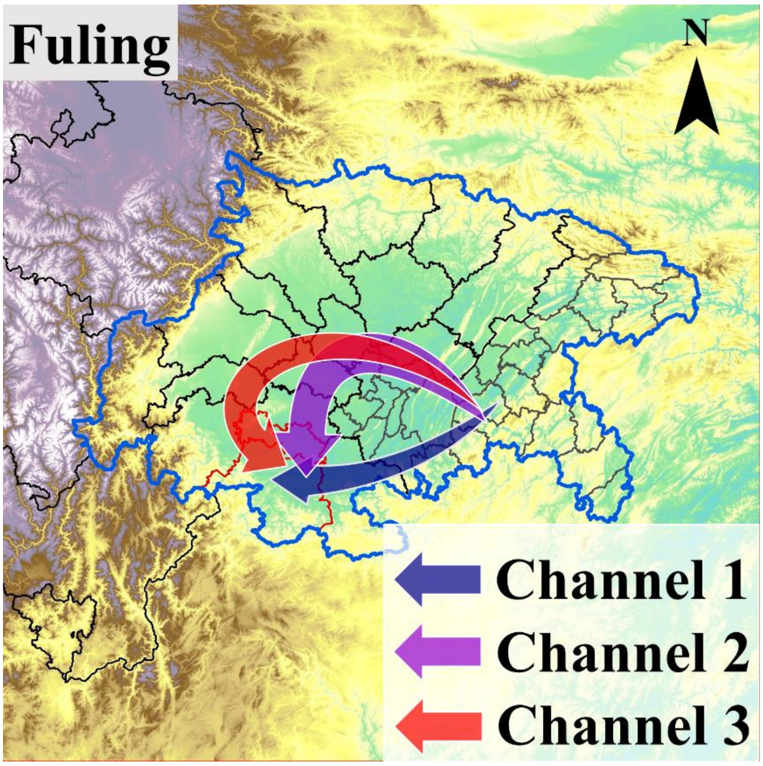

Figure S165 The identified 3 channels originating in Fuling.

Table S30 Occurrence frequencies of each channel originating in Fuling in four seasons.

City	Channel	Layer	Season			
			autumn	spring	summer	winter
Fuling	1	LBL	3.2%			
	2	LBL		33.3%		
		UBL			9.7%	22.6%
	3	LBL	19.4%			38.7%
		UBL	12.9%		6.5%	9.7%

7.7 Nanchuan

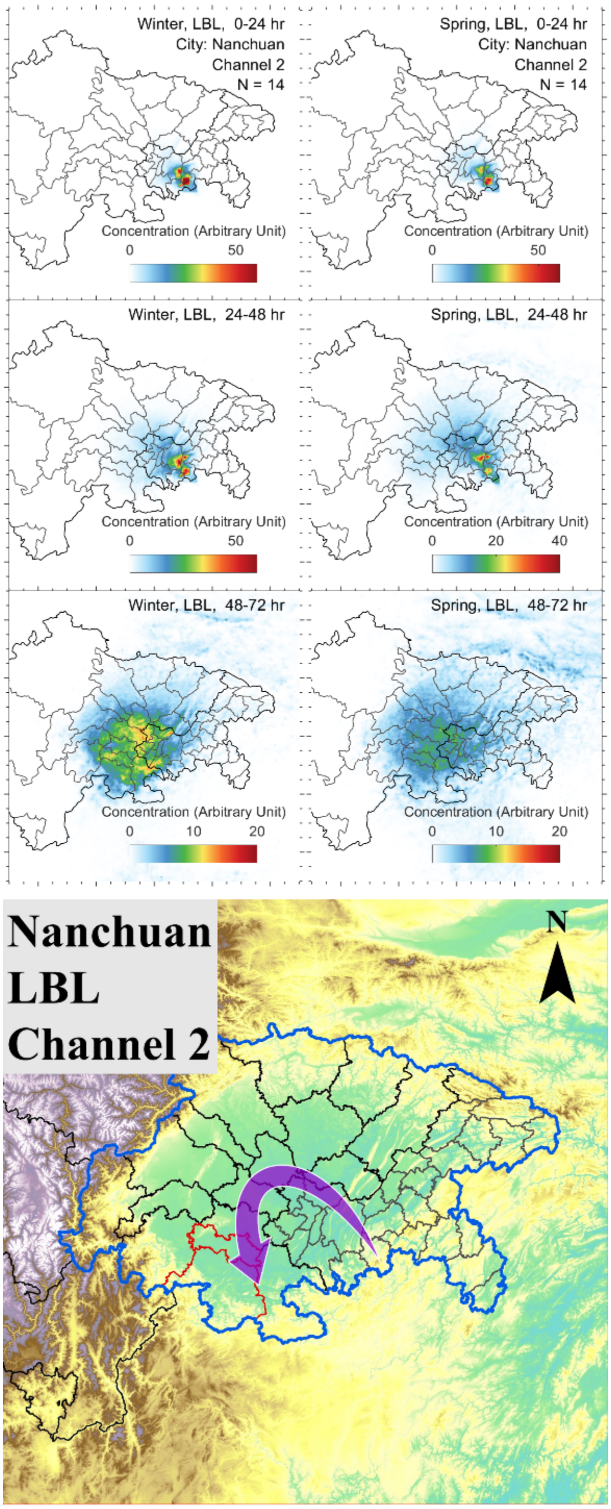

Figure S166 Channel 2 of Nanchuan at LBL.

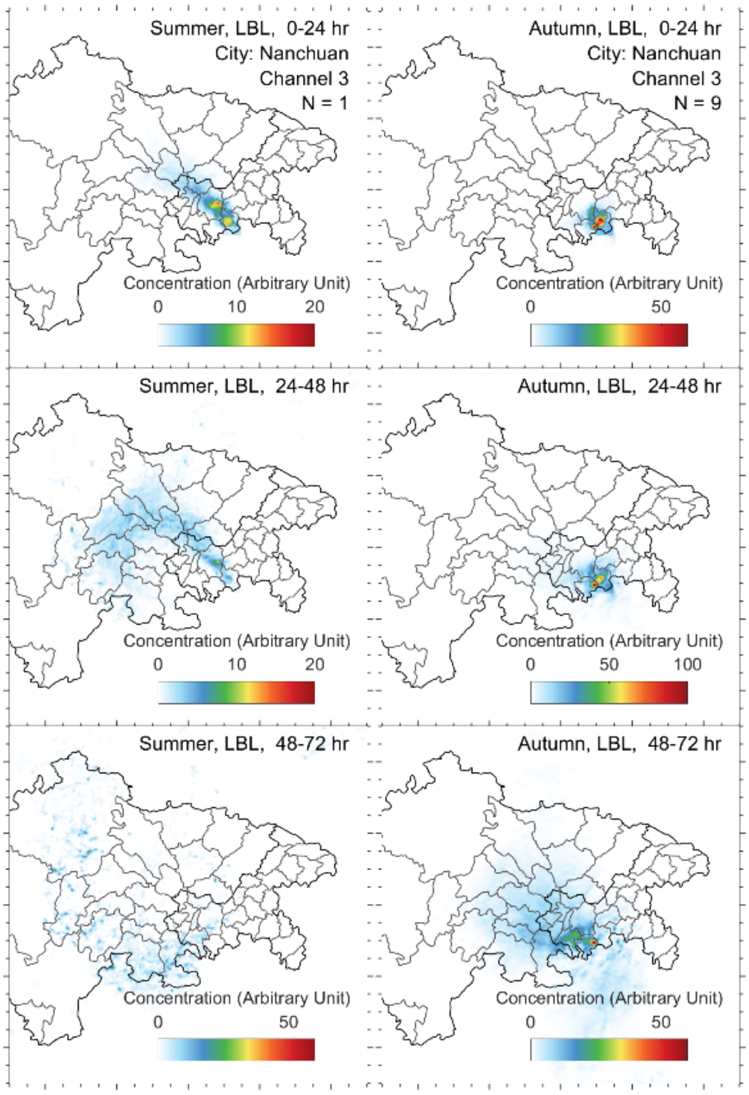
Summer, LBL, 0-24 hr
City: Nanchuan
Channel 3
N = 1
Concentration (Arbitrary Unit)
0 10 20
Autumn, LBL, 0-24 hr
City: Nanchuan
Channel 3
N = 9
Concentration (Arbitrary Unit)
0 50
Summer, LBL, 24-48 hr
Concentration (Arbitrary Unit)
0 10 20
Autumn, LBL, 24-48 hr
Concentration (Arbitrary Unit)
0 50 100
Summer, LBL, 48-72 hr
Concentration (Arbitrary Unit)
0 50
Autumn, LBL, 48-72 hr
Concentration (Arbitrary Unit)
0 50

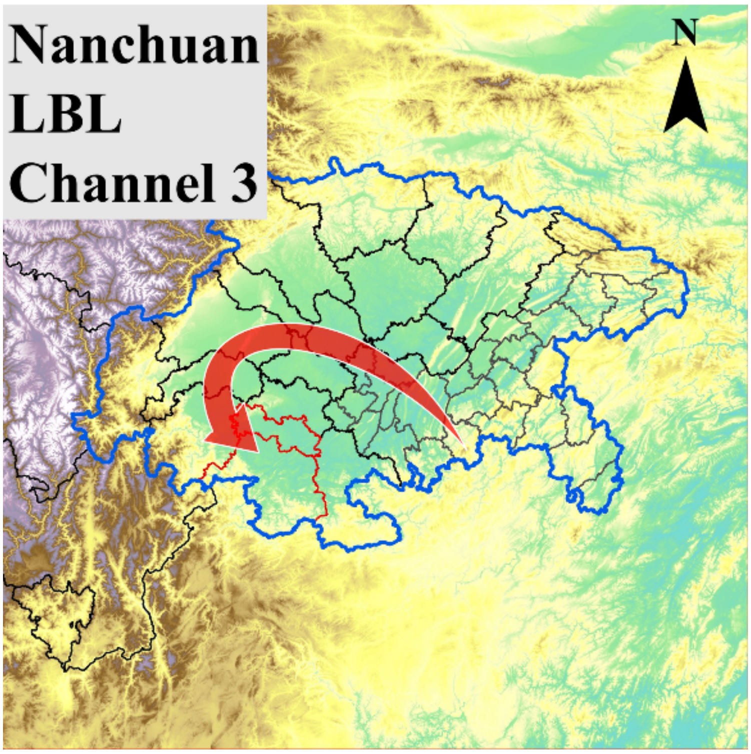
Nanchuan
LBL
Channel 3
N

Figure S167 Channel 3 of Nanchuan at LBL.

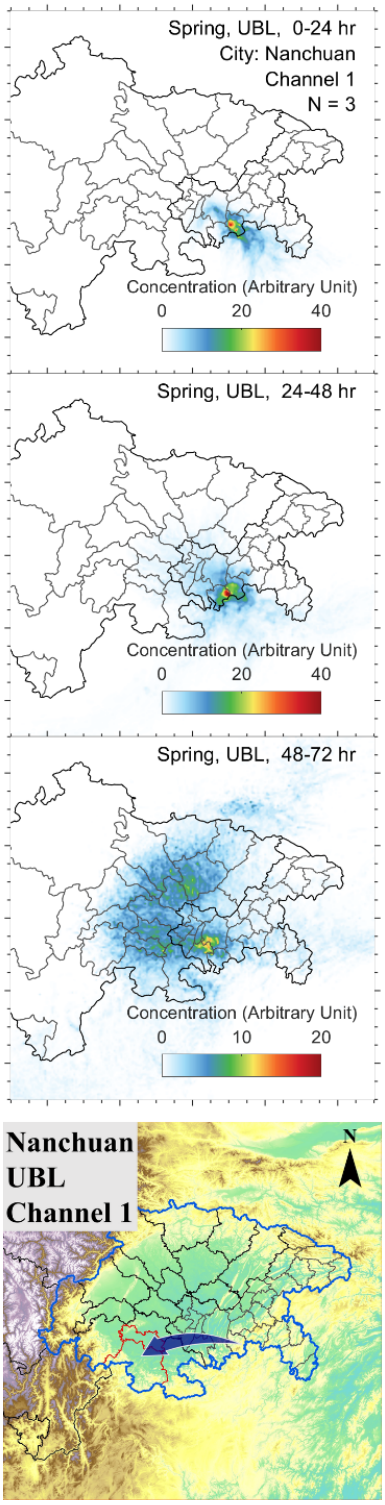

Figure S168 Channel 1 of Nanchuan at UBL.

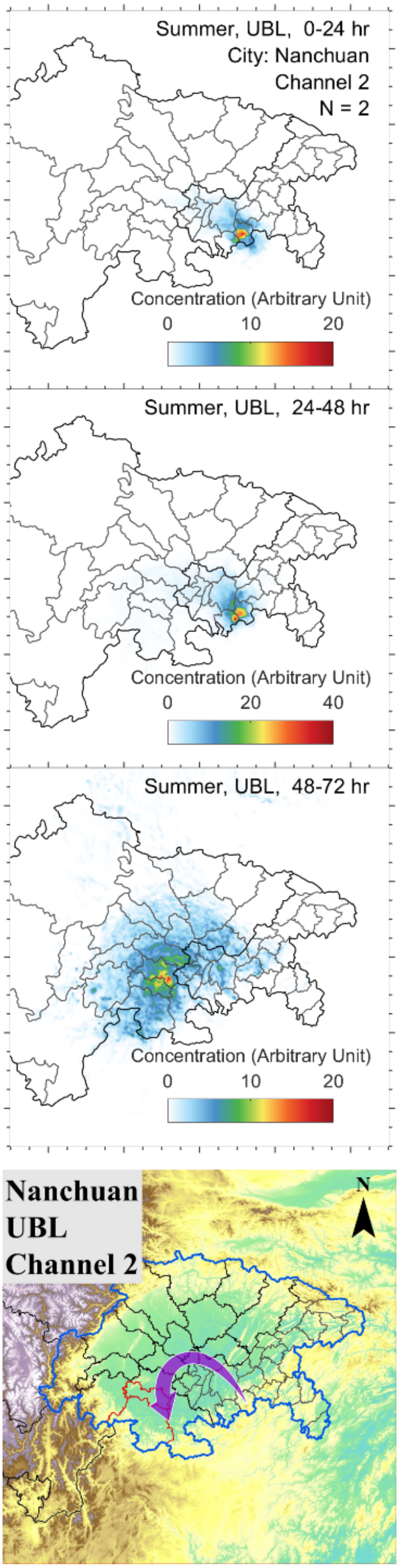

Summer, UBL, 0-24 hr
City: Nanchuan
Channel 2
N = 2
Concentration (Arbitrary Unit)
0
10
20
Summer, UBL, 24-48 hr
Concentration (Arbitrary Unit)
0
20
40
Summer, UBL, 48-72 hr
Concentration (Arbitrary Unit)
0
10
20
Nanchuan
UBL
Channel 2
N

Figure S169 Channel 2 of Nanchuan at UBL.

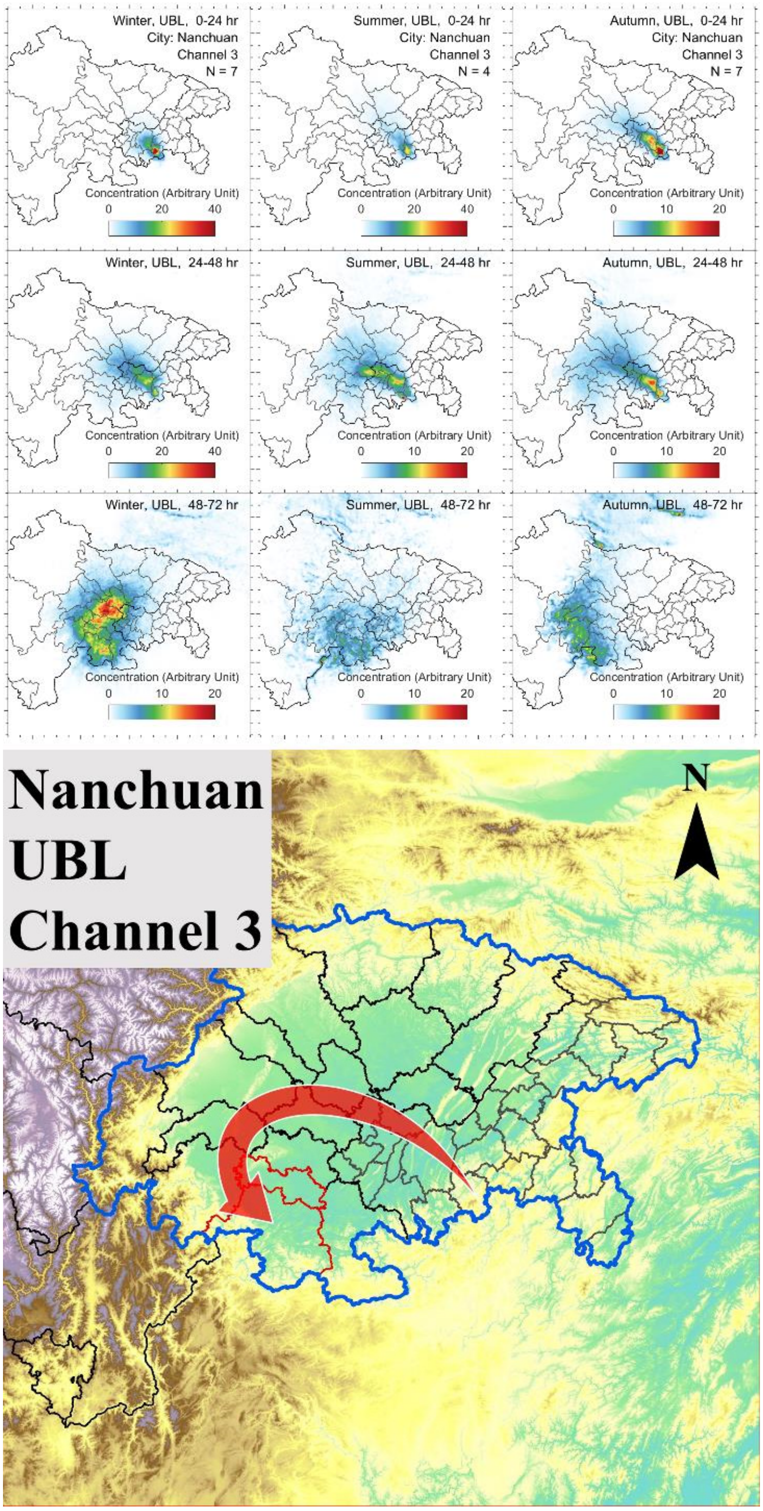

Figure S170 Channel 3 of Nanchuan at UBL.

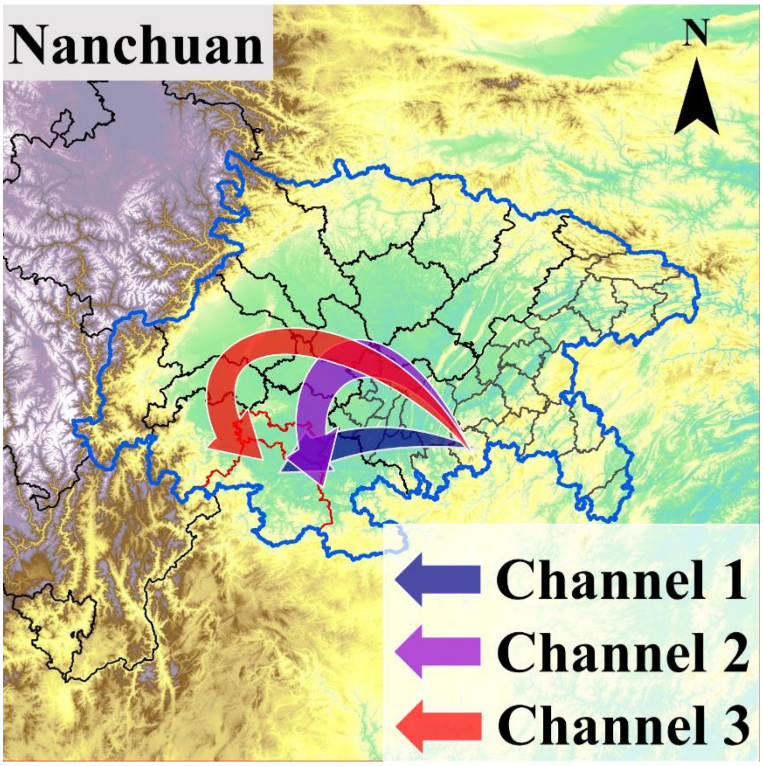

Figure S171 The identified 3 channels originating in Nanchuan.

Table S31 Occurrence frequencies of each channel originating in Nanchuan in four seasons.

City	Channel	Layer	Season			
			autumn	spring	summer	winter
Nanchuan	1	UBL		10.0%		
	2	LBL		46.7%		45.2%
		UBL			6.5%	
	3	LBL	29.0%		3.2%	
		UBL	22.6%		12.9%	22.6%

8. Southeast Chongqing Urban Agglomeration

8.1 Wulong

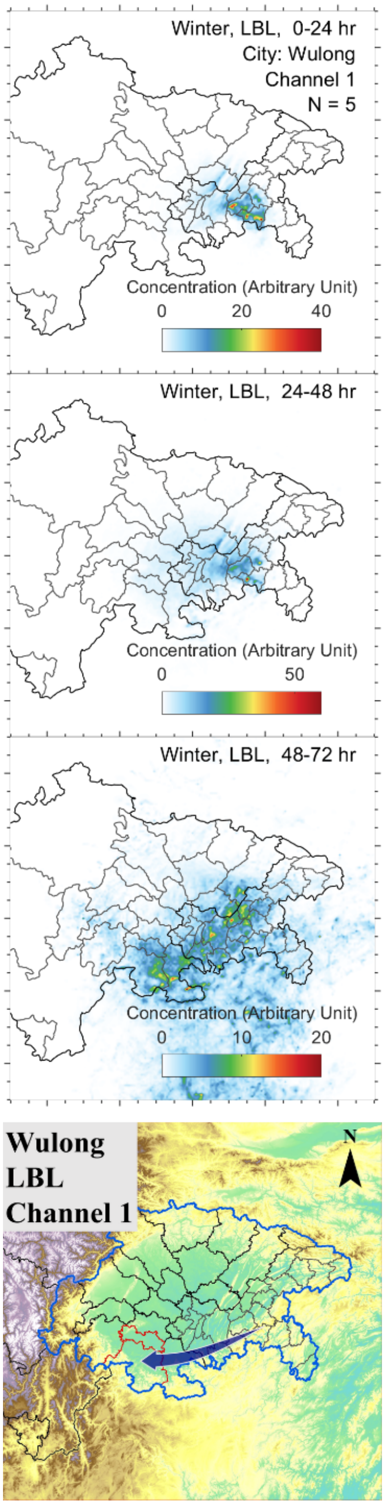

Figure S172 Channel 1 of Wulong at LBL.

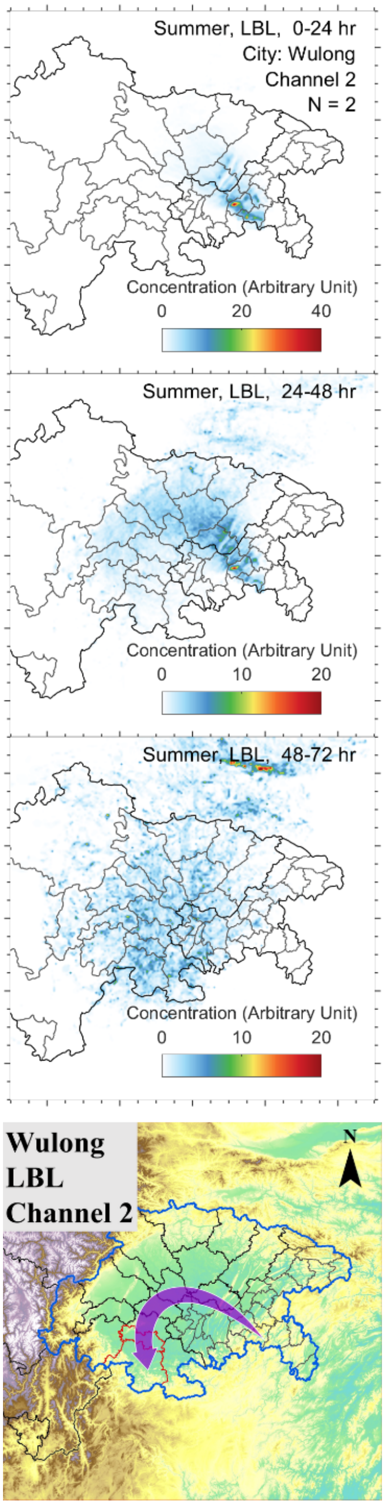

Figure S173 Channel 2 of Wulong at LBL.73

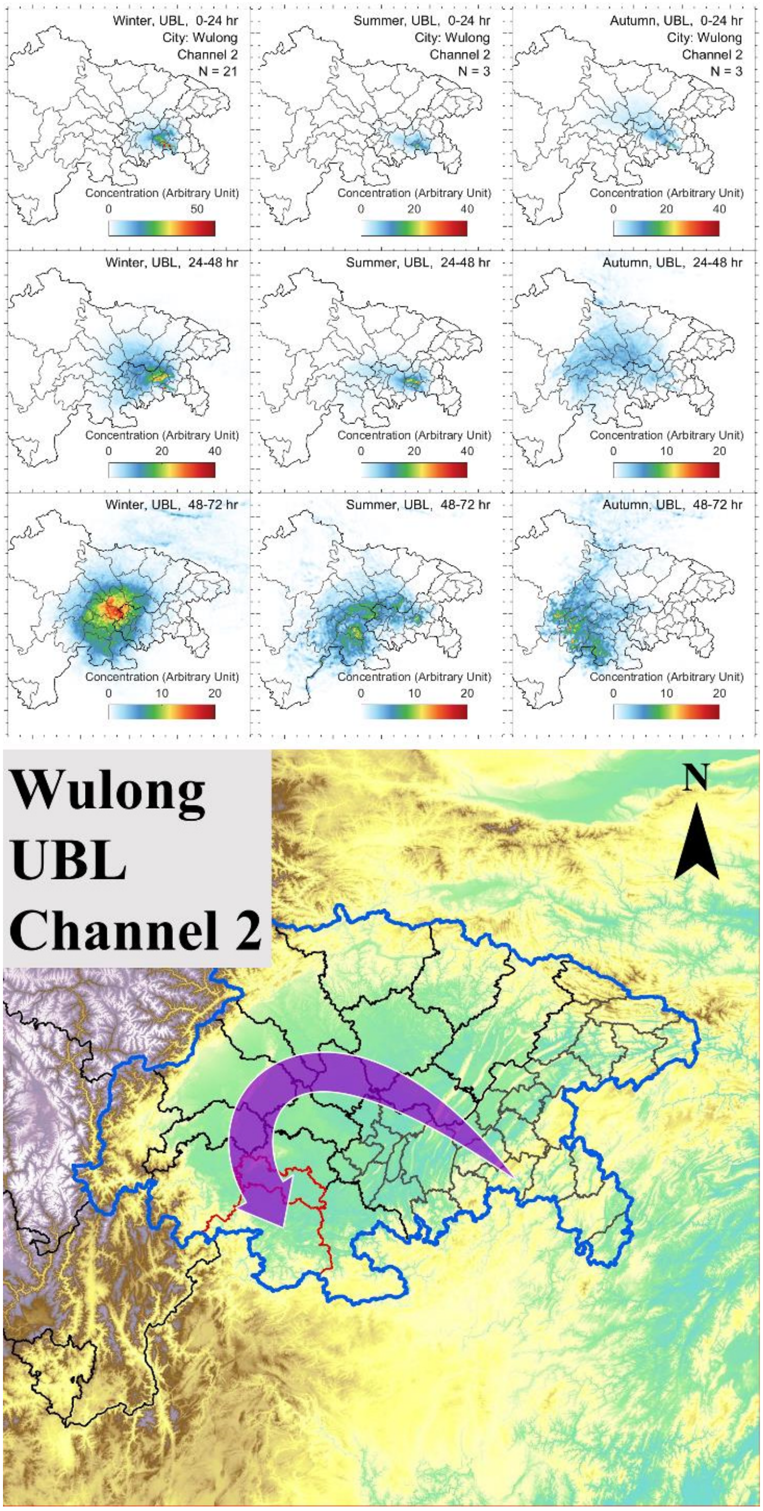

Figure S174 Channel 2 of Wulong at UBL.

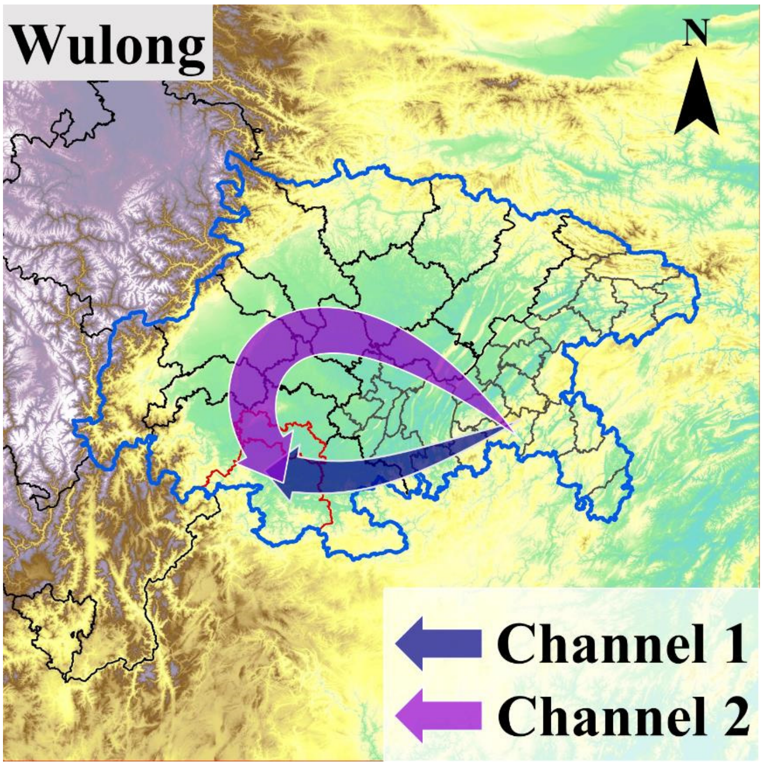

Figure S175 The identified 2 channels originating in Wulong.

Table S32 Occurrence frequencies of each channel originating in Wulong in four seasons.

City	Channel	Layer	Season			
			autumn	spring	summer	winter
Wulong	1	LBL				16.1%
	2	LBL			6.5%	
		UBL	9.7%		9.7%	67.7%

8.2 Pengshui

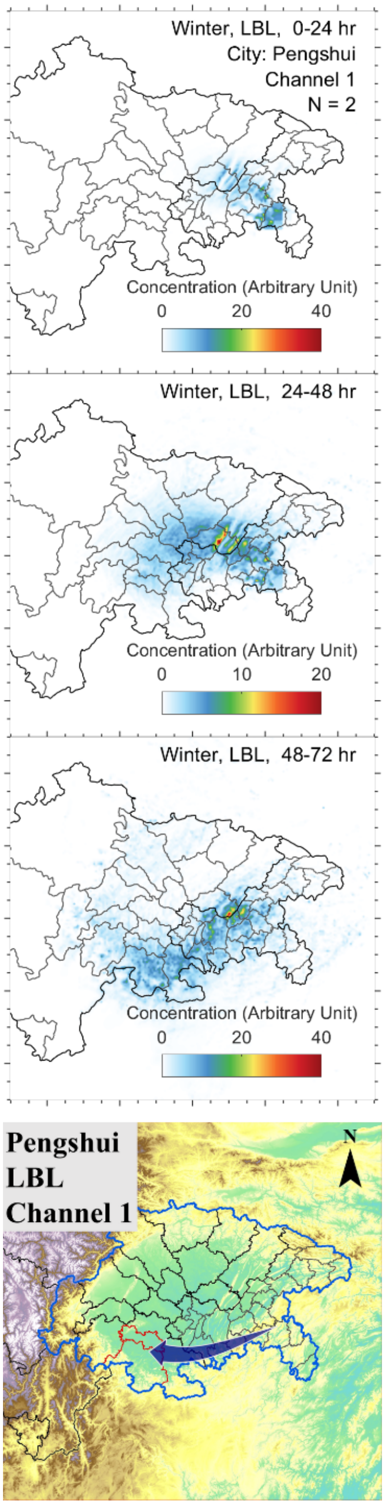

Figure S176 Channel 1 of Pengshui at LBL.

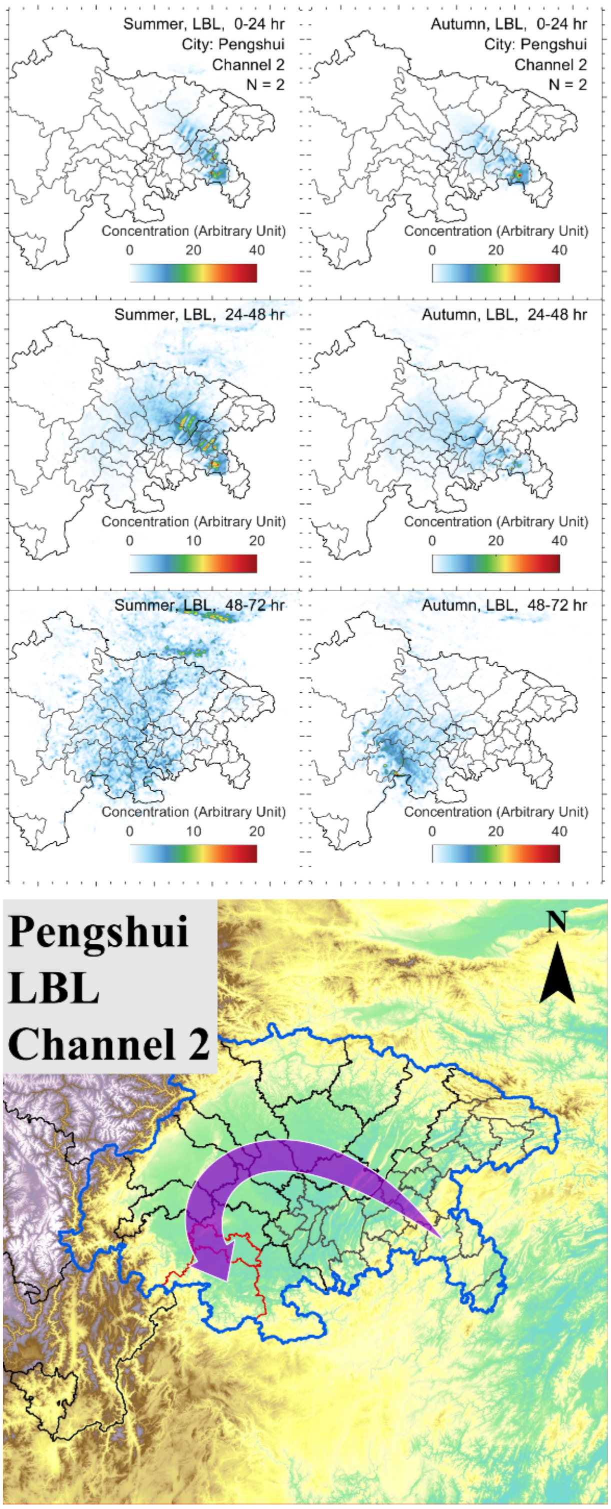

Figure S177 Channel 2 of Pengshui at LBL.

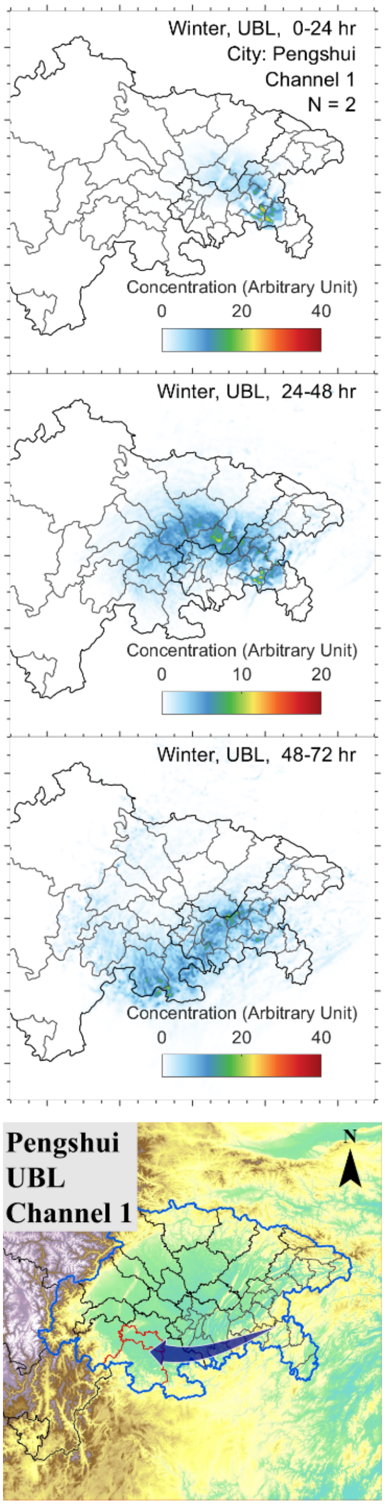

Figure S178 Channel 1 of Pengshui at UBL.

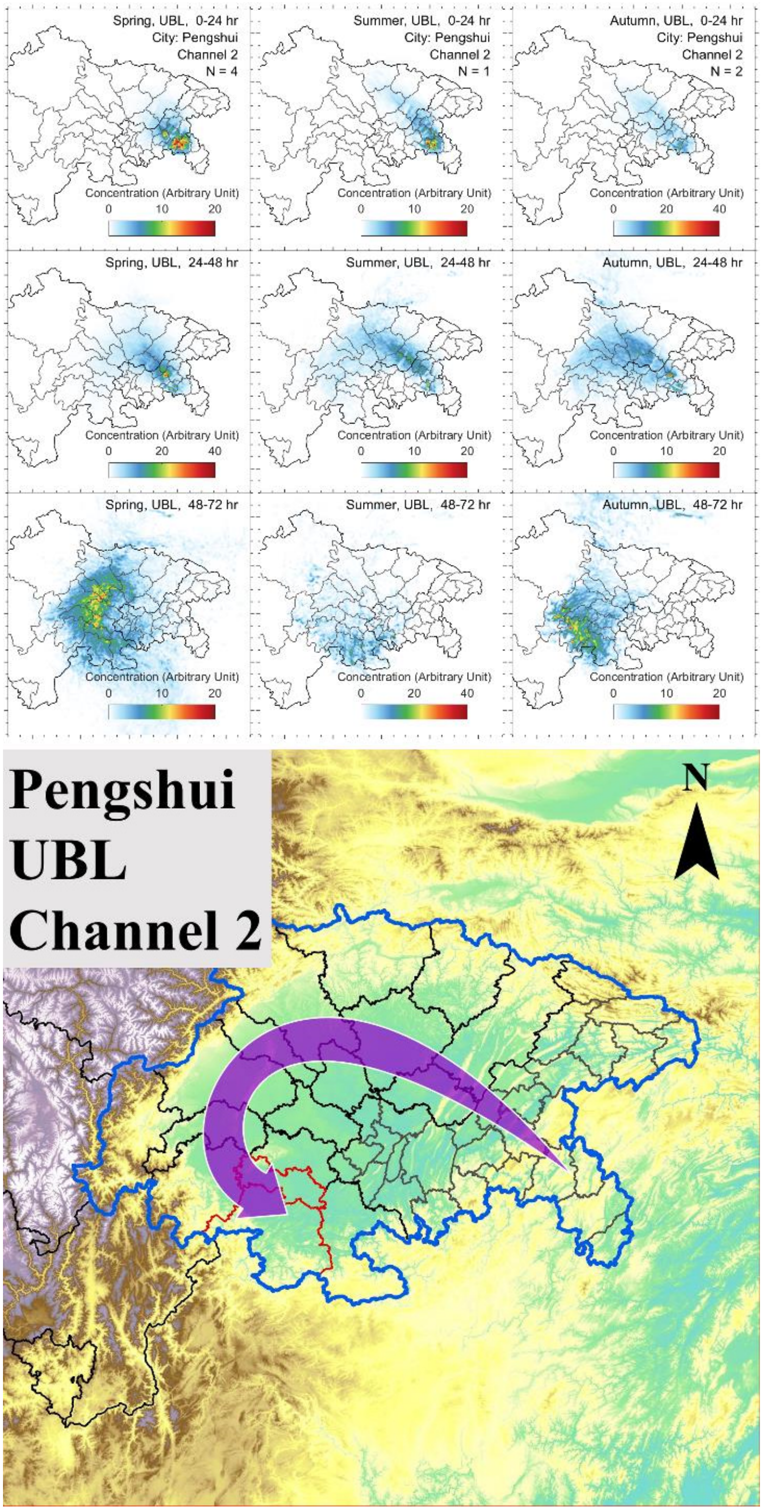

Figure S179 Channel 2 of Pengshui at UBL.

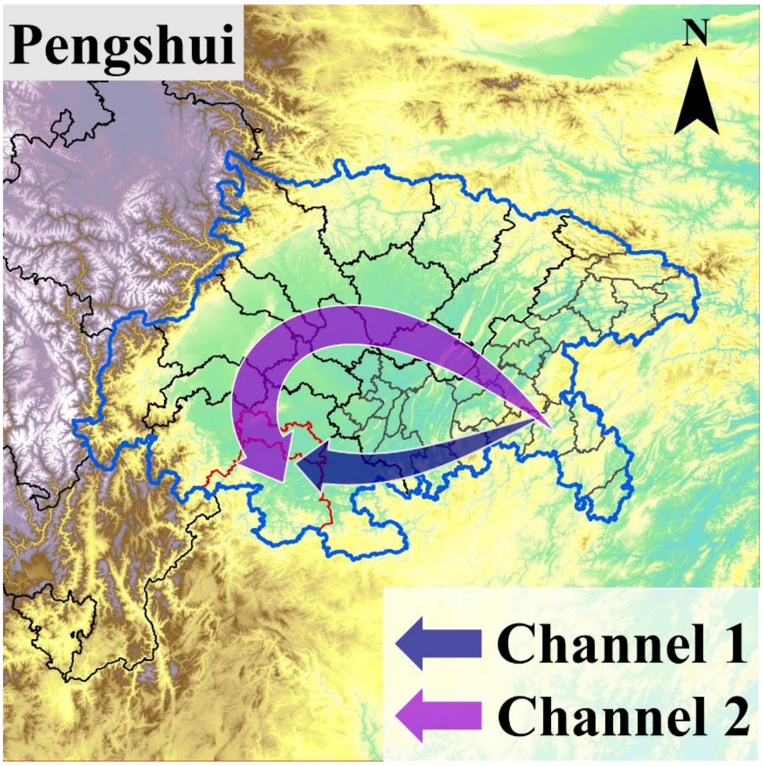

Figure S180 The identified 2 channels originating in Pengshui.

Table S33 Occurrence frequencies of each channel originating in Pengshui in four seasons.

City	Channel	Layer	Season			
			autumn	spring	summer	winter
Pengshui	1	LBL				6.5%
		UBL				6.5%
	2	LBL	6.5%		6.5%	
		UBL	6.5%	13.3%	3.2%	

8.3 Qianjiang

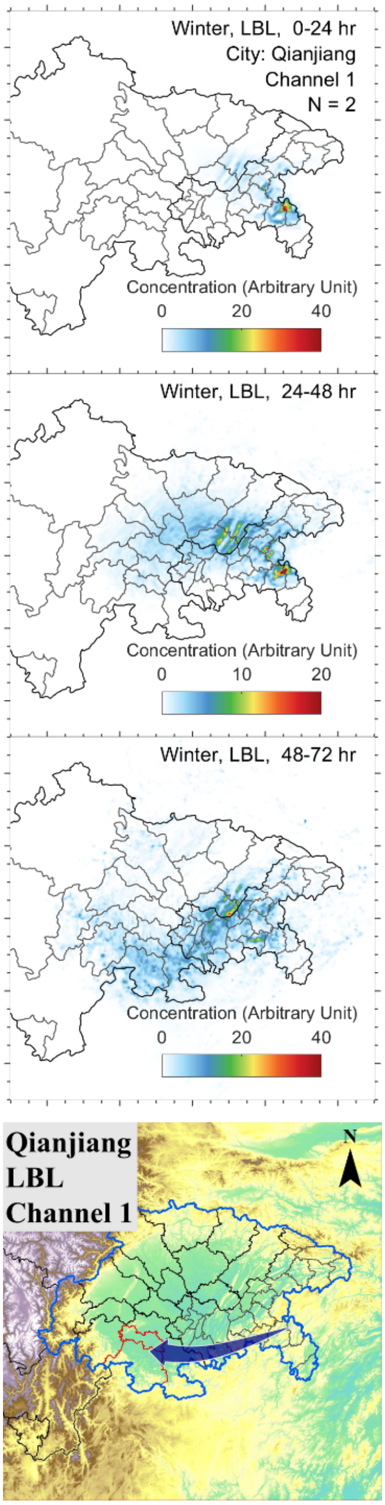

Figure S181 Channel 1 of Qianjiang at LBL.

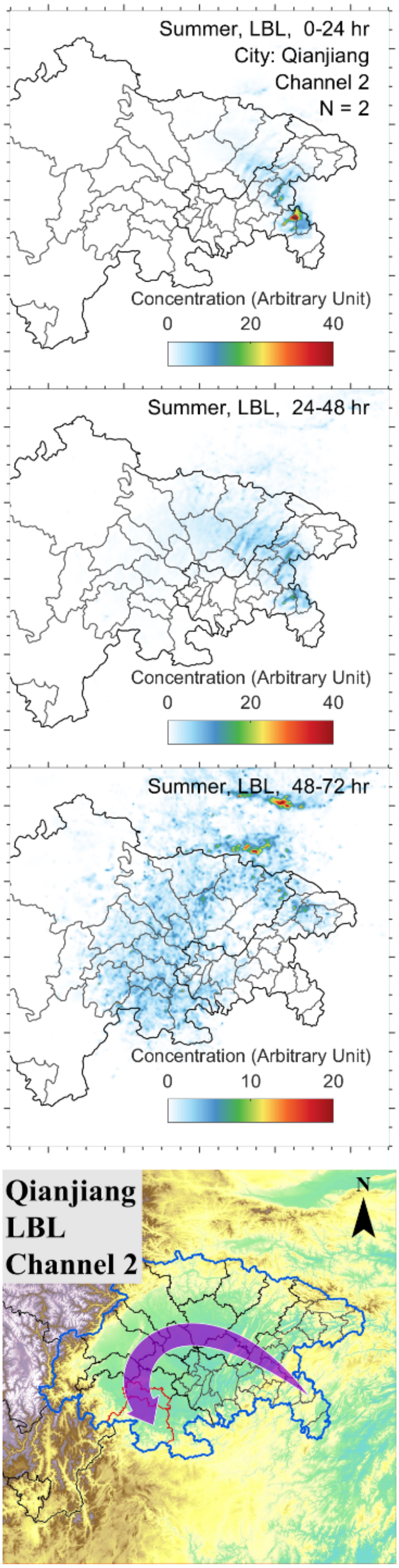

Summer, LBL, 0-24 hr
City: Qianjiang
Channel 2
N = 2
Concentration (Arbitrary Unit)
0 20 40
Summer, LBL, 24-48 hr
Concentration (Arbitrary Unit)
0 20 40
Summer, LBL, 48-72 hr
Concentration (Arbitrary Unit)
0 10 20
Qianjiang
LBL
Channel 2
N

Figure S182 Channel 2 of Qianjiang at LBL.

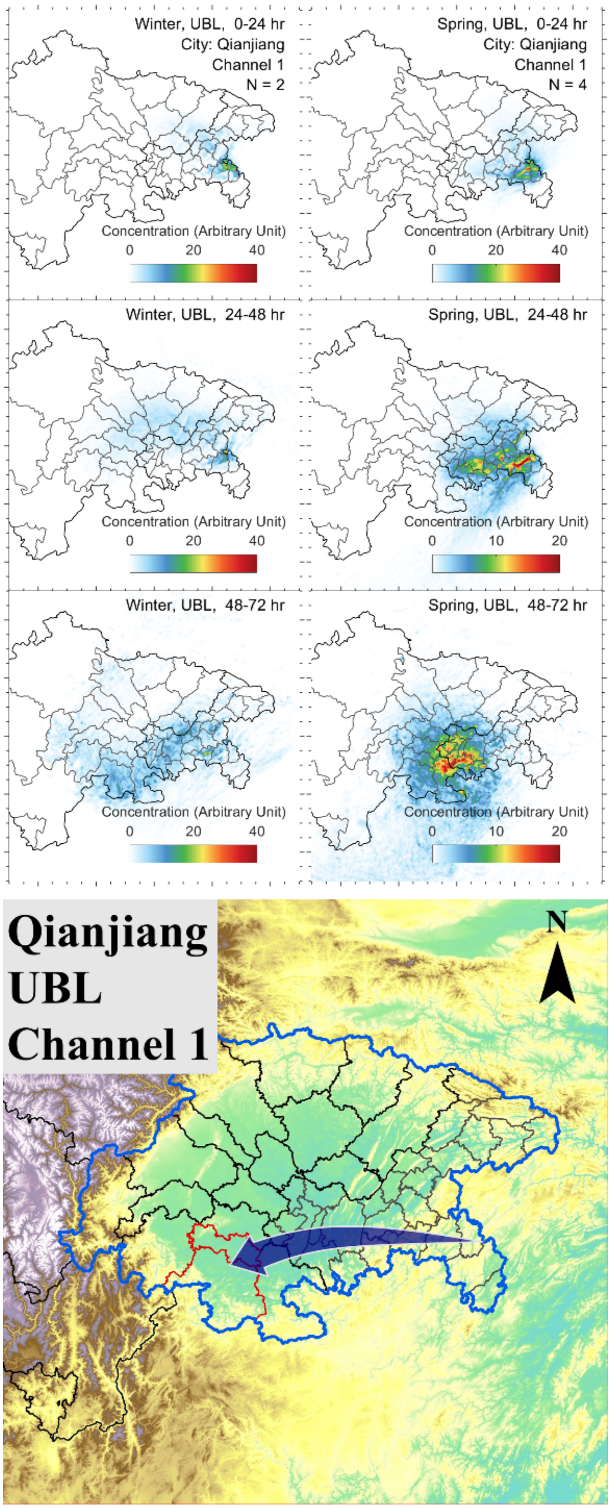

Figure S183 Channel 1 of Qianjiang at UBL.

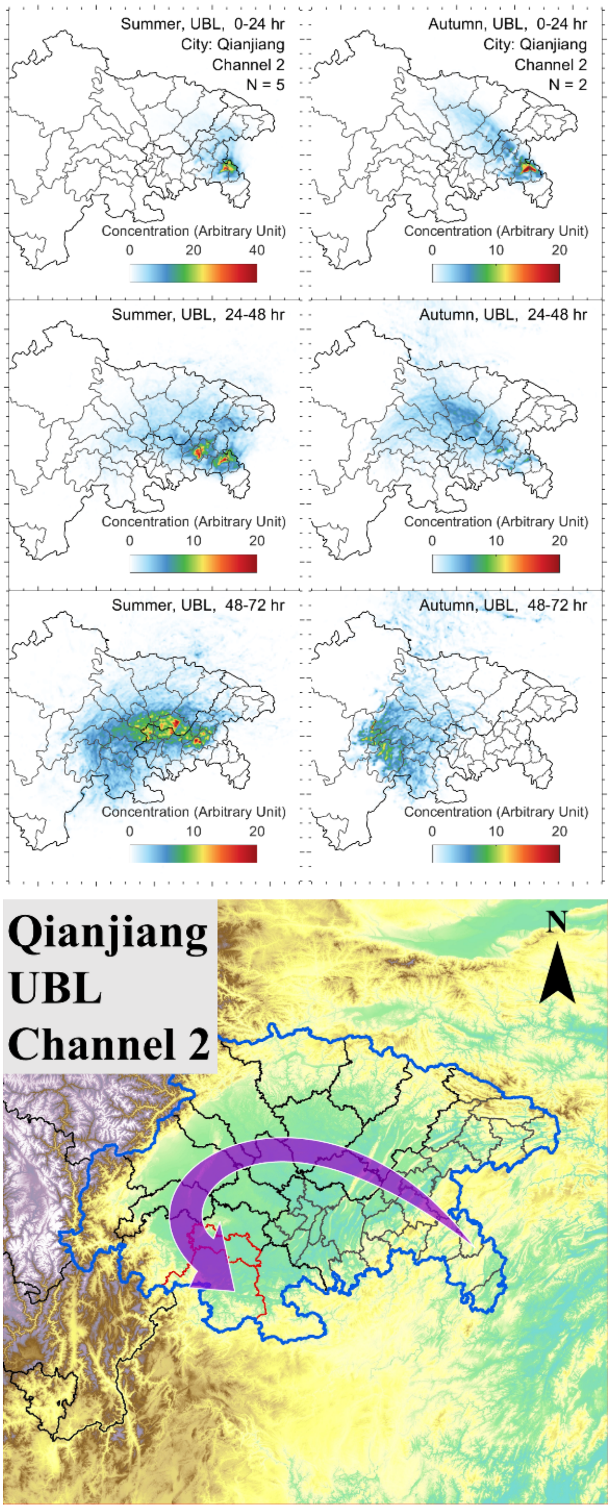

Figure S184 Channel 2 of Qianjiang at UBL.

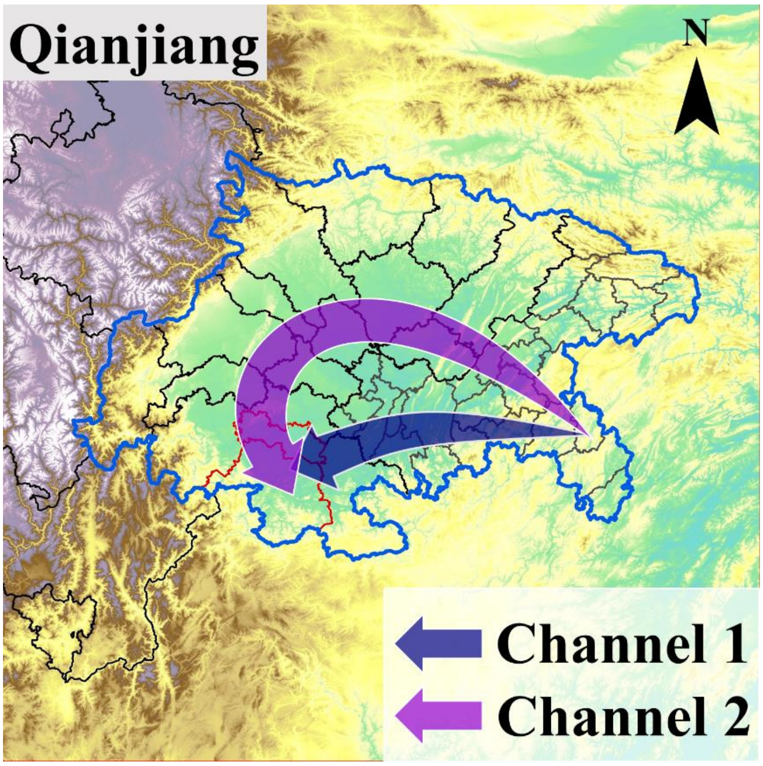

Figure S185 The identified 2 channels originating in Qianjiang.

Table S34 Occurrence frequencies of each channel originating in Qianjiang in four seasons.

City	Channel	Layer	Season			
			autumn	spring	summer	winter
Qianjiang	1	LBL				6.5%
		UBL		13.3%		6.5%
	2	LBL			6.5%	
		UBL	6.5%		16.1%	

8.4 Shizhu

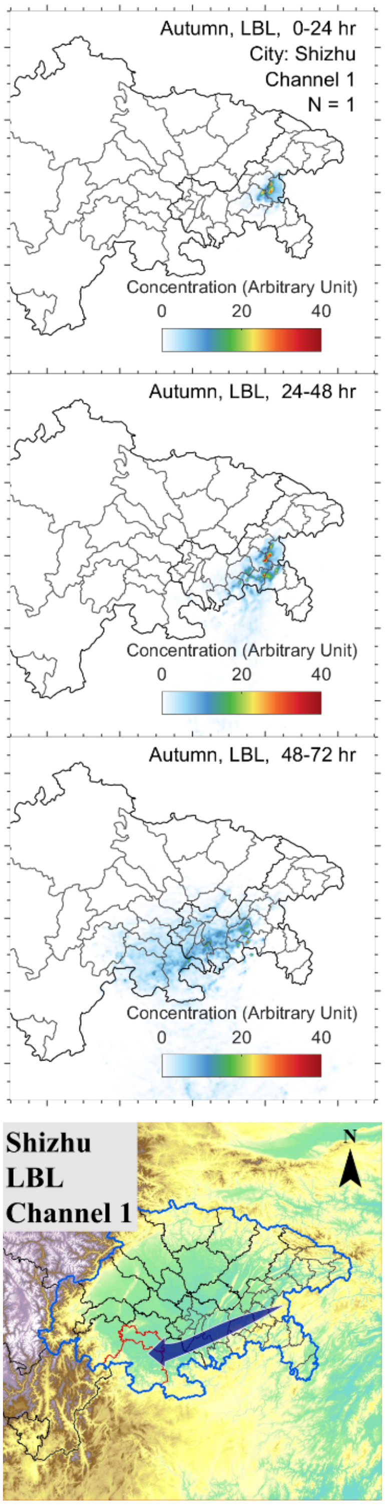

Figure S186 Channel 1 of Shizhu at LBL.

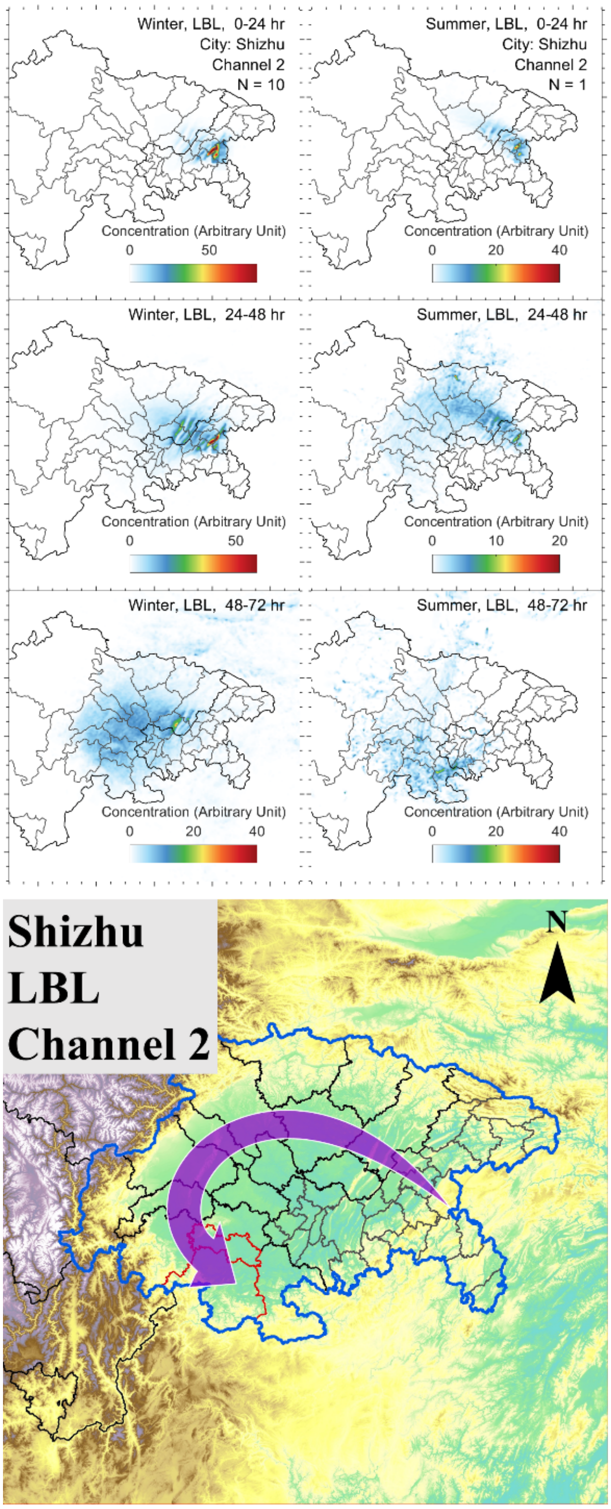

Figure S187 Channel 2 of Shizhu at LBL.

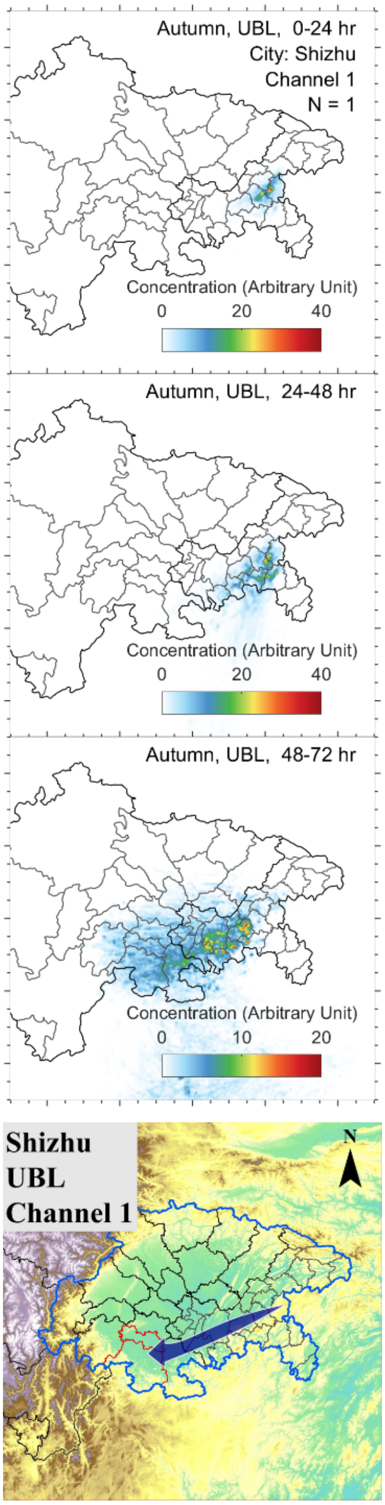

Figure S188 Channel 1 of Shizhu at UBL.

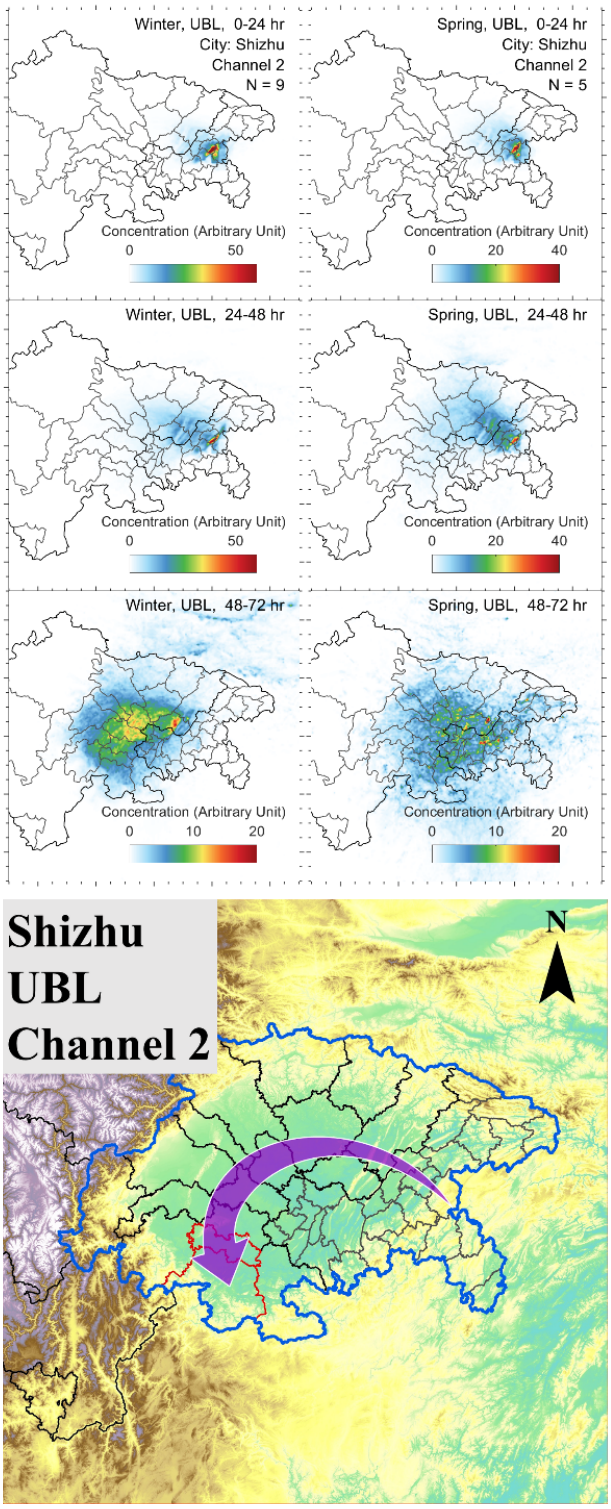

Figure S189 Channel 2 of Shizhu at UBL.

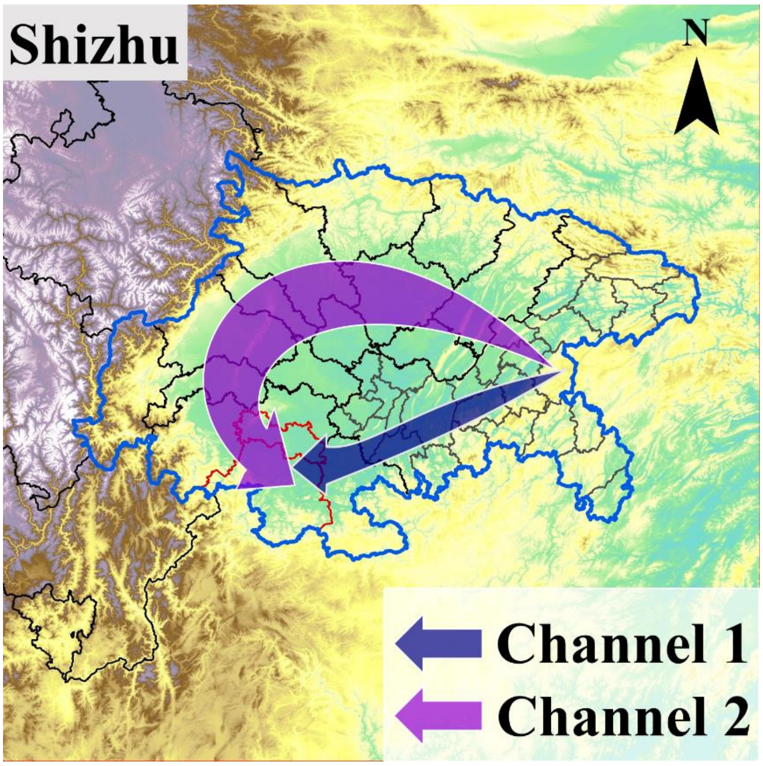

Figure S190 The identified 2 channels originating in Shizhu.

Table S35 Occurrence frequencies of each channel originating in Shizhu in four seasons.

City	Channel	Layer	Season			
			autumn	spring	summer	winter
Shizhu	1	LBL	3.2%			
		UBL	3.2%			
	2	LBL			3.2%	32.3%
		UBL		16.7%	9.7%	29.0%

8.5 Xiushan

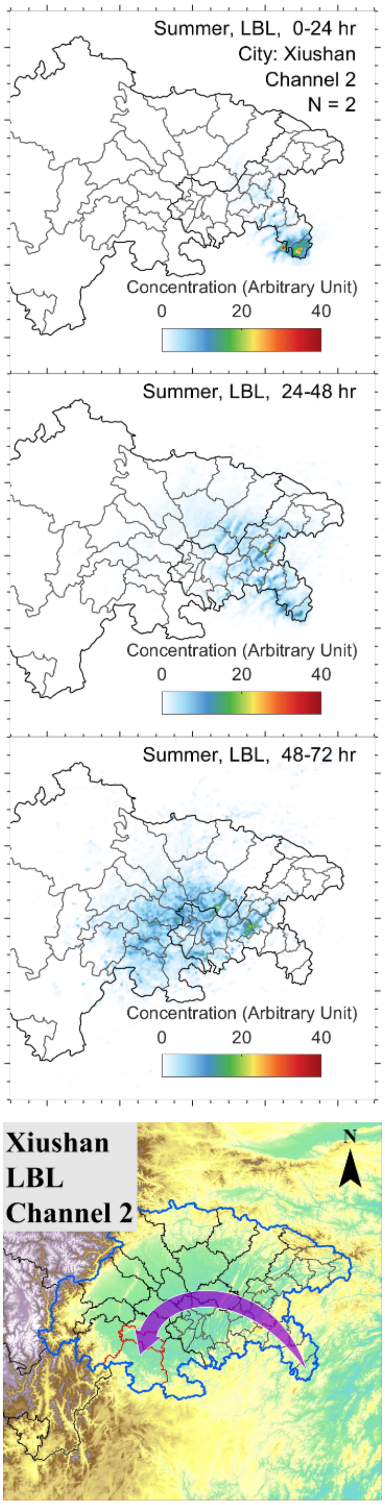

Figure S191 Channel 2 of Xiushan at LBL.

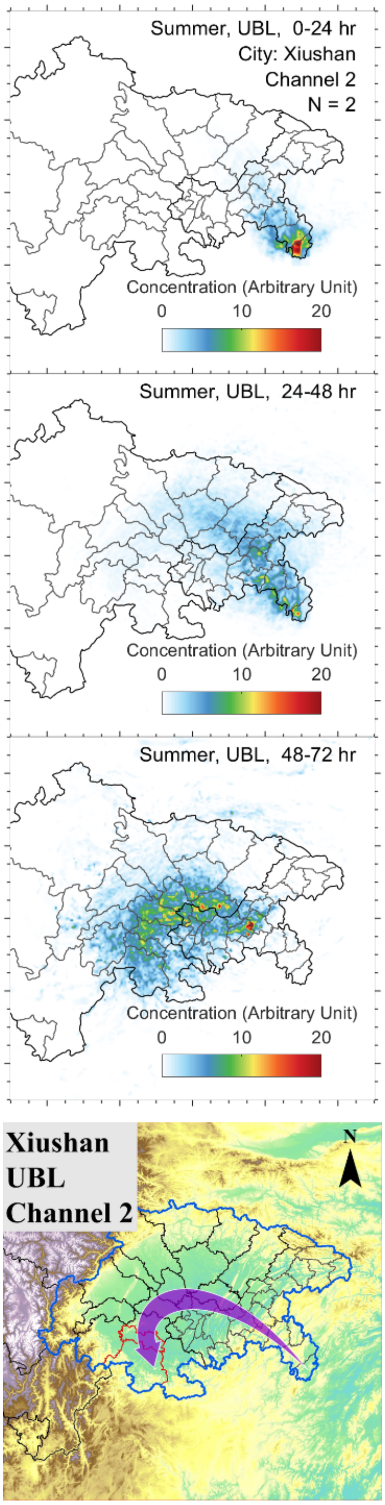

Figure S192 Channel 2 of Xiushan at UBL.

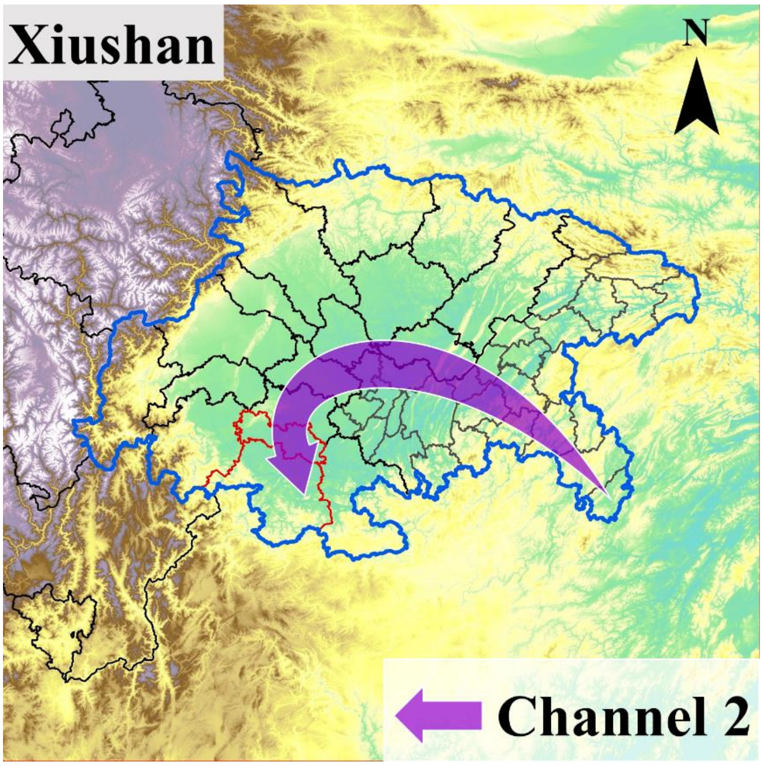

Figure S193 The identified channel originating in Xiushan.

Table S36 Occurrence frequency of the channel originating in Xiushan in four seasons.

City	Channel	Layer	Season			
			autumn	spring	summer	winter
Xiushan	2	LBL			6.5%	
		UBL			6.5%	

9. Northeast Chongqing Urban Agglomeration

9.1 Wanzhou

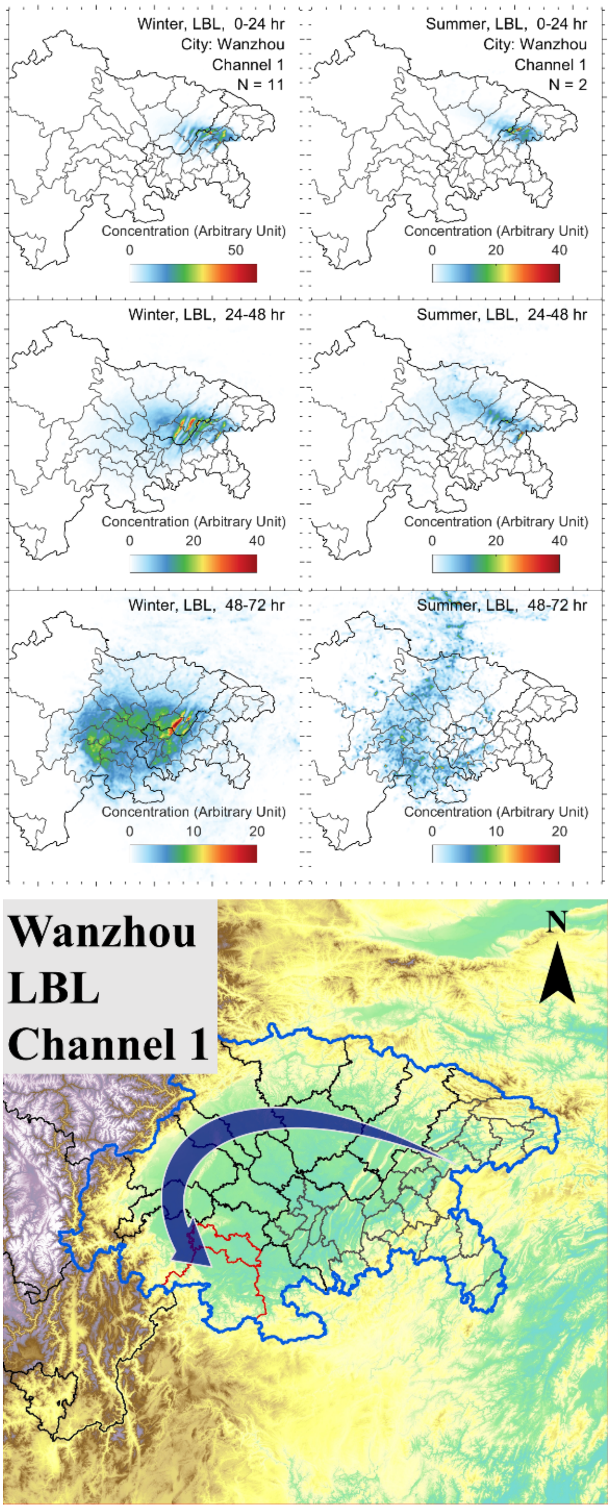

Figure S194 Channel 1 of Wanzhou at LBL.

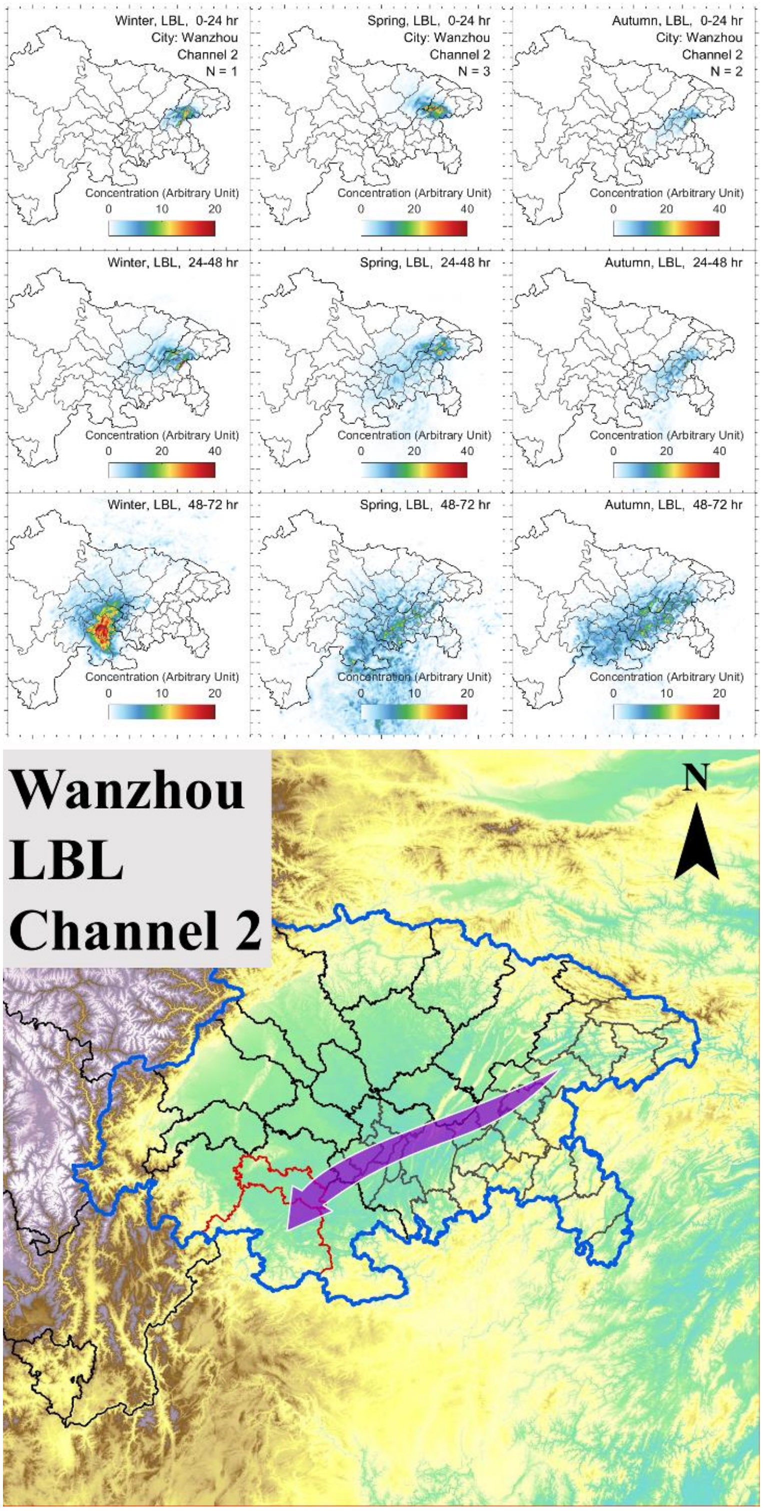

Figure S195 Channel 2 of Wanzhou at LBL.

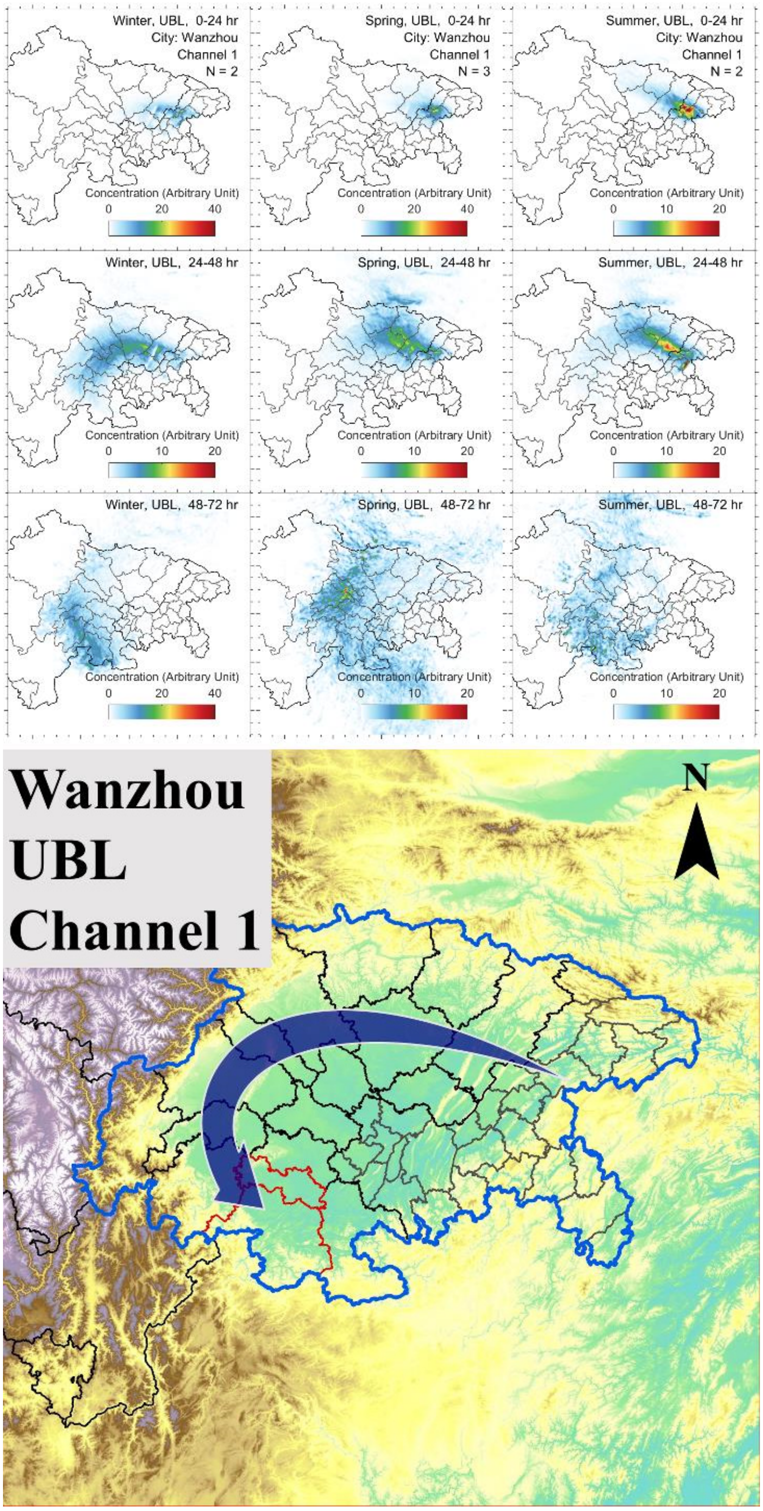

Figure S196 Channel 1 of Wanzhou at UBL.

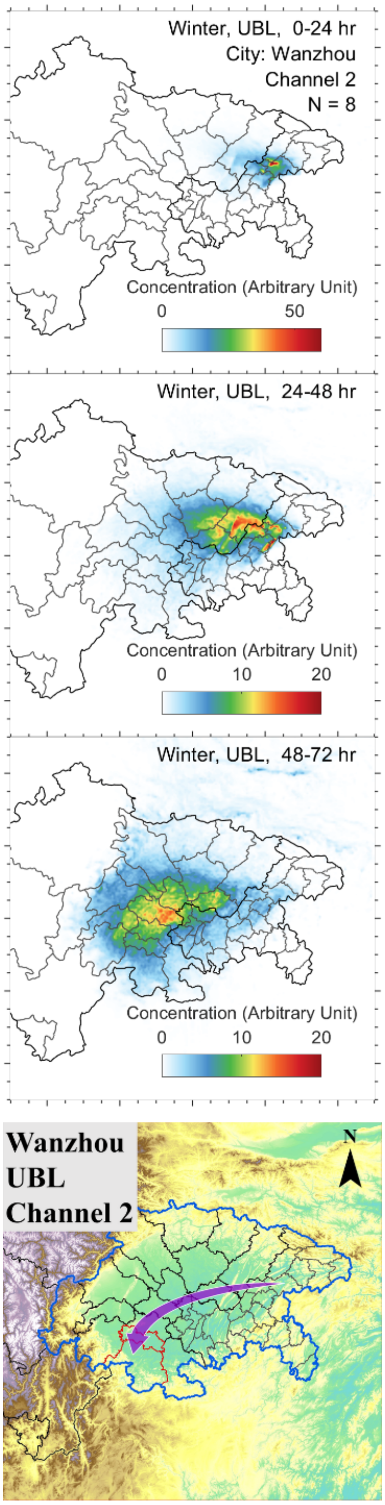

Figure S197 Channel 2 of Wanzhou at UBL.

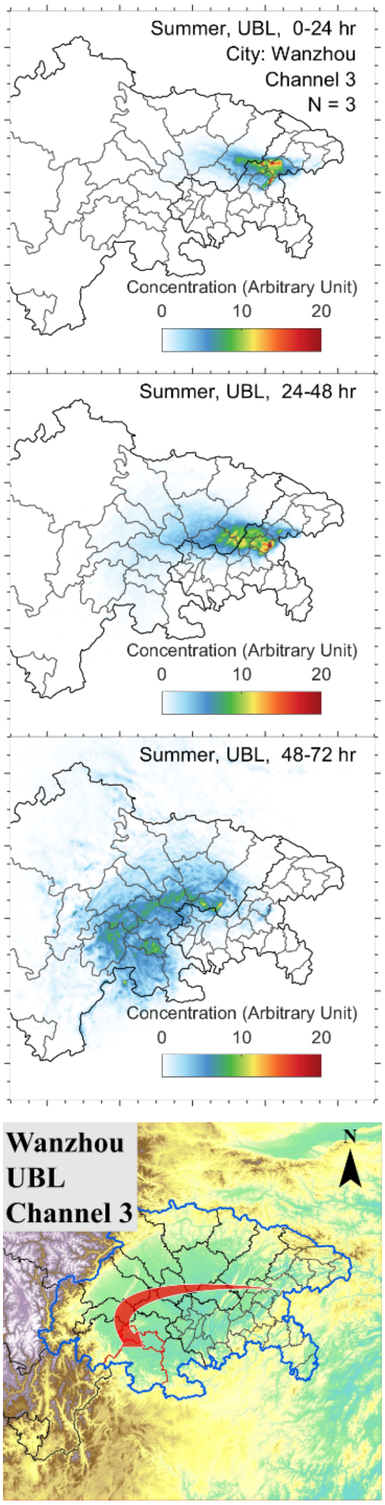

Figure S198 Channel 3 of Wanzhou at UBL.

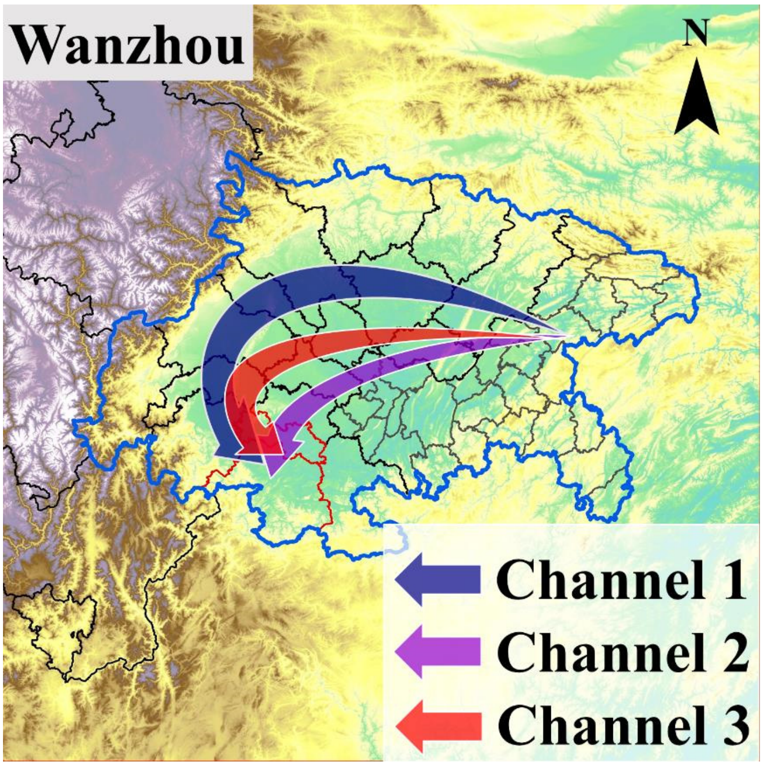

Figure S199 The identified 3 channels originating in Wanzhou.

Table S37 Occurrence frequencies of each channel originating in Wanzhou in four seasons.

City	Channel	Layer	Season			
			autumn	spring	summer	winter
Wanzhou	1	LBL			6.5%	35.5%
		UBL		10.0%	6.5%	6.5%
	2	LBL	6.5%	10.0%		3.2%
		UBL				25.8%
	3	UBL			9.7%	

9.2 Kaizhou

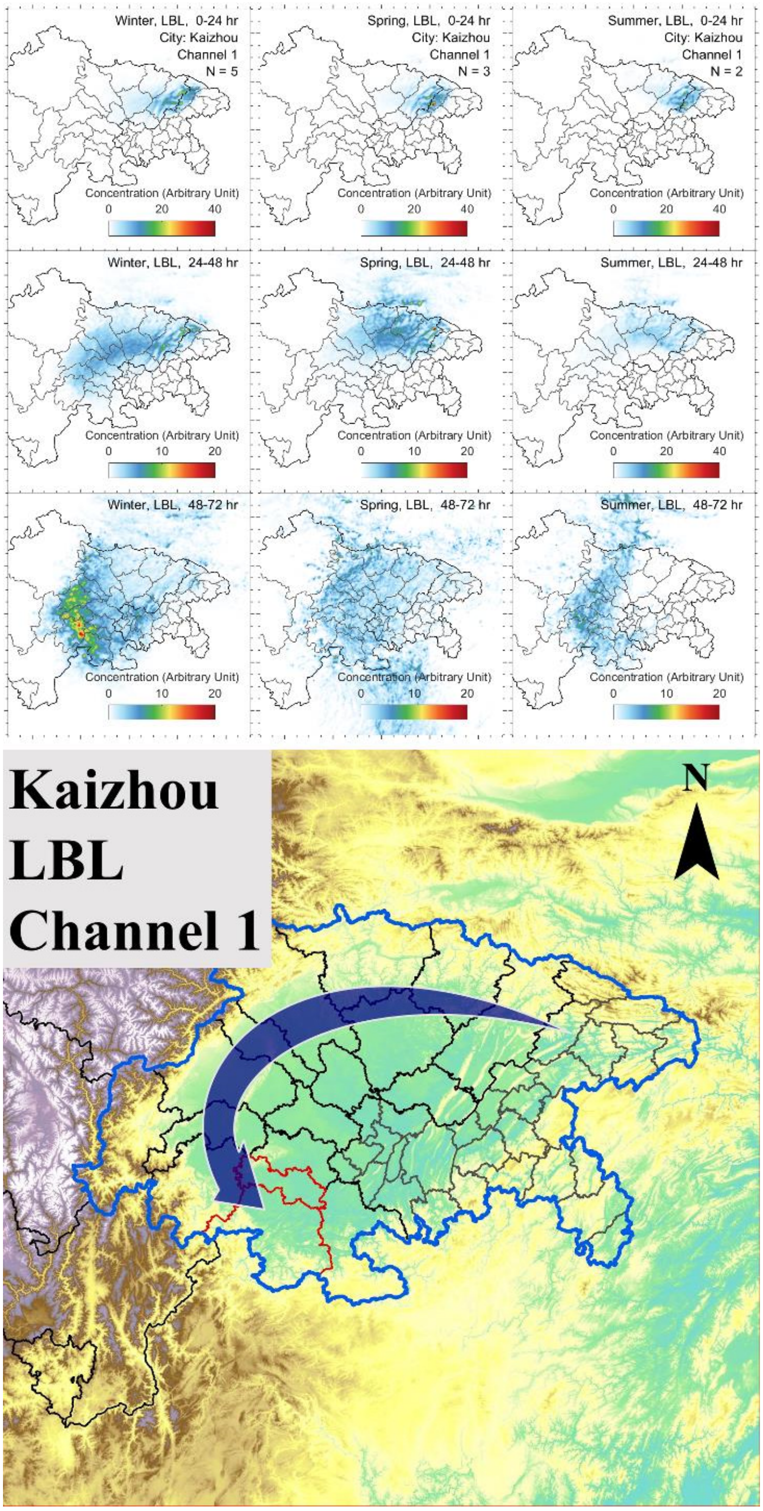

Figure S200 Channel 1 of Kaizhou at LBL.

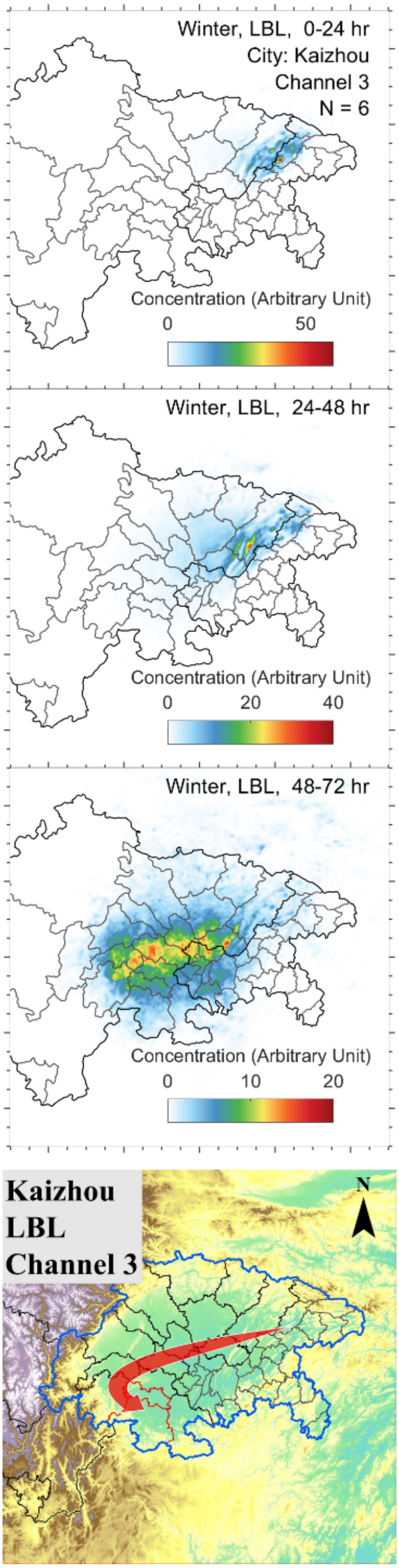

Winter, LBL, 0-24 hr
City: Kaizhou
Channel 3
N = 6
Concentration (Arbitrary Unit)
0
50
Winter, LBL, 24-48 hr
Concentration (Arbitrary Unit)
0
20
40
Winter, LBL, 48-72 hr
Concentration (Arbitrary Unit)
0
10
20
Kaizhou
LBL
Channel 3
N

Figure S201 Channel 3 of Kaizhou at LBL.

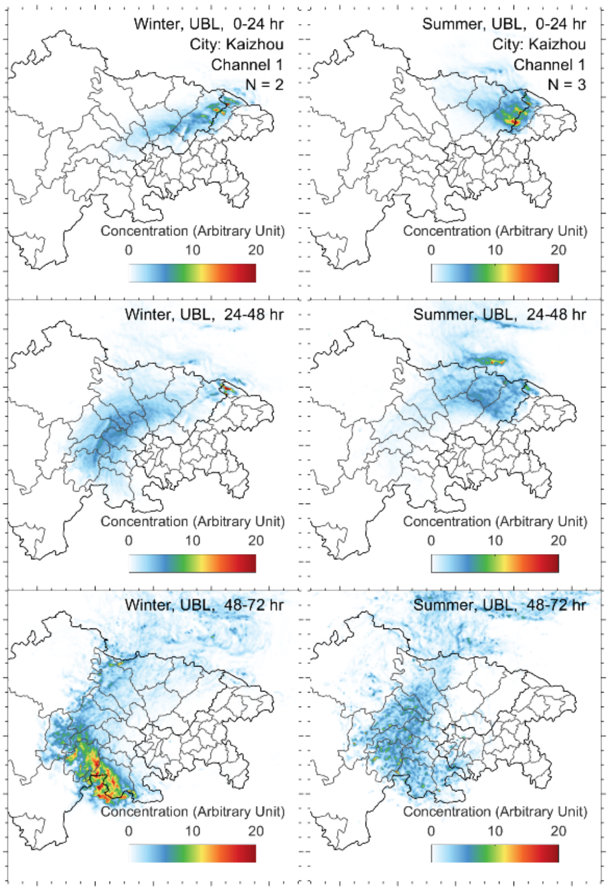

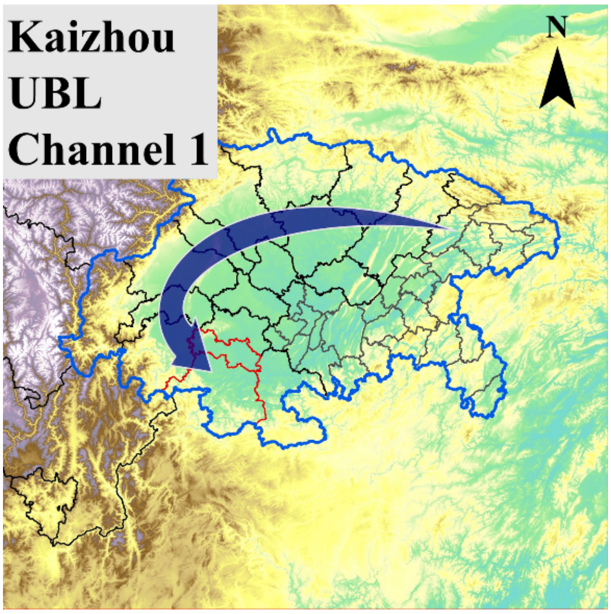

Figure S202 Channel 1 of Kaizhou at UBL.

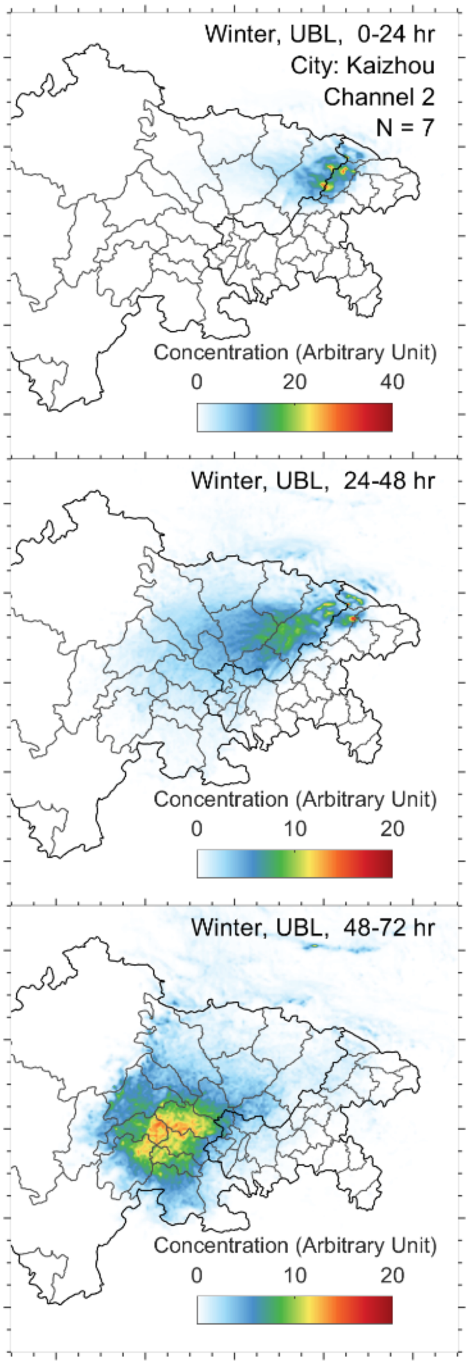
Winter, UBL, 0-24 hr
City: Kaizhou
Channel 2
N = 7
Concentration (Arbitrary Unit)
0
20
40
Winter, UBL, 24-48 hr
Concentration (Arbitrary Unit)
0
10
20
Winter, UBL, 48-72 hr
Concentration (Arbitrary Unit)
0
10
20

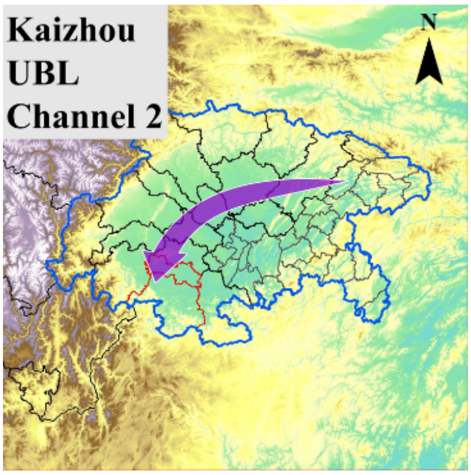
Kaizhou
UBL
Channel 2
N

Figure S203 Channel 2 of Kaizhou at UBL.

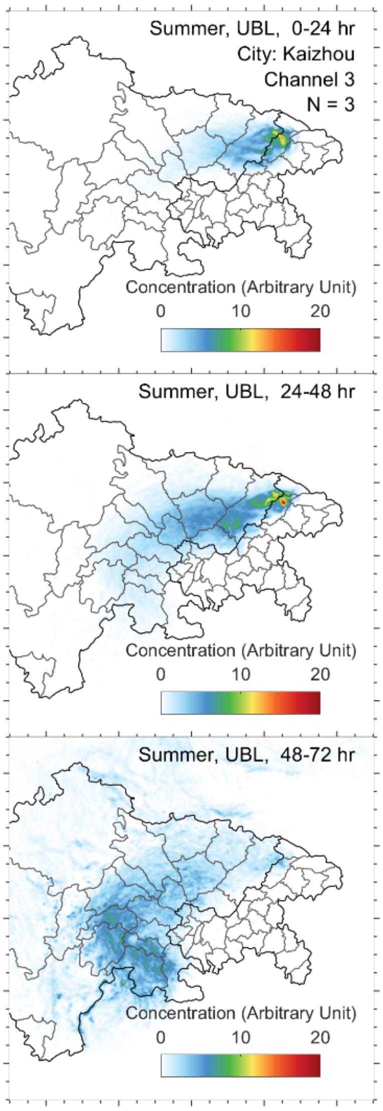

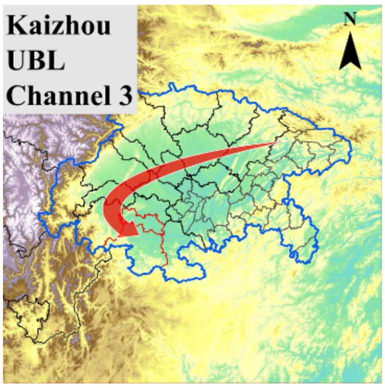

Figure S204 Channel 3 of Kaizhou at UBL.

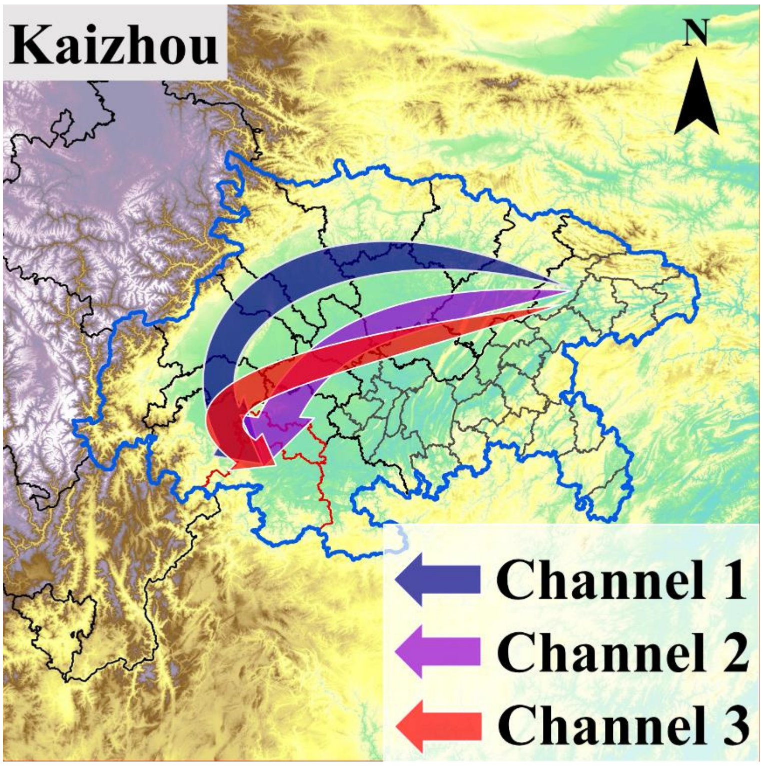

Figure S205 The identified 3 channels originating in Kaizhou.

Table S38 Occurrence frequencies of each channel originating in Kaizhou in four seasons.

City	Channel	Layer	Season			
			autumn	spring	summer	winter
Kaizhou	1	LBL		10.0%	6.5%	16.1%
		UBL		10.0%	9.7%	6.5%
	2	UBL				22.6%
	3	LBL				19.4%
		UBL			9.7%	

9.3 Chengkou

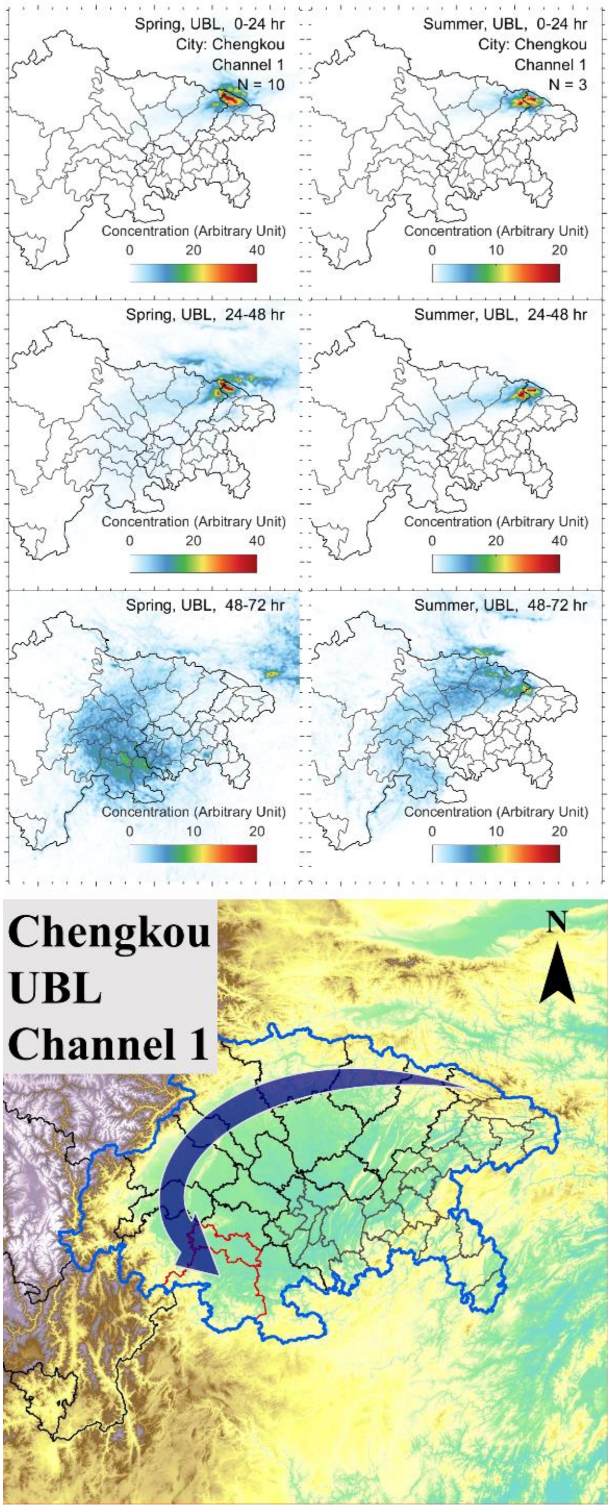

Figure S206 Channel 1 of Chengkou at UBL.

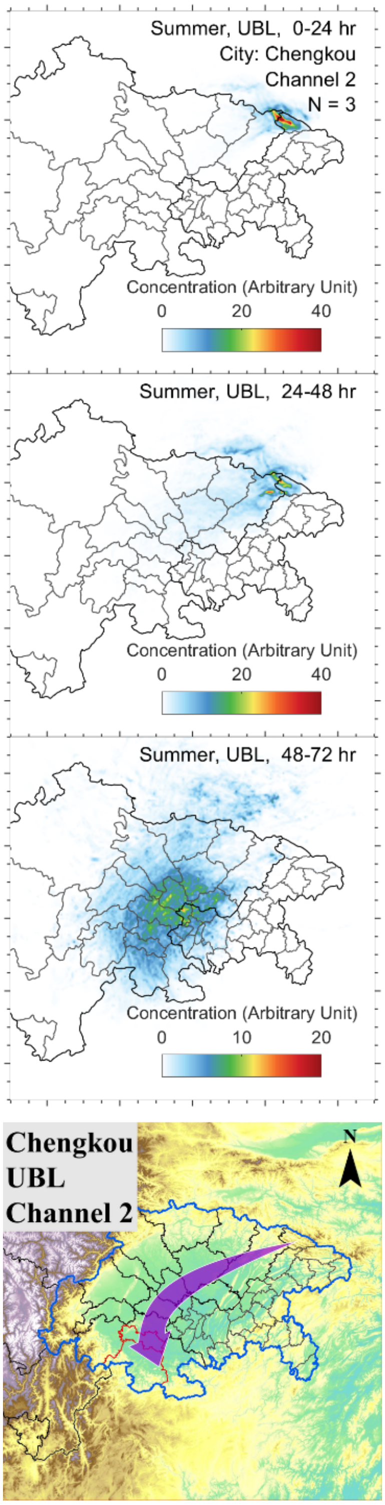

Figure S207 Channel 2 of Chengkou at UBL.

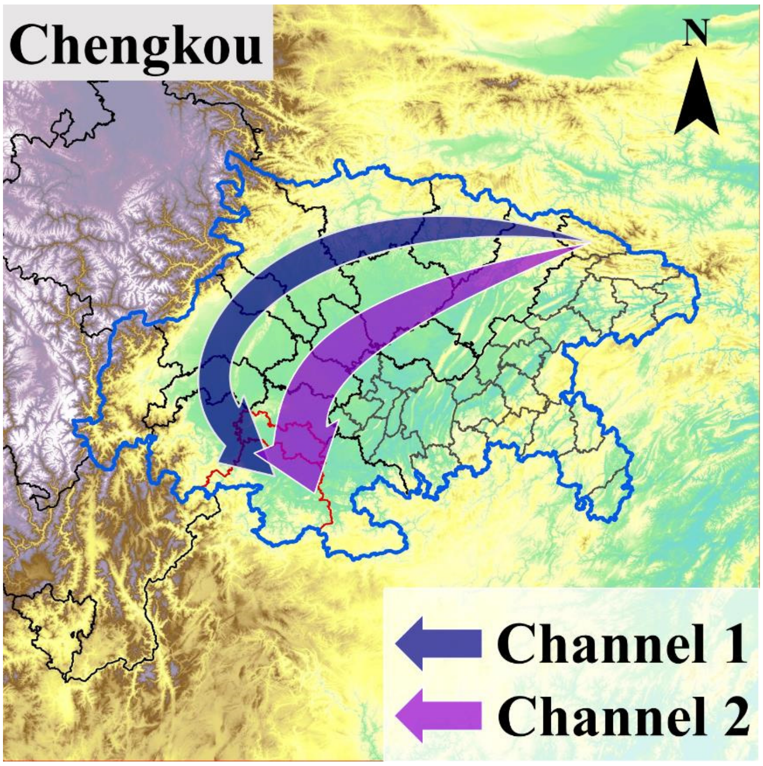

Figure S208 The identified 2 channels originating in Chengkou.

Table S39 Occurrence frequencies of each channel originating in Chengkou in four seasons.

City	Channel	Layer	Season			
			autumn	spring	summer	winter
Chengkou	1	UBL		33.3%	9.7%	
	2	UBL			9.7%	

9.4 Wuxi

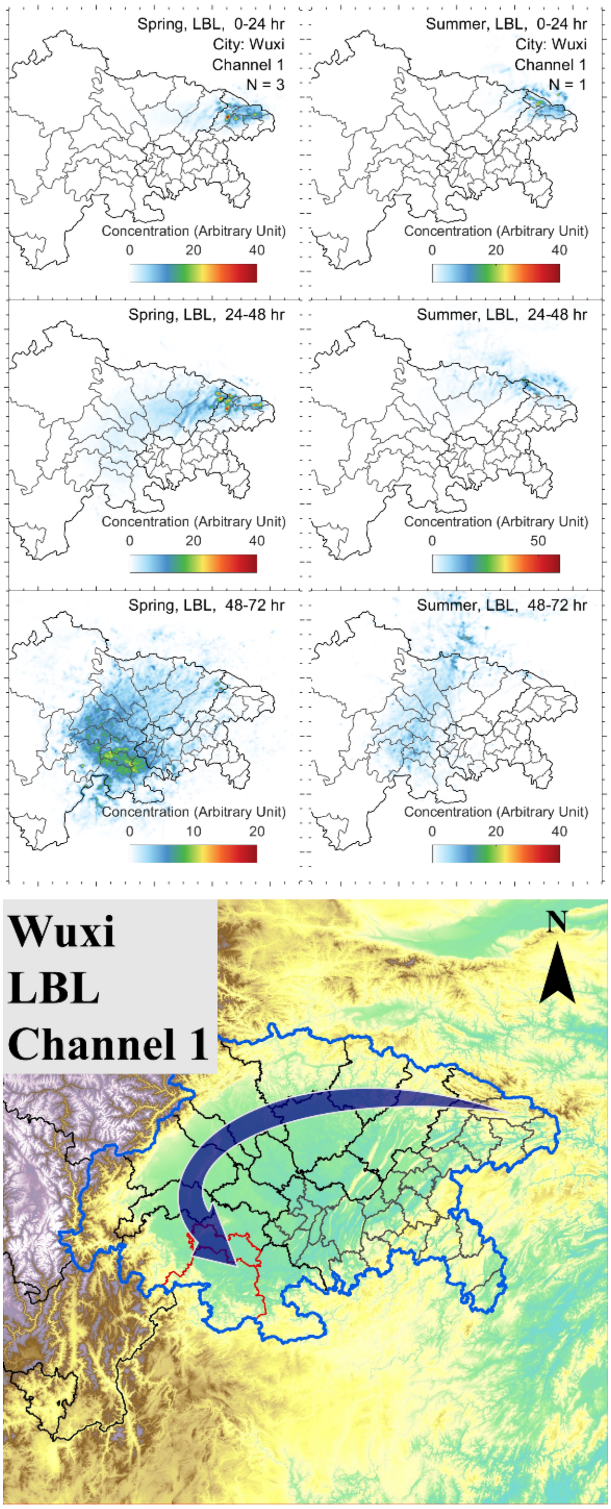

Figure S209 Channel 1 of Wuxi at LBL.

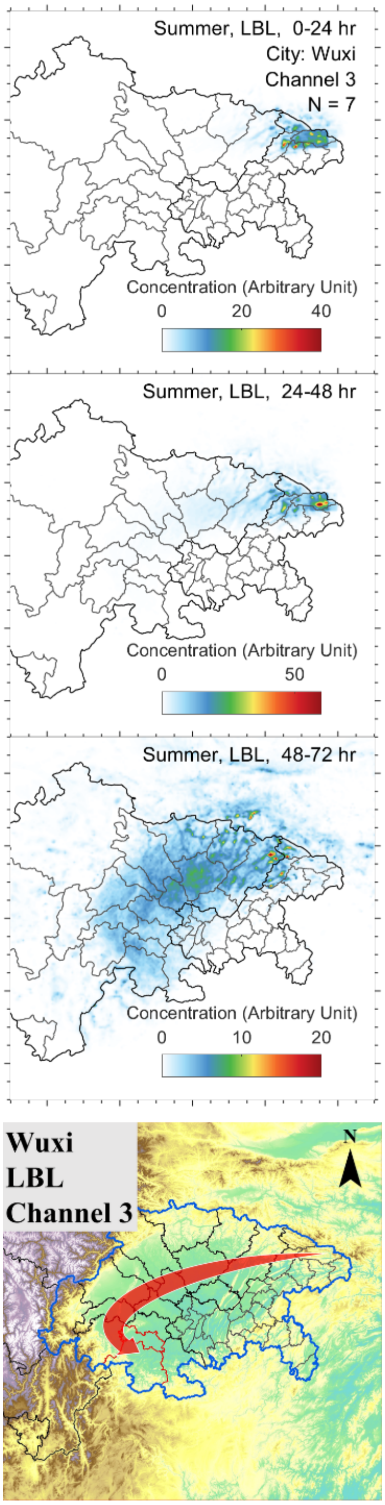

Figure S210 Channel 3 of Wuxi at LBL.

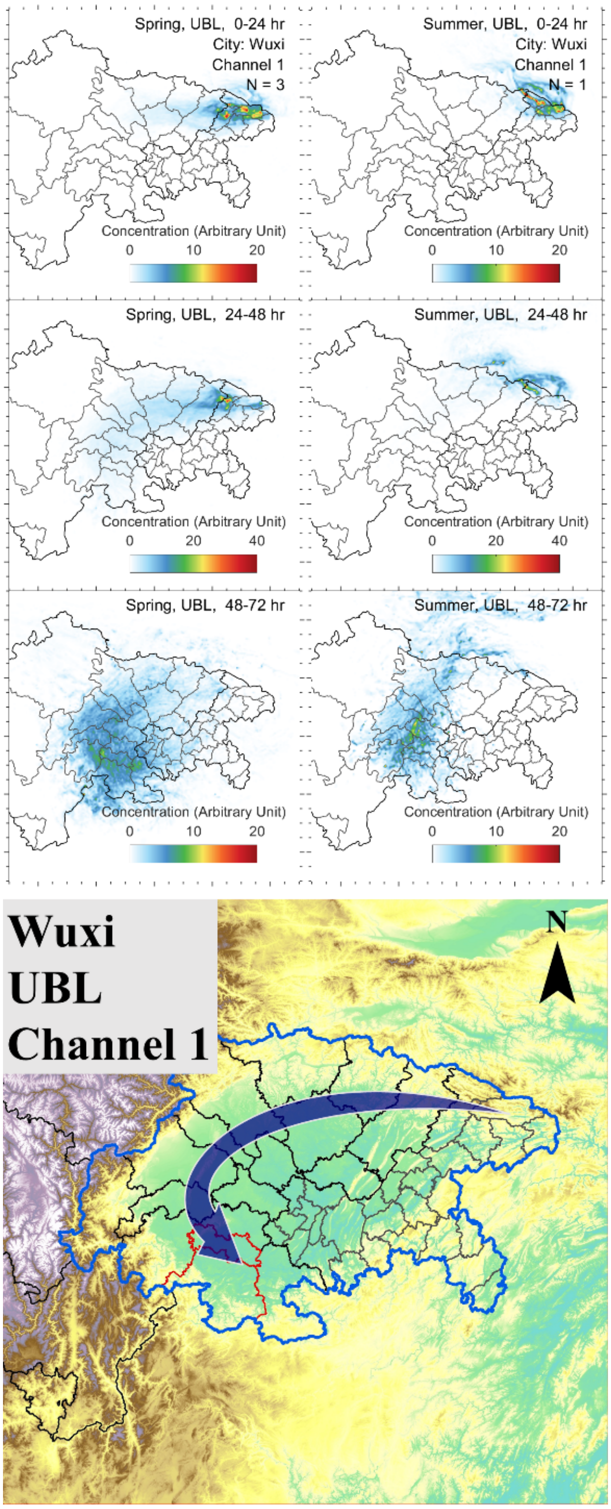

Figure S211 Channel 1 of Wuxi at UBL.

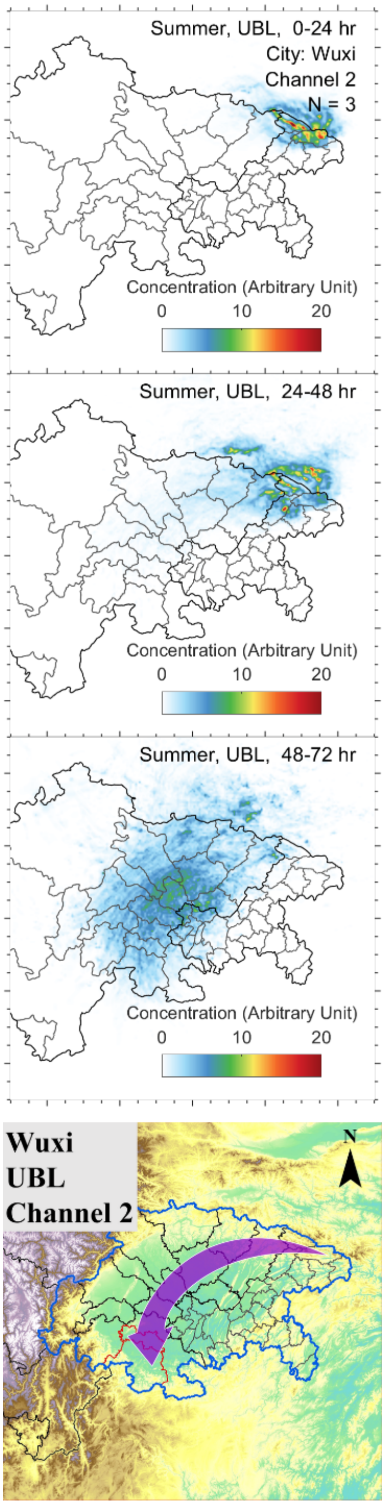

Figure S212 Channel 2 of Wuxi at UBL.

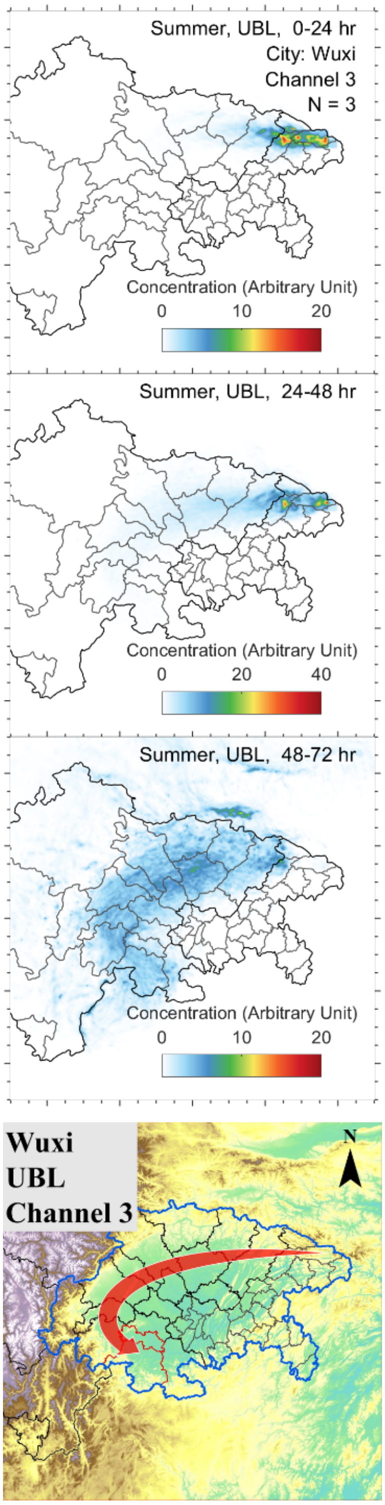

Figure S213 Channel 3 of Wuxi at UBL.

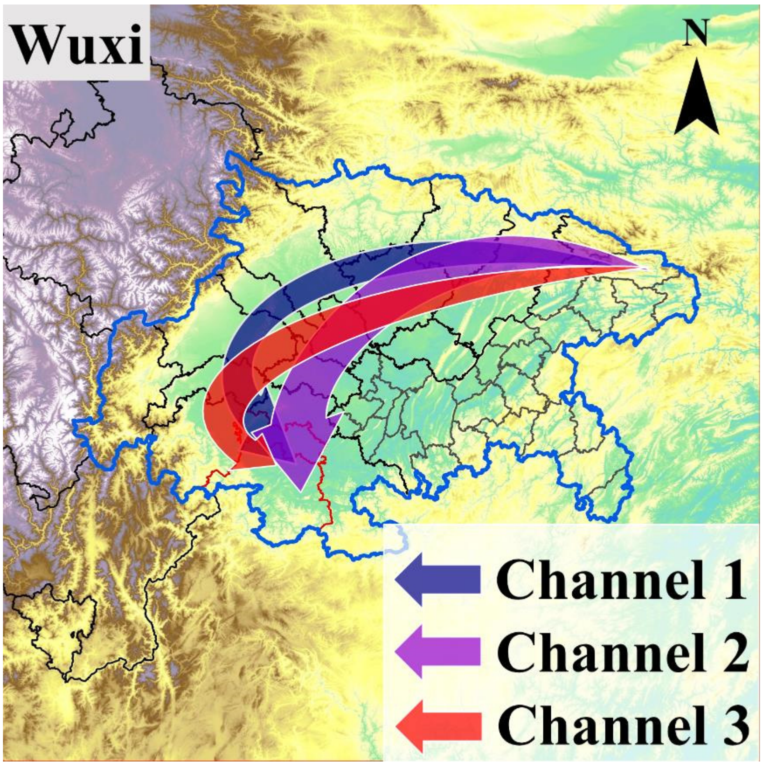

Figure S214 The identified 3 channels originating in Wuxi.

Table S40 Occurrence frequencies of each channel originating in Wuxi in four seasons.

City	Channel	Layer	Season			
			autumn	spring	summer	winter
Wuxi	1	LBL		10.0%	3.2%	
		UBL		10.0%	3.2%	
	2	UBL			9.7%	
	3	LBL			22.6%	
		UBL			9.7%	

9.5 Yunyang

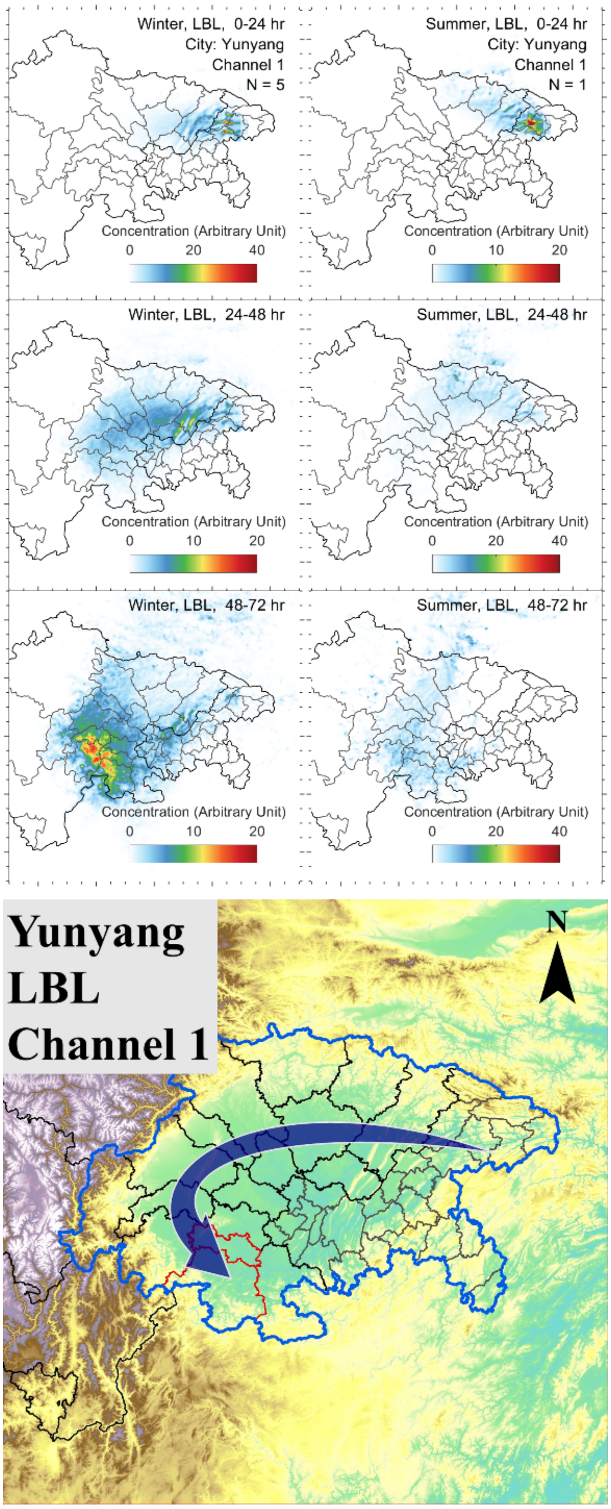

Figure S215 Channel 1 of Yunyang at LBL.

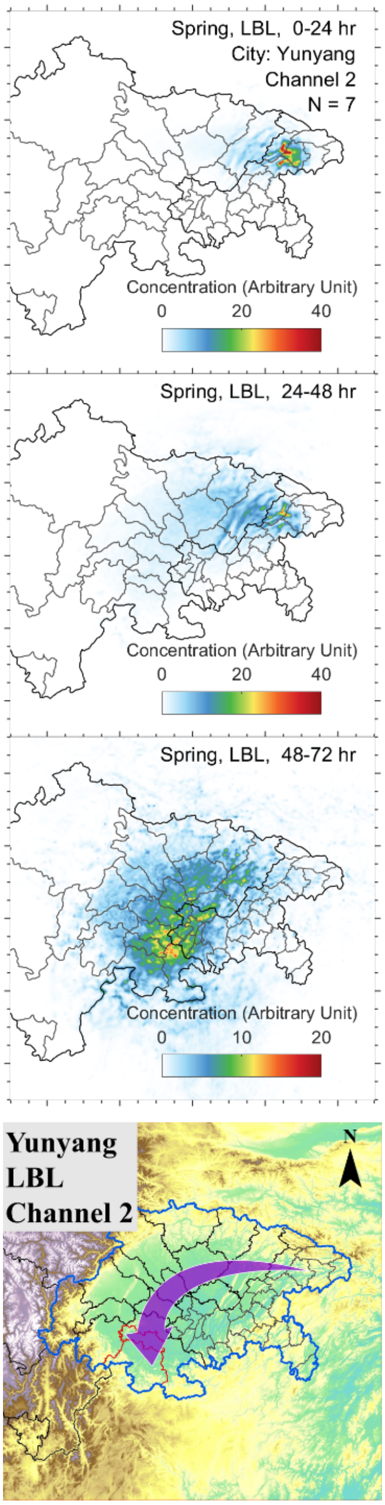

Figure S216 Channel 2 of Yunyang at LBL.

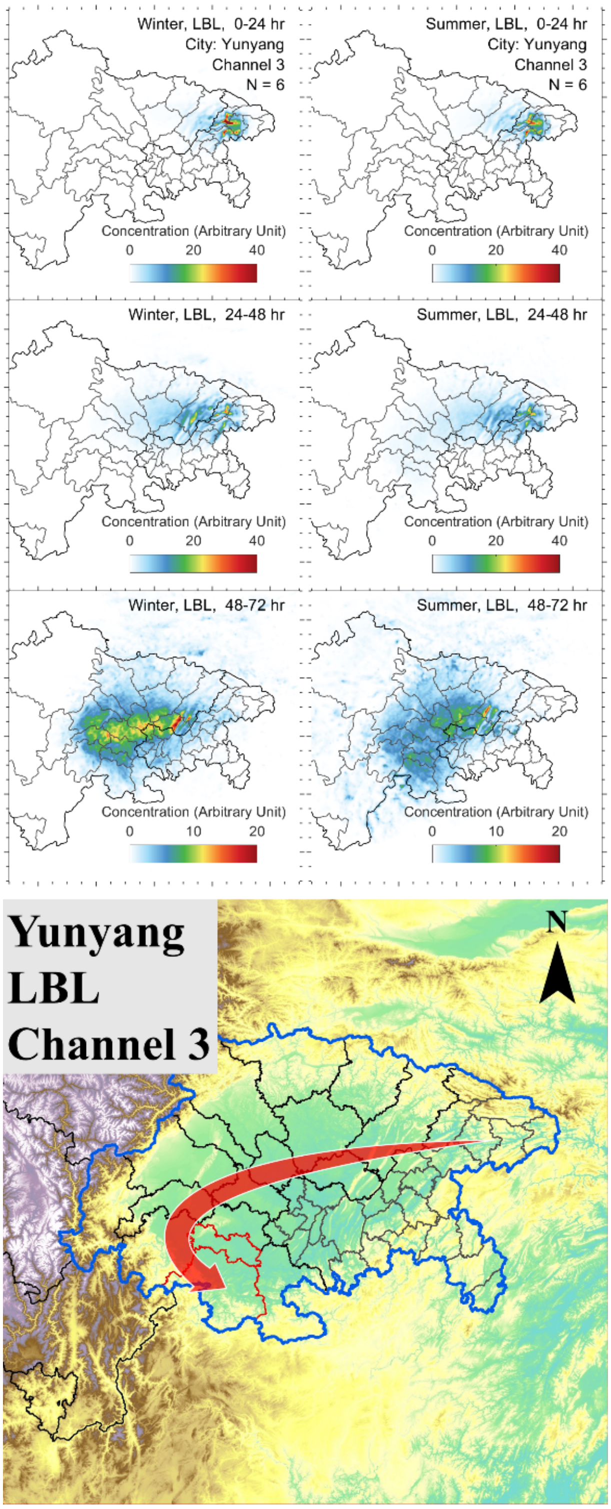

Figure S217 Channel 3 of Yunyang at LBL.

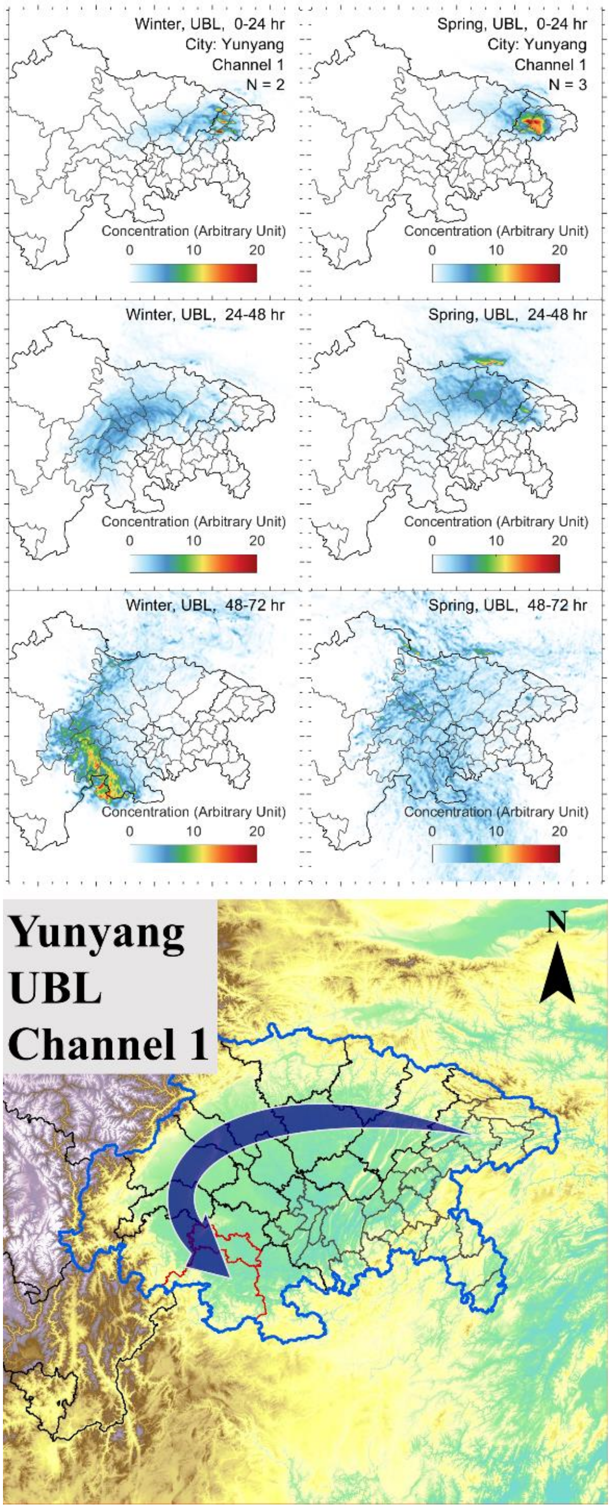

Figure S218 Channel 1 of Yunyang at UBL.

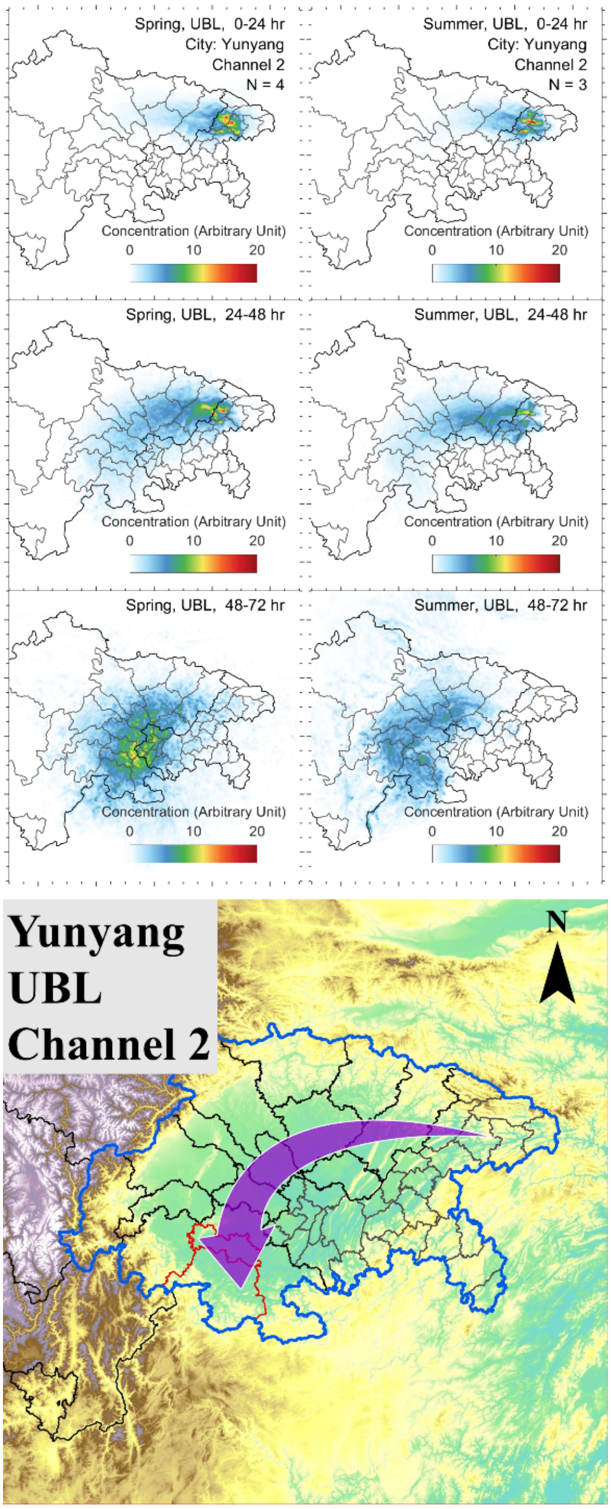

Figure S219 Channel 2 of Yunyang at UBL.

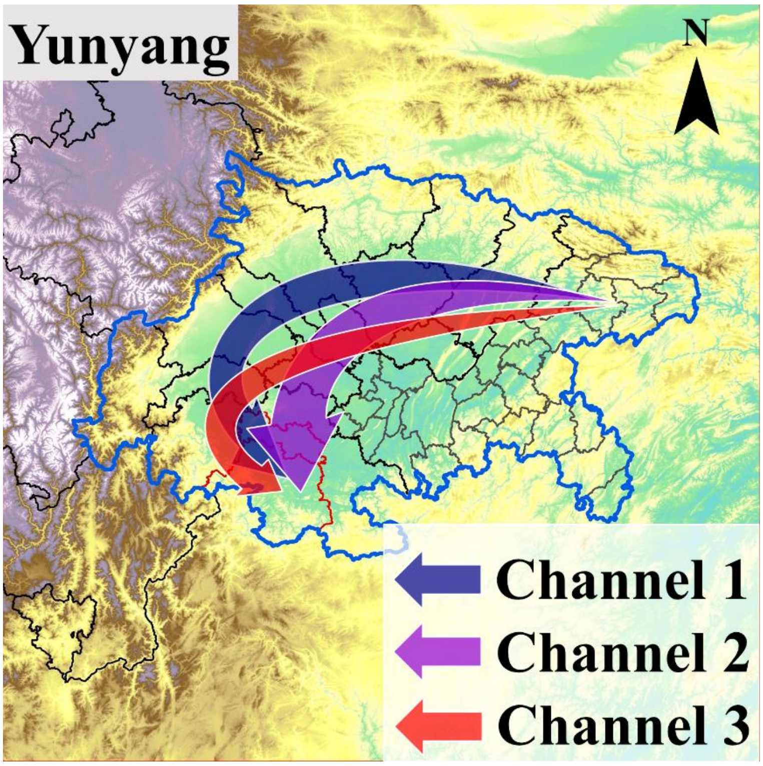

Figure S220 The identified 3 channels originating in Yunyang.

Table S41 Occurrence frequencies of each channel originating in Yunyang in four seasons.

City	Channel	Layer	Season			
			autumn	spring	summer	winter
Yunyang	1	LBL			3.2%	16.1%
		UBL		10.0%		6.5%
	2	LBL		23.3%		
		UBL		13.3%	9.7%	
	3	LBL			19.4%	19.4%

9.6 Fengjie

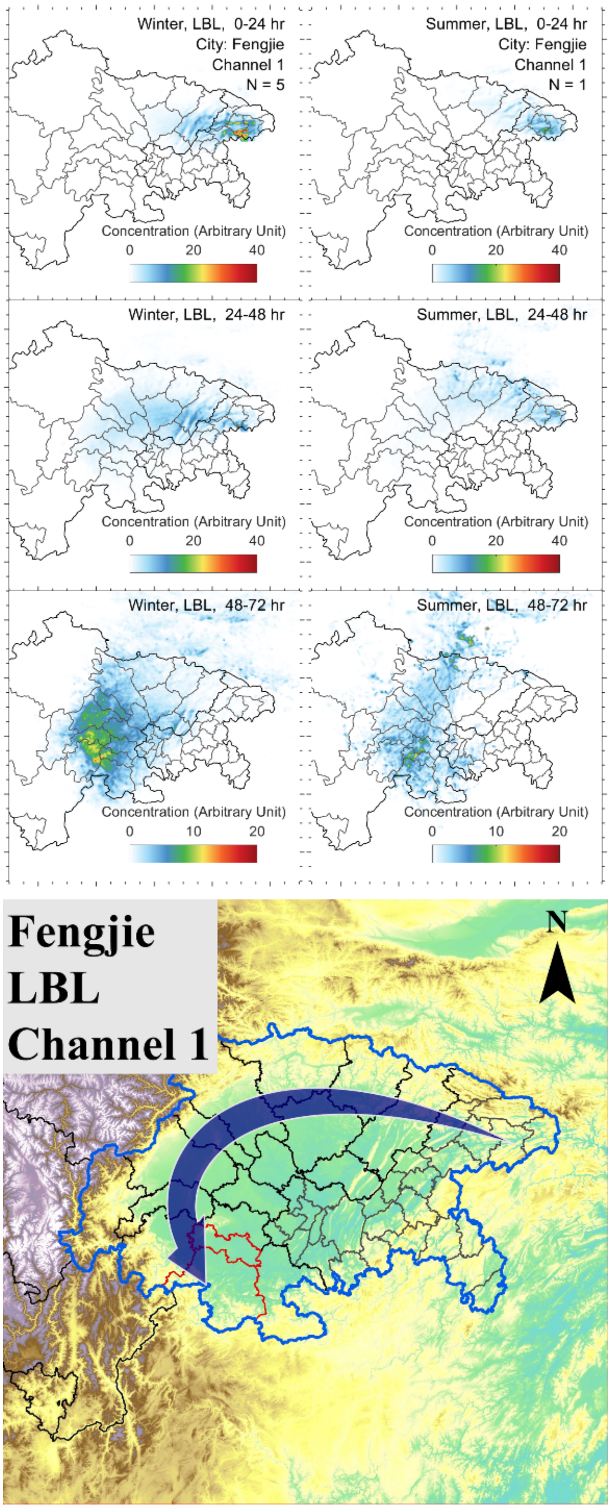

Figure S221 Channel 1 of Fengjie at LBL.

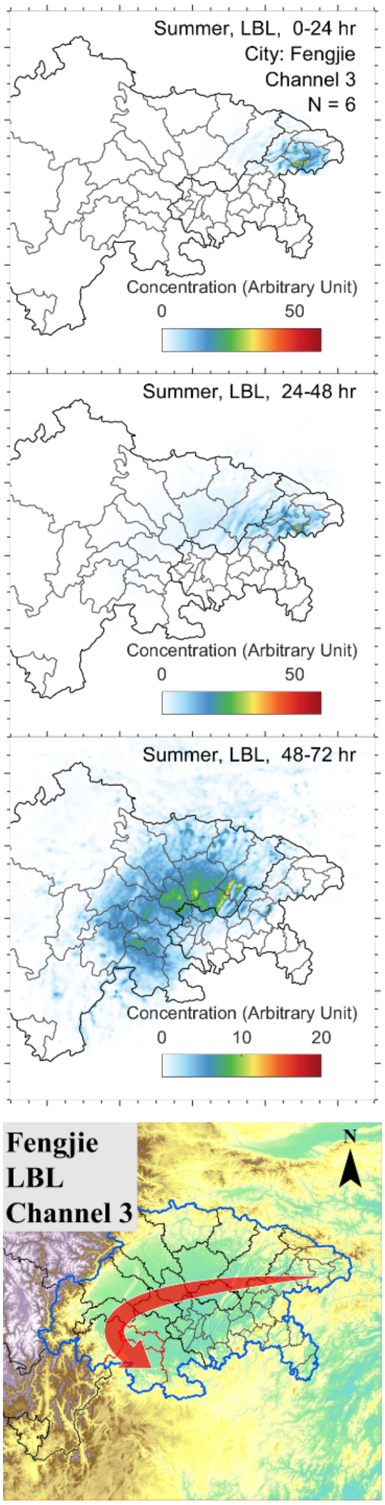

Figure S222 Channel 3 of Fengjie at LBL.

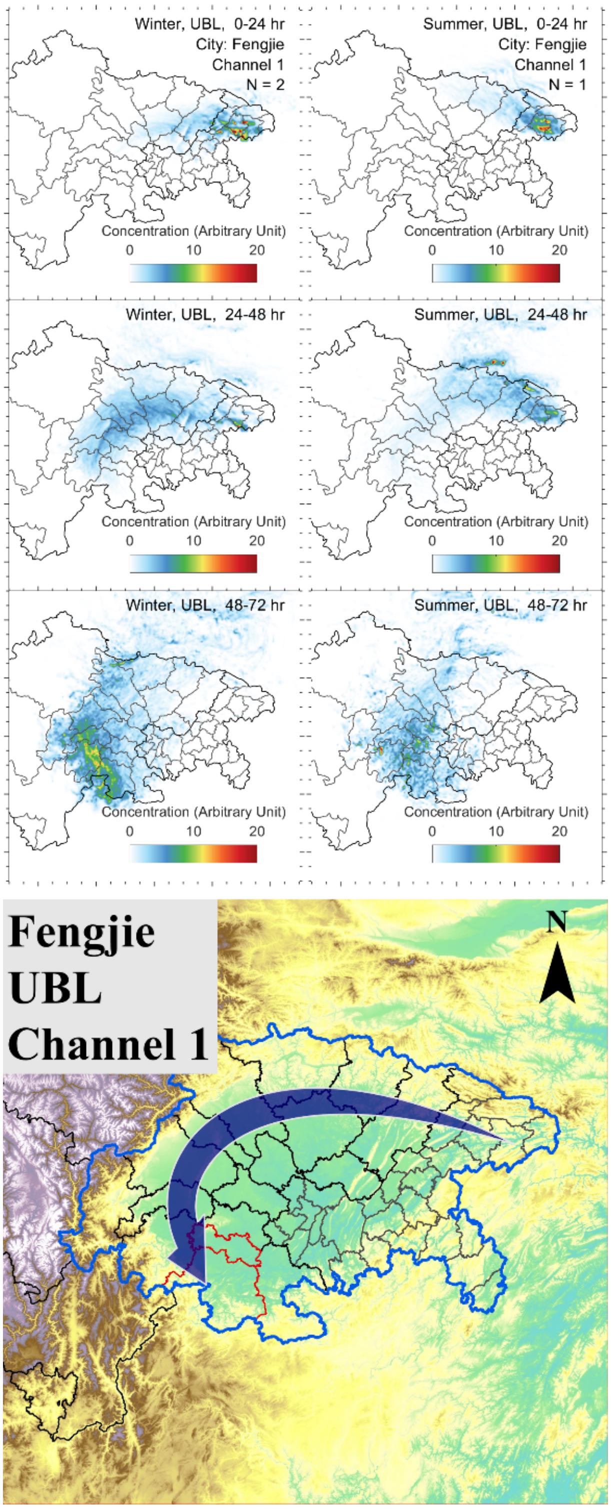

Figure S223 Channel 1 of Fengjie at UBL.

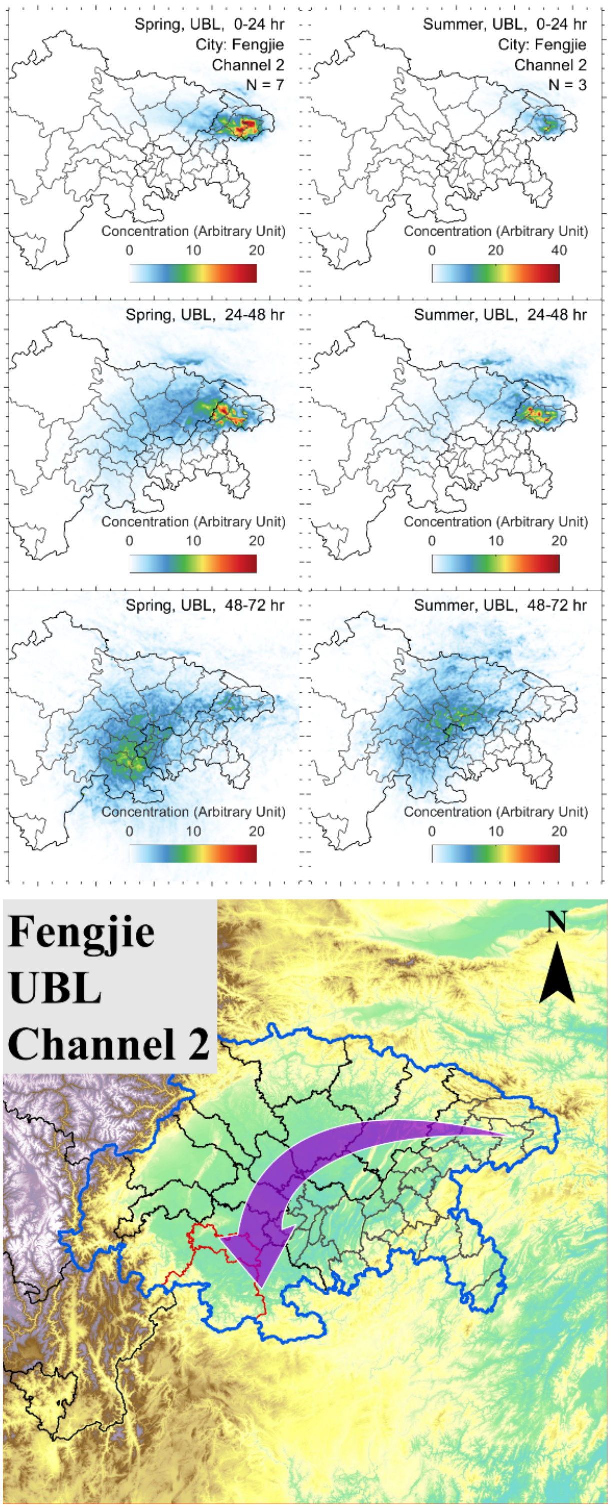

Figure S224 Channel 2 of Fengjie at UBL.

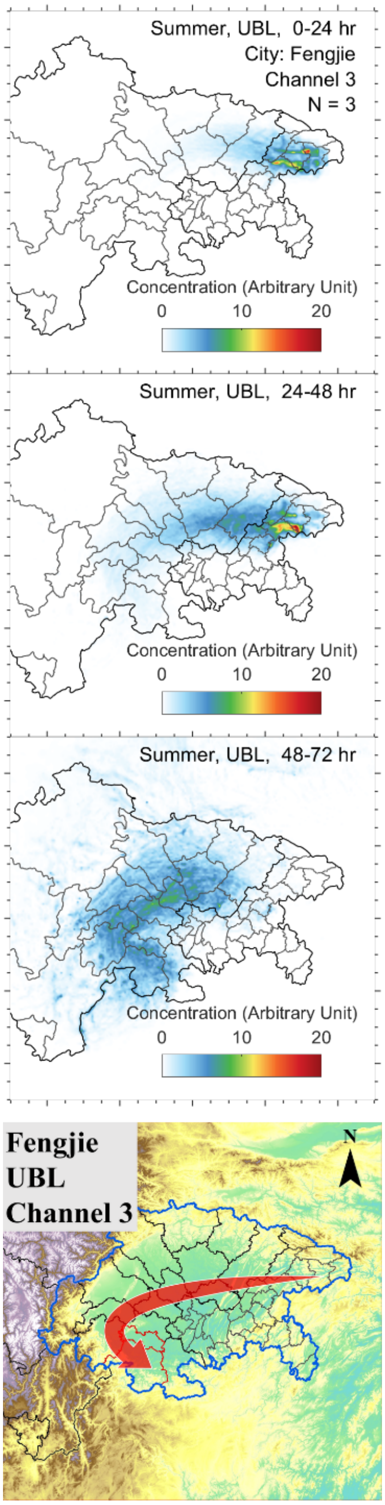

Figure S225 Channel 3 of Fengjie at UBL.

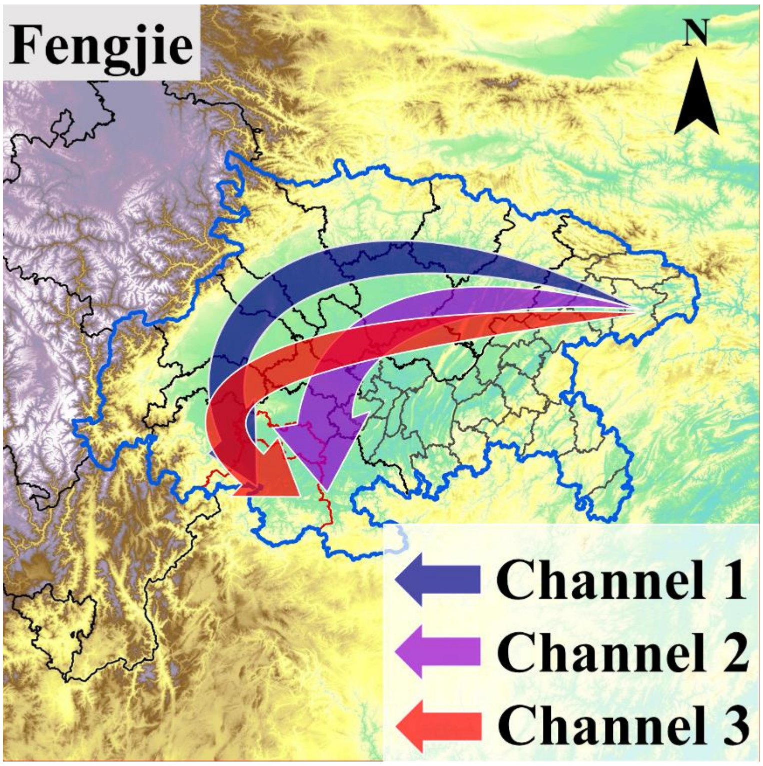

Figure S226 The identified 3 channels originating in Fengjie.

Table S42 Occurrence frequencies of each channel originating in Fengjie in four seasons.

City	Channel	Layer	Season			
			autumn	spring	summer	winter
Fengjie	1	LBL			3.2%	16.1%
		UBL			3.2%	6.5%
	2	UBL		23.3%	9.7%	
	3	LBL			19.4%	
		UBL			9.7%	

9.7 Wushan

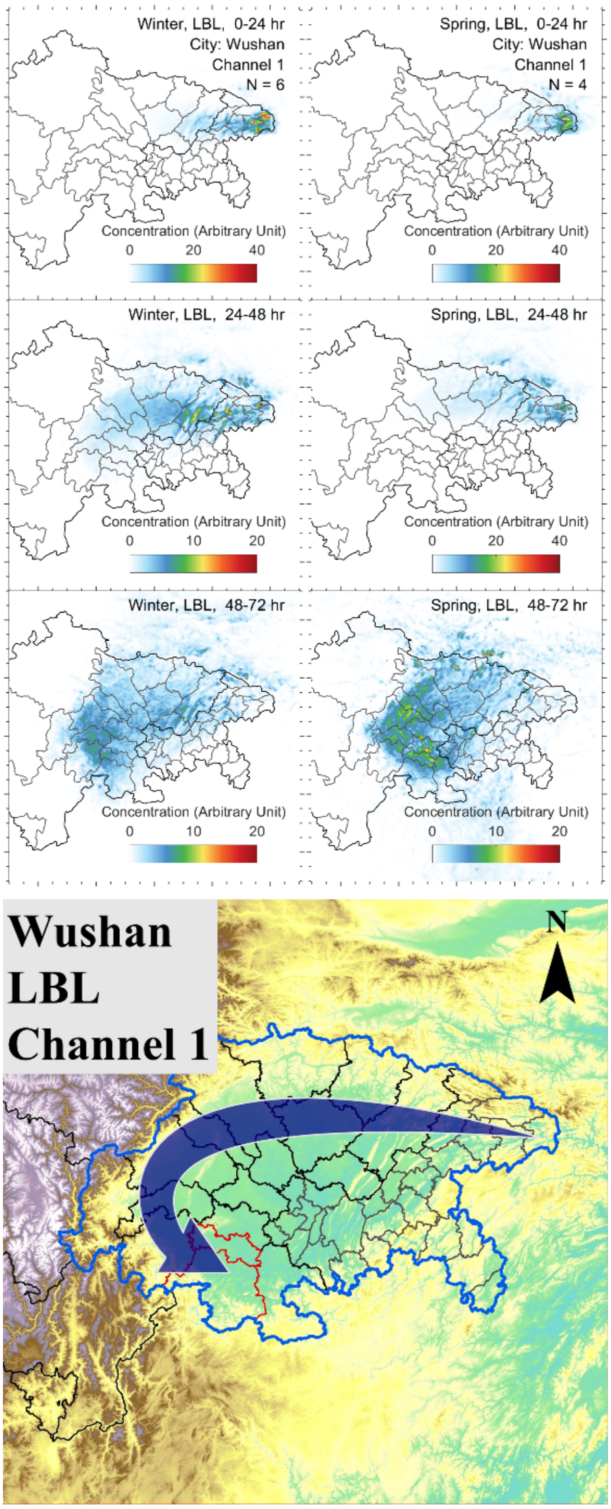

Figure S227 Channel 1 of Wushan at LBL.

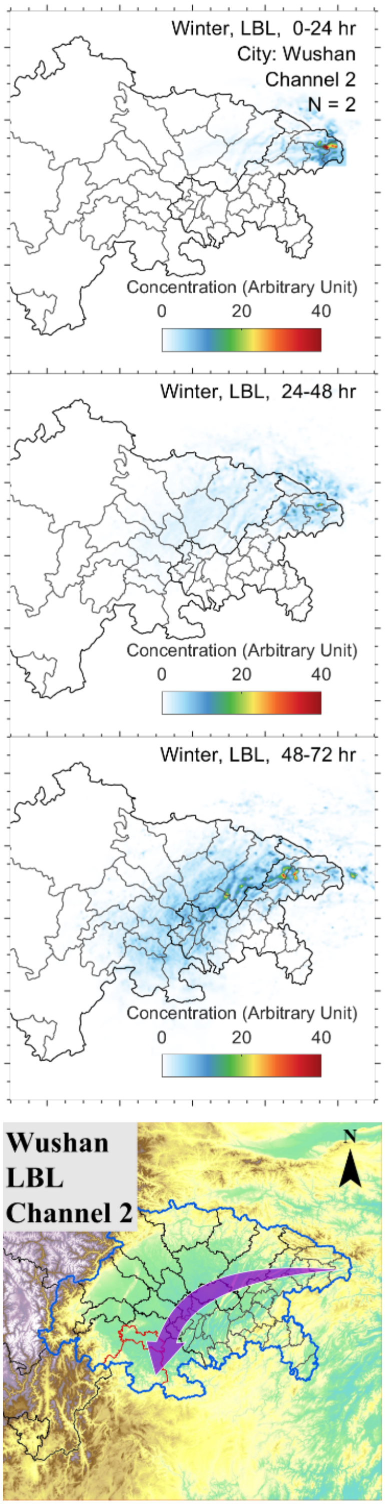

Figure S228 Channel 2 of Wushan at LBL.

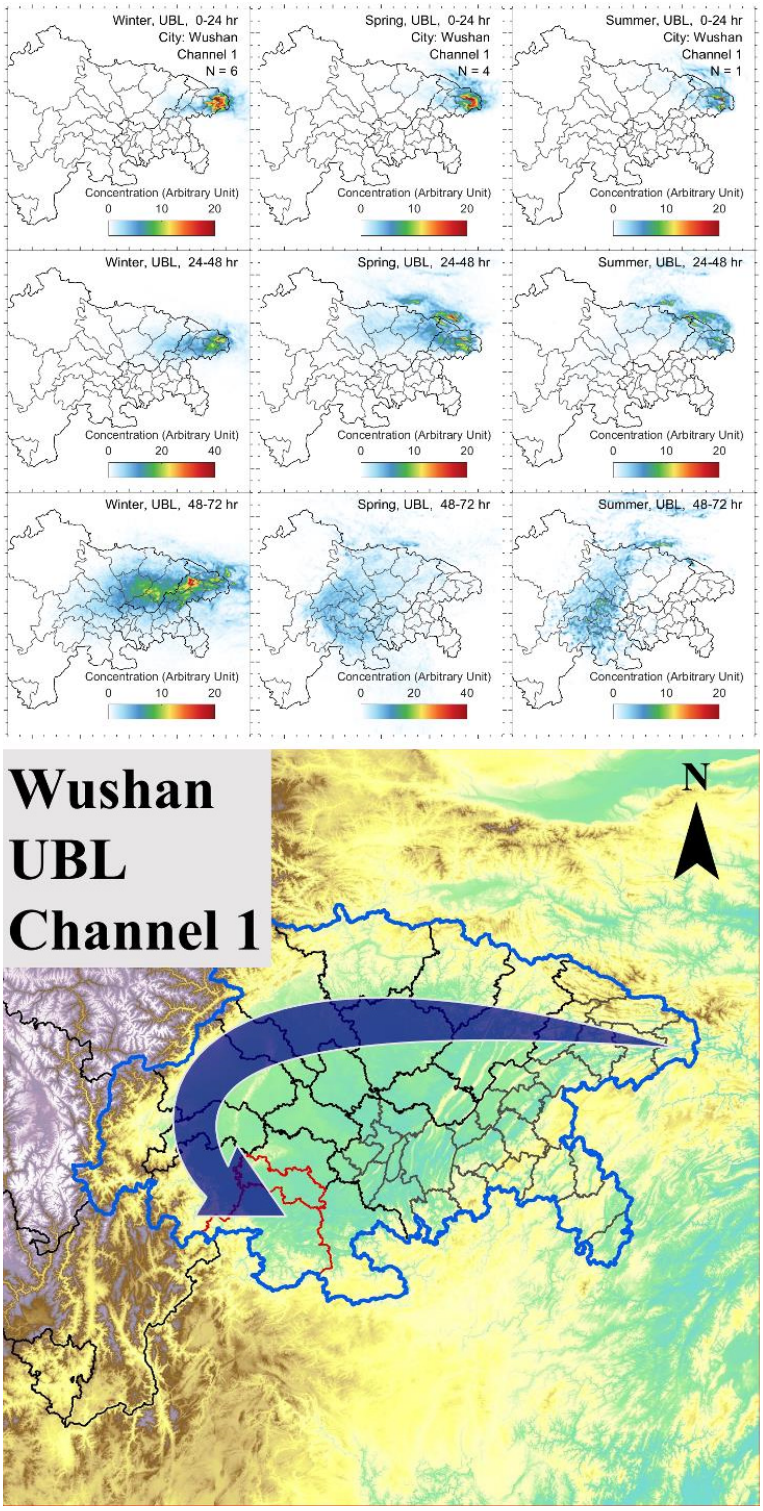

Figure S229 Channel 1 of Wushan at UBL.

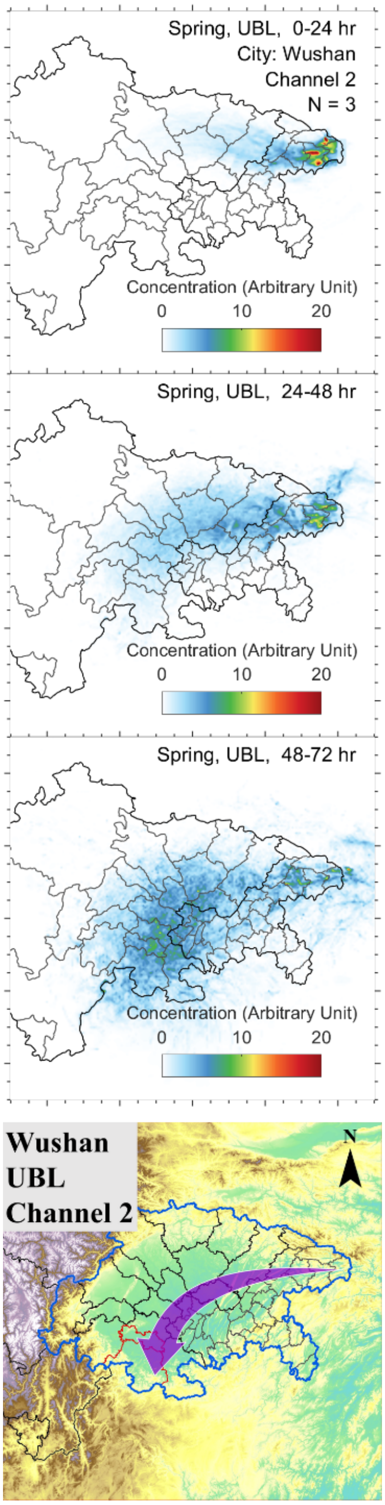

Figure S230 Channel 2 of Wushan at UBL.

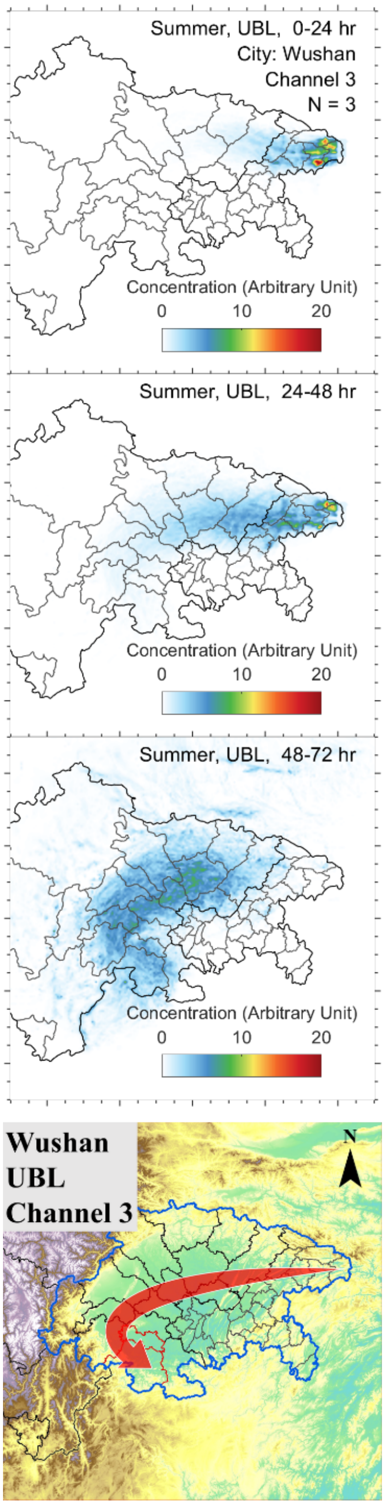

Figure S231 Channel 3 of Wushan at UBL.

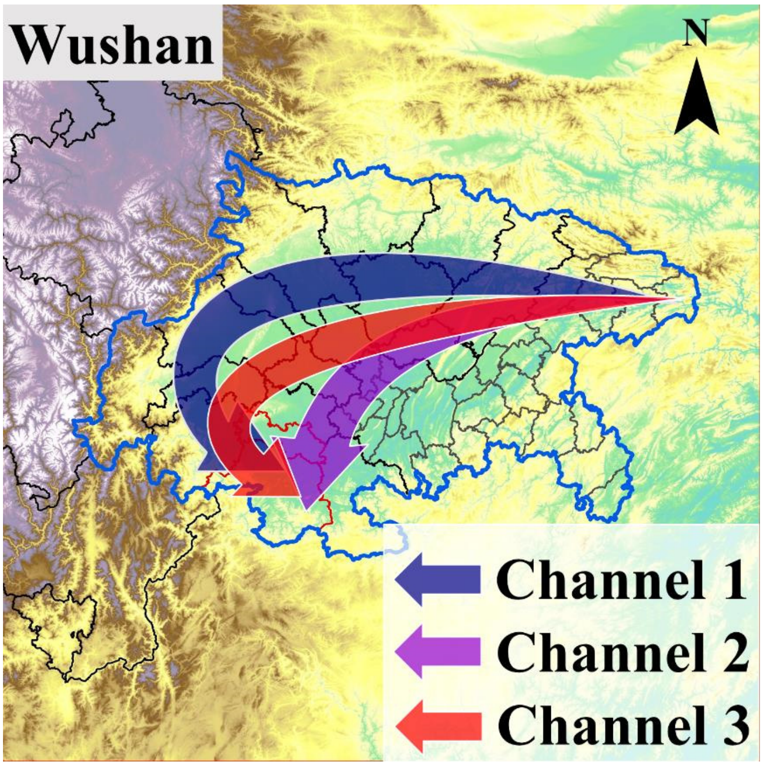

Figure S232 The identified 3 channels originating in Wushan.

Table S43 Occurrence frequencies of each channel originating in Wushan in four seasons.

City	Channel	Layer	Season			
			autumn	spring	summer	winter
Wushan	1	LBL		13.3%		19.4%
		UBL		13.3%	3.2%	19.4%
	2	LBL				6.5%
		UBL		10.0%		
	3	UBL			9.7%	
